# microRNAs and the evolution of complex multicellularity: identification of a large, diverse complement of microRNAs in the brown alga *Ectocarpus*

**DOI:** 10.1093/nar/gkv578

**Published:** 2015-06-22

**Authors:** James E. Tarver, Alexandre Cormier, Natalia Pinzón, Richard S. Taylor, Wilfrid Carré, Martina Strittmatter, Hervé Seitz, Susana M. Coelho, J. Mark Cock

**Affiliations:** 1 School of Earth Sciences, University of Bristol, Life Sciences Building, 24 Tyndall Avenue, Bristol BS8 1TQ, UK; 2 Genome Evolution Laboratory, Department of Biology, The National University of Ireland, Maynooth, Kildare, Ireland; 3 Sorbonne Université, UPMC Univ Paris 06, CNRS, Algal Genetics Group, UMR 8227, Integrative Biology of Marine Models, Station Biologique de Roscoff, CS 90074, F-29688 Roscoff, France; 4 Institute of Human Genetics, UPR 1142, CNRS, 34396 Montpellier Cedex 5, France

## Abstract

There is currently convincing evidence that microRNAs have evolved independently in at least six different eukaryotic lineages: animals, land plants, chlorophyte green algae, demosponges, slime molds and brown algae. MicroRNAs from different lineages are not homologous but some structural features are strongly conserved across the eukaryotic tree allowing the application of stringent criteria to identify novel microRNA loci. A large set of 63 microRNA families was identified in the brown alga *Ectocarpus* based on mapping of RNA-seq data and nine microRNAs were confirmed by northern blotting. The *Ectocarpus* microRNAs are highly diverse at the sequence level with few multi-gene families, and do not tend to occur in clusters but exhibit some highly conserved structural features such as the presence of a uracil at the first residue. No homologues of *Ectocarpus* microRNAs were found in other stramenopile genomes indicating that they emerged late in stramenopile evolution and are perhaps specific to the brown algae. The large number of microRNA loci in *Ectocarpus* is consistent with the developmental complexity of many brown algal species and supports a proposed link between the emergence and expansion of microRNA regulatory systems and the evolution of complex multicellularity.

## INTRODUCTION

MicroRNAs (miRNAs) are small, 20–24 nucleotide RNA molecules (exceptionally up to 26 nucleotides) that regulate gene expression by affecting the translation or the stability of target gene transcripts. These small RNA molecules are generated from the double stranded regions of hairpin-containing transcripts by the action of RNAseIII endonucleases such as Drosha and Dicer and are then incorporated into RNA-induced silencing complexes (RISCs), which use the miRNAs as guides to recognize and bind to specific RNA targets. miRNAs have been shown to play key roles in the regulation of many important processes in both plants and animals (1,2) and it has been suggested that the acquisition of these versatile regulatory molecules may have been a key factor in the evolution of complex multicellularity (3–5).

The lack of sequence similarity between plant and animal miRNA families and marked differences between the pathways that generate miRNAs in the two groups suggest that these molecules evolved independently in the two lineages (6–9). In contrast, key components of the miRNA system, such as Dicer endonucleases and Argonaute (which is the central component of RISCs), are found in diverse eukaryotic lineages and are thought to be very ancient and perhaps common to all eukaryotes (10,11). These proteins are thought to have evolved originally as components of systems involving other classes of small RNA, such as the small interfering RNAs (siRNAs), and only later to have been recruited as components of miRNA pathways (9). Like miRNAs, siRNAs are small RNA molecules generated by endonuclease digestion but they may be derived from diverse sources of double stranded RNA such as viral genomes, long transcribed inverted repeats or the products of convergent transcription. miRNAs on the other hand, are derived by endonuclease digestion of self-complementary precursor RNAs that form hairpin structures.

Although miRNAs were originally identified in land plants and animals, it has become increasingly clear in recent years that these are not the only eukaryotic lineages to have evolved regulatory systems based on these small RNA molecules. Within the animal lineage, the miRNAs of demosponges are unrelated to those of other animal groups and may have evolved independently (12). Similarly, the unicellular green alga *Chlamydomonas reinhardtii* also appears to possess an miRNA system that is unrelated to that of the land plant lineage (13,14), and no miRNA gene has been shown to be shared between land plants and green algae (15). There is also convincing evidence for the presence of miRNA systems in the brown alga *Ectocarpus* (16) and the social amoeba *Dictyostelium* (17,18). In addition, microRNA-like molecules have been reported in the fungus *Neurospora crassa* (19). Together these reports suggest that at least six or seven different eukaryotic groups possess miRNA systems. Moreover, these miRNA systems appear to have evolved independently in each group because no miRNAs are shared across groups and many intermediate lineages do not possess miRNAs (20). For example, miRNAs have been reported in the brown algae but no strong candidate miRNA loci have been identified in the genomes of three diatoms, which represent another lineage within the stramenopiles (21,22). A common evolutionary origin for the miRNA systems of diverse eukaryotic lineages therefore seems highly unlikely, as it would have required widespread loss of miRNA systems from intermediate lineages and extensive sequence divergence of shared miRNA loci. It is possible, however, that additional eukaryotic groups possess miRNA systems that have not yet been characterized. Indeed, putative miRNAs have been described in several additional lineages, although closer examination of the reported molecules has often failed to support their classification as miRNAs (20). Given the key roles of miRNAs as regulatory molecules in a broad range of processes and their implication in major evolutionary transitions such as the emergence of complex multicellularity (3–5), it is important both to experimentally confirm and characterize miRNA regulatory systems in groups where these systems exist and to clearly confirm their absence from other lineages. Here, we used deep sequencing of small RNA molecules, together with northern blot analysis, to identify and characterize miRNA loci in the filamentous model brown alga *Ectocarpus* and applied a set of stringent criteria to distinguish strong candidate miRNAs from other genomic sources of small RNAs such as siRNA loci. This analysis demonstrated that a recently described set of candidate miRNAs (23) are highly unlikely to correspond to miRNA loci and are more likely siRNAs, but also identified a large repertoire of 63 miRNA families in the *Ectocarpus* genome, the large majority of which had not been described previously. The complexity of the miRNA system in *Ectocarpus* is discussed in the light of the emergence of complex multicellularity in the brown algal lineage. We also discuss the importance of applying stringent criteria to validate candidate miRNA loci in the context of understanding miRNA emergence and evolution across the eukaryotic tree.

## MATERIALS AND METHODS

### *Ectocarpus* strains and culture

Two near-isogenic, male and female inbred lines Ec602 and Ec603 were derived from the male strain Ec137 and the female strain Ec25 by repeatedly crossing male and female sibling progeny for six generations (see Ahmed et al. for a detailed pedigree (24)). Ec137 (which carries the *immediate upright* mutation) (25), and Ec25 are siblings of the genome sequenced strain Ec32 (16). Two replicates of gametophytes for each sex were cultivated under standard conditions (26) and frozen at maturity (4 weeks old). Males bore many plurilocular gametangia, females were larger with fewer plurilocular gametangia. All material was examined under binocular and light microscopes to verify the presence of plurilocular gametangia and pools of about 400 individuals from these synchronous cultures were frozen in liquid nitrogen for each replicate.

### Small RNA sequencing

The generation of 3 203 265 and 3 911 417 small RNA sequence reads for the sporophyte and gametophyte generations of *Ectocarpus*, respectively, has been described previously (16). An additional 77 702 501 small RNA reads (46 161 660 male and 31 540 841 female) were generated for the duplicate, near-isogenic male and female gametophyte samples (Supplementary Table S1). For the latter, small RNAs were isolated and prepared for sequencing by Fasteris (Plan-les-Ouates, Switzerland). Between four and 12 μg of total RNA was extracted for each replicate using the Qiagen Mini kit. RNA was separated on a polyacrylamide gel and the 15–30 nucleotide fraction isolated by excision. Addition of single-stranded adapters and PCR amplification was carried out using the DGE-Small RNA kit (Illumina, San Diego, USA) and small RNAs were sequenced on a HiSeq 2000 (Illumina). The sRNA sequence data can be accessed in the SRA Knowledge Base with the accession number SRP052304.

Adaptor sequence was removed from the raw sequence reads in Galaxy (27) and sequences of <18 or >26 nucleotides or which contained one or more unknown nucleotides were discarded.

### Mapping of sRNA sequence reads to the *Ectocarpus* genome and transcriptome

The filtered reads were mapped against the *Ectocarpus* genome using Bowtie2 (28) with default parameters. Only fully mapped reads were retained (–end-to-end option in Bowtie2). Read coverage for genomic feature (exons, introns, rRNA, tRNA, snoRNA and intergenic regions) was obtained using Samtools (29). *Ectocarpus* snoRNA loci were predicted using ACAseeker and CDseeker. Coordinates of other genomic features, including rRNA and tRNA loci, were obtained from the *Ectocarpus* genome database at Orcae (http://bioinformatics.psb.ugent.be/orcae/overview/Ectsi) (30).

Sliding window analysis of sRNA read coverage was calculated using a custom script and a non-overlapping sliding window of 25 kb. The data is presented as sRNA read counts per window. Visual analysis of the mapping pattern of the sRNA reads onto the genome indicated that it was not consistent with more than a very limited level of contamination by degraded mRNA fragments. This conclusion was also supported by the fact that 47% of the reads that mapped to mRNA-encoding regions of the genome mapped to the antisense strand compared to the mRNA transcript (data not shown).

Expression levels (transcript abundance) of protein-coding genes in male and female gametophytes were determined using the Illumina RNA-seq dataset described by Ahmed *et al*. (24).

### Identification of *Ectocarpus* protein-coding genes with potential roles in small RNA pathways

*Ectocarpus* homologues of plant, animal and fungal protein-coding genes that have been implicated in various aspects of sRNA biogenesis and function were identified by screening for species to species best reciprocal Blastp matches.

### Identification and characterization of miRNA loci in *Ectocarpus*

*Ectocarpus* is distantly related to both land plants and animals. Screens for miRNAs therefore employed both miRDeep2 (31), which implemented criteria for the identification of animal miRNAs, and miRDeep-p (32), a modified version of miRDeep that was adapted for the identification of plant miRNAs by allowing extended precursor sequences. After filtering, the reads from all six samples (Supplementary Table S1) were combined into a single dataset and provided as input for each program. Candidate miRNA precursors were then extracted from the output files and the miRDeep-2 and miRDeep-p outputs compared using Blast to identify and remove redundant candidate miRNA precursors that had been identified by both programs.

Custom scripts, which incorporated Bowtie (28), were used to align all the sRNA sequence reads to the candidate precursor miRNA loci, with no mismatches allowed. For each miRNA locus, the sRNA species with the highest read count was compared with miRBase using Blast and the most similar match recovered if matches were detected. The entire precursor sequence was folded with Vienna RNAfold (33) and a further script was implemented to combine the output of this analysis with the sRNA read mapping results and miRBase Blast search results.

Similar analyses of sRNA read mapping were also carried out for 23 *Ectocarpus* miRNA loci recently reported by Billoud *et al*. (23).

### Investigation of the genomic origin of the *Ectocarpus* miRNA loci

To identify miRNA families, ungapped alignments of either the mature miRNA sequences or just the seed regions (nucleotides 2–8) were generated with Muscle (34) and pairwise sequence identity calculated using MEGA (35). Pre-miRNA sequences were analysed with RepeatMasker (http://repeatmasker.org) against Repbase to detect sequence relationships with repeated elements. Similarity with other genomic regions was detected using Blastn and the pre-miRNA sequences as a query against the *Ectocarpus* genome sequence (http://bioinformatics.psb.ugent.be/orcae/overview/Ectsi) (30). The principal aim of the latter analysis was to determine whether the *Ectocarpus* miRNA loci might have been derived from duplicated copies of protein-coding genes.

### Searches for homologues of *Ectocarpus* miRNA loci in other stramenopile genomes

Searches were carried out for homologues of *Ectocarpus* miRNA loci in the genomes of four stramenopile species: *Thalassiosira pseudonana* (diatom; Thaps3 assembled and unmapped scaffolds, http://genome.jgi-psf.org/Thaps3/Thaps3.download.ftp.html) (36), *Phaeodactylum tricornutum* (diatom; Phatr2 assembled and unmapped scaffolds, http://genome.jgi-psf.org/Phatr2/Phatr2.download.ftp.html) (37), *Aureococcus anophagefferens* (Pelagophyceae; http://genome.jgi-psf.org/Auran1/Auran1.download.ftp.html) (38) and *Nannochloropsis oceanica* (Eustigmatophyceae; https://bmb.natsci.msu.edu/BMB/assets/File/benning/genome_assembly.txt) (39). Two different strategies were used. The first involved comparing the entire pre-miRNA sequences with the genomes using Blastn and then analysing the results manually for extended regions of similarity that preferentially included the miRNA and miRNA* regions of the pre-miRNA. The second method involved querying both the miRNA and miRNA* sequences against the genomes and retaining matches with less than four mismatches. The region surrounding each match was then recovered from the subject genome sequence and tested for the ability to form a hairpin loop with sufficient complementary base-pairing between the candidate miRNA and miRNA* sequences.

### Comparisons with miRNA loci from other eukaryotic lineages

Structural features of the *Ectocarpus* miRNAs were compared with those of miRNAs from species belonging to other eukaryotic lineages. The sets of miRNAs from the other eukaryotic lineages had been validated previously (40,41) using the same four criteria that we employed in this study to select valid *Ectocarpus* miRNAs (see the Results and Discussion section for details of the four criteria). The species used for the comparisons were *Drosphila melanogaster, Danio rerio* (animals), *Amphimedon queenslandica* (demosponges), *Dictyostelium discoideum* (slime molds), *Arabidopsis thaliana, Physcomitrella patens* (land plants), *Chlamydomonas reinhardtii* (chlorophyte green algae). The miRNA expression data were recovered from miRBase (8). Foldback lengths (42) were calculated for the miRNAs from each species using precursor sequences deposited in miRBase v21 that had both the 5′ and 3′ products annotated and have been previously validated as genuine (40,41), and from the annotated *Ectocarpus* miRNAs described herein. The region corresponding to each miRNA precursor was identified in the respective genome sequence using Blast and the region, together with 100 nucleotides of flanking sequence in both directions, was recovered. RNAfold (33) was used to predict secondary structure, and the foldback was deemed to have ended either at the first occurrence of three consecutive unbound nucleotides or at the occurrence of another secondary structure.

### Northern blot analysis

Samples of either 50 or 63 μg of total RNA from male or female *Ectocarpus* strains were subjected to northern blot analysis as previously described (43). DNA oligonucleotide probes complementary to the miRNAs of interest were radioactively labelled at the 5′-end using T4 polynucleotide kinase.

### Searches for potential target genes of *Ectocarpus* miRNA loci

Potential target genes of *Ectocarpus* miRNAs were identified using the web version of TAPIR (http://bioinformatics.psb.ugent.be/webtools/tapir/) in precise mode with the default options.

## RESULTS AND DISCUSSION

### Sequence analysis of gametophyte small RNAs

The first description of miRNA loci in the brown alga *Ectocarpus* was based on the analysis of about seven million sRNA sequences generated using both sporophyte and gametophyte tissue (16). For the present study, an additional 78 million sRNA sequence reads were generated using replicate samples of RNA from male and female gametophytes. Mapping of the sRNA sequence reads to the genome indicated that they were derived from all chromosomes, with no obvious bias towards particular linkage groups or regions within linkage groups (Supplementary Figure S1). After exclusion of reads corresponding to ribosomal RNA (rRNA), transfer RNAs (tRNAs) and small nucleolar RNAs (snoRNAs), the highest coverage of mapped sRNA reads per base pair was for transposable elements (Table 1). This confirms an earlier observation (16) and suggests a possible role for these sRNAs in maintaining genome stability by repressing transposition. Small RNAs have been associated with silencing of transposable elements in a broad range of eukaryotic organisms, including animals, plants and fungi (44). Thirty seven percent of the mapped reads corresponded to regions annotated as genes, with the exon regions being covered slightly more densely than the introns (1.5-fold).

**Table 1.** Mapping of sRNA reads to different fractions of the *Ectocarpus* genome

Genome fraction or feature	sRNA read count	Cumulative size (bp)	Read coverage (reads per bp)
Exons	3 469 027	25 662 441	0.14
Introns	10 424 142	81 093 270	0.13
Intergenic	7 050 763	73 482 052	0.10
Transposons	8 915 590	10 605 262	0.84
tRNA	755 534	21 829	34.61
rRNA	7 092 058	7903	897.39
snoRNA	57 845	88 311	0.66

One unusual structural feature of the *Ectocarpus* genome is that the coding strands of adjacent protein-coding genes exhibit a strong tendency to alternate between the two strands of the DNA as one scans along the chromosome, a feature that is normally associated with very small eukaryotic genomes (16). One consequence of this is that 9508 of the 16 192 genes in the *Ectocarpus* genome are part of a convergently transcribed gene pair, i.e. the two genes are located adjacent to one another on the chromosome and transcribed convergently. Pairs of convergent transcription units have been reported to be an important source of sRNAs in both animals and land plants (45–47). This is thought to be because overlap between the pairs of transcripts generates regions of double-stranded RNA. In *Ectocarpus*, the number of sRNA reads that mapped to genes that were members of convergent gene pairs (median FPKM 0.20) was slightly, but significantly (Kruskal–Wallis test, *P*-value < 8.1e−09), greater than for the other genes in the genome (median FPKM 0.18). However, analysis of mRNA-seq expression data showed that convergent genes were also expressed at a slightly higher level than non-convergent genes (mRNA median FPKM of 10.1 compared with 8.8, Kruskal–Wallis test, *P* = 6.8e−16) and when the number of sRNA reads per gene was normalized for this difference there was no significant difference between genes that were members of convergent pairs and the other genes in the genome (Kruskal–Wallis test, *P* = 0.77). This indicates that convergent gene pairs are not a preferential source of sRNAs in *Ectocarpus*.

An analysis was also carried out to identify protein-coding genes with potential roles in small RNA pathways in *Ectocarpus*. Reciprocal best Blast analysis identified >30 homologues of plant, animal and fungal genes that have been implicated in various aspects of sRNA biogenesis and function (Table 2).

**Table 2.** *Ectocarpus* homologues of proteins involved in microRNA function or related small RNA pathways in other species

Query species	Query gene	Function	Accession number	*Ectocarpus* best Blastp E-value	Reciprocal best blast (species to species)	*Ectocarpus* homologue
Ath	DCL2	Dicer	NP_566199.4	1E−12	Yes	Esi0039_0031
Aga	Ago1	Argonaute	EAA00062.4	3E−94	Yes	Esi0203_0032
Ddi	AgnA	Argonaute (piwi)	EAL69296.1	2E−29	No	No homologue
Ath	RDR1	RNA-dependent RNA polymerase	NP_172932	4E−30	Yes	Esi0512_0001
Ath	RDR6	RNA-dependent RNA polymerase	NP_190519	4E−88	Yes	Esi0100_0017
Ath	SDE3	SDE3/MOV10/Armitage	AAK40099.1	3E−62	Yes	Esi0216_0047
Ath	DAWDLE	pri-miRNA generation	NP_188691.1	3E−47	Yes	Esi0132_0041
Ath	SQUINT	pre-miRNA processing	Q9C566	1E−54	No	Multiple cyclophilins
Ath	HSP90	pre-miRNA processing	AED96244.1	0	Yes	Esi0138_0009
Ath	HASTY	Nuclear export (exportin5/MSN5/HASTY)	Q84UC4	0.00002	Yes	Esi0059_0032
Ath	SERRATE	RNA binding protein that may maintain hairpin structure or direct Dicer	Q9ZVD0	8E−10	Yes	Esi0289_0007
Ath	HYL1	RNA binding protein that may maintain hairpin structure or direct Dicer	NP_563850.1	No hit	n/a	No homologue
Ath	TOUGH	RNA binding protein that may maintain hairpin structure or direct Dicer	AAR99647.1	1E−23	Yes	Esi0125_0056
Ath	HEN1	2′-O-Methylation of miRNAs	NP_567616.1	No hit	n/a	No homologue
Ath	SUO	miRNA-mediated translational repression	NP_190388.2	No hit	n/a	No homologue
Ath	MOS2	miRNA processing	NP_174617.1	2E−23	Yes	Esi0084_0044
Ath	PRL5	miRNA processing	NP_193325.1	6E−131	Yes	Esi0025_0074
Ath	CDC5	miRNA processing	NP_172448.1	3E−69	Yes	Esi1122_0001
Ath	SICKLE	miRNA biogenesis	NP_567704.1	No hit	n/a	No homologue
Ath	KTF1/RDM3/SPT5-like	AGO4 interactor	NP_196049.1	2E−16	No	No homologue
Ath	CBP20	Cap binding complex	NP_199233.1	5E−39	Yes	Esi0206_0003
Ath	CBP80	Cap binding complex	NP_565356.1	6E−28	Yes	Esi0155_0015
Ath	DECAPPING1	Decapping complex	NP_563814.1	4E−16	Yes	Esi0489_0024
Ath	DECAPPING2	Decapping complex	Q8GW31	3E−40	Yes	Esi0010_0022
Ath	VARICOSE	Decapping complex	AEE75331.1	3E−19	Yes	Esi0205_0050
Ath	3-HYDROXY-3-METHYLGLUTARYL CoA REDUCTASE	Isoprenoid synthesis protein that affects miRNA action	NP_177775.2	3E−83	Yes	Esi0027_0087
Ath	HYDRA1	Isoprenoid synthesis protein that affects miRNA action	NP_173433.1	0.00000001	No	No homologue
Ath	SMALL RNA DEGRADING NUCLEASE 1	miRNA degradation	AEE78626.1	1E−25	Yes	Esi0118_0050
Ath	HESO	miRNA uridylation	NP_181504.2	3E−14	No	Uridyltransferases eg. Esi0771_0003
Ath	AMP1	Inhibition of protein production	NP_567007.1	1E−87	Yes	Esi0122_0005
Ath	KATANIN	Cytoskeleton genes that affect miRNA action	NP_178151.1	4E−93	Yes	Esi0007_0029
Ath	dsRNA BINDING PROTEIN4	tasiRNA biogenesis	Q8H1D4	No hit	n/a	No homologue
Ath	SGS3	RNA-directed DNA methylation	AAF73960.1	No hit	n/a	No homologue
Ath	C-TERMINAL DOMAIN PHOSPHATASE-LIKE1	Phosphorylation role in dsRNA gene regulation	NP_193898.3	0.023	No	No homologue
Ath	CLSY1	Generation of 24nt rasiRNAs	NP_189853.1	3E−17	No	No homologue
Ath	PolIV	siRNA synthesis	NP_176490.2	7E−42	No	No homologue
Ath	NRPE1	siRNA synthesis	NP_181532.2	9E−43	No	No homologue
Dme	Pasha	Drosha complex	AAF57175.1	No hit	n/a	No homologue
Hsa	EWSR1	Drosha complex	NP_053733.1	1E−16	Yes	Esi0222_0008
Hsa	p68/DDX5	Drosha complex	NP_004387.1	3E−87	Yes	Esi0013_0199
Hsa	p72/DDX17	Drosha complex	NP_001091974.1	5E−157	Yes	Esi0007_0206
Hsa	Fus	Drosha complex	AAC35285.1	2E−12	No	No homologue
Hsa	ADAR	pri-and/or pre-miRNA editing	EAW53187.1	0.000003	No	No homologue
Hsa	TRBP	pre-miRNA processing	Q15633.3	No hit	n/a	No homologue
Hsa	PACT	pre-miRNA processing	AAL68925.1	1E−26	No	No homologue
Dme	loquacious	pre-miRNA processing	AAY40789.1	No hit	n/a	No homologue
Hsa	KSRP	Promoter of miRNA biogenesis	AAB53222.1	2E−11	No	No homologue
Hsa	Lin28	Drosha/Dicer inhibitor	AAH28566.1	No hit	n/a	No homologue
Hsa	TNRC6 (GW182)	RISC component	NP_055309.2	0.84	No	No homologue
Hsa	TNRC6A	Ago interactor	Q8NDV7	No hit	n/a	No homologue
Hsa	TNRC6B/KIAA1093	Ago interactor	TNC6B_HUMAN	No hit	n/a	No homologue
Hsa	TNRC6C	Ago interactor	Q9HCJ0	No hit	n/a	No homologue
Hsa	TRIM65	Ubiquitination of TNRC6	NP_775818.2	0.000001	No	No homologue
Dme	R2D2	Double-stranded RNA binding protein	Q9VLW8	No hit	n/a	No homologue
Dme	FMR1	miRNA biogenesis	Q9NFU0	No hit	n/a	No homologue
Dme	BEL	ATP-dependent RNA helicase	Q9VHP0	1E−134	Yes	Esi0186_0022
Dme	RM62	DEAD-box RNA helicase	P19109	1E−140	No	Multiple RNA helicases
Cel	ERI1	RNA exonuclease	O44406	6E−26	Yes	Esi0039_0083
Cel	RDE-4	siRNA production	Q22617	No hit	n/a	No homologue
Cel	SID1	Systemic RNA interference	Q9GZC8	No hit	n/a	No homologue
Spo	Hrr1	RNA-directed RNA polymerase complex	O74465	2E−41	No	No homologue
Spo	Cid12	Poly(A) polymerase	O74518	0.00000002	No	Esi0053_0139 (Poly(A) polymerase)
Spo	Chp1	RNAi pathway	Q10103	No hit	n/a	No homologue
Spo	Tas3	RNAi pathway	O94687	No hit	n/a	No homologue

For Dicer, Argonaute and RNA-dependent RNA polymerase, searches were carried out with multiple sequences from diverse eukaryote lineages (10). Ath, *Arabidopsis thaliana*; Aga, *Anopheles gambiae*, Ddi, *Dictyostelium discoideum*; Dme, *Drosophila melanogaster*; Hsa, *Homo sapiens*; Cel, *Caenorhabditis elegans*; Spo, *Schizosaccharomyces pombe*; n/a, not applicable.

### *Ectocarpus* has a large and diverse repertoire of microRNAs

A screen was carried out for miRNA loci using the algorithms miRDeep2 and miRDeep-p, which are optimized to detect animal-like and plant-like miRNAs respectively, together with custom scripts. This analysis identified 1882 candidate miRNA loci, which were then manually filtered following established criteria based on highly conserved features common to both animal and plant miRNA loci (20,40,41): (i) at least 15 nucleotides of the miRNA must pair with the opposite arm of the hairpin, (ii) there should be evidence for the expression of both the miRNA and the miRNA*, (iii) the 3p product should extend two nucleotides beyond the 5p product at its 3′ end (with a corresponding extension at the 3′ end of the 5p product), (iv) 5′ cleavage of the miRNA must be precise, with the clear majority of the reads (at least 66%) starting at the same nucleotide.

**Figure 1.**
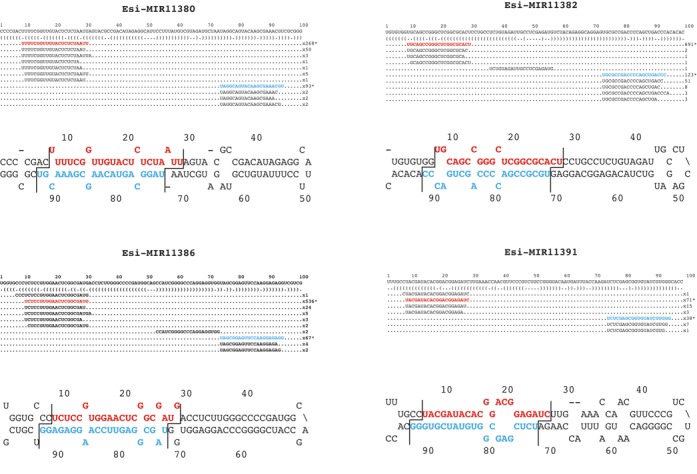
Representative *Ectocarpus* miRNA loci. Representation of read data mapping and the positions of the miRNA (red) and miRNA* (blue) on the predicted hairpin for four representative *Ectocarpus* miRNA loci. Note the high degree of homogeneity of 5′ ends. Lines cutting across the hairpins indicate the two nucleotide offset typical of Dicer processing. Similar diagrams for the full set of 64 miRNAs are shown in Supplementary Figure S2.

**Figure 2.**
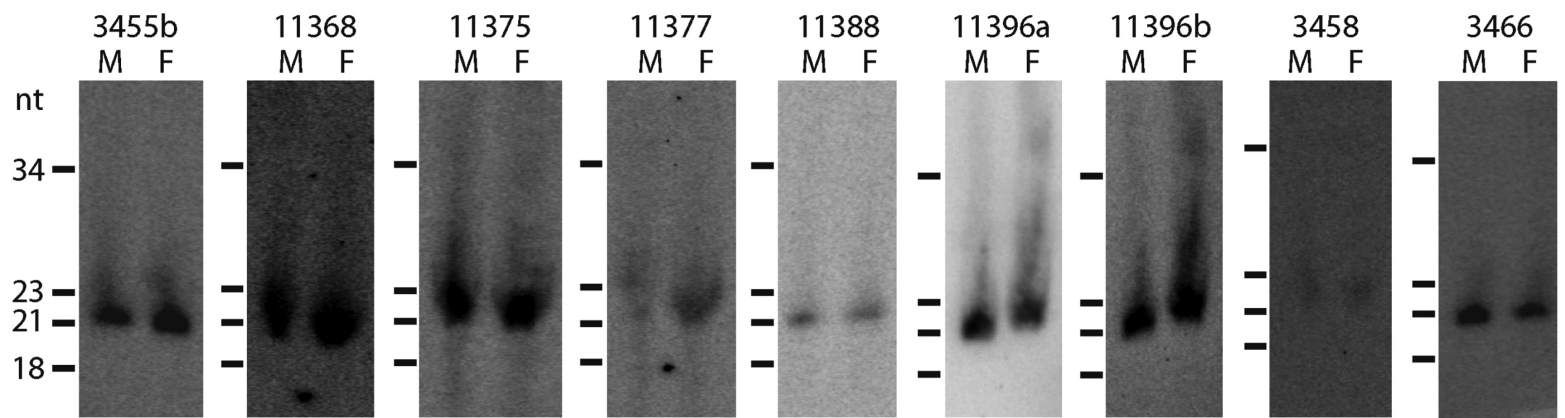
Northern blot analysis of miRNA expression in male and female gametophytes. Hybridization to 50 (esi-MIR3455b, esi-MIR11368, esi-MIR11375, esi-MIR11377, esi-MIR11388, Esi-miR3458, Esi-miR3466) or 62.3 (esi-MIR11396a, esi-MIR11396b) μg of male or female total RNA per lane. Exposure times were the same for all samples except for esi-MIR11396a, which is highly abundant and was exposed for one day rather than 4 days.

The final set of 63 microRNA families (representing 64 loci) included six of the miRNA families previously described by Cock *et al*. (16), together with 57 newly identified families (Supplementary Table S2, Figure 1 and Supplementary Figure S2). Northern blot analysis was carried out to independently validate a subset of nine of these miRNA loci using RNA from a separate set of RNA samples. sRNA species of the expected size were detected in both male and female gametophyte RNA samples for all of the nine miRNA loci (Figure 2). The relative abundances of the miRNAs, estimated from the northern blot analysis, corresponded approximately with estimations based on RNA-seq, with some miRNAs, such as esi-MIR11396a and esi-MIR11368, being expressed at high to very high levels and others, such as esi-MIR11377 and esiMiR3458, being less abundant.

A striking feature of *Ectocarpus* miRNAs is their remarkable diversity, with almost every miRNA constituting a distinct miRNA gene family. When the seed regions of the miRNAs (nucleotides 2–8) (48) were compared, only one pair of genes (esi-MIR11384a/esi-MIR11384b) was classed as belonging to the same family. The same result was obtained when a criterion of at least 85% identity between entire, mature miRNAs (15) was used to define members of a gene family. Even when this latter criterion was considerably relaxed to at least 75% identity, only three families, each with two members, were identified. These observations suggest that miRNA gene duplication has not played an important role in the generation of new miRNA loci in the brown algal lineage. This is in stark contrast to the role that both individual gene and whole genome duplications have played in miRNA family expansion in both animals (49,50) and land plants (15). The low number of paralogues within miRNA families in *Ectocarpus* is consistent with both the lack of evidence for any whole genome duplication events in the lineage leading to this organism and the unusually low number of tandem duplications of protein coding genes (823) identified in this species (16).

Mapping of the 64 miRNA loci to the *Ectocarpus* genome indicated that they were distributed randomly across the chromosomes (Supplementary Figure S1). Clusters of miRNAs (defined here as being within 5 kb (15)) are common in both animals and land plants (15,49,51,52). In contrast, the *Ectocarpus* miRNA loci exhibited very little tendency to cluster in the genome, with only two pairs of loci being separated by <5 kb. The miRNAs encoded by one of these pairs of clusters shared 76% identity, suggesting that they may have been derived from a tandem duplication event. However, such local duplication events appear to have been very rare.

There is evidence that some miRNA loci in both animals and plants produce more than one pair of miRNA-like molecules from a single pre-miRNA hairpin structure (53–56). These additional miRNA-like molecules are often in phase with the miRNA/miRNA* pair, in which case they have been called miRNA-offset RNAs (moRNAs). There is accumulating evidence that these additional miRNA-like molecules have biological functions (55,56) and, therefore, they may contribute significantly to the total size of the miRNA repertoire in some species. In plants these miRNA-like molecules tend to exhibit a strong preference for a U or A nucleotide at the 5′ end (90% in *Arabidopsis*) (56) but this does not appear to be the case in animals (53). We did not obtain evidence that this type of miRNA-like molecule occurs commonly in *Ectocarpus*, but esi-MIR11352 was of interest because a putative moRNA (UCUUUGAUCGGACAUGUUUCU) with a 5′ U nucleotide and 5′ processing homogeneity was detected for this locus, along with a potential ‘star’ product (Supplementary Figure S2).

**Figure 3.**
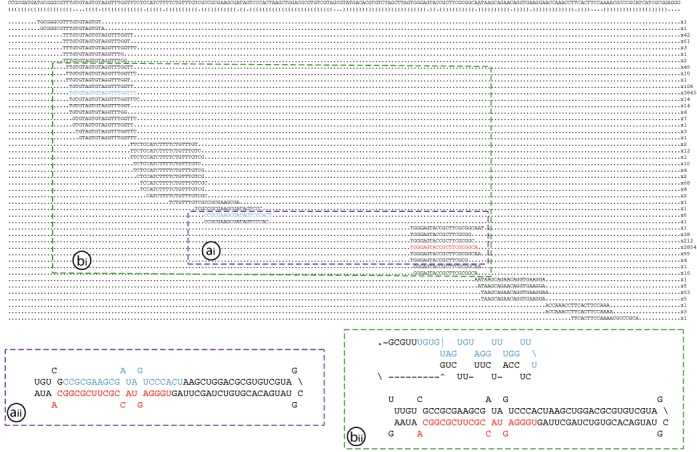
Example of a weak candidate miRNA. Weak candidate miRNA 8 was identified by miRDeep2 (31) with a high score (1.6e+3). The locus encodes potential miRNA (2854 reads) and miRNA* (6 reads) products (ai), with the expected 2 nucleotide offset and a characteristic hairpin loop (aii). However, when the precursor sequence was extended, two regions were identified on the 5′ side that exhibited higher expression (3845 and 68 reads, respectively) than the miRNA* sequence originally annotated by miRDeep2 (bi). When this longer precursor was folded (bii) it no longer formed a characteristic hairpin, and the two products lacked both the required offset and sufficient complementary base pairing.

In addition to the 64 miRNA loci identified, we also noted the presence of a large number of loci that were identified by miRdeep2 and/or miRdeep-p and fulfilled the majority of the criteria we used to define miRNA loci but were located in genomic regions consisting of complex, extensive palindromic sequences that generated multiple sRNA species over a region of several hundred base pairs (see Figure 3 for an example of such a locus). Sixty-five of these additional loci, which we classified as weak miRNA candidates, are shown in Supplementary Figure S3. The analysis of these loci highlighted the importance of manually checking the genomic context of a candidate miRNA even if loci are computationally predicted with high confidence. Further analysis will be required to determine whether these loci actually produce functional miRNAs, but it is possible that they may represent so-called transitional miRNAs (57), i.e. newly emerging miRNA loci.

### Quantitative PCR is not a suitable strategy for identifying novel miRNAs

Detailed analysis of the mapped sRNA reads allowed us to demonstrate that 23 *Ectocarpus* miRNA loci recently described by Billoud *et al*. (23) failed to pass the quality control criteria applied here. These candidate miRNAs were part of a larger set (500–1500 depending on criteria) that had been identified using a bioinformatic approach. A subset of 72 candidates were analysed by Billoud *et al*. using quantitative PCR and 23 were subsequently reported as miRNAs. However, we were unable to validate any of these 23 candidate miRNAs using our sRNA read data. For five of the candidates no sRNA reads mapped to the loci, for an additional thirteen candidate miRNAs the most abundant class of read was not the annotated miRNA product and for the final five candidates the miRNA/miRNA* pairing was clearly incorrect (Supplementary Figure S4). Thus the candidate miRNAs identified by Billoud *et al*. are most likely siRNAs.

Quantitative PCR is commonly used to validate candidate miRNAs identified by bioinformatic approaches due to its low cost. The analysis carried out in this study identified limitations of this approach and demonstrated the importance of validation using sRNA read data. sRNA read data allows key criteria such as evidence for the existence of both miRNA and miRNA* species, homogeneity of 5′ processing and pre-miRNA processing consistent with dicer activity, to be tested. Whilst quantitative PCR is clearly useful for the quantification of known miRNAs, as a tool to validate novel candidate miRNA loci it suffers from the weakness of not being able to distinguish miRNAs from rare RNA species, siRNAs or degraded products of diverse RNA transcripts.

### Expression patterns of *Ectocarpus* miRNAs

Expression levels (Supplementary Table S2) varied between 0.24 and 8387.33 RPM for the miRNA and between 0.01 and 131.95 RPM for the miRNA* (the miRNA being defined as the most strongly expressed of the two species (58)).

Sex-biased expression of miRNA loci has been reported for both animals (59–62) and land plants (63). Statistical tests, implemented with DEseq and EdgeR, were therefore carried out to determine whether any of the miRNA loci were differentially expressed in male and female individuals, but no statistically significant differences were detected. Similarly, there was no evidence that the miRNAs were differentially expressed between the sporophyte and gametophyte generations of the life cycle. Note that the RNA blot analysis did not provide any evidence for differential expression of the miRNA loci between male and female individuals (Figure 2), in agreement with the analysis of the RNA-seq data.

### Prediction of miRNA target genes

Sequence complementarity between miRNAs and their target mRNAs varies across eukaryotic groups, with plant and green algal miRNAs tending to have a high level of complementarity with their target genes and animal miRNA, in contrast, tending to have low complementarity (although there is evidence that plant miRNAs can also have low complementarity targets (64)). As a first step towards identifying putative targets of *Ectocarpus* miRNAs, we carried out a search, using TAPIR (65), based on the assumption that complementarity between the miRNA and mRNA target was high. This analysis identified 160 potential target genes in the *Ectocarpus* genome (Supplementary Table S3), with individual miRNAs being matched to between zero (17 miRNAs) and 13 target genes. Experimental validation will be required to verify that these genes are actually targets of the *Ectocarpus* miRNAs.

Interestingly, seven of the 160 genes were predicted to be targeted by two miRNAs. In four of these seven cases, the two miRNAs had different seed regions and targeted different regions of the gene. Note however that, in general, the high diversity of the seed regions of the *Ectocarpus* miRNAs suggests that there is unlikely to be a high level of target redundancy in this species, i.e. in most cases target genes are unlikely to be targeted by multiple miRNA loci.

Functions could be predicted for 104 of the putative target genes based on sequence information and this analysis indicated that they were involved in a broad range of cellular processes. Strongly represented cellular processes included cellular signalling and regulation (11 genes), proteolysis (11 genes), membrane function (10 genes) and genes with a probable role in defence (10 genes), with an additional 18 genes involved in general protein-protein interactions.

### Genomic origin of the *Ectocarpus* miRNAs

Several mechanisms have been described for the generation of new miRNA loci; these include: (i) duplication of existing miRNA loci (66), (ii) generation of miRNA loci from duplicated copies of protein-coding genes (67,68), (iii) evolution from transposable elements (17,69,70) and (iv) evolution from the many hairpin regions scattered throughout the genome (52,71,72). The near absence of miRNA families and miRNA clusters in *Ectocarpus* suggests that duplication of miRNA loci has not been not a major mechanism for the generation of new miRNA loci in this species. Similarly, comparison of the *Ectocarpus* pre-miRNA sequences with transposon sequences using RepeatMasker and with the *Ectocarpus* protein-coding genes using Blast did not detect any evidence that the miRNAs were derived from the latter features. By deduction, therefore, these analyses suggest that hairpin regions in the genome may have been an important source of new miRNA loci in this lineage. Hairpin regions within introns may have been favoured during this process because they had the advantage of already being transcribed. Evolution of miRNA loci from genomic hairpins is thought to have been an important mechanism of miRNA genesis in animals, and it has been suggested that this mechanism, as opposed to recruitment of duplicated fragments of future target genes, may have been favoured by the low level of sequence similarity between animal miRNAs and their targets (15). It remains to be determined whether this is also the case for *Ectocarpus*. The search carried out in this study identified potential targets that shared high similarity with the *Ectocarpus* miRNAs but further analysis will be required to validate these potential target genes.

The majority of the *Ectocarpus* miRNA loci are located within protein-coding genes (75%). This contrasts with the situation observed in land plants, where most (84%) miRNA loci are located in intergenic regions (15) and is more similar to that of several animals including humans and *Drosophila*, where nearly half of the miRNA genes occur in introns (52,73). One of the factors that may explain the observed distribution of *Ectocarpus* miRNA loci is that protein coding genes, and particularly intron sequence, constitute an exceptionally large proportion of the genome sequence in this species (16). Indeed, the *Ectocarpus* miRNAs that occur within protein-coding genes were found principally located within introns, the only exceptions being three miRNAs that were located in untranslated regions. All but three of the miRNA loci that were located within protein coding genes were transcribed from the same strand as the host gene (Supplementary Table S2), indicating that the intronic miRNAs could be co-transcribed as part of their host gene mRNA. However, no correlation was detected between the abundances of these miRNAs (RPM) and the abundance of their host gene's mRNA (FPKM) in the duplicate male and female gametophyte samples (Spearman's rank correlation coefficient *ρ* = 0.0019, *P*-value = 0.98). Lack of correlation between the abundances of intronic miRNAs and their host gene mRNA transcripts has also been observed in animal systems (74) (but see also (75)). When the abundances of these two molecules are not correlated, this may be either because their relative abundances are significantly influenced by post-transcriptional processes affecting the processing or stability of at least one of the two types of molecule or because the two features are transcribed independently (or a combination of these two phenomena). Several studies have indicated that a significant proportion of human intronic miRNAs possess their own promoters, which could function independently of the host gene promoter (e.g. (76,77)). It is possible that many of the intronic *Ectocarpus* miRNAs are also transcribed independently of the host gene. Note that, while the emergence of new miRNA loci may be favoured in regions of the genome that are already transcribed such as introns, subsequent acquisition of an independent promoter would confer greater flexibility of expression. In this respect it is interesting to note that, in animals, evolutionarily old intronic miRNA loci appear to be more likely to possess their own promoter region than young intronic miRNA loci (76).

None of the intronic miRNAs were mirtrons. The intron that contains miRNA esi-MIR11390 (intron 3 of gene Esi0084_0039) is predicted to form a stem-loop that involves the entire sequence of the intron but the miRNA/miRNA* duplex is not located next to the splice site.

### Evolutionary origins of the *Ectocarpus* miRNA loci

Comparisons of miRNA complements of diverse species within both the land plant and animal lineages has shown that these loci accumulate gradually over evolutionary time, that their sequences are strongly conserved and that they are rarely lost once acquired (40,41,78). Where loss of miRNA loci has occurred, this can often be correlated with genome reduction and phenotypic simplification, for example in lineages that have adopted a parasitic life history (79). It has recently been suggested that miRNA loss is a more common phenomenon than previously reported (80) but this latter study did not adequately take into account the widely appreciated phenomenon of apparent loss of miRNA loci due to the use of low coverage genome and/or small RNA sequence data, which can lead to considerable over-estimation of the rate of miRNA loss during evolution (40,81,82). Current evidence therefore indicates that a certain proportion of miRNA loci are conserved over long periods of evolutionary time. Based on this observation, we carried out a search for homologues of the *Ectocarpus* miRNAs in other stramenopile lineages.

At present, the *Ectocarpus* genome is the only complete genome sequence available for the brown algae, but the genomes of several other members of the stramenopile supergroup have been sequenced. A search was carried out for sequences homologous to the 64 *Ectocarpus* pre-miRNA regions in the genomes of two diatom species, *Thalassiosira pseudonana* (36) and *Phaeodactylum tricornutum* (37) and members of the Pelagophyceae (*Aureococcus anophagefferens*) (38) and the Eustigmatophyceae (*Nannochloropsis oceanica*) (39). The latter two classes are more closely related to the brown algae than the diatoms (83). Blastn search results were analysed for matching regions that exhibited at least partial conservation of the miRNA and/or miRNA* sequences and could potentially encode RNAs with hairpin structures, but no clear matches were found in any of the four species analysed. Recent estimates indicate that these four species of stramenopiles may all have diverged from the brown algal lineage more than 400 Mya (83). It is therefore possible that extensive divergence over this length of evolutionary time may have obscured homologies. However, given that subsets of both animal and land plant miRNA loci have been strongly conserved over similar periods of time (15,40,41,49), this is unlikely to have been the case for all of the miRNA loci. Moreover, recent extensive searches of three diatom genomes failed to find any strong candidate miRNA loci, indicating that this stramenopile group does not possess a miRNA regulatory system (21,22). Taken together, these observations suggest that the *Ectocarpus* miRNA loci have evolved since the brown algal lineage diverged from that of the Eustigmatophyceae.

There is currently convincing evidence for the existence of miRNA loci in six diverse eukaryotic groups: metazoans, demosponges, slime molds, land plants, chlorophyte green algae (*Chlamydomonas*) and brown algae (1,2,12–14,16,17). Despite considerable conservation of miRNAs within lineages, there are no well-supported cases of miRNA loci being shared between lineages, suggesting that miRNA systems have evolved independently in each lineage, presumably from existing systems such as siRNAs. Interestingly, almost all of the organisms that have been shown to possess miRNAs exhibit some form of multicellularity (*Chlamydomonas* being an exception) and, conversely, the eukaryotic groups that exhibit the highest levels of multicellular complexity—animals, land plants and brown algae (3)—all possess miRNA systems. This correlation between complex multicellularity and the presence of regulatory systems based on miRNAs has led several authors to suggest that the latter may have played a key role in the evolution of the former (4,5). This suggestion is supported by the fact that, in animals at least, developmental complexity (estimated either based on numbers of different cell types or by scoring morphological characters) is approximately correlated with the complexity of the miRNA component of the genome (50,84,85). A similar correlation can be made across eukaryotic groups. We show here that the three eukaryotic lineages that exhibit the highest levels of developmental complexity— animals, land plants and brown algae—also have considerably more complex miRNA repertoires (at least 60 miRNA loci) than less developmentally complex organisms. For example, *Drosophila, Arabidopsis* and *Ectocarpus* possess 110, 64 and 63 miRNA loci, respectively ((40,41) and this study). In contrast, organisms from lineages with a lower level of developmentally complexity, such as *Amphimedon* (eight miRNAs), *Dictyostelium* (11 miRNAs) and *Chlamydomonas* (10 miRNAs), have markedly fewer miRNA loci (40,41).

### Comparison of miRNA structural features across eukaryotic lineages

**Figure 4.**
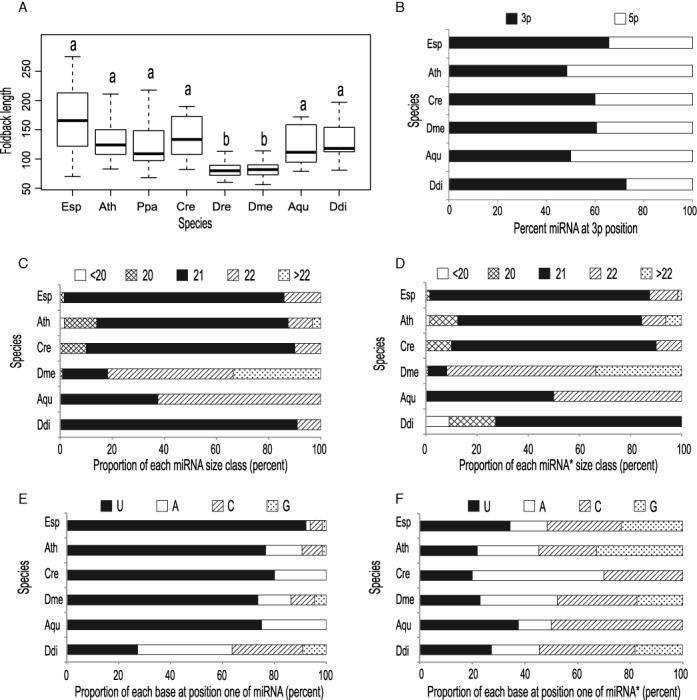
Structural characteristics of miRNA loci from different eukaryotic lineages. (**A**) Variation in foldback length, a and b indicate statistically different size ranges (Kruskal–Wallis test, padj = 1.2e−10), (**B**) position of the miRNA (3p or 5p) on the hairpin, (**C**, **D**) miRNA and miRNA* size distributions, (**E**, **F**) proportions of U, A, C and G at the first residue in miRNAs and miRNA*s from different lineages. The ranges of miRNA size (Kruskal-Wallis test, padj = 2.2e−16), miRNA* size (Kruskal-Wallis test, padj = 2.2e−16) and preference for a uracil residue at position one of the miRNA (Fisher exact test, *P* = 0.0002) were significantly different across species. Aqu, *Amphimedon queenslandica* (number of miRNAs = 8); Ath, *Arabidopsis thaliana* (*n* = 69); Cre, *Chlamydomonas reinhardtii* (*n* = 10); Dre*, Danio rerio* (*n* = 166); Ddi, *Dictyostelium discoideum* (*n* = 11); Dme, *Drosophila melanogaster* (*n* = 110); Esp, *Ectocarpus* sp. (*n* = 64); Ppa, *Physcomitrella patens* (*n* = 40).

If the miRNA systems of diverse eukaryotic lineages evolved independently from a common, ancestral small-RNA-based regulatory system (Table 2) then we would expect the different, extant miRNA systems to exhibit marked differences due to their independent evolutionary histories. To explore this prediction, structural features of the *Ectocarpus* miRNA loci were compared with those of miRNA loci identified in other lineages. On average, the *Ectocarpus* miRNA foldbacks were longer than those of any of the other eukaryotic lineages (170 nt) but were more similar to the long foldbacks of land plant (e.g. *Arabidopsis*, 136 nt), green algal (*Chlamydomonas*, 140 nt) and slime mold (*Dictyostelium*, 132 nt) miRNA loci than to the markedly shorter foldbacks (∼82 nt) of eumetazoan miRNA loci (Figure 4). Note that the foldbacks of the *Amphimedon* miRNA loci were significantly longer than those of *Drosophila* or zebrafish, supporting an independent origin for the miRNAs in this lineage.

The majority of the *Ectocarpus* miRNAs were 21 nucleotides in length (84.3%), the remaining ten loci producing miRNAs of 20 (one locus) or 22 nucleotides (Figure 4). Land plants, *Chlamydomonas* and *Dictyostelium* show a similar preference for 21 nucleotide miRNAs, whereas animal and demosponge miRNAs do not show this bias. As expected, the size ranges of miRNA*s from different species followed a similar pattern to that of the miRNAs (Figure 4).

The *Ectocarpus* miRNAs also showed an exceptionally strong tendency to have a U residue at the first position (92%) whereas this was considerably less marked for the miRNA* sequences (36%). This bias was observed for all miRNAs independent of whether they corresponded to the 5p or the 3p product. The preference for U at the first position was variable across the other eukaryotic lineages (Figure 4). A strong bias was also observed for *Chlamydomonas* (80%), land plant (e.g. 74% for *Arabidopsis*), demosponge (75%) and animal (e.g. 73% for *Drosophila* and around 40% for animals in general (86)) miRNAs, whereas no bias (22%) was observed for *Dictyostelium*. None of these organisms showed a bias for a particular residue at the first position of the miRNA* (Figure 4). Note, however, that the lack of a strong bias does not necessarily mean that the miRNA* species are not selected as guide strands because different argonaute proteins may have different sequence preferences (87).

Analysis of the crystal structure of human Ago2 protein bound to miRNA has indicated that a short loop within the middle (MID) domain, called the nucleotide specificity loop, is likely to play a key role in determining preference for specific 5′ miRNA nucleotides (preference for U and A over G and C). The *Ectocarpus* genome encodes one Argonaute homologue (Esi0203_0032, Table 2), which is 39.8% identical (66.2% similar) to human Ago2. Residues involved in non-specific binding of the 5′ miRNA nucleotide, such as Ago2 Y529, Q545 and K570 are conserved in the *Ectocarpus* protein but the region corresponding to the nucleotide specificity loop is highly divergent. Structural analysis of AGO/miRNA complexes will therefore be required to determine whether steric constraints imposed by the AGO protein underlie the bias towards 5′ U residues in brown algal miRNAs.

In *Ectocarpus*, there was a weak preference for the miRNA to be located in the 3p rather than the 5p position (66%). This was also the case for *Dictyostelium* (73%) *Drosophila* (61%) and *Chlamydomonas* (60%), whereas miRNAs tended to be evenly distributed between the two positions in *Arabidopsis* (48%) and *Amphimedon* (50%).

When these various structural features are taken together, the miRNA repertoires of each eukaryotic lineage exhibit different ranges of characteristics, a pattern that is consistent with each miRNA system having an independent evolutionary origin. The *Ectocarpus* miRNA loci are more similar to land plant miRNAs in terms of their structure but resemble animal miRNA in other respects, such as their strong tendency to be located within genes for example. We also noted that the structures of animal miRNA loci are quite distinct from those of miRNA loci from all the other eukaryotic groups, in particular foldbacks are significantly shorter. This unusual structure feature of animal miRNAs may reflect a molecular constraint specific to that lineage, such as the involvement of a dual RNAseIII Drosha/Dicer system in miRNA processing for example.

miRNA loci from different eukaryotic lineages also exhibited differences in terms of their expression. For example, on average, the miRNA product of an *Ectocarpus* miRNA locus was 446 times more abundant than the miRNA* product, allowing the two products to be clearly distinguished. Similar marked preferences for the miRNA product were observed for *Chlamydomonas* and *Arabidopsis* miRNA loci (425x and 225x, respectively) but the situation was different in *Dictyostelium* and in *Drosophila*, where mean miRNA/miRNA* abundance ratios were only 18× and 83×, respectively. The low ratio observed for *Drosophila* is consistent with the observation that both miRNA and miRNA* species have been shown to be involved in gene regulation in this species (88).

Interestingly, the 65 weak candidate *Ectocarpus* miRNAs shared a number of structural characteristics with the 64 genuine miRNAs, including a tendency to be located within protein-coding genes (67%), a strong bias towards having a U residue at the first position of the miRNA (95% for the miRNA but only 30% for the miRNA*) and a strong bias towards miRNAs that are 21 nucleotides in length (92%). These observations support the hypothesis that the weak candidate loci may represent evolving or nascent miRNA loci (7,57,89,90).

## CONCLUSIONS

Analysis of sRNA read mapping and application of a set of strict criteria allowed us to demonstrate that a previously identified set of 23 *Ectocarpus* loci that had been thought to be sources of miRNAs are more probably siRNA sources. However, the same analysis also allowed the identification of a large number of previously undescribed miRNA loci bringing the total number of well-supported miRNA loci in *Ectocarpus* to 64. The identification of these new loci considerably expands the size of the miRNA complement in this organism and provides additional support for the presence of *bone fide* miRNAs in the brown algae. The 64 *Ectocarpus* miRNA loci were classified into 63 families indicating an exceptionally high level of sequence diversity compared with miRNA repertoires from other eukaryotic lineages. The *Ectocarpus* miRNA loci exhibited a number of other exceptional features including the long lengths of their foldback loops, a very strong preference for a uracil at the start of the miRNA and a very marked difference between the abundances of the miRNA and the miRNA* species.

*Ectocarpus* miRNA loci share features with both animal and plant miRNAs but are not homologous to the miRNAs in these other lineages, consistent with the hypothesis that miRNAs have evolved independently in each of these three lineages. This hypothesis is further supported by the absence of homologues of *Ectocarpus* miRNAs in other stramenopile genomes, suggesting that the brown algal miRNA repertoire evolved after the diversification of this eukaryotic supergroup. Given the developmental complexity of some brown algal species, the discovery of this large repertoire of miRNA loci in *Ectocarpus* also reinforces the proposed link between the acquisition of miRNAs and the emergence of complex multicellularity (3–5). It is particularly striking that the three eukaryotic lineages that exhibit the highest levels of multicellularity complexity appear to possess significantly more miRNAs than species from lineages that exhibit less developmental complexity.

An important aim for the future will be to develop methodologies to investigate the mechanism of biogenesis and to identify the cellular functions of the *Ectocarpus* miRNA loci. This study did not find any evidence for differential expression of miRNA loci in males or females or in the different generations of the life cycle. Additional analyses will be required to determine whether these genes are regulated in response to other stimuli or coincidentally with other developmental events. Another important future question concerns the evolutionary origins of these loci. Are the miRNA loci conserved in other brown algal species? Did their emergence in the stramenopile lineage predate the evolution of complex multicellularity in this group? At present, genome sampling within the stramenopiles is too sparse to allow this type of question to be addressed, but this situation is likely to change rapidly in the coming years.

Finally, there is a danger that the proliferation, in recent years, of poorly substantiated reports of miRNAs from diverse eukaryotic species, often based on the application of inappropriate methodologies, will obscure the deep evolutionary history of these key regulatory molecules. We demonstrate here the importance of combining deep sRNA read data with stringent selection criteria and a reference genome sequence for the unambiguous detection and validation of miRNA loci. We hope that this study will contribute towards the development of a generally adopted, rigorous miRNA validation mechanism and thereby, in the longer term, to an improved understanding of miRNA evolution within the eukaryotic tree.

### Note added in proof

Following submission to miRBase, an additional family of two members (esi-MIR11396a and esi-MIR11396b) was identified based on similarity between hairpin sequences bringing the number of miRNA families to 62.

## ACCESSION NUMBER

SRP052304.

## SUPPLEMENTARY DATA

Supplementary Data are available at NAR Online.

SUPPLEMENTARY DATA
