# Supplementary material for: microRNAs and the evolution of complex multicellularity: identification of a large, diverse complement of microRNAs in the brown alga Ectocarpus
Source: Nucleic Acids Res. 2015 Jun 22;43(13):6384–98. doi: 10.1093/nar/gkv578 (PMC4513859; doi:10.1093/nar/gkv578)
Supplement: SUPPLEMENTARY DATA [file supp_43_13_6384__index.html]

microRNAs and the evolution of complex multicellularity: identification of a large, diverse complement of microRNAs in the brown alga Ectocarpus — SUPPLEMENTARY DATA 

# microRNAs and the evolution of complex multicellularity: identification of a large, diverse complement of microRNAs in the brown alga *Ectocarpus*

## SUPPLEMENTARY DATA

- SUPPLEMENTARY DATA
- SUPPLEMENTARY DATA
- SUPPLEMENTARY DATA
- SUPPLEMENTARY DATA
