# Supplementary material for: microRNAs and the evolution of complex multicellularity: identification of a large, diverse complement of microRNAs in the brown alga Ectocarpus
Source: Nucleic Acids Res. 2015 Jun 22;43(13):6384–98. doi: 10.1093/nar/gkv578 (PMC4513859; doi:10.1093/nar/gkv578)
Supplement: SUPPLEMENTARY DATA [file supp_gkv578_Ectocarpus_miRNA_supplementary_NAR_3jun15.docx]

Supplementary data for:

**microRNAs and the evolution of complex multicellularity: identification of a large, diverse complement of microRNAs in the brown alga *Ectocarpus***

James E. Tarver^1,2^, Alexandre Cormier^3^, Natalia Pinzón^4^, Richard S. Taylor^1^, Wilfrid Carré^3^, Martina Strittmatter^3^, Hervé Seitz^4^, Susana M. Coelho^3^ and J. Mark Cock^3^

^1^School of Earth Sciences, University of Bristol, Life Sciences Building, 24 Tyndall Avenue, Bristol BS8 1TQ, UK

^2^Genome Evolution Laboratory, Department of Biology, The National University of Ireland, Maynooth, Kildare, Ireland

^3^Sorbonne Université, UPMC Univ Paris 06, CNRS, Algal Genetics Group, UMR 8227, Integrative Biology of Marine Models, Station Biologique de Roscoff, CS 90074, F-29688, Roscoff, France

^4^Institute of Human Genetics, UPR 1142, CNRS, 34396 Montpellier Cedex 5, France

**SUPPLEMENTARY TABLES**

**Table S1.** Small RNA sequence data used in this study.

**Table S2.** *Ectocarpus* miRNA loci. Genome localisation, structural information and expression data for the 64 good quality candidate miRNA loci. The four quality criteria (Crit. 1 to Crit. 4) that were applied to select valid miRNA loci listed are described in the main text. 3p, 3 prime; 5p, 5 prime; LG, linkage group; sctg, supercontig; S, sense; AS, antisense.

**Table S3.** Putative mRNA targets of *Ectocarpus* miRNAs. Genes marked with one or two asterisks are predicted to be targets of either two identical or two different miRNAs, respectively.

**SUPPLEMENTARY FIGURES**

**Figure S1.** Mapping of sRNA sequence data and miRNA loci onto the *Ectocarpus* genome. The histograms indicate the number of sRNA reads that mapped to each 25 kbp window along the 28 linkage groups of the *Ectocarpus* genome, which are represented as pseudochromosomes, i.e. ordered concatenations of genetically linked supercontigs (1,2). A total of 37,764,959 reads were mapped to the genome (Table S1). Vertical lines with a pinhead indicate the map positions of 46 of the 64 miRNA loci. Note the absence of any marked miRNA clustering. Eighteen of the miRNA loci are on supercontigs that have not been mapped to linkage groups and are therefore not shown here. miRBase IDs are indicated as in the following example: 11346, esi-MIR11346. LG, linkage group.

**Figure S2.** *Ectocarpus* miRNA loci. Representations of sRNA read data mapping for the 64 good quality candidate miRNA loci in *Ectocarpus*. The most abundant 5p and 3p products are indicated in blue and red, respectively.

**Figure S3.** Additional loci in the *Ectocarpus* genome that resemble miRNA loci. Representations of sRNA read data mapping and the positions of the miRNA and miRNA* (both marked with an asterisk) together with the predicted hairpin for an additional 65 *Ectocarpus* loci that fulfilled the majority of the criteria used to define miRNA loci but which are located in genomic regions consisting of complex, extensive palindromic sequences that generate multiple sRNA species over a region of several hundred base pairs.

**Figure S4.** Results of the sRNA mapping analysis for the 23 miRNA loci proposed by Billoud *et al.* (3). The miRNA sequences reported by Billoud *et al.* are indicated in green. The most abundant reads for each arm of the hairpin are indicated in red (5p) and blue (3p).

**SUPPLEMENTARY REFERENCES**

1. Heesch, S., Cho, G.Y., Peters, A.F., Le Corguillé, G., Falentin, C., Boutet, G., Coëdel, S., Jubin, C., Samson, G., Corre, E. *et al.* (2010) A sequence-tagged genetic map for the brown alga *Ectocarpus siliculosus* provides large-scale assembly of the genome sequence. *New Phytol*, **188**, 42-51.

2. Cock, J.M., Sterck, L., Rouzé, P., Scornet, D., Allen, A.E., Amoutzias, G., Anthouard, V., Artiguenave, F., Aury, J., Badger, J. *et al.* (2010) The *Ectocarpus* genome and the independent evolution of multicellularity in brown algae. *Nature*, **465**, 617-621.

3. Billoud, B., Nehr, Z., Le Bail, A. and Charrier, B. (2014) Computational prediction and experimental validation of microRNAs in the brown alga *Ectocarpus siliculosus*. *Nucleic Acids Res*, **42**, 417-429.
