# Supplementary material for: microRNAs and the evolution of complex multicellularity: identification of a large, diverse complement of microRNAs in the brown alga Ectocarpus
Source: Nucleic Acids Res. 2015 Jun 22;43(13):6384–98. doi: 10.1093/nar/gkv578 (PMC4513859; doi:10.1093/nar/gkv578)
Supplement: SUPPLEMENTARY DATA [file supp_gkv578_FigS2_S3_S4.pdf]

Supplementary file containing Figures S2, S3 and S4

Figure S2. *Ectocarpus* miRNA loci. Representations of sRNA read data mapping for the 64 good quality candidate miRNA loci in *Ectocarpus*. The most highly expressed read on the 5' arm is highlighted in blue and on the 3' arm in red.

Figure S3. Additional loci in the *Ectocarpus* genome that resemble miRNA loci. Representations of sRNA read data mapping and the positions of the miRNA and miRNA* (both marked with an asterisk) together with the predicted hairpin. These candidates fulfilled the majority of the criteria used to define miRNA loci but were located in genomic regions consisting of complex, extensive palindromic sequences that generate multiple sRNA species over a region of several hundred base pairs.

Figure S4. Results of the sRNA mapping analysis for the 23 miRNA loci proposed by Billoud *et al.* 2014 are indicated in green. The most abundant reads for each arm of the hairpin are indicated in red (5p) and blue (3p).

Figure S2. Ectocarpus miRNA loci. Representations of sRNA read data mapping for the 64 good quality candidate miRNA loci in Ectocarpus.

```
esi-MIR3453

1        10         20         30         40         50         60         70         80         90         100
GACGAACAGCUUCUCCUCGAUCGCCCGCCUGCACAAGUAUAUAUACAAGCCUAUCGUACCAUAUACUUGUGCAGGCGGACGAUUGAGGAGAAGUUGUCCGUCC
((((.((((((((((((((((((.((((((((((((((((((.(((.........)))..)))))))))))))))))).)))))))))))))))))).)))).
.........CUUCUCCUCGAUCGCCCGCCU.........................................................................x5
..........UUCUCCUCGAUCGCCCGCCUG........................................................................x1449*
..........UUCUCCUCGAUCGCCCGCCU.........................................................................x1154
..........UUCUCCUCGAUCGCCCGCC..........................................................................x107
..........UUCUCCUCGAUCGCCCGC...........................................................................x38
..........UUCUCCUCGAUCGCCCGCCUGC.......................................................................x31
..........UUCUCCUCGAUCGCCCGCCUGCA......................................................................x5
...........UCUCCUCGAUCGCCCGCCUGC.......................................................................x152
...........UCUCCUCGAUCGCCCGCCUG........................................................................x34
...........UCUCCUCGAUCGCCCGCCU.........................................................................x22
...........UCUCCUCGAUCGCCCGCCUGCA......................................................................x5
...........UCUCCUCGAUCGCCCGCC..........................................................................x3
............CUCCUCGAUCGCCCGCCU.........................................................................x9
............CUCCUCGAUCGCCCGCCUG........................................................................x2
.............UCCUCGAUCGCCCGCCUGCA......................................................................x4
.............UCCUCGAUCGCCCGCCUGC.......................................................................x1
.............UCCUCGAUCGCCCGCCUG........................................................................x1
........................................................................AGGCGGACGAUUGAGGAGAAG..........x4
........................................................................AGGCGGACGAUUGAGGAGAA...........x2
........................................................................AGGCGGACGAUUGAGGAGA............x1
.........................................................................GGCGGACGAUUGAGGAGAAGU.........x164
.........................................................................GGCGGACGAUUGAGGAGAAG..........x17
.........................................................................GGCGGACGAUUGAGGAGAAGUU........x16
.........................................................................GGCGGACGAUUGAGGAGA............x3
.........................................................................GGCGGACGAUUGAGGAGAA...........x1
..........................................................................GCGGACGAUUGAGGAGAAGU.........x1

Sequence   1 Structure      1
 ENERGY =  -70.7    1
Bases    1 to  103

         10         20         30         40         50
-    A                  C                  A-   AAGC
 GACG ACAGCUUCUCCUCGAUCG CCGCCUGCACAAGUAUAU  UAC    \
 CUGC UGUUGAAGAGGAGUUAGC GGCGGACGUGUUCAUAUA  AUG    C
C    C                  A                  CC   CUAU
 100        90        80        70        60
 __________________________________________________
```

Esi-MIR3455b

```
1       10        20        30        40        50        60        70        80        90       100
GGCCAUCAUCCUCAGGUUCCACCGGAGAUUGAACUCCAGCCAAGCCGAGAGCUUGCUCGAGUUUCAACCUCCGGUGGAACUUGAGGACGGUGGCCA
(((((((.(((((((((((((((((((.((((((((.(((.((((.....))))))).))).))))).))))))))))))))))))).))))))).
...CAUCAUCCUCAGGUUCCACCGGA......................................................................x1
.....UCAUCCUCAGGUUCCACCGGA......................................................................x37638*
.....UCAUCCUCAGGUUCCACCGGAG.....................................................................x1510
.....UCAUCCUCAGGUUCCACCGG.......................................................................x770
.....UCAUCCUCAGGUUCCACCGGAGA....................................................................x28
.....UCAUCCUCAGGUUCCACCG........................................................................x11
.....UCAUCCUCAGGUUCCACC.........................................................................x3
......CAUCCUCAGGUUCCACCGGA......................................................................x258
......CAUCCUCAGGUUCCACCGG.......................................................................x1
......CAUCCUCAGGUUCCACCGGAG.....................................................................x1
.......AUCCUCAGGUUCCACCGGA......................................................................x196
.......AUCCUCAGGUUCCACCGGAG.....................................................................x2
........UCCUCAGGUUCCACCGGA......................................................................x1
.......................................................................CGGUGGAACUUGAGGACGGUG....x441*
.......................................................................CGGUGGAACUUGAGGACGGU.....x62
.......................................................................CGGUGGAACUUGAGGACGG......x56
.......................................................................CGGUGGAACUUGAGGACG.......x5
```

```
Sequence   1 Structure      1
 ENERGY =  -68.8    1
Bases    1 to   96

         10        20        30         40
-       A                   A     -   C   C    CG
 GGCCAUC UCCUCAGGUUCCACCGGAG UUGAA CUC AGC AAGC  \
 CCGGUGG AGGAGUUCAAGGUGGCCUC AACUU GAG UCG UUCG  A
A       C                   C     U   C   -    AG
     90        80        70        60         50
__________________________________________________
```

esi-MIR3457

```
1       10        20        30        40        50        60        70        80        90       100       110       120       130       140       150
CGGUGUUGCUGAAUGCCAGGACAAGCUAAACGAAUAAACUUCGAGUUUGGCAUUCGCGCACUGCUUGGCGUUGAGGUCAAACCGUGCGCGAAUGUCAAACUCGAAGUGUAUUCGCUUAGCUUGUCCUGGGAUUCCGUAGCAUCU
.((((((((.((((.((((((((((((((.((((((.((((((((((((((((((((((((.(.(((((......))))).).)))))))))))))))))))))))).)))))).)))))))))))))).)))).)))))))).
......UGCUGAAUGCCAGGACAAGCU.....................................................................................................................x8
......UGCUGAAUGCCAGGACAAGC......................................................................................................................x1
......UGCUGAAUGCCAGGACAAGCUA....................................................................................................................x1
........CUGAAUGCCAGGACAAGCUAA...................................................................................................................x18
........CUGAAUGCCAGGACAAGCUAAA..................................................................................................................x4
........CUGAAUGCCAGGACAAGCUA....................................................................................................................x1
.........UGAAUGCCAGGACAAGCUAAA..................................................................................................................x4983*
.........UGAAUGCCAGGACAAGCUAA...................................................................................................................x411
.........UGAAUGCCAGGACAAGCUAAAC.................................................................................................................x26
.........UGAAUGCCAGGACAAGCUA....................................................................................................................x26
.........UGAAUGCCAGGACAAGCU.....................................................................................................................x5
.........UGAAUGCCAGGACAAGCUAAACG................................................................................................................x1
..........GAAUGCCAGGACAAGCUAAA..................................................................................................................x9
..........GAAUGCCAGGACAAGCUAA...................................................................................................................x1
...........AAUGCCAGGACAAGCUAAA..................................................................................................................x2
...........AAUGCCAGGACAAGCUAA...................................................................................................................x1
.............UGCCAGGACAAGCUAAACGAA..............................................................................................................x1
..............................CGAAUAAACUUCGAGUUUGGC.............................................................................................x10
...................................................AUUCGCGCACUGCUUGGCGUU........................................................................x1
.........................................................................AGGUCAAACCGUGCGCGAAUGU.................................................x1
..............................................................................................UCAAACUCGAAGUGUAUUCGC.............................x154
..............................................................................................UCAAACUCGAAGUGUAUUCGCU............................x20
..............................................................................................UCAAACUCGAAGUGUAUUCG..............................x3
```

```
..............................................................................................UCAAACUCGAAGUGUAUU................................x1
...............................................................................................CAAACUCGAAGUGUAUUCGCU............................x529
...............................................................................................CAAACUCGAAGUGUAUUCG..............................x2
...............................................................................................CAAACUCGAAGUGUAUUCGC.............................x2
...............................................................................................CAAACUCGAAGUGUAUUCGCUUA..........................x2
................................................................................................AAACUCGAAGUGUAUUCGCU............................x2
.................................................................................................AACUCGAAGUGUAUUCGCU............................x1
............................................................................................................UAUUCGCUUAGCUUGUCCUGG...............x2
............................................................................................................UAUUCGCUUAGCUUGUCCUGGG..............x1
..............................................................................................................UUCGCUUAGCUUGUCCUGGGA.............x4
..............................................................................................................UUCGCUUAGCUUGUCCUGGGAU............x2
...............................................................................................................UCGCUUAGCUUGUCCUGGGA.............x2
...............................................................................................................UCGCUUAGCUUGUCCUGGGAUU...........x1
..................................................................................................................CUUAGCUUGUCCUGGGAUUCCGU.......x5
..................................................................................................................CUUAGCUUGUCCUGGGAUUCC.........x2
..................................................................................................................CUUAGCUUGUCCUGGGAUUCCG........x2
...................................................................................................................UUAGCUUGUCCUGGGAUUCCG........x5672
...................................................................................................................UUAGCUUGUCCUGGGAUUCCGU.......x4461
...................................................................................................................UUAGCUUGUCCUGGGAUUCC.........x372
...................................................................................................................UUAGCUUGUCCUGGGAUUCCGUA......x55
...................................................................................................................UUAGCUUGUCCUGGGAUU...........x39
...................................................................................................................UUAGCUUGUCCUGGGAUUC..........x37
...................................................................................................................UUAGCUUGUCCUGGGAUUCCGUAG.....x5
....................................................................................................................UAGCUUGUCCUGGGAUUCCGU.......x281301*
....................................................................................................................UAGCUUGUCCUGGGAUUCCGUA......x32567
....................................................................................................................UAGCUUGUCCUGGGAUUCCG........x3432
....................................................................................................................UAGCUUGUCCUGGGAUUCCGUAG.....x689
....................................................................................................................UAGCUUGUCCUGGGAUUCC.........x123
....................................................................................................................UAGCUUGUCCUGGGAUUC..........x15
....................................................................................................................UAGCUUGUCCUGGGAUUCCGUAGC....x15
....................................................................................................................UAGCUUGUCCUGGGAUUCCGUAGCAU..x3
.....................................................................................................................AGCUUGUCCUGGGAUUCCGU.......x735
.....................................................................................................................AGCUUGUCCUGGGAUUCCGUA......x104
.....................................................................................................................AGCUUGUCCUGGGAUUCCG........x30
.....................................................................................................................AGCUUGUCCUGGGAUUCCGUAG.....x2
......................................................................................................................GCUUGUCCUGGGAUUCCGU.......x107
......................................................................................................................GCUUGUCCUGGGAUUCCGUA......x27
......................................................................................................................GCUUGUCCUGGGAUUCCG........x3
.......................................................................................................................CUUGUCCUGGGAUUCCGU.......x18
.......................................................................................................................CUUGUCCUGGGAUUCCGUA......x4
........................................................................................................................UUGUCCUGGGAUUCCGUA......x16
........................................................................................................................UUGUCCUGGGAUUCCGUAGCA...x4
........................................................................................................................UUGUCCUGGGAUUCCGUAGC....x2
........................................................................................................................UUGUCCUGGGAUUCCGUAG.....x1
........................................................................................................................UUGUCCUGGGAUUCCGUAGCAU..x1
.........................................................................................................................UGUCCUGGGAUUCCGUAGCAU..x3
...........................................................................................................................UCCUGGGAUUCCGUAGCAUCUx2
```

```
Sequence    1 Structure       1
 ENERGY =  -99.9    1
Bases    1 to  144

        10        20        30        40        50        60        70
C        U    G              A      A                        U C     GU
 GGUGUUGC GAAU CCAGGACAAGCUAA CGAAUA ACUUCGAGUUUGGCAUUCGCGCAC G UUGGC  U
 CUACGAUG CUUA GGUCCUGUUCGAUU GCUUAU UGAAGCUCAAACUGUAAGCGCGUG C AACUG  G
U        C    G              C      G                        C A     GA
  140       130       120       110       100        90        80
```

esi-MIR3458

```
1       10         20         30         40         50         60         70         80         90         100
UUGGAAGGACUUUCUGUUGGACGCAUAUUGGGCACUGCUUCAACUUGCUGCGCAUUAUAGCAGUACCCAAUGUGCGUCCAAAAGAUCGUCCUUCCAC
.(((((((((..(((.((((((((((((((((.((((((......(((...)))....)))))).)))))))))))))))))).)))..))))))))).
......GGACUUUCUGUUGGACGCAUA......................................................................x105*
......GGACUUUCUGUUGGACGCAU.......................................................................x18
.......GACUUUCUGUUGGACGCAUA......................................................................x1
........................................................................UGCGUCCAAAAGAUCGUCCUU....x1675*
........................................................................UGCGUCCAAAAGAUCGUCCU.....x60
........................................................................UGCGUCCAAAAGAUCGUCCUUC...x4
........................................................................UGCGUCCAAAAGAUCGUC.......x3
.........................................................................GCGUCCAAAAGAUCGUCCUU....x11
...........................................................................GUCCAAAAGAUCGUCCUU....x1

Sequence   1 Structure      1
 ENERGY =  -59.2    1
Bases    1 to   97

        10        20        30        40
U         UU   G                C      UCAACU   U
 UGGAAGGAC  UCU UUGGACGCAUAUUGGG ACUGCU      UGC \
 ACCUUCCUG  AGA AACCUGCGUGUAACCC UGACGA      ACG G
C         CU   A                A      UAUU--   C
      90        80        70        60
 __________________________________________________
```

esi-MIR3460

```
1       10         20         30         40         50         60         70         80         90         100        110        120
ACTTACGATGTAAGGGAATCTGCAGCTTGTGAGGTGCTGCAGAAAACCCCCCAGGCGCTGGGTTTTTCTTCTGCCGCACCTCACGAGCTGCAGGTTCCCTTACATCGCAAG
.(((.((((((((((((((((((((((((((((((((.((((((.....(((((...)))))......)))))).)))))))))))))))))))))))))))))))).)))
.............*********************..............................................XXXXXXXXXXXXXXXXXXXXX..........
..TTACGATGTAAGGGAATCTG.........................................................................................x1.(0.01%)
..TTACGATGTAAGGGAATCTGC........................................................................................x26.(0.28%)
...TACGATGTAAGGGAATCTGC........................................................................................x2.(0.02%)
...TACGATGTAAGGGAATCTGCA.......................................................................................x2.(0.02%)
........TGTAAGGGAATCTGCAGCTTGTGAGG.............................................................................x1.(0.01%)
..........TAAGGGAATCTGCAGCTTGTG................................................................................x14.(0.15%)
...........AAGGGAATCTGCAGCTTGTGA...............................................................................x1.(0.01%)
............AGGGAATCTGCAGCTTGTGAGG.............................................................................x1.(0.01%)
.............GGGAATCTGCAGCTTGTGAGG.............................................................................x31.(0.34%)*
.............GGGAATCTGCAGCTTGTGAG..............................................................................x1.(0.01%)
.............GGGAATCTGCAGCTTGTG................................................................................x1.(0.01%)
.............GGGAATCTGCAGCTTGTGAGGT............................................................................x1.(0.01%)
...............................................................................CTCACGAGCTGCAGGTTCCCTT..........x5.(0.05%)
................................................................................TCACGAGCTGCAGGTTCC.............x4.(0.04%)
................................................................................TCACGAGCTGCAGGTTCCCTT..........x8380.(91.11%)*
................................................................................TCACGAGCTGCAGGTTCCC............x17.(0.18%)
................................................................................TCACGAGCTGCAGGTTCCCT...........x475.(5.16%)
................................................................................TCACGAGCTGCAGGTTCCCTTA.........x126.(1.37%)
.................................................................................CACGAGCTGCAGGTTCCCTTA.........x1.(0.01%)
.................................................................................CACGAGCTGCAGGTTCCCT...........x1.(0.01%)
.................................................................................CACGAGCTGCAGGTTCCCTT..........x46.(0.5%)
..................................................................................ACGAGCTGCAGGTTCCCT...........x1.(0.01%)
..................................................................................ACGAGCTGCAGGTTCCCTT..........x11.(0.12%)
..................................................................................ACGAGCTGCAGGTTCCCTTA.........x1.(0.01%)
...................................................................................CGAGCTGCAGGTTCCCTT..........x1.(0.01%)
........................................................................................TGCAGGTTCCCTTACATCGCA..x47.(0.51%)

Sequence 1 Structure 1
  ENERGY = -81.0 1
Bases 1 to 11
```

```
        10        20        30        40         50
A   A                                    U      AACCC-     G
 CUU CGAUGUAAGGGAAUCUGCAGCUUGUGAGGUGC GCAGAA      CCCAG \
 GAA GCUACAUUCCCUUGGACGUCGAGCACUCCACG CGUCUU      GGGUC C
-   C                                    C      CUUUUU     G
110       100        90        80        70         60

__________________________________________________
```

esi-MIR3466

```
1       10        20        30        40        50        60        70        80        90        100       110       120       130       140       150
AUCUGUCUCUUCCGGAGGACUACCAUCACACUAGCGUUUUUGUACCACGUGUUGACCAUGCAGGGCAUCAGUGUUGGCGCCGUUUCUGUUCAACACGUGGUAAGAAAAAGCUUUUGUGAUGGUCGUCCUCCGAAAGGGCCAGGA
.((((.(((((.((((((((.(((((((((..(((.(((((.(((((((((((((.((...(.(((.(((....))).)))).)...)).)))))))))))).))))).)))..))))))))).)))))))).))))).)))).
.......UCUUCCGGAGGACUACCAUCA....................................................................................................................x1
........CUUCCGGAGGACUACCAUCACA..................................................................................................................x3
.........UUCCGGAGGACUACCAUCACA..................................................................................................................x1124*
.........UUCCGGAGGACUACCAUCAC...................................................................................................................x31
.........UUCCGGAGGACUACCAUCA....................................................................................................................x3
.........UUCCGGAGGACUACCAUC.....................................................................................................................x2
.........UUCCGGAGGACUACCAUCACAC.................................................................................................................x2
..........UCCGGAGGACUACCAUCACA..................................................................................................................x7
............CGGAGGACUACCAUCACA..................................................................................................................x6
...............................................................................................CGUGGUAAGAAAAAGCUUUUG............................x1
....................................................................................................................UGAUGGUCGUCCUCCGAAAGG.......x173*
....................................................................................................................UGAUGGUCGUCCUCCGAAAG........x45
....................................................................................................................UGAUGGUCGUCCUCCGAA..........x3
....................................................................................................................UGAUGGUCGUCCUCCGAAA.........x3
```

```
Sequence   1 Structure      1
 ENERGY =  -79.2    1
Bases     1 to  144

         10        20        30        40        50         60        70
A    U     C        U         CU   G     G             C  UGC G   A   G
 UCUG CUCUU CGGAGGAC ACCAUCACA  AGC UUUUU UACCACGUGUUGA CA   A GGC UCA U
 GGAC GGGAA GCCUCCUG UGGUAGUGU  UCG AAAAG AUGGUGCACAACU GU   U CCG GGU G
A    C     A        C         UU   A     A             U  CUU G   C   U
  140       130       120       110       100         90        80

 __________________________________________________
```

esi-MIR3468

```
1       10        20        30        40        50        60        70        80        90        100       110       120       130       140
CAAGAAGCGCAGCGAAAAUGGACGAGAAGAAGAAAACAGCGGAGGCUGCAACAGAGACAUGUAUAUGAAAAUUCUGGUUACCCAUGCCUGCGUUGCAGCCUCCACUGAUUUCUUCUUCUUUUCCAGGUUGGCUGCGUUUUUUU
.((((((((((((.((..((((.((((((((((((.(((.((((((((((((..((.((((...((.(.......).))....)))).))..))))))))))).))).)))))))))))).))))..)).)))))))))))).
........GCAGCGAAAAUGGACGAGAAG..................................................................................................................x2
........GCAGCGAAAAUGGACGAGAAGA.................................................................................................................x2
........GCAGCGAAAAUGGACGAGAA...................................................................................................................x1
.........CAGCGAAAAUGGACGAGAAGA.................................................................................................................x24*
...........GCGAAAAUGGACGAGAAGAAG...............................................................................................................x1
......................CGAGAAGAAGAAAACAGCGGA....................................................................................................x1
.............................................................................................UGCAGCCUCCACUGAUUUCUU.............................x12
.............................................................................................UGCAGCCUCCACUGAUUUCU..............................x1
.............................................................................................UGCAGCCUCCACUGAUUUCUUC............................x1
...............................................................................................CAGCCUCCACUGAUUUCUUCUU..........................x1
...............................................................................................CAGCCUCCACUGAUUUCUUCU...........................x1
```

```
....................................................................................................UCCACUGAUUUCUUCUUCUUU......................x1
...................................................................................................................UUCUUUUCCAGGUUGGCUGCG.......x718*
...................................................................................................................UUCUUUUCCAGGUUGGCUGC........x24
...................................................................................................................UUCUUUUCCAGGUUGGCUGCGU......x3
...................................................................................................................UUCUUUUCCAGGUUGGCUG.........x3
...................................................................................................................UUCUUUUCCAGGUUGGCU..........x2
....................................................................................................................UCUUUUCCAGGUUGGCUGCGU......x94
....................................................................................................................UCUUUUCCAGGUUGGCUGCG.......x2
....................................................................................................................UCUUUUCCAGGUUGGCUGCGUU.....x1
......................................................................................................................UUUUCCAGGUUGGCUGCG.......x1
........................................................................................................................UUCCAGGUUGGCUGCGUUUUUU.x1
```

```
Sequence    1 Structure       1
 ENERGY =  -77.2     1
Bases     1 to  143

        10        20        30        40        50        60          70
C            G  AA    C            A   C            AG  A    UAU-  G AA
 AAGAAGCGCAGC AA  UGGA GAGAAGAAGAAA CAG GGAGGCUGCAAC  AG CAUG    AU A  A
 UUUUUUGCGUCG UU  ACCU UUCUUCUUCUUU GUC CCUCCGACGUUG  UC GUAC    UG U  U
U            G  GG    U            A   A            CG  C    CCAU  G CU
 140       130       120       110       100         90        80
 __________________________________________________
```

Esi-MIR3469b

```
1        10        20        30        40        50        60        70        80        90        100
CACUCUCCGCCUCUGGGGUGGACAUGUGAACACCGUCGCCAUCCAACAGUACGCAUGUCGAGGAUGACGACGGUGUUCACAUAUCCACCCCAGAGGCGGAGG
...(((((((((((((((((((.(((((((((((((((.(((((.(((.......)))...))))).))))))))))))))).)))))))))))))))))))
..........CUCUGGGGUGGACAUGUGAAC.......................................................................x6
..........CUCUGGGGUGGACAUGUGAA........................................................................x1
...........UCUGGGGUGGACAUGUGAACA......................................................................x308*
...........UCUGGGGUGGACAUGUGAAC.......................................................................x31
...........UCUGGGGUGGACAUGUGAA........................................................................x2
...........UCUGGGGUGGACAUGUGA.........................................................................x2
...........UCUGGGGUGGACAUGUGAACAC.....................................................................x1
............CUGGGGUGGACAUGUGAACA......................................................................x2
............CUGGGGUGGACAUGUGAAC.......................................................................x1
..................UGGACAUGUGAACACCGUCGC...............................................................x4
.........................................................................UGUUCACAUAUCCACCCCAGA........x12
...........................................................................UUCACAUAUCCACCCCAGAGG......x242*
...........................................................................UUCACAUAUCCACCCCAGAG.......x5
...........................................................................UUCACAUAUCCACCCCAGA........x1
............................................................................UCACAUAUCCACCCCAGAGGC.....x55
............................................................................UCACAUAUCCACCCCAGAGG......x8
............................................................................UCACAUAUCCACCCCAGA........x1
............................................................................UCACAUAUCCACCCCAGAG.......x1
```

```
Sequence    1 Structure       1
 ENERGY =  -79.8     1
Bases     1 to  102

        10        20        30        40          50
CAC                   C               C     A--   GUA
   UCUCCGCCUCUGGGGUGGA AUGUGAACACCGUCG CAUCC   ACA   \
   GGAGGCGGAGACCCCACCU UACACUUGUGGCAGC GUAGG   UGU   C
---                   A               A     AGC   ACG
   100        90        80        70        60
 __________________________________________________
```

Esi-MIR11345

```
1        10        20        30        40        50        60        70        80        90        100       110       120
```

```
UAGGUGCUACGCUCCGUCUGUAGUGAGGUUCCAAGCUUUUUGUCCGCAUGCCCGUCACCCUGUGCAGACGUACAAACAGCUUGCGGUUUGACUACAGAGGGAGCAGAACCUACCUAGACCU
(((((.((..(((((.((((((((.(((((.((((((.(((((.((..(((.((......)).)))..)).))))).)))))).))))).)))))))).))))))).))))).........
...............GUCUGUAGUGAGGUUCCAAGC.....................................................................................x4*
....................................UUUUUGUCCGCAUGCCCGUCAC...............................................................x2
......................................UUUGUCCGCAUGCCCGUCAC...............................................................x1
............................................CGCAUGCCCGUCACCCUGUGCAGAC....................................................x1
................................................................................UUGCGGUUUGACUACAGAGGG....................x1021*
................................................................................UUGCGGUUUGACUACAGAGGGA...................x31
................................................................................UUGCGGUUUGACUACAGAGG.....................x11
................................................................................UUGCGGUUUGACUACAGAG......................x9
................................................................................UUGCGGUUUGACUACAGA.......................x2
.................................................................................UGCGGUUUGACUACAGAGGG....................x4
..................................................................................GCGGUUUGACUACAGAGGG....................x2
..................................................................................GCGGUUUGACUACAGAGG.....................x1
......................................................................................................GCAGAACCUACCUAGACC.x1

Sequence   1 Structure      1
 ENERGY =  -49.5    1
Bases    1 to  121

                 10        20        30        40        50
---------     G  AC      G        G     C      U     C  CA    C  UC
         UAGGU CU  GCUCC UCUGUAGU AGGUU CAAGCU UUUGU CG  UGC CG  A
         AUCCA GA  CGAGG AGACAUCA UUUGG GUUCGA AAACA GC  ACG GU  C
UCCAGAUCC     A  --      G        G     C      C     U  AG    U  CC
 .       110         100        90        80        70        60
 __________________________________________________
```

Esi-MIR11346

```
1       10        20        30        40        50        60        70        80        90        100       110       120
UAGAGCGGAAGCAAAUAACCGGUCUUGUGUGGUUGUUCAAGCUUGACUGCGUGGGAAGUCUGACUGCCCAAGCAGCCAAGUUUGAACAAGCACACAAGUCCGGUUAUCACUCGCACUCUUUU
.((((.(..((...((((((((.((((((((.(((((((((((((.((((.((((.(((...))).)))).)))).))))))))))))).)))))))).))))))))..))..).))))...
..................CCGGUCUUGUGUGGUUGUUCA...................................................................................x10*
..................CCGGUCUUGUGUGGUUGUUC....................................................................................x1
....................GGUCUUGUGUGGUUGUUCAAG.................................................................................x1
.........................UGUGUGGUUGUUCAAGCUUGA............................................................................x1
.......................................AGCUUGACUGCGUGGGAAGU...............................................................x1
..................................................................................UGAACAAGCACACAAGUCCGG...................x1
....................................................................................AACAAGCACACAAGUCCGGUU.................x4*
....................................................................................AACAAGCACACAAGUCCGGUUA................x2

Sequence   1 Structure      1
 ENERGY =  -67.8    1
Bases    1 to  122


          10        20        30        40        50        60
U--    C GA   CAA        U        G             A    G    A   C
   AGAG G  AG   AUAACCGG CUUGUGUG UUGUUCAAGCUUG CUGC UGGG AGU \
   UCUC C  UC   UAUUGGCC GAACACAC AACAAGUUUGAAC GACG ACCC UCA U
UUU    A GC   AC-        U        G             C    A    G   G
120       110        100        90        80        70
 __________________________________________________
```

Esi-MIR11347

```
1       10        20        30        40        50        60        70        80        90        100       110       120       130       140       150       160
CCUGAAAGGUUAUCGCCGAUGUCCGUCGAAGAGGAUGGCACUUGAGCCACGUCAGGCGAAAUGGCCUGCAUGUUCGGUUUCUGCAGACCGUUUUGCGUGACGUGGCUCACGGGUCAUCCUCCGUGGUGAACAUCGACGAUAACCUUUCAGACCUUACUGU
.((((((((((((((.((((((.(((((..(((((((((.((((((((((((((.(((((((((.(((((.(........)))))).))))))))).)))))))))))).)))))))))))..))))).)))))).))))))))))))))..........
.........UUAUCGCCGAUGUCCGUCGAAGA................................................................................................................................x2
.........UUAUCGCCGAUGUCCGUCGA...................................................................................................................................x1
..........UAUCGCCGAUGUCCGUCGAA..................................................................................................................................x1
............UCGCCGAUGUCCGUCGAAGAG...............................................................................................................................x382*
```

```
............UCGCCGAUGUCCGUCGAAGA................................................................................................................................x108
............UCGCCGAUGUCCGUCGAAG.................................................................................................................................x19
............UCGCCGAUGUCCGUCGAA..................................................................................................................................x2
............UCGCCGAUGUCCGUCGAAGAGG..............................................................................................................................x1
.............CGCCGAUGUCCGUCGAAGA................................................................................................................................x1
..............GCCGAUGUCCGUCGAAGAG...............................................................................................................................x3
........................................................................................................................CCGUGGUGAACAUCGACGAUA...................x3*

Sequence   1 Structure      1
 ENERGY =  -96.3    1
Bases    1 to  160

                 10        20        30        40         50        60        70
C---------              C      C     AA         A  -            G         C     U UUC
          CUGAAAGGUUAUCG CGAUGU CGUCG  GAGGAUGGC CU UGAGCCACGUCA GCGAAAUGG CUGCA G   G
          GACUUUCCAAUAGC GCUACA GUGGU  CUCCUACUG GG ACUCGGUGCAGU CGUUUUGCC GACGU C   G
UGUCAUUCCA              A      A     GC         -  C            G         A     - UUU
.       150       140       130       120        110       100        90         80
```

Esi-MIR11348

```
1       10        20        30        40        50        60        70        80        90        100       110
GAAACUCCCCUCAGAUCUCACGUCAACGGCACGCCGGUGAUCAACCUUGUCCCAGCUACAAGGUUGAUCACCGGCGUGCCGUCGACGUGAGAUGCGAGGGGAGGCUCUGACG
((..((((((((..((((((((((.(((((((((((((((((((((((((.......))))))))))))))))))))))))).))))))))))..))))))))..)).....
............AGAUCUCACGUCAACGGCACG...............................................................................x2*
............AGAUCUCACGUCAACGGCAC................................................................................x1
...............UCUCACGUCAACGGCACGCCG............................................................................x2
...............UCUCACGUCAACGGCACGCC.............................................................................x1
.......................................................CUACAAGGUUGAUCACCGGCG....................................x1
........................................................UACAAGGUUGAUCACCGGCG....................................x1
............................................................................UGCCGUCGACGUGAGAUGCGA...............x3276*
............................................................................UGCCGUCGACGUGAGAUGCG................x518
............................................................................UGCCGUCGACGUGAGAUGC.................x83
............................................................................UGCCGUCGACGUGAGAUGCGAG..............x76
............................................................................UGCCGUCGACGUGAGAUG..................x1
.............................................................................GCCGUCGACGUGAGAUGCGA...............x3
..............................................................................CCGUCGACGUGAGAUGCGA...............x2
.................................................................................UCGACGUGAGAUGCGAGGGGA..........x1

Sequence   1 Structure      1
 ENERGY =  -89.6    1
Bases    1 to  112

             10        20        30        40        50
-----  AA         AG          A                         CCC
     GA  CUCCCCUC  AUCUCACGUC ACGGCACGCCGGUGAUCAACCUUGU   \
     CU  GAGGGGAG  UAGAGUGCAG UGCCGUGCGGCCACUAGUUGGAACA   A
GCAGU  CG         CG          C                         UCG
110       100        90        80        70        60
```

Esi-MIR11349

```
1       10        20        30        40        50        60        70        80        90        100       110       120       130       140       150       160       170
180
CCCUCUGCUCUACUUUUUAUCUCCUAAUAUACACGAAGUCCUGUGUCAGCCUUUUCUGUGCGGCUCUGACAAAUUGCUGUCCGCCUCAUCGUGUGGCGUACAGCUUGUUGUCAGAAGCGCAAAGAAAAAAAUGACCCCAGGCCCUUGUAUAUAUUUGGAGGAAGGAGUAGGGUUGAGAU
GGA
(((((.((((((((((((.(((((.((((((.((((.(.((((.((((...((((((.((((..((((((((...(((((.((((.((...)).)))).)))))...))))))))..)))).))))))...))))..)))).).)))).)))))).))))))))))))))))).)))..
)).
```

```
.....UGCUCUACUUUUUAUCUCCUAA.........................................................................................................................................................
...x11*
.....UGCUCUACUUUUUAUCUCCUA..........................................................................................................................................................
...x2
.....UGCUCUACUUUUUAUCUCCUAAU........................................................................................................................................................
...x1
...........................UAUACACGAAGUCCUGUGUCA....................................................................................................................................
...x2
...........................................................................................................................................................UGGAGGAAGGAGUAGGGUUGA....
...x32*
...........................................................................................................................................................UGGAGGAAGGAGUAGGGUUG.....
...x18
...........................................................................................................................................................UGGAGGAAGGAGUAGGGUU......
...x3

Sequence    1 Structure       1
 ENERGY =   -84.6     1
Bases     1 to  182

           10        20        30        40         50        60        70        80
-  --   U            A     U      C    A U    U-    GCC      G    GC        AUU     C    U  U
 CC  CUC GCUCUACUUUUU UCUCC AAUAUA ACGA G CCUG  GUCA   UUUUCU UGCG  UCUGACAA   GCUGU CGCC CA \
 GG  GAG UGGGAUGAGGAA GGAGG UUAUAU UGUU C GGAC  CAGU   AAAAGA ACGC  AGACUGUU   CGACA GCGG GU C
A  UA   U            -     U      A    C C    CC    AAA      A    GA        GUU     U    U  G
180       170        160       150       140       130       120       110       100
 __________________________________________________

Esi-MIR11350

1       10        20        30        40        50        60        70        80        90        100       110       120
GAACUGGAAUAUUUUCGAGAUCGGACAGCUUCAGUGAGCGGCAUUCGCUCCAUGAAGCUCAGGGAGCGACAUUUCCUUUCUGAAGCUGUCGGAUCUCAAUGAUAUCCCCGCCUCUCUCUG
.....((.(((((...((((((.(((((((((((.(((.((.(((((((((.(((...))).))))))).)).))))).))))))))))).))))))...))))).))............
.............UUCGAGAUCGGACAGCUUCAG......................................................................................x180*
.............UUCGAGAUCGGACAGCUUCA.......................................................................................x2
.............UUCGAGAUCGGACAGCUUCAGU.....................................................................................x1
..............UCGAGAUCGGACAGCUUCAG......................................................................................x1
................................................................................UGAAGCUGUCGGAUCUCAAUGA..................x2
.................................................................................GAAGCUGUCGGAUCUCAAUG...................x1*

Sequence    1 Structure       1
 ENERGY =   -52.8     1
Bases     1 to  120




               10        20        30        40         50
GAACU-------  A     UUC      G           U   C  C  -       A   A
            GG AUAUU   GAGAUC GACAGCUUCAG GAG GG AU UCGCUCC UGA \
            CC UAUAG   CUCUAG CUGUCGAAGUC UUC CU UA AGCGAGG ACU G
GUCUCUCUCCGC  C     UAA      G           U   -  U  C       G   C
.       110       100        90        80         70        60
 __________________________________________________

Esi-MIR11351

1       10        20        30        40        50        60        70        80        90        100       110
ACUGCCACGGCCCUUCACCCCCUCAUGAACCGGGCCGUGUCGCGCUGCGGGAAAGCAUAGCGACACGGUCCGGUUCAUGAGGUGGUGAAGGGCCGUUGCAGUCAUGACCAUCC
(((((.(((((((((((((.(((((((((((((((((((((((..(((......)))..))))))))))))))))))))))).))))))))))))).)))))...........
.........GCCCUUCACCCCCUCAUGAAC...................................................................................x4*
.......................UCAUGAACCGGGCCGUGUCGC.....................................................................x2
.........................................................................UUCAUGAGGUGGUGAAGGG.....................x53
.........................................................................UUCAUGAGGUGGUGAAGGGC....................x15
```

```
.........................................................................UUCAUGAGGUGGUGAAGGGCC...................x3
.........................................................................UUCAUGAGGUGGUGAAGG......................x1
..........................................................................UCAUGAGGUGGUGAAGGGCCG..................x2410*
..........................................................................UCAUGAGGUGGUGAAGGGCC...................x1224
..........................................................................UCAUGAGGUGGUGAAGGG.....................x931
..........................................................................UCAUGAGGUGGUGAAGGGC....................x514
..........................................................................UCAUGAGGUGGUGAAGGGCCGU.................x36
..........................................................................UCAUGAGGUGGUGAAGGGCCGUU................x9
..........................................................................UCAUGAGGUGGUGAAGGGCCGUUG...............x1
...........................................................................CAUGAGGUGGUGAAGGGCCG..................x3
...........................................................................CAUGAGGUGGUGAAGGGC....................x2
...........................................................................CAUGAGGUGGUGAAGGGCC...................x1
............................................................................AUGAGGUGGUGAAGGGCCG..................x4
............................................................................AUGAGGUGGUGAAGGGCC...................x1
..................................................................................UGGUGAAGGGCCGUUGCAGUCA.........x1

Sequence    1 Structure       1
 ENERGY =  -91.9     1
Bases     1 to  113

                   10        20        30        40        50
-----------     C             C                       GC    GG
           ACUGC ACGGCCCUUCACC CCUCAUGAACCGGGCCGUGUCGC  UGC  G
           UGACG UGCCGGGAAGUGG GGAGUACUUGGCCUGGCACAGCG  ACG  A
CCUACCAGUAC     U             U                       AU    AA
 110       100        90        80        70        60
 __________________________________________________

Esi-MIR11352

1        10        20        30        40        50        60        70        80        90        100       110       120       130       140       150
GCAUCCGCAUCUAGAAGUUCCUUGGCGACUUCUUUGAUCGGACAUGUUUCUGCGUCCCGUUCAGCUGGUUCAUUAGCCAGCUGAACGGGAAACAGCAAUGUCAUCGAUAGAAGAAGACGCCGACGGACUGCUUGAGGCGGAUGCGACCGCAGC
((((((((.((.((.(((((.((((((.(((((((.(((((((((....(((..((((((((((((((((....))))))))))))))))..)))..)))))..)))).))))))).)))))).))))).)).)).)))))))).........
.........UCUAGAAGUUCCUUGGCGACU...........................................................................................................................x2834*
.........UCUAGAAGUUCCUUGGCGAC............................................................................................................................x37
.........UCUAGAAGUUCCUUGGCGA.............................................................................................................................x23
.........UCUAGAAGUUCCUUGGCGACUU..........................................................................................................................x21
.........UCUAGAAGUUCCUUGGCG..............................................................................................................................x1
..........CUAGAAGUUCCUUGGCGACU...........................................................................................................................x6
...........UAGAAGUUCCUUGGCGACU...........................................................................................................................x3
............AGAAGUUCCUUGGCGACU...........................................................................................................................x1
.............................UUCUUUGAUCGGACAUGUUUCU......................................................................................................x51
.............................UUCUUUGAUCGGACAUGUUUC.......................................................................................................x9
.............................UUCUUUGAUCGGACAUGUUUCUG.....................................................................................................x4
.............................UUCUUUGAUCGGACAUGUUU........................................................................................................x1
..............................UCUUUGAUCGGACAUGUUUCU......................................................................................................x141
..............................UCUUUGAUCGGACAUGUUUC.......................................................................................................x16
..............................UCUUUGAUCGGACAUGUUU........................................................................................................x5
..............................UCUUUGAUCGGACAUGUUUCUG.....................................................................................................x1
....................................................................UUCAUUAGCCAGCUGAACGG.................................................................x1
..............................................................................................GCAAUGUCAUCGAUAGAAGA.......................................x1
..............................................................................................GCAAUGUCAUCGAUAGAAGAAG.....................................x1
...................................................................................................................GACGCCGACGGACUGCUUGAGG................x36
...................................................................................................................GACGCCGACGGACUGCUUGAG.................x5
....................................................................................................................ACGCCGACGGACUGCUUGAGG................x200*
....................................................................................................................ACGCCGACGGACUGCUUGAG.................x13
....................................................................................................................ACGCCGACGGACUGCUUG...................x3
....................................................................................................................ACGCCGACGGACUGCUUGA..................x2

Sequence    1 Structure       1
 ENERGY =  -85.4     1
Bases     1 to  153
```

```
                 10         20         30         40          50         60         70
---------        A  U  A      C      A        G    --      GUUU   CG                C
        GCAUCCGC UC AG AGUUC UUGGCG CUUCUUU AUCG  GACAU     CUG  UCCCGUUCAGCUGGUU A
        CGUAGGCG AG UC UCAGG AGCCGC GAAGAAG UAGC  CUGUA     GAC  AGGGCAAGUCGACCGA U
CGACGCCAG        G  U  G      C      A        A     UA     AC--   AA                U
 150       140        130       120       110       100          90         80
```

____

Esi-MIR11353

```
1        10         20         30         40         50         60         70         80         90         100        110        120        130        140        150        160
CGGACGCAUUCAUUCUCGUCACCCACCAACGACCAUGUUUUCAUGGCGUAUGCUCGUGGCGACGAUGAUACGGGUACCACCGUCUUCACCAGCAUAGGCCAUUAGGACCGGCUCGUUGCUGGGUGACGGGUGCGCAUGCCUCGUACUUGCCCCGGUACUCC
..((.((((.(.(.(((((((((((.((((((((..(((((.(((((.((((((.((((.((((.((..........)).)))).)))).)))))).))))).))))).)).)))))).))))))))))).).).)))).))(((((......)))))...
..........CAUUCUCGUCACCCACCAACG..................................................................................................................................x34*
..........CAUUCUCGUCACCCACCAAC...................................................................................................................................x4
...........AUUCUCGUCACCCACCAACG..................................................................................................................................x1
..........................................................................UACCACCGUCUUCACCAGCA...................................................................x2
..............................................................................................UAGGCCAUUAGGACCGGCU................................................x7
..............................................................................................UAGGCCAUUAGGACCGGCUCG..............................................x6
..............................................................................................UAGGCCAUUAGGACCGGCUCGU.............................................x1
...................................................................................................................UUGCUGGGUGACGGGUGCGCA.........................x196*
...................................................................................................................UUGCUGGGUGACGGGUGCG...........................x14
...................................................................................................................UUGCUGGGUGACGGGUGCGCAU........................x13
...................................................................................................................UUGCUGGGUGACGGGUGCGC..........................x12
...................................................................................................................UUGCUGGGUGACGGGUGC............................x2
```

```
Sequence   1 Structure      1
 ENERGY =  -72.1    1
Bases    1 to  161
```

```
         10         20         30          40         50         60         70
.-CG  C    U A U           C      -  AU      C      G       C    C     A  AUAC
    GA GCAU C U CUCGUCACCCA CAACGA CC  GUUUU AUGGC UAUGCU GUGG GACG UG    G
    CU CGUA G G GGGCAGUGGGU GUUGCU GG  CAGGA UACCG AUACGA CACU CUGC AC    G
\ --  C    C C U           C      C  C-      U      G       C    U     C  CAUG
    140       130       120       110         100        90         80

         150
  ---     UG
     GUACU  C
     CAUGG  C
  CCU     CC
 160
```

____

Esi-MIR11354

```
1        10         20         30         40         50         60         70         80         90         100        110
GUUGUACAGUAGUCUCUCCGAUGUCCCAGCUUCCCGCCCAUCGACCACUGAACAGUUGUCGAUGGGCGGGAAGCUGGGACAUCGAAGGUACUUUCUCGUCGGUGGUGUGGU
.(((.((((.(((.(((.(((((((((((((((((((((((((((.(((....))).))))))))))))))))))))))))))).))).)))..)).))))).........
........GUAGUCUCUCCGAUGUCCCAGCU................................................................................x1
.........UAGUCUCUCCGAUGUCCCAGC.................................................................................x30*
.........UAGUCUCUCCGAUGUCCCAG..................................................................................x4
.........UAGUCUCUCCGAUGUCCCAGCU................................................................................x1
..............................UUCCCGCCCAUCGACCACUGAACAGU.......................................................x3
.........................................................................CUGGGACAUCGAAGGUACUUU.................x1
..........................................................................UGGGACAUCGAAGGUACUUUC................x110*
..........................................................................UGGGACAUCGAAGGUACUUUCU...............x21
..........................................................................UGGGACAUCGAAGGUACUUU.................x8
```

```
Sequence   1 Structure      1
```

```
 ENERGY =  -67.6    1
Bases    1 to  111

                10         20         30         40         50
G--------   U  -  U-   C   C                               C   G
         UUG AC AG  AGU UCU CGAUGUCCCAGCUUCCCGCCCAUCGAC ACU A
         GGC UG UC  UCA GGA GCUACAGGGUCGAAGGGCGGGUAGCUG UGA A
UGGUGUGGU   -  C  UU   U   A                               U   C
 .       100         90         80         70         60
```

Esi-MIR11355

```
1       10        20        30        40        50        60        70        80        90        100       110       120       130
ACACCCCCUGCAGCGUGGUCCCGAACAUUGGACAACAGUUGGUCUAUUAGGCGUAGAACGAUGUCUUUAGACUUAACUUUUGUCCAACGGUCGGGACCACGCCACAGGGGUGUUGAAUGGGGAAUGU
(((((((.((..((((((((((((.(.((((((((.((((((((((..((((((......)))))).))))).))))).)))))))).).))))))))))))..)))))))))..............
.............CGUGGUCCCGAACAUUGGACA.............................................................................................x2*
...............................................................................UUGUCCAACGGUCGGGACCAC...........................x1
................................................................................UGUCCAACGGUCGGGACCACG..........................x4
..................................................................................UCCAACGGUCGGGACCACGCC........................x525*
..................................................................................UCCAACGGUCGGGACCACGC.........................x340
..................................................................................UCCAACGGUCGGGACCACG..........................x32
..................................................................................UCCAACGGUCGGGACCAC...........................x13
..................................................................................UCCAACGGUCGGGACCACGCCA.......................x5
....................................................................................CAACGGUCGGGACCACGCC........................x2
.....................................................................................AACGGUCGGGACCACGCC........................x1
```

```
Sequence   1 Structure      1
 ENERGY =  -68.4    1
Bases    1 to  127

                     10        20        30        40         50
--------------      C  CA          A A        C     -     UU      AG
              ACACCCC UG  GCGUGGUCCCGA C UUGGACAA AGUUG GUCUA  AGGCGU  A
              UGUGGGG AC  CGCACCAGGGCU G AACCUGUU UCAAU CAGAU  UCUGUA  A
UGUAAGGGGUAAGU      -  AC          G C        U     U     U-      GC
     120       110        100        90        80        70         60
```

Esi-MIR11356

```
1       10        20        30        40        50        60        70        80        90        100       110       120       130       140       150       160       170
GAAGCCCGACAUGGCGUAGAGUUGGUUCAGGGUCGCCGCAUUCCCCGCGCACACCAUCCCGCGUCAACGACUGCGUCGUGGAGAUGCAUUGGUUGAUGCGGGAUGGGGUGCGGGGGAAUGCGGCGGUCCUGGGCCAACUGAACACCGUGUCGGGCUUCCGCGUGCG
((((((((((((((.((..(((((((((((((((((((((((((((((((.(.(((((((((((((((.(.((((((.....)))))).).))))))))))))))))))))))))))))))))))))))))))))))))..)).)))))))))))))))........
...........UGGCGUAGAGUUGGUUCAGG.......................................................................................................................................x1
................UAGAGUUGGUUCAGGGUCGCC.................................................................................................................................x1938*
................UAGAGUUGGUUCAGGGUCGC..................................................................................................................................x611
................UAGAGUUGGUUCAGGGUCG...................................................................................................................................x61
................UAGAGUUGGUUCAGGGUCGCCG................................................................................................................................x2
................UAGAGUUGGUUCAGGGUC....................................................................................................................................x1
.................AGAGUUGGUUCAGGGUCGCC.................................................................................................................................x3
.................AGAGUUGGUUCAGGGUCGC..................................................................................................................................x1
......................................................................................................AUGGGGUGCGGGGGAAUGCG............................................x1
...........................................................................................................................CGGUCCUGGGCCAACUGAACA......................x4*
...........................................................................................................................CGGUCCUGGGCCAACUGAAC.......................x1
................................................................................................................................................CCGUGUCGGGCUUCCGCGUGC.x1
```

```
Sequence   1 Structure      1
 ENERGY =  -138.5    1
Bases    1 to  167
```

```
                10         20         30         40         50         60         70         80
--------              C  AG                                 A A               G C      GU
        GAAGCCCGACAUGG GU  AGUUGGUUCAGGGUCGCCGCAUUCCCCCGCGC C CCAUCCCGCGUCAAC A UGCGUC  \
        CUUCGGGCUGUGCC CA  UCAACCGGGUCCUGGCGGCGUAAGGGGGCGUG G GGUAGGGCGUAGUUG U ACGUAG  G
GCGUGCGC              A  AG                                 - -               G U      AG
      160       150       140       130       120       110         100         90
 _____________________________________________________
```

Esi-MIR11357

```
1       10        20        30        40        50        60        70        80        90       100       110       120       130       140       150       160       170
CAUUUCGGGUGGGUUUUGUUCAUCUCGUUCUUCCUCGGCGAUGCCACCUUCCCAGCUGCUGUCAACAACGCCGGUCGUGAUCUUGCGCCUGGCGUUGUUGACUGCAGCUGGGAAGGAGUCAUCGCCGAGGAAGAAGGCGAUGAACAAAACCCACUCGAAAUGGG
((((((((((((((((((((((((.(.((((((((((((((((.(.(((((((((((((.((((((((((((((.(((......))).)))))))))))))).))))))))))))).).)))))))))))))))).).))))))))))))))))))))))))..
.................GUUCAUCUCGUUCUUCCUCGG..............................................................................................................................x2
..................UUCAUCUCGUUCUUCCUCGGC.............................................................................................................................x83
..................UUCAUCUCGUUCUUCCUCGG..............................................................................................................................x22
..................UUCAUCUCGUUCUUCCUCG...............................................................................................................................x1
...................UCAUCUCGUUCUUCCUCGGCG............................................................................................................................x193*
...................UCAUCUCGUUCUUCCUCGGC.............................................................................................................................x177
...................UCAUCUCGUUCUUCCUCGG..............................................................................................................................x26
...................UCAUCUCGUUCUUCCUCGGCGA...........................................................................................................................x24
...................UCAUCUCGUUCUUCCUCG...............................................................................................................................x1
...................UCAUCUCGUUCUUCCUCGGCGAU..........................................................................................................................x1
....................CAUCUCGUUCUUCCUCGGCG............................................................................................................................x1
.............................................................UCAACAACGCCGGUCGUGAUCUUGC..............................................................................x1
............................................................................................................................CCGAGGAAGAAGGCGAUGAAC...................x11*
............................................................................................................................CCGAGGAAGAAGGCGAUGAACAA.................x3
............................................................................................................................CCGAGGAAGAAGGCGAUGAA....................x2
............................................................................................................................CCGAGGAAGAAGGCGAUGAACA..................x1
.............................................................................................................................CGAGGAAGAAGGCGAUGAACA..................x2
.............................................................................................................................CGAGGAAGAAGGCGAUGAAC...................x1
..............................................................................................................................GAGGAAGAAGGCGAUGAACAA.................x7
..............................................................................................................................GAGGAAGAAGGCGAUGAACA..................x6
..............................................................................................................................GAGGAAGAAGGCGAUGAACAAA................x3

Sequence   1 Structure      1
 ENERGY =  -134.7    1
Bases    1 to  164

         10        20        30        40        50        60        70        80
--                        U G              C A           U              U   GA
  CAUUUCGGGUGGGUUUUGUUCAUC C UUCUUCCUCGGCGAUG C CCUUCCCAGCUGC GUCAACAACGCCGG CGU  U
  GUAAAGCUCACCCAAAACAAGUAG G AAGAAGGAGCCGCUAC G GGAAGGGUCGACG CAGUUGUUGCGGUC GCG  C
GG                        C G              U A           U              C   UU
  160       150       140       130       120       110       100        90
 _____________________________________________________
```

Esi-MIR11358

```
1       10        20        30        40        50        60        70        80        90       100       110       120       130       140       150
AUUUGUCUCCAACCUCCCCGAGAUGGUGAUCCUGUUCGGACGAUGGCAGGCCUCAGCCACCUGUGGUAUACCCACCUGUCACAUGUGGCUGAGGUCUGCCAUCAGAACCAACAGAAUCAGCGCCCCGGUGAGGGUGGGGACAAGA
.((((((((((.((((.(((.(.((.((((.(((((.((..(((((((((((((((((((.(((((((........))))))).))))))))))))))))))).....))))))).)))).)).).))).)))).)))))))))).
.....UCUCCAACCUCCCCGAGAUGG.......................................................................................................................x3
.......UCCAACCUCCCCGAGAUGGUGA....................................................................................................................x1
.............................UCCUGUUCGGACGAUGGCAGG...............................................................................................x39*
.............................UCCUGUUCGGACGAUGGCAG................................................................................................x10
.............................UCCUGUUCGGACGAUGGCAGGC..............................................................................................x7
.............................UCCUGUUCGGACGAUGGCA.................................................................................................x1
.............................UCCUGUUCGGACGAUGGC..................................................................................................x1
```

```
...............................CUGUUCGGACGAUGGCAGG...............................................................................................x1
...............................CUGUUCGGACGAUGGCAGGCC.............................................................................................x1
...............................CUGUUCGGACGAUGGCAGGCCU............................................................................................x1
..................................UUCGGACGAUGGCAGGCCUCA..........................................................................................x10
..................................UUCGGACGAUGGCAGGCC.............................................................................................x1
..............................................................................................UCUGCCAUCAGAACCAACAGA..............................x4
..............................................................................................UCUGCCAUCAGAACCAACAGAA.............................x3
..............................................................................................UCUGCCAUCAGAACCAACAGAAUCAG.........................x2
..............................................................................................UCUGCCAUCAGAACCAACAGAAU............................x2
..............................................................................................UCUGCCAUCAGAACCAACAGAAUC...........................x1
................................................................................................UGCCAUCAGAACCAACAGAAUC...........................x83*
................................................................................................UGCCAUCAGAACCAACAGAAUCA..........................x1
................................................................................................UGCCAUCAGAACCAACAGA..............................x1
.................................................................................................GCCAUCAGAACCAACAGAAUC...........................x1
.....................................................................................................UCAGAACCAACAGAAUCAGCGC......................x6
......................................................................................................................AGCGCCCCGGUGAGGGUGGGGA.....x1

Sequence   1 Structure      1
 ENERGY =  -85.5    1
Bases    1 to  145

        10        20        30        40          50        60        70
A          A    C   A A  G    C     C  AC--                   C        UAC
 UUUGUCUCCA CCUC CCG G UG UGAU CUGUU GG    GAUGGCAGGCCUCAGCCAC UGUGGUA   C
 GAACAGGGGU GGAG GGC C GC ACUA GACAA CC    CUACCGUCUGGAGUCGGUG ACACUGU   C
A          G    U   C C  G    A     -  AAGA                   U        CCA
   140       130       120       110        100        90        80
 ________________________________________________
```

Esi-MIR11359

```
1       10        20        30        40        50        60        70        80        90        100
ACGCCUCGUUCACUCCAAGCGCACAGGAACCAUGAUCGUCUCUGCGCAGAGAUGAUGAUCGUUGUUGUGGGCUUGGAGCGAACGAAGCGUGACGUUUGGA
((((.((((((.((((((((.(((((.(((.((.(((((((((....))))))))).)).))).))))).)))))))).)))))).))))..........
...CCUCGUUCACUCCAAGCGCACA............................................................................x6
...CCUCGUUCACUCCAAGCGCA..............................................................................x2
....CUCGUUCACUCCAAGCGCACA............................................................................x42*
....CUCGUUCACUCCAAGCGCAC.............................................................................x2
....CUCGUUCACUCCAAGCGCACAG...........................................................................x1
.....UCGUUCACUCCAAGCGCACA............................................................................x5
...................................................................UGGGCUUGGAGCGAACGAAGC............x5245*
...................................................................UGGGCUUGGAGCGAACGAAG.............x173
...................................................................UGGGCUUGGAGCGAACGAAGCG...........x122
...................................................................UGGGCUUGGAGCGAACGAA..............x80
...................................................................UGGGCUUGGAGCGAACGA...............x6
...................................................................UGGGCUUGGAGCGAACGAAGCGU..........x1
....................................................................GGGCUUGGAGCGAACGAAGC............x33
....................................................................GGGCUUGGAGCGAACGAAGCG...........x1
.....................................................................GGCUUGGAGCGAACGAAGC............x1
......................................................................GCUUGGAGCGAACGAAGC............x4

Sequence   1 Structure      1
0 ENERGY =  -57.7    1
Bases    1 to  100

                  10        20        30        40
----------    C      A        G     G   C  G         G
          ACGC UCGUUC CUCCAAGC CACAG AAC AU AUCGUCUCU C
          UGCG AGCAAG GAGGUUCG GUGUU UUG UA UAGUAGAGA G
AGGUUUGCAG    A      C        G     G   C  G         C
.        90        80        70        60        50
____________________________________________
```

Esi-MIR11360

```
1       10        20        30        40        50        60        70        80        90        100
ACUAGCUCGCGUCUGCGCGGUGAAUGCCGAAGAAGGGAUUAGAAUCAAGUCCAACUCCUUCGUCGGCAUUAAUCGAGCAGACGUUGUGCUAGUGGAGAUGGA
((((((.(((((((((.((((.((((((((.((((((.((.((......)).)).)))))).)))))))).)))).))))))).)).))))))).........
.....CUCGCGUCUGCGCGGUGAAUG............................................................................x1*
......UCGCGUCUGCGCGGUGAAUGC...........................................................................x1
............................................UCAAGUCCAACUCCUUCGUCGGC...................................x1
.................................................UCCAACUCCUUCGUCGGCA..................................x2
....................................................................UUAAUCGAGCAGACGUUGUGCU............x2093*
....................................................................UUAAUCGAGCAGACGUUGUG..............x287
....................................................................UUAAUCGAGCAGACGUUGUGC.............x266
....................................................................UUAAUCGAGCAGACGUUGUGCUA...........x43
....................................................................UUAAUCGAGCAGACGUUGU...............x16
....................................................................UUAAUCGAGCAGACGUUG.................x5
.....................................................................UAAUCGAGCAGACGUUGUGCUA...........x79
.....................................................................UAAUCGAGCAGACGUUGUGCU............x55
.....................................................................UAAUCGAGCAGACGUUGUGC.............x1
......................................................................AAUCGAGCAGACGUUGUGCU............x1
......................................................................AAUCGAGCAGACGUUGUG..............x1
Sequence   1 Structure      1
 ENERGY =  -49.4    1
Bases    1 to  102

                  10        20        30        40
---------     U  -       G    G        A      A  A  AU
         ACUAGC CG CGUCUGC CGGU AAUGCCGA GAAGGG UU GA  C
         UGAUCG GU GCAGACG GCUA UUACGGCU CUUCCU AA CU  A
AGGUAGAGG     U  U       A    A        G      C  C  GA
100        90        80        70        60        50
```

Esi-MIR11361

```
1       10        20        30        40        50        60        70        80        90        100       110
GCCCGCGUUGCUUCUUGACGAGGAGGAUGUCAGCUUCUGCCGUUUUGCAGUUGUUUUGAAACUGAAAGACGGCAGGCGCGGACCUCUUCCUCGUCAAGAACAACG
.....(((((.((((((((((((((((.(((.((.(((((((((((.(((((.......))))).))))))))))).)).))).))))))))))))))))))))))
..............UUGACGAGGAGGAUGUCAGCU......................................................................x2943*
..............UUGACGAGGAGGAUGUCAGC.......................................................................x98
..............UUGACGAGGAGGAUGUCAG........................................................................x17
..............UUGACGAGGAGGAUGUCAGCUU.....................................................................x12
..............UUGACGAGGAGGAUGUCA.........................................................................x5
...............UGACGAGGAGGAUGUCAGCU......................................................................x11
...............UGACGAGGAGGAUGUCAG........................................................................x1
................GACGAGGAGGAUGUCAGCU......................................................................x7
.................ACGAGGAGGAUGUCAGCU......................................................................x2
.....................................................UUUUGAAACUGAAAGACGGCAGGCG...........................x1
......................................................UUUGAAACUGAAAGACGGCAGGC............................x1
........................................................UGAAACUGAAAGACGGCAG..............................x1
..............................................................................CGGACCUCUUCCUCGUCAAGA......x236*
..............................................................................CGGACCUCUUCCUCGUCAAG.......x18
..............................................................................CGGACCUCUUCCUCGUCAAGAA.....x3
..............................................................................CGGACCUCUUCCUCGUCAA........x2
..............................................................................CGGACCUCUUCCUCGUCA.........x1
...............................................................................GGACCUCUUCCUCGUCAAGA......x2

Sequence   1 Structure      1
 ENERGY =  -63.4    1
Bases    1 to  105

        10        20        30        40        50
GCCCG     C                U   A  U           G     GUU
     CGUUG UUCUUGACGAGGAGGA GUC GC UCUGCCGUUUU CAGUU   \
     GCAAC AAGAACUGCUCCUUCU CAG CG GGACGGCAGAA GUCAA   U
-----     -                C   G  C           A     AGU
```

```
         100        90         80         70         60
 _________________________________________________

Esi-MIR11362

1        10        20        30        40        50        60        70        80        90       100
AAGAAGCUUGAGAUGCCCCAUGAGCUACGGAUGGCGGUGGUGCGUGACGCACCUCGGCCACCCGCAGUUCGUGGUGCAUAUCGAGCUUCUUGAGCUUG
(((((((((((.((((.(((((((((.(((.((((.(.((((((...)))))).).)))).))).))))))))).)))).))))))))))).......
....AGCUUGAGAUGCCCCAUGAGC.........................................................................x3*
....................................................................UCGUGGUGCAUAUCGAGCUUC.........x22*
....................................................................UCGUGGUGCAUAUCGAGCUUCU........x1
....................................................................UCGUGGUGCAUAUCGAGCUU..........x1

Sequence   1 Structure       1
 ENERGY =  -60.8     1
Bases     1 to   98

               10        20        30        40
-------           G    C         A   A    G U      U
       AAGAAGCUUGA AUGC CCAUGAGCU CGG UGGC G GGUGCG \
       UUCUUCGAGCU UACG GGUGCUUGA GCC ACCG C CCACGC G
GUUCGAG           A    U         C   C    G U      A
       90        80        70        60        50
 _________________________________________________

Esi-MIR11363

1        10        20        30        40        50        60        70        80        90       100
CCAGCUCCCCUAGCAAUGGUCUGCAUCGACAUGGCGAGUGAAACAAAGGUCACGCCCUUUGUUUCACACACCAUGUCGACGCAGACCACCGCUACGGGGGG
....((((((((((..((((((((.(((((((((.(.((((((((((((......)))))))))))).).))))))))).))))))))..)))).))))))
..........UAGCAAUGGUCUGCAUCGACA......................................................................x319*
..........UAGCAAUGGUCUGCAUCGA........................................................................x1
............GCAAUGGUCUGCAUCGACA......................................................................x2
...........................................................................UCGACGCAGACCACCGCUACGG....x31*
...........................................................................UCGACGCAGACCACCGCUACG.....x7
...........................................................................UCGACGCAGACCACCGCUAC......x1
.............................................................................GACGCAGACCACCGCUACGG....x1


Sequence   1 Structure       1
 ENERGY =  -66.0     1
Bases     1 to  101

        10         20        30        40        50
CCAG      -    AA        A         C A            UC
    CUCCCC UAGC  UGGUCUGC UCGACAUGG G GUGAAACAAAGG  A
    GGGGGG AUCG  ACCAGACG AGCUGUACC C CACUUUGUUUCC  C
----      C    CC        C         A A            CG
   100        90        80        70        60
 _________________________________________________

Esi-MIR11364

1        10        20        30        40        50        60        70        80        90       100
AUGUCCUGGGCUGGCCGAGAGAUCCCGGCGACCGCAUCUCGGGAAUGCCUUCCCUAGAUGCGAGCGGCGGGUUUCGUCAGCCAACCCAGGACAGGCC
.(((((((((.((((.((((((.((((.((..(((((((.(((((....))))).)))))))..)).)))))))).)).)))).)))))))))....
...UCCUGGGCUGGCCGAGAGAUCCCG......................................................................x3
......UGGGCUGGCCGAGAGAUCCCG......................................................................x42*
......UGGGCUGGCCGAGAGAUC.........................................................................x5
......UGGGCUGGCCGAGAGAUCCC.......................................................................x4
......UGGGCUGGCCGAGAGAUCC........................................................................x4
.......GGGCUGGCCGAGAGAUCCCG......................................................................x1
.....................................................................GGUUUCGUCAGCCAACCCAGG.......x3*
.....................................................................GGUUUCGUCAGCCAACCCAGGA......x1
```

```
Sequence   1 Structure      1
 ENERGY =  -62.7    1
Bases    1 to   97

           10        20        30        40
A---         C    C  -    U    G  AC       C     U
    UGUCCUGGG UGGC GA GAGA CCCG CG  CGCAUCU GGGAA G
    ACAGGACCC ACCG CU CUUU GGGC GC  GCGUAGA CCCUU C
CCGG         A    A  G    -    G  GA       U     C
      90        80         70        60        50
```

Esi-MIR11365

```
1       10        20        30        40        50        60        70        80        90       100
GGAUGGGAGACGGUGUCGACCAUUGUUUUGCGGUAGAUGUACCGUGGAGUGCAUUCGCCACGAACCGAUGGUCGACACCGUCUCCUAUCCUGUGUG
(((((((((((((((((((((((((.((((.(((.(((((((......))))))).))).)))).)))))))))))))))))))))))))......
...UGGGAGACGGUGUCGACCAUU........................................................................x2*
....................................................................UGGUCGACACCGUCUCCUAUC.......x95*
....................................................................UGGUCGACACCGUCUCCUAU........x12
....................................................................UGGUCGACACCGUCUCCUA.........x4
.....................................................................GGUCGACACCGUCUCCUAU........x1
.....................................................................GGUCGACACCGUCUCCUAUC.......x1
```

```
Sequence   1 Structure      1
 ENERGY =  -66.2    1
Bases    1 to   96

              10        20        30        40
------                         U    C   A       CG
      GGAUGGGAGACGGUGUCGACCAUUG UUUG GGU GAUGUAC  U
      CCUAUCCUCUGCCACAGCUGGUAGC AAGC CCG UUACGUG  G
GUGUGU                         C    A   C       AG
     90        80        70        60        50
```

Esi-MIR11366

```
1       10        20        30        40        50        60        70        80        90       100
CAAGAAGGUCGGGGGCACCUUCAACGAGAACAGGGAAAGGUAGCGUGUAGUACCUUUCCCUUUUUCUCGUUGAAUGUUCCGCCGACCUUCUGGUUUA
..((((((((((.((.((.(((((((((((.(((((((((((........)))))))))))..))))))))))).)).)).))))))))))......
....AAGGUCGGGGGCACCUUCAAC........................................................................x1*
.....................................................................UUGAAUGUUCCGCCGACCUUC.......x8
.....................................................................UUGAAUGUUCCGCCGACCUU........x2
.....................................................................UUGAAUGUUCCGCCGACCU.........x1
......................................................................UGAAUGUUCCGCCGACCUUCU......x574*
......................................................................UGAAUGUUCCGCCGACCUUC.......x80
......................................................................UGAAUGUUCCGCCGACCUU........x16
```

```
Sequence   1 Structure      1
 ENERGY =  -59.1    1
Bases    1 to   97

            10        20        30         40
CA----          G  C  C           C-           GCG
      AGAAGGUCGG GG AC UUCAACGAGAA  AGGGAAAGGUA   U
      UCUUCCAGCC CC UG AAGUUGCUCUU  UCCCUUUCCAU   G
AUUUGG          G  U  U           UU           GAU
      90        80        70        60        50
```

Esi-MIR11367

```
1       10        20        30        40        50        60        70        80        90        100
CGACCUGGCUCGAACCCUCCGAGGUUCAAGCGUUGGAGGCCACUCGCACGCACGCCCGAAUGGCCUCCAACGGUGGAUUCUCGGAGGAUUCGAACCAGGUC
.(((((((.(((((.(((((((((.((.(.((((((((((((.(((..(....)..))).)))))))))))).).)).))))))))).))))).)))))))
.........UCGAACCCUCCGAGGUUCAAGC......................................................................x2*
.........UCGAACCCUCCGAGGUUCAAG.......................................................................x2
..............CCCUCCGAGGUUCAAGCGUUG..................................................................x1
.........................................................................UGGAUUCUCGGAGGAUUCGAAC......x34*
.........................................................................UGGAUUCUCGGAGGAUUCGAACC.....x3
.........................................................................UGGAUUCUCGGAGGAUUCGAA.......x2
..........................................................................GGAUUCUCGGAGGAUUCGAAC......x2

Sequence   1 Structure      1
 ENERGY =  -67.6    1
Bases    1 to  101

        10        20        30        40        50
C       C     C         U  A G            C   CA G
 GACCUGG UCGAA CCUCCGAGG UC A CGUUGGAGGCCA UCG  C C
 CUGGACC AGCUU GGAGGCUCU AG U GCAACCUCCGGU AGC  G A
-       A     A         U  G G            A   CC C
100        90        80        70        60
 __________________________________________________
```

Esi-MIR11368

```
1       10        20        30        40        50        60        70        80        90        100
GUCCGUCUGUGCUGACCCCAAGUUCAAGGAUGUGCCCUUUUGGGCGGGACCGCUCAAAAGCGCACAUCCUUGAACUAGGGGUCAUCGCAGACGGACGGAUCA
(((((((((((.(((((((.(((((((((((((((.((((((((((....)))))))))).))))))))))))))).))))))).)))))))))))......
.....UCUGUGCUGACCCCAAGUUCA............................................................................x2
.......UGUGCUGACCCCAAGUUCAAG..........................................................................x83*
.......UGUGCUGACCCCAAGUUCAA...........................................................................x8
.......UGUGCUGACCCCAAGUUCAAGG.........................................................................x1
...................................................................CCUUGAACUAGGGGUCAUCGCAGA...........x1
.....................................................................UUGAACUAGGGGUCAUCGCAG............x33
.....................................................................UUGAACUAGGGGUCAUCGCA.............x7
.....................................................................UUGAACUAGGGGUCAUCGCAGA...........x1
......................................................................UGAACUAGGGGUCAUCGCAGA...........x41962*
......................................................................UGAACUAGGGGUCAUCGCAG............x617
......................................................................UGAACUAGGGGUCAUCGCA.............x94
......................................................................UGAACUAGGGGUCAUCGCAGAC..........x91
......................................................................UGAACUAGGGGUCAUCGC..............x9
......................................................................UGAACUAGGGGUCAUCGCAGACG.........x1
.......................................................................GAACUAGGGGUCAUCGCAGA...........x76
.......................................................................GAACUAGGGGUCAUCGCA.............x1
........................................................................AACUAGGGGUCAUCGCAGA...........x10
........................................................................AACUAGGGGUCAUCGCAG............x1
.........................................................................ACUAGGGGUCAUCGCAGA...........x2

Sequence   1 Structure      1
 ENERGY =  -79.5    1
Bases    1 to  102

              10        20        30        40
------           C       A               C          G
      GUCCGUCUGUG UGACCCC AGUUCAAGGAUGUGC CUUUUGGGCG G
      CAGGCAGACGC ACUGGGG UCAAGUUCCUACACG GAAAACUCGC A
ACUAGG           U       A               C          C
100        90        80        70        60        50
 __________________________________________________
```

Esi-MIR11369

```
1       10        20        30        40        50        60        70        80        90        100
```

```
GGCGGGGCGUGGACGUUGGGCACCCACUCCUUCGACAAACCGAUUCAAGCUUGUCGAACGGUUGGGUGCCCAGCGUCCACGCCCCGCCAUUGAAGGUGCUG
((((((((((((((((((((((((((..((((((((((.(........).)))))))).)).)))))))))))))))))))))))))).............
..CGGGGCGUGGACGUUGGGCACC.............................................................................x19
..CGGGGCGUGGACGUUGGG.................................................................................x2
..CGGGGCGUGGACGUUGGGCAC..............................................................................x1
...GGGGCGUGGACGUUGGGCACC.............................................................................x112*
...GGGGCGUGGACGUUGGGCAC..............................................................................x16
...GGGGCGUGGACGUUGGGCA...............................................................................x14
...GGGGCGUGGACGUUGGGC................................................................................x3
....GGGCGUGGACGUUGGGCACC.............................................................................x3
.....GGCGUGGACGUUGGGCACC.............................................................................x2
........................CACUCCUUCGACAAACCGAUU........................................................x2
.......................................CCGAUUCAAGCUUGUCGA............................................x1
...........................................UUCAAGCUUGUCGAACGGUUGG....................................x2
...........................................UUCAAGCUUGUCGAACGGUUGGG...................................x1
............................................UCAAGCUUGUCGAACGGUUGGG...................................x27
............................................UCAAGCUUGUCGAACGGUUGG....................................x19
............................................UCAAGCUUGUCGAACGGUUG.....................................x6
............................................UCAAGCUUGUCGAACGGUU......................................x3
..................................................................UGCCCAGCGUCCACGCCCCGC..............x150*
..................................................................UGCCCAGCGUCCACGCCCCGCC.............x26
..................................................................UGCCCAGCGUCCACGCCCCG...............x6
..................................................................UGCCCAGCGUCCACGCCCCGCCA............x3
..................................................................UGCCCAGCGUCCACGCCCC................x3
..................................................................UGCCCAGCGUCCACGCCC.................x1
...................................................................GCCCAGCGUCCACGCCCCGC..............x1
...................................................................GCCCAGCGUCCACGCCCCGCC.............x1
```

```
Sequence   1 Structure      1
 ENERGY =  -75.7    1
Bases    1 to  101
```

```
                     10        20        30         40
-------------                          CU  -        A CGA
             GGCGGGGCGUGGACGUUGGGCACCCA  CC UUCGACAA C   U
             CCGCCCCGCACCUGCGACCCGUGGGU  GG AAGCUGUU G   U
GUCGUGGAAGUUA                          U-  C        C AAC
 .        90        80        70         60        50
```

Esi-MIR11370

```
1       10        20        30        40        50        60        70        80        90        100
GUUGGGUCAGUCGCCAAGUUCAUGGGCAGGGUCGCUACCCCAACUCGAUCGCGGUGGGCGGCGACGCCUGCCCAUGUACUGAGGUGACUGACCCAA
.((((((((((((((.(((.((((((((((((((((.(((..((.((....))))))).)))))).)))))))))).)))..))))))))))))))
......UCAGUCGCCAAGUUCAUGGG......................................................................x103*
......UCAGUCGCCAAGUUCAUGG.......................................................................x6
......UCAGUCGCCAAGUUCAUGGGC.....................................................................x3
......UCAGUCGCCAAGUUCAUG........................................................................x1
.......CAGUCGCCAAGUUCAUGGG......................................................................x1
.......................................................................CCAUGUACUGAGGUGACUGACC...x1
........................................................................CAUGUACUGAGGUGACUGACC...x1*
```

```
Sequence   1 Structure      1
 ENERGY =  -68.0    1
Bases    1 to   96
```

```
        10         20        30         40
G              A-   U          -      A   CA  U  A
 UUGGGUCAGUCGCC  AGU CAUGGGCAGG GUCGCU CCC  AC CG U
 AACCCAGUCAGUGG  UCA GUACCCGUCC CAGCGG GGG  UG GC C
-              AG   U          G      C   --  -  G
      90        80        70        60           50
```

Esi-MIR11371

```
1       10        20        30        40        50        60        70        80        90        100
CACAAGCUAUGGAACAAGAUAUAGACGUCAAGCGGGGGACGGCAGACUCCCGUUCCCCGCGCUUGAUGUCUAUAUCUUGGUCCGUAGUGUGUACGUAUGACG
.(((.((((((((.((((((((((((((((((((((((((((.......))).))))).)))))))))))))))))))).)))))))).)))..........
...AAGCUAUGGAACAAGAUAUAGA.............................................................................x1
....AGCUAUGGAACAAGAUAUAGA.............................................................................x8*
....AGCUAUGGAACAAGAUAUAG..............................................................................x1
......................................................................UAUAUCUUGGUCCGUAGUGUG...........x4542*
......................................................................UAUAUCUUGGUCCGUAGUGU............x264
......................................................................UAUAUCUUGGUCCGUAGUGUGU..........x72
......................................................................UAUAUCUUGGUCCGUAGUG.............x6
......................................................................UAUAUCUUGGUCCGUAGU..............x4
......................................................................UAUAUCUUGGUCCGUAGUGUGUA.........x1
.......................................................................AUAUCUUGGUCCGUAGUGUG...........x52
.......................................................................AUAUCUUGGUCCGUAGUGUGU..........x5
.......................................................................AUAUCUUGGUCCGUAGUGU............x4
........................................................................UAUCUUGGUCCGUAGUGUG...........x9
........................................................................UAUCUUGGUCCGUAGUGUGU..........x3
........................................................................UAUCUUGGUCCGUAGUGU............x2
........................................................................UAUCUUGGUCCGUAGUGUGUA.........x1

Sequence   1 Structure      1
 ENERGY =  -61.4    1
Bases    1 to  102

                 10        20        30          40
C---------   A        A                    -     -   CAG
          ACA GCUAUGGA CAAGAUAUAGACGUCAAGCG GGGGA CGG   \
          UGU UGAUGCCU GUUCUAUAUCUGUAGUUCGC CCCCU GCC   A
GCAGUAUGCA   G        G                    G     U   CUC
100        90        80        70        60        50
```

Esi-MIR11372

```
1       10        20        30        40        50        60        70        80        90        100
UUCUCCAGUUGGCUCUCCAACAACCCCGCCUCUGCCUCGCGUUCGCCAAUUUUGUGUCGAAAUCGAGGCAGAGGCGUGGUUGUUGGAGAACCAAGUGG
....(((.((((.((((((((((((.(((((((((((((..((((.((......)).))))..))))))))))))).)))))))))))).)))).)))

........UUGGCUCUCCAACAACCCCGC.....................................................................x2
........UUGGCUCUCCAACAACCCCGCC....................................................................x1
.........UGGCUCUCCAACAACCCCGCC....................................................................x6884*
.........UGGCUCUCCAACAACCCCGC.....................................................................x2158
.........UGGCUCUCCAACAACCCCG......................................................................x132
.........UGGCUCUCCAACAACCCCGCCU...................................................................x29
.........UGGCUCUCCAACAACCCC.......................................................................x10
..........GGCUCUCCAACAACCCCGCC....................................................................x59
..........GGCUCUCCAACAACCCCGC.....................................................................x15
..........GGCUCUCCAACAACCCCGCCU...................................................................x3
...........GCUCUCCAACAACCCCGCC....................................................................x1
.............UCUCCAACAACCCCGCCUCUG................................................................x4
.............UCUCCAACAACCCCGCCU...................................................................x1
..............................UCUGCCUCGCGUUCGCCAAUU...............................................x7
..............................UCUGCCUCGCGUUCGCCAAUUU..............................................x5
..............................UCUGCCUCGCGUUCGCCAAU................................................x3
..............................UCUGCCUCGCGUUCGCCAAUUUU.............................................x2
.....................................................GUGUCGAAAUCGAGGCAGAGG........................x1
.......................................................GUCGAAAUCGAGGCAGAGG........................x1
.....................................................................AGAGGCGUGGUUGUUGGAGA.........x1
.........................................................................GCGUGGUUGUUGGAGAACCAA....x2
..........................................................................CGUGGUUGUUGGAGAACCAAG...x24*
..........................................................................CGUGGUUGUUGGAGAACC......x4
```

```
..........................................................................CGUGGUUGUUGGAGAACCAA....x4
............................................................................UGGUUGUUGGAGAACCAAGUGGx16
............................................................................UGGUUGUUGGAGAACCAAGUG.x1
.............................................................................GGUUGUUGGAGAACCAAGUGGx1

Sequence   1 Structure      1
 ENERGY =  -66.1    1
Bases    1 to   98

        10        20        30        40        50
UUCU   G    C            C             CG    C  AU
    CCA UUGG UCUCCAACAACC CGCCUCUGCCUCG  UUCG CA  U
    GGU AACC AGAGGUUGUUGG GCGGAGACGGAGC  AAGC GU  U
----   G    A            U             UA    U  GU
           90        80        70        60
 __________________________________________________

Esi-MIR11373

1       10        20        30        40        50        60        70        80        90        100
CACUCUUUGUCCUGUUCUUCUCCGCCAGGAGAACCGCGUGGGGUUGAUUCAACCCCAAGCGGCGCUCCUGGCGGGAAAUUCCAGACAAAGAGUGG
(((((((((((.((...(((.((((((((((..((((.((((((((...)))))))).))))..)))))))))))))....))))))))))))).
.....UUUGUCCUGUUCUUCUCCGCCA....................................................................x204*
.....UUUGUCCUGUUCUUCUCCGC......................................................................x16
.....UUUGUCCUGUUCUUCUCCGCC.....................................................................x15
.....UUUGUCCUGUUCUUCUCCG.......................................................................x1
.....UUUGUCCUGUUCUUCUCC........................................................................x1
......UUGUCCUGUUCUUCUCCGCCAG...................................................................x1
......UUGUCCUGUUCUUCUCCGCC.....................................................................x1
......................................................................GCGGGAAAUUCCAGACAAAGA....x5*

Sequence   1 Structure      1
 ENERGY =  -65.1    1
Bases    1 to   95

         10         20        30        40
-           C  UUC-   U          AA    G        A
 CACUCUUUGUC UG    UUC CCGCCAGGAG  CCGC UGGGGUUG \
 GUGAGAAACAG AC    AAG GGCGGUCCUC  GGCG ACCCCAAC U
G           -  CUUA   -          GC    A        U
    90         80         70        60        50
__________________________________________________

Esi-MIR11374

1       10        20        30        40        50        60        70        80        90        100
UCCUUUUUCGUCUUUCCCUGUUCUACUCCUCUGUCGCCACAAAAUAAAUGUCUUGUAGUGACGAAGGGGUAGGACAAGCAGACGAAGAAGGAUGGAU
(((((((((((((...(.((((((((((((.((((((.((((.((....)).)))).)))))).))))))))))))).).))))))))))))).....
...UUUUUCGUCUUUCCCUGUUCUAC.......................................................................x3*
......................................................................AGGACAAGCAGACGAAGAAGG......x9*
......................................................................AGGACAAGCAGACGAAGAAG.......x1

Sequence   1 Structure      1
 ENERGY =  -46.3    1
Bases    1 to   97

             10        20        30        40
-----             UUC C            C      C    A  A
     UCCUUUUUCGUCU   C UGUUCUACUCCU UGUCGC ACAA AU A
     AGGAAGAAGCAGA   G ACAGGAUGGGGA GCAGUG UGUU UG A
UAGGU             C-- A            A      A    C  U
      90        80          70        60        50
__________________________________________________
```

Esi-MIR11375

```
1       10        20        30        40        50        60        70        80        90        100
GGCAAGACCGUCGUUGUGUCAGCUGAUAACAUACACGCACAUCAAAAAGUAUGCAUGUGCAUGUAUGUUAUCAGCAGACACAACGACGGACUUGGCCGUGUAU
((((((.(((((((((((((.((((((((((((((.((((((((.......)).)))))).)))))))))))))).))))))))))))).))).)))......
.....GACCGUCGUUGUGUCAGCUGA.............................................................................x1
.......CCGUCGUUGUGUCAGCUGAUA...........................................................................x1*
...................................................................UUAUCAGCAGACACAACGACG...............x4
...................................................................UUAUCAGCAGACACAACGAC................x2
......................................................................UCAGCAGACACAACGACGGAC............x14626*
......................................................................UCAGCAGACACAACGACGGA.............x969
......................................................................UCAGCAGACACAACGACGGACU...........x524
......................................................................UCAGCAGACACAACGACGG..............x14
......................................................................UCAGCAGACACAACGACG...............x4
......................................................................UCAGCAGACACAACGACGGACUU..........x3
.......................................................................CAGCAGACACAACGACGGAC............x30
.......................................................................CAGCAGACACAACGACGGACU...........x17
.......................................................................CAGCAGACACAACGACGGA.............x3
........................................................................AGCAGACACAACGACGGAC............x59
........................................................................AGCAGACACAACGACGGACU...........x4
........................................................................AGCAGACACAACGACGGA.............x1
........................................................................AGCAGACACAACGACGGACUU..........x1
.........................................................................GCAGACACAACGACGGAC............x3

Sequence   1 Structure      1
 ENERGY =  -63.4    1
Bases    1 to  103

               10        20        30        40
------   -   A             A              C      -  AAA
      GGC AAG CCGUCGUUGUGUC GCUGAUAACAUACA GCACAU CA   \
      CCG UUC GGCAGCAACACAG CGACUAUUGUAUGU CGUGUA GU   A
UAUGUG   G   A             A              A      C  AUG
 100       90        80        70        60        50

__________________________________________________
```

Esi-MIR11376

```
1       10        20        30        40        50        60        70        80        90        100
GAUCGUCGUGUCCUGCAGUUGCUUCCCCGUAGUGGUUGGCGAAAGUAUUACUUCGUAAGCCAUGACGGGACGGCAGUUGGAGGACACGUACGAUCC
((((((((((((((.((((((((..(((((.((((((.(((((........))))).)))))).)))))..)))))))).)))))))).)))))).
.....UCGUGUCCUGCAGUUGCUUCC......................................................................x34*
.....UCGUGUCCUGCAGUUGCUUC.......................................................................x9
.....UCGUGUCCUGCAGUUGCUU........................................................................x1
.......GUGUCCUGCAGUUGCUUCC......................................................................x1
......................................................................CGGCAGUUGGAGGACACGUACG....x6*

Sequence   1 Structure      1
 ENERGY =  -55.9    1
Bases    1 to   96

          10        20        30        40
-      -        G        UC      A      G     AGU
 GAUCGU CGUGUCCU CAGUUGCU  CCCGU GUGGUU GCGAA   A
 CUAGCA GCACAGGA GUUGACGG  GGGCA UACCGA UGCUU   U
C      U        G        CA      G      A     CAU
     90        80        70        60        50
__________________________________________________
```

Esi-MIR11377

```
1       10        20        30        40        50        60        70        80        90        100
```

```
UGAGAAGAGAGCGCCCGCGACAACCUGUGGGCCUAUGACCUGCAAGCUUGCACGCCGCAGGGCAACAGGUCGUCCCGGGCGCUCUCUUCUGUGCAGAGAAU
..(((((((((((((((.(((.((((((.(.(((.((.(.((((....)))).).)).))).).)))))).))).)))))))))))))))...........
....AAGAGAGCGCCCGCGACAACC............................................................................x5*
....AAGAGAGCGCCCGCGACAACCU...........................................................................x1
.....AGAGAGCGCCCGCGACAACC............................................................................x1
.....................................................................UCGUCCCGGGCGCUCUCUUCU...........x8922*
.....................................................................UCGUCCCGGGCGCUCUCUUC............x1581
.....................................................................UCGUCCCGGGCGCUCUCUU.............x281
.....................................................................UCGUCCCGGGCGCUCUCUUCUG..........x135
.....................................................................UCGUCCCGGGCGCUCUCU..............x39
.....................................................................UCGUCCCGGGCGCUCUCUUCUGU.........x17
......................................................................CGUCCCGGGCGCUCUCUUCU...........x19
......................................................................CGUCCCGGGCGCUCUCUUC............x3
......................................................................CGUCCCGGGCGCUCUCUU.............x1
.......................................................................GUCCCGGGCGCUCUCUUCU...........x23
.......................................................................GUCCCGGGCGCUCUCUUC............x3
........................................................................UCCCGGGCGCUCUCUUCU...........x2

Sequence    1 Structure       1
 ENERGY =  -56.6    1
Bases     1 to  101

                 10        20        30        40
UG---------               C   A      G G   A  A C    A
           AGAAGAGAGCGCCCG GAC ACCUGU G CCU UG C UGCA G
           UCUUCUCUCGCGGGC CUG UGGACA C GGA GC G ACGU C
UAAGAGACGUG               C   C      A G   C  C C    U
 .        90        80        70        60        50
__________________________________________________
```

Esi-MIR11378

```
1       10        20        30        40        50        60        70        80        90        100

GGUACGGUAGAGGACAGAUCGUAAGGUAGUUUUGUCCUCUCCUCACGUCGAGAAGGGGACUAAACGUACCCUAUUUUUUGUCCUCCUUCGUACAAGU
.((((((..(((((((((..(((.((((((((.(((((((.(((.....))).))))))).)))).)))).)))..)))))))))..))))))....
.....GGUAGAGGACAGAUCGUAAGG.......................................................................x4*
.....GGUAGAGGACAGAUCGUAAG........................................................................x1
.....GGUAGAGGACAGAUCGUA..........................................................................x1
......................................................................CUAUUUUUUGUCCUCCUUCGU......x20*
......................................................................CUAUUUUUUGUCCUCCUUCGUA.....x1

Sequence    1 Structure       1
 ENERGY =  -45.8    1
Bases     1 to   97



           10        20         30        40
G---      UA         UC    A    -    U       C   AC
    GUACGG  GAGGACAGA  GUA GGUA GUUU GUCCUCU CUC  \
    CAUGCU  CUCCUGUUU  UAU CCAU CAAA CAGGGGA GAG  G
UGAA      UC         UU    C    G    U       A   CU
      90        80        70        60        50
__________________________________________________
```

Esi-MIR11379

```
1       10        20        30        40        50        60        70        80        90        100
AGUCGUGAAGAUCCCGAAUCAAACAUGGUGUUAACCUUCUCGAGAGCAGAUGACGAGAAAGUUAACACCAUGCGUGAUUCGGGAUCUUUUCGACUCGCUGCA
(((((.(((((((((((((((..((((((((((((.((((((..(.....)..)))))).))))))))))))..))))))))))))))).))))).......
```

```
.....UGAAGAUCCCGAAUCAAACAUG...........................................................................x246*
.....UGAAGAUCCCGAAUCAAACAU............................................................................x129
.....UGAAGAUCCCGAAUCAAACA.............................................................................x83
.....UGAAGAUCCCGAAUCAAACAUGG..........................................................................x4
.....UGAAGAUCCCGAAUCAAAC..............................................................................x1
......................................................................UGCGUGAUUCGGGAUCUUUUCG..........x4146*
......................................................................UGCGUGAUUCGGGAUCUUUU............x182
......................................................................UGCGUGAUUCGGGAUCUUUUCGA.........x101
......................................................................UGCGUGAUUCGGGAUCUUUUC...........x71
......................................................................UGCGUGAUUCGGGAUCUUU.............x6
......................................................................UGCGUGAUUCGGGAUCUUUUCGAC........x1
.......................................................................GCGUGAUUCGGGAUCUUUUCG..........x11
.......................................................................GCGUGAUUCGGGAUCUUUU............x2
.......................................................................GCGUGAUUCGGGAUCUUUUCGA.........x1
........................................................................CGUGAUUCGGGAUCUUUUCGA.........x9
........................................................................CGUGAUUCGGGAUCUUUUCG..........x2
.........................................................................GUGAUUCGGGAUCUUUUCG..........x1
..........................................................................UGAUUCGGGAUCUUUUCG..........x3

Sequence   1 Structure      1
 ENERGY =  -59.5    1
Bases    1 to  102

               10        20        30        40
-------     U               AA             C      AG GC
       AGUCG GAAGAUCCCGAAUCA  CAUGGUGUUAAC UUCUCG  A  \
       UCAGC UUUCUAGGGCUUAGU  GUACCACAAUUG AAGAGC  U  A
ACGUCGC     U               GC             A      AG AG
100        90        80        70        60        50
 _________________________________________________

Esi-MIR11380

1       10        20        30        40        50        60        70        80        90        100
CCCCGACUUUUCGGUUGUACUCUCUAAUUAGUACGCCGACAUAGAGGCAUUCCUUUAUGUCGUAGAUGCUAAUAGGCAGUACAAGCGAAACGUCGCGGG
(((((((.(((((.(((((((.((((.(((((((..(((((((((((....)))))))))))..).)))))))))).))))))).))))).)))).)))
.......UUUUCGGUUGUACUCUCUAAUU......................................................................x268*
.......UUUUCGGUUGUACUCUCUAAU.......................................................................x50
.......UUUUCGGUUGUACUCUCUAAUUA.....................................................................x3
.......UUUUCGGUUGUACUCUCUA.........................................................................x1
.......UUUUCGGUUGUACUCUCUAA........................................................................x1
........UUUCGGUUGUACUCUCUAAUU......................................................................x5
........UUUCGGUUGUACUCUCUAAU.......................................................................x1
........................................................................UAGGCAGUACAAGCGAAACGU......x93*
........................................................................UAGGCAGUACAAGCGAAAC........x2
........................................................................UAGGCAGUACAAGCGAAA.........x2
........................................................................UAGGCAGUACAAGCGAAACG.......x2

Sequence   1 Structure      1
 ENERGY =  -50.2    1
Bases    1 to   99

         10        20        30         40
   -    U     G       C    A      - GC           C
CCC CGAC UUUCG UUGUACU UCUA UUAGUA C  CGACAUAGAGG A
GGG GCUG AAAGC AACAUGA GGAU AAUCGU G  GCUGUAUUUCC U
   C    C     G       C    -      A AU           U
        90        80         70        60         50
 _________________________________________________

Esi-MIR11381

1       10        20        30        40        50        60        70        80        90        100
CACCUGAAUGCCAACAGAGCCGACCGUAUCCAACCUGCAGUGGUCUUAACACUACAGGUUGGAUGCGGUCGGCUCUGUUGGUAGGUGGGUGUUUAAGGGA
```

```
((((((..(((((((((((((((((((((((((((((.((((.......)))).)))))))))))))))))))))))))))))..)))))).........
....UGAAUGCCAACAGAGCCGACC...........................................................................x10*
....UGAAUGCCAACAGAGCCGA............................................................................x1
....................................................................UCGGCUCUGUUGGUAGGUGGG...........x1195*
....................................................................UCGGCUCUGUUGGUAGGUGG............x807
....................................................................UCGGCUCUGUUGGUAGGUG.............x151
....................................................................UCGGCUCUGUUGGUAGGU..............x11
....................................................................UCGGCUCUGUUGGUAGGUGGGU..........x2
.....................................................................CGGCUCUGUUGGUAGGUGGG...........x2
.....................................................................CGGCUCUGUUGGUAGGUGG............x2
.....................................................................CGGCUCUGUUGGUAGGUG.............x1
......................................................................GGCUCUGUUGGUAGGUGG............x3
```

```
Sequence   1 Structure      1
0 ENERGY =  -74.0    1
Bases    1 to  100

                 10        20        30        40
---------      AA                    C    GUC
         CACCUG  UGCCAACAGAGCCGACCGUAUCCAACCUG AGUG   \
         GUGGGU  AUGGUUGUCUCGGCUGGCGUAGGUUGGAC UCAC   U
AGGGAAUUU      GG                    A    AAU
.        90        80        70        60        50
```

Esi-MIR11382

```
1       10        20        30        40        50        60        70        80        90       100
UGUGUGGUGCAGCCGGGCUCGGCGCACUCCUGCCUCUGUAGAUUGCCUCGAGAUGUCUACAGAGGCAGGAGUGCGCCGACCCCAGCUGACCCACACAC
(((((((..((((.(((.(((((((((((((((((((((((((..(.....)..))))))))))))))))))))))))).))).)))).))))))).
.......UGCAGCCGGGCUCGGCGCACU......................................................................x691*
.......UGCAGCCGGGCUCGGCGCAC.......................................................................x2
.......UGCAGCCGGGCUCGGCGCA........................................................................x1
........GCAGCCGGGCUCGGCGCACU......................................................................x1
..................................UCUGUAGAUUGCCUCGAGAUG...........................................x1
.......................................................................UGCGCCGACCCCAGCUGACCC......x123*
.......................................................................UGCGCCGACCCCAGCUGACC.......x51
.......................................................................UGCGCCGACCCCAGCUGAC........x8
.......................................................................UGCGCCGACCCCAGCUGACCCA.....x3
.......................................................................UGCGCCGACCCCAGCUGA.........x3
```

```
Sequence   1 Structure      1
 ENERGY =  -78.6    1
Bases    1 to   98

         10        20        30        40
-       UG    C   C                         UG CU
 UGUGUGG  CAGC GGG UCGGCGCACUCCUGCCUCUGUAGAU  C  \
 ACACACC  GUCG CCC AGCCGCGUGAGGACGGAGACAUCUG  G  C
C       CA    A   C                         UA AG
       90        80        70        60        50
```

Esi-MIR11383

```
1       10        20        30        40        50        60        70        80        90       100
AGUCGACGUUAGCGAGGUCGCAGUCAACUUCUCUGACCGACCACGAUGUCUCGGUCAGGGAAGUCGAGUGCCGCCCCGCUAUCGUCGACGCGCCCA
.(((((((.(((((.(((.(((.((.((((((((((((((..........)))))))))))))).)).))).))).))))).)))))))......
...CGACGUUAGCGAGGUCGCAGU.........................................................................x1*
....GACGUUAGCGAGGUCGCAGUC........................................................................x1
....GACGUUAGCGAGGUCGCAGU.........................................................................x1
....GACGUUAGCGAGGUCGCAG..........................................................................x1
..............................................UGUCUCGGUCAGGGAAGUCGA.............................x1
...................................................................GUGCCGCCCCGCUAUCGUCGA........x23
```

```
...................................................................GUGCCGCCCCGCUAUCGUCG.........x1
....................................................................UGCCGCCCCGCUAUCGUCGAC.......x45*
....................................................................UGCCGCCCCGCUAUCGUCGA........x5
```

```
Sequence   1 Structure      1
 ENERGY =  -56.7    1
Bases    1 to   96

              10        20        30        40
A------       U     A   C   G  A              CCAC
       GUCGACG UAGCG GGU GCA UC ACUUCUCUGACCGA    G
       CAGCUGC AUCGC CCG CGU AG UGAAGGGACUGGCU    A
ACCCGCG       U     C   C   G  C              CUGU
     90        80        70        60        50
 __________________________________________________
```

Esi-MIR11384a and Esi-MIR11384b, have identical sequences but are located at two distinct loci in the genome, sctg_665-20503-20406 and sctg_630-13034-12937, respectively.

```
1       10        20        30        40        50        60        70        80        90       100
CGAAGGCAAGACAAAGCUAAAUGACGGGAUUCGUUGCCUUGAGUGAUGCACUCAUGAUAAACGCAUGCCGUCAUUUAGUUUUGUCGUGCCUUGAGAGA
..((((((.((((((((((((((((((.((.((((....((((((...)))))).....)))).)).)))))))))))))))))).))))))......
.....GCAAGACAAAGCUAAAUGACG........................................................................x1*
......................................................................UCAUUUAGUUUUGUCGUGCCU.......x81*
......................................................................UCAUUUAGUUUUGUCGUGCCUU......x4
.......................................................................CAUUUAGUUUUGUCGUGCCUU......x1
........................................................................AUUUAGUUUUGUCGUGCCU.......x1
Sequence   1 Structure      1
 ENERGY =  -50.7    1
Bases    1 to   98

            10        20        30         40
CG----      A                  G  U    GCCU-      A
      AAGGCA GACAAAGCUAAAUGACGG AU CGUU     UGAGUG \
      UUCCGU CUGUUUUGAUUUACUGCC UA GCAA     ACUCAC U
AGAGAG      G                  G  C    AUAGU      G
       90        80        70        60        50
 __________________________________________________
```

Esi-MIR11385

```
1       10        20        30        40        50        60        70        80        90       100
GUGCGGCGAGGGGAAUGGUUCGAAAAGGGAAGCUUGCGCAAAUAUCGUGGCCUAUUAGCGCUAGCUGCUCUUUUCGAACCAUUCCCCUCGCCGCAUCUAUUU
(((((((((((((((((((((((((((((.((((.((((.((((........)))).)))).)))).)))))))))))))))))))))))))))))......
.UGCGGCGAGGGGAAUGGUUCG................................................................................x1
.UGCGGCGAGGGGAAUGGUUC.................................................................................x1
...CGGCGAGGGGAAUGGUUCGAAAAGG..........................................................................x1
.......GAGGGGAAUGGUUCGAAAAGG..........................................................................x63*
.......GAGGGGAAUGGUUCGAAAAG...........................................................................x1
.......................................AAAUAUCGUGGCCUAUUAGCGCUAGC.....................................x1
......................................................................UUUUCGAACCAUUCCCCUCGC...........x3685*
......................................................................UUUUCGAACCAUUCCCCUCG............x1431
......................................................................UUUUCGAACCAUUCCCCUC.............x40
......................................................................UUUUCGAACCAUUCCCCU..............x23
......................................................................UUUUCGAACCAUUCCCCUCGCC..........x9
.......................................................................UUUCGAACCAUUCCCCUCGC...........x29
.......................................................................UUUCGAACCAUUCCCCUCGCC..........x20
.......................................................................UUUCGAACCAUUCCCCUCG............x13
........................................................................UUCGAACCAUUCCCCUCGCCG.........x7
........................................................................UUCGAACCAUUCCCCUCGC...........x2
........................................................................UUCGAACCAUUCCCCUCGCC..........x1
........................................................................UUCGAACCAUUCCCCUCG............x1
.........................................................................UCGAACCAUUCCCCUCGCCGC........x4
.........................................................................UCGAACCAUUCCCCUCGCC..........x2
```

```
Sequence    1 Structure       1
 ENERGY =   -72.6     1
Bases     1 to  102

              10        20        30        40
------                             A     U     A     UCG
      GUGCGGCGAGGGGAAUGGUUCGAAAAGGG AGCU GCGC AAUA    U
      UACGCCGCUCCCCUUACCAAGCUUUUCUC UCGA CGCG UUAU    G
UUUAUC                             G     U     A     CCG
100        90        80        70        60        50
 __________________________________________________
```

Esi-MIR11386

```
1       10         20         30         40         50         60         70         80         90         100
UGGUGCCCUCUCCGUGGAACUCGGCGAUGACCUCUUGGGCCCCGAUGGCAGCCAUCGGGGCCCAGGAGGUGGUAGCGGAGUUCCAAGGAGAGGUCGUCG
.((((.(((((((.((((((((.((.((.(((((((((((((((((((...))))))))))))))))))).)).)).)))))))).))))))).)))).
.....CCCUCUCCGUGGAACUCGGCGAUG......................................................................x1
........UCUCCGUGGAACUCGGCGAUG......................................................................x536*
........UCUCCGUGGAACUCGGCGAU.......................................................................x24
........UCUCCGUGGAACUCGGCGAUGA.....................................................................x5
........UCUCCGUGGAACUCGGCGA........................................................................x3
.........CUCCGUGGAACUCGGCGAUG......................................................................x2
...................................................CCAUCGGGGCCCAGGAGGUGG...........................x2
........................................................................UAGCGGAGUUCCAAGGAGAGG......x67*
........................................................................UAGCGGAGUUCCAAGGAGA........x4
........................................................................UAGCGGAGUUCCAAGGAGAG.......x2

Sequence    1 Structure       1
 ENERGY =   -78.3     1
Bases     1 to   99

        10        20        30        40
U    C       G        G  G  G                   C
 GGUG CCUCUCC UGGAACUC GC AU ACCUCUUGGGCCCCGAUGG \
 CUGC GGAGAGG ACCUUGAG CG UG UGGAGGACCCGGGGCUACC A
G    U       A        G  A  G                   G
        90        80        70        60
 __________________________________________________
```

Esi-MIR11387

```
1       10         20         30         40         50         60         70         80         90         100
GCCGAAUGAGUCCGACCUACCUCACGCGGAUUGCAGCCAUUCGGAGACUCCCGAUUCUCCGCAAUCCUCGUCAGGUAGGAUGGACUCCGUCGGCUUGUCCUU
(((((..(((((((.(((((((.(((.(((((((......((((......))))......))))))).))).))))))).)))))))..)))))........
....AAUGAGUCCGACCUACCUCAC.............................................................................x1
....AAUGAGUCCGACCUACCUCACG............................................................................x1*
......................................................................UCAGGUAGGAUGGACUCCGUCG..........x763*
......................................................................UCAGGUAGGAUGGACUCCGUC...........x112
......................................................................UCAGGUAGGAUGGACUCCGU............x107
......................................................................UCAGGUAGGAUGGACUCCG.............x8
......................................................................UCAGGUAGGAUGGACUCC..............x1
......................................................................UCAGGUAGGAUGGACUCCGUCGG.........x1
.......................................................................CAGGUAGGAUGGACUCCGUCG..........x81
.......................................................................CAGGUAGGAUGGACUCCGUC...........x7
.......................................................................CAGGUAGGAUGGACUCCGU............x1
........................................................................AGGUAGGAUGGACUCCGUCG..........x2
........................................................................AGGUAGGAUGGACUCCGUC...........x2
...........................................................................UAGGAUGGACUCCGUCGGCUU......x2

Sequence    1 Structure       1
 ENERGY =   -53.5     1
Bases     1 to  102
```

```
                10        20        30        40
--------     AU        A       C   C       AGCCAU    AG
        GCCGA  GAGUCCG CCUACCU ACG GGAUUGC      UCGG  A
        CGGCU  CUCAGGU GGAUGGA UGC CCUAACG      AGCC  C
UUCCUGUU     GC        A       C   U       CCUCUU    CU
100        90        80        70        60        50
 __________________________________________________
```

Esi-MIR11388

```
1       10        20        30        40        50        60        70        80        90        100
CCCGUUGAAGUUGAGGUUUUCGAGGUUGUGUUGCUGUUCGAGGAGUUGCUGCUGGAACCGCAGCAAAACCUCGAGAAUCUCAACCACAACGGG
(((((((..((((((((((((((((((.((((((.((((.((.((...)).)).)))).)))))).))))))))))))))))))..)))))))
....UUGAAGUUGAGGUUUUCGAGG....................................................................x1835
....UUGAAGUUGAGGUUUUCGAGGU...................................................................x99
....UUGAAGUUGAGGUUUUCGAG.....................................................................x41
....UUGAAGUUGAGGUUUUCGA......................................................................x21
.....UGAAGUUGAGGUUUUCGAGGU...................................................................x24163*
.....UGAAGUUGAGGUUUUCGAGG....................................................................x1091
.....UGAAGUUGAGGUUUUCGAGGUU..................................................................x339
.....UGAAGUUGAGGUUUUCGAG.....................................................................x66
.....UGAAGUUGAGGUUUUCGA......................................................................x48
......GAAGUUGAGGUUUUCGAGGU...................................................................x26
......GAAGUUGAGGUUUUCGAGG....................................................................x3
.......AAGUUGAGGUUUUCGAGGU...................................................................x3
........AGUUGAGGUUUUCGAGGU...................................................................x3
..........UUGAGGUUUUCGAGGUUGUGU..............................................................x2
.........................UUGUGUUGCUGUUCGAGGAGU...............................................x2
.........................UUGUGUUGCUGUUCGAGGAGUU..............................................x1
..........................UGUGUUGCUGUUCGAGGAGUUG.............................................x3
..........................UGUGUUGCUGUUCGAGGAGU...............................................x2
..........................UGUGUUGCUGUUCGAGGA.................................................x1
..........................UGUGUUGCUGUUCGAGGAGUU..............................................x1
.......................................GAGGAGUUGCUGCUGGAACCG.................................x1
.......................................GAGGAGUUGCUGCUGGAACC..................................x1
.........................................GGAGUUGCUGCUGGAACCG.................................x1
.....................................................................CUCGAGAAUCUCAACCACAAC...x18*
.....................................................................CUCGAGAAUCUCAACCACA.....x3
.....................................................................CUCGAGAAUCUCAACCACAA....x3
......................................................................UCGAGAAUCUCAACCACAACG..x16
```

```
Sequence   1 Structure      1
 ENERGY =  -59.4    1
Bases     1 to   93

        10        20        30        40
       AA                 G      U    G  G  U
CCCGUUG  GUUGAGGUUUUCGAGGUU UGUUGC GUUC AG AG \
GGGCAAC  CAACUCUAAGAGCUCCAA ACGACG CAAG UC UC U
       AC                 A      C    G  G  G
  90        80        70        60        50
 __________________________________________________
```

Esi-MIR11389

```
1       10        20        30        40        50        60        70        80        90        100
UUGUCGUGCACAACGUACCUUUCGAGAUAGGUGGCGACGACAGGUGACUCACCUGUCGUCGCCACCCAUCUCGAAUGGUACGUUGUGUAGCACCACAAGCG
((((.((((((((((((((.((((((((.(((((((((((((((((...))))))))))))))))).)))))))).))))))))))...)))).))))...
.UGUCGUGCACAACGUACCUUUCGAGA..........................................................................x1
......UGCACAACGUACCUUUCGAGA..........................................................................x35*
......UGCACAACGUACCUUUCGAGAU.........................................................................x1
........CACAACGUACCUUUCGAGA..........................................................................x1
```

```
...........................UAGGUGGCGACGACAGGUGACUCA..................................................x2
...........................UAGGUGGCGACGACAGGUGACUC...................................................x1
......................................................................UCGAAUGGUACGUUGUGUAGCA.........x111*
......................................................................UCGAAUGGUACGUUGUGUAGCACC.......x101
......................................................................UCGAAUGGUACGUUGUGUAGCAC........x90
......................................................................UCGAAUGGUACGUUGUGUAGC..........x53
......................................................................UCGAAUGGUACGUUGUGUAG...........x3
........................................................................GAAUGGUACGUUGUGUAGCAC........x1

Sequence   1 Structure      1
 ENERGY =  -73.4    1
Bases    1 to  101

              10        20        30        40
---    C    ---         U         A                 A
   UUGU GUGC   ACAACGUACC UUCGAGAU GGUGGCGACGACAGGUG \
   AACA CACG   UGUUGCAUGG AAGCUCUA CCACCGCUGCUGUCCAC C
GCG    C    AUG         U         C                 U
 .        90        80        70        60        50
 __________________________________________________

Esi-MIR11390

1       10        20        30        40        50        60        70        80        90        100
GGAUAGGUCGUUAGUUCUUGAGACACGGCGUUUGAAGAACAUGAAUUUGUUCCUCCAGCAACUCCAUGUCUCAAGAGCUAACGACCUAUCCGUUC
(((((((((((((((((((((((((.((.(((.((.(((((......))))).))....))).)).)))))))))))))))))))))))))....
..AUAGGUCGUUAGUUCUUGAGA........................................................................x3*
...UAGGUCGUUAGUUCUUGAGA........................................................................x1
......................................................................UCAAGAGCUAACGACCUAUCC....x256*
......................................................................UCAAGAGCUAACGACCUAUC.....x11

Sequence   1 Structure      1
 ENERGY =  -55.4    1
Bases    1 to   95

            10        20        30            40
----                         C  C   U---  A      UG
    GGAUAGGUCGUUAGUUCUUGAGACA GG GUU    GA GAACA  A
    CCUAUCCAGCAAUCGAGAACUCUGU CC CAA    CU CUUGU  A
CUUG                         A  U   CGAC  C      UU
    90        80        70        60        50
 __________________________________________________

Esi-MIR11391

1       10        20        30        40        50        60        70        80        90        100
UUUGCCUACGAUACACGGACGGAGAUCUUGAAACCAACGUUCCCGUCUGCCGGGGACAAUGAUUUACCAAGAUCUCGAGCGGUGUAUCGUGGGCACC
..((((((((((((((.(...((((((((((((.((..(((((((.....)))))))..)).)))..)))))))))...).))))))))))))))..
.....CUACGAUACACGGACGGAGAUC......................................................................x1
......UACGAUACACGGACGGAGAUC......................................................................x71*
......UACGAUACACGGACGGAGAU.......................................................................x15
......UACGAUACACGGACGGAGA........................................................................x3
........................................................................UCUCGAGCGGUGUAUCGUGGG....x38*
........................................................................UCUCGAGCGGUGUAUCGUGG.....x7
........................................................................UCUCGAGCGGUGUAUCGUG......x1

Sequence   1 Structure      1
 ENERGY =  -50.9    1
Bases    1 to   97

        10        20        30           40
UU              G ACG         --   C  AC       UC
  UGCCUACGAUACAC G   GAGAUCUUG  AAA CA  GUUCCCG  \
  ACGGGUGCUAUGUG C   CUCUAGAAC  UUU GU  CAGGGGC  U
```

```
CC              G GAG         CA    A  AA       CG
      90        80        70        60        50
 __________________________________________________
```

Esi-MIR11392

```
1       10        20        30        40        50        60        70        80        90        100
GAGGUGGCAGCGGAGCUCGAGGGCGAGGCCAUCGAUUUUACUGUGCUGUGCCUCGGCAGAGUCGAUACCCUCGCCCUGGAACUCGGUCAUUACCUCCUGG
(((((((..((.(((.((.(((((((((..((((((((.....(((((.....)))))))))))))..))))))))).)).))).))..)))))))....
..GGUGGCAGCGGAGCUCGAGGGCGAGG........................................................................x1
....UGGCAGCGGAGCUCGAGGGCG...........................................................................x1
.......CAGCGGAGCUCGAGGGCGAGG........................................................................x4*
......................................................................UCGCCCUGGAACUCGGUCAUU.........x2040*
......................................................................UCGCCCUGGAACUCGGUCAU..........x96
......................................................................UCGCCCUGGAACUCGGUCAUUA........x41
......................................................................UCGCCCUGGAACUCGGUCA...........x13
......................................................................UCGCCCUGGAACUCGGUCAUUAC.......x4
......................................................................UCGCCCUGGAACUCGGUC............x4
......................................................................UCGCCCUGGAACUCGGUCAUUACC......x1
.......................................................................CGCCCUGGAACUCGGUCAUU.........x28
.......................................................................CGCCCUGGAACUCGGUCA...........x2
........................................................................GCCCUGGAACUCGGUCAUU.........x7
........................................................................GCCCUGGAACUCGGUCAUUA........x1
........................................................................GCCCUGGAACUCGGUCAU..........x1

Sequence   1 Structure      1
0 ENERGY =  -51.7    1
Bases    1 to  100

            10        20        30        40        50
----       CA  G   C  G         CC        UACUG     UG
    GAGGUGG  GC GAG UC AGGGCGAGG  AUCGAUUU     UGCUG  \
    CUCCAUU  UG CUC AG UCCCGCUCC  UAGCUGAG     ACGGC  C
GGUC       AC  G   A  G         CA        -----     UC
.        90        80        70        60
 __________________________________________________
```

Esi-MIR11393

```
1       10        20        30        40        50        60        70        80        90        100
CACAACGAGUGUCAGCAGCCUGCGUGCUGCUCUUCCCAUUUCAACUUGUCGGUCUUGGGAUGAGCAGCACGCAGGCUGCUGACACUCGUUGCACCA
..((((((((((((((((((((((((((((((.(((((.................))))).))))))))))))))))))))))))))))))).....
.....CGAGUGUCAGCAGCCUGCGUG......................................................................x7*
.....CGAGUGUCAGCAGCCUGCGU.......................................................................x1
.....................................................................CGCAGGCUGCUGACACUCGUU......x136*
.....................................................................CGCAGGCUGCUGACACUCGU.......x2

Sequence   1 Structure      1
 ENERGY =  -75.2    1
Bases    1 to   96

           10        20        30        40
CA---                              U     UUUCAACU
     CAACGAGUGUCAGCAGCCUGCGUGCUGCUC UCCCA        \
     GUUGCUCACAGUCGUCGGACGCACGACGAG AGGGU        U
ACCAC                              U     UCUGGCUG
     90        80        70        60        50
 __________________________________________________
```

Esi-MIR11394

```
1       10        20        30        40        50        60        70        80        90        100
CCAUACGUUAGCACUAGCGGGACCUUGCAACUCUUCGCUGACCGUGUCCUUCGAGGGAAGAGUUGUAUGAUCCCGCUUGUGCUUUCGCUUGGCGACU
```

```
(((..((..(((((.(((((((.(.(((((((((((.(((((...))).....)).))))))))))).).))))))).)))))..))..))).....
..AUACGUUAGCACUAGCGGGACCU........................................................................x1
...UACGUUAGCACUAGCGGGACC.........................................................................x80*
...UACGUUAGCACUAGCGGGACCU........................................................................x60
...UACGUUAGCACUAGCGGGAC..........................................................................x8
...UACGUUAGCACUAGCGGGA...........................................................................x6
........................UUGCAACUCUUCGCUGACCGUGUC.................................................x4
........................UUGCAACUCUUCGCUGACCGU....................................................x1
.........................................CCGUGUCCUUCGAGGGAAGAG...................................x1
...................................................................UGAUCCCGCUUGUGCUUUCGC.........x2
...................................................................UGAUCCCGCUUGUGCUUUCGCUU.......x1
.....................................................................AUCCCGCUUGUGCUUUCGCUU.......x2
......................................................................UCCCGCUUGUGCUUUCGCUUG......x259*
......................................................................UCCCGCUUGUGCUUUCGCUU.......x62
......................................................................UCCCGCUUGUGCUUUCGCU........x2

Sequence   1 Structure      1
 ENERGY =  -48.6    1
Bases     1 to   97

             10        20        30             40
-----   UA  UU     U       C U           G  -----   C
     CCA  CG  AGCAC AGCGGGA C UGCAACUCUUC CU     GAC \
     GGU  GC  UCGUG UCGCCCU G AUGUUGAGAAG GA     CUG G
UCAGC   UC  UU     U       A U           G  GCUUC   U
      90        80        70        60        50
 __________________________________________________
```

Esi-MIR11395

```
1       10        20        30        40        50        60        70        80        90        100
AGGAAGGUAGACCCGGCAAAGUUCGAGGACGAUUGCUCGCCGCUUCUACCUCCGCGACGAGCAAUCGUCCUCAAACUUUUGCCGGGUCUACCUUCCUCCUCU
(((((((((((((((((((((((.(((((((((((((((.(((..........))).))))))))))))))).))).)))))))))))))))))))).....
......GUAGACCCGGCAAAGUUCGAGG..........................................................................x3
.......UAGACCCGGCAAAGUUCGAGG..........................................................................x108*
.......UAGACCCGGCAAAGUUCGAG...........................................................................x8
.......UAGACCCGGCAAAGUUCGAGGA.........................................................................x3
.......UAGACCCGGCAAAGUUCGA............................................................................x2
................................................................UCGUCCUCAAACUUUUGCCGGG................x1
...................................................................UCCUCAAACUUUUGCCGGGUCU.............x1
......................................................................UCAAACUUUUGCCGGGUCUACC..........x428*
......................................................................UCAAACUUUUGCCGGGUCUAC...........x61
......................................................................UCAAACUUUUGCCGGGUCUA............x18
......................................................................UCAAACUUUUGCCGGGUCUACCU.........x2
......................................................................UCAAACUUUUGCCGGGUC..............x1
........................................................................AAACUUUUGCCGGGUCUACC..........x2

Sequence   1 Structure      1
 ENERGY =  -75.9    1
Bases     1 to  102

             10        20         30        40
-----                    -   C               C   UUCU
     AGGAAGGUAGACCCGGCAAA GUU GAGGACGAUUGCUCG CGC    A
     UCCUUCCAUCUGGGCCGUUU CAA CUCCUGCUAACGAGC GCG    C
UCUCC                    U   A               A   CCUC
100        90        80        70        60        50
 __________________________________________________
```

Esi-MIR11396a

```
1       10        20        30        40        50        60        70        80        90        100
GAACGGUCCACGACGGCCAGCUCAAGGGAUGCUUUUGUUCAGCACAAUGUUCUGGACAAGAAGCAUCCCUUUAGCUGGUUGCCGUAGAGCUUUCCGAGAAA
(((.(.((.(((.(((((((((.(((((((((((((((((((.((...)).)))))).))))))))))))).))))))))).))).)).).))).......
```

```
....GGUCCACGACGGCCAGCUCAA............................................................................x2
.....GUCCACGACGGCCAGCUCAAG...........................................................................x811*
.....GUCCACGACGGCCAGCUCAA............................................................................x110
.....GUCCACGACGGCCAGCUCA.............................................................................x25
.....GUCCACGACGGCCAGCUCAAGG..........................................................................x3
.....GUCCACGACGGCCAGCUC..............................................................................x2
......UCCACGACGGCCAGCUCAAGG..........................................................................x96
......UCCACGACGGCCAGCUCAAG...........................................................................x51
......UCCACGACGGCCAGCUCA.............................................................................x1
......UCCACGACGGCCAGCUCAA............................................................................x1
........CACGACGGCCAGCUCAAG...........................................................................x1
........................................AGCACAAUGUUCUGGACAA..........................................x2
.........................................GCACAAUGUUCUGGACAA..........................................x1
....................................................................CUUUAGCUGGUUGCCGUAGAGC...........x2
.....................................................................UUUAGCUGGUUGCCGUAGAGC...........x8412
.....................................................................UUUAGCUGGUUGCCGUAGAG............x769
.....................................................................UUUAGCUGGUUGCCGUAGAGCU..........x661
.....................................................................UUUAGCUGGUUGCCGUAGA.............x134
.....................................................................UUUAGCUGGUUGCCGUAGAGCUU.........x123
.....................................................................UUUAGCUGGUUGCCGUAGAGCUUUC.......x8
.....................................................................UUUAGCUGGUUGCCGUAGAGCUUU........x6
.....................................................................UUUAGCUGGUUGCCGUAG..............x2
......................................................................UUAGCUGGUUGCCGUAGAGCU..........x316747*
......................................................................UUAGCUGGUUGCCGUAGAGC...........x19465
......................................................................UUAGCUGGUUGCCGUAGAGCUU.........x9923
......................................................................UUAGCUGGUUGCCGUAGAG............x3968
......................................................................UUAGCUGGUUGCCGUAGA.............x852
......................................................................UUAGCUGGUUGCCGUAGAGCUUU........x331
......................................................................UUAGCUGGUUGCCGUAGAGCUUUC.......x130
.......................................................................UAGCUGGUUGCCGUAGAGCU..........x973
.......................................................................UAGCUGGUUGCCGUAGAGCUU.........x918
.......................................................................UAGCUGGUUGCCGUAGAGC...........x41
.......................................................................UAGCUGGUUGCCGUAGAGCUUU........x20
.......................................................................UAGCUGGUUGCCGUAGAG............x5
.......................................................................UAGCUGGUUGCCGUAGAGCUUUC.......x1
........................................................................AGCUGGUUGCCGUAGAGCU..........x452
........................................................................AGCUGGUUGCCGUAGAGC...........x15
........................................................................AGCUGGUUGCCGUAGAGCUU.........x14
........................................................................AGCUGGUUGCCGUAGAGCUUUC.......x1
.........................................................................GCUGGUUGCCGUAGAGCU..........x45
.........................................................................GCUGGUUGCCGUAGAGCUU.........x2
..........................................................................CUGGUUGCCGUAGAGCU..........x1
```

```
Sequence   1 Structure      1
 ENERGY =  -51.3    1
Bases    1 to  101
```

```
               10        20        30         40
-------   C G  C   A         C             -      C  A
       GAA G UC ACG CGGCCAGCU AAGGGAUGCUUUU GUUCAG AC \
       CUU C AG UGC GUUGGUCGA UUCCCUACGAAGA CAGGUC UG A
AAGAGCC   U G  A   C         U             A      U  U
 .        90        80        70        60        50
```

Esi-MIR11396b

```
1       10         20         30         40         50         60         70         80         90         100
GGAACGGUCAACGACAACAAGCUGAAGGGAUGCUUUUGUUCAGCACGAUGUUCUGAACGAAAGCAUCCCUUCAGCUAGUUGUCGUUGAGUUUUC
.(((.(.((((((((((.(((((((((((((((((((((((((.((...)).))))))))))))))))))))))))).)))))))))).).)))
```

```
......GUCAACGACAACAAGCUGAAG...................................................................x128*
......GUCAACGACAACAAGCUGAA....................................................................x2
..................................UUUGUUCAGCACGAUGUUCU........................................x1
.....................................GUUCAGCACGAUGUUCUGA......................................x1
........................................CAGCACGAUGUUCUGAACGAAA................................x1
........................................CAGCACGAUGUUCUGAACG...................................x1
..........................................GCACGAUGUUCUGAACGAAAGC..............................x6
..........................................GCACGAUGUUCUGAACGAAA................................x4
..........................................GCACGAUGUUCUGAACGAAAG...............................x1
..........................................GCACGAUGUUCUGAACGAAAGCA.............................x1
....................................................CUGAACGAAAGCAUCCCU........................x1
.................................................................UCCCUUCAGCUAGUUGUCGUU........x8
.....................................................................UUCAGCUAGUUGUCGUUGAGU....x4
......................................................................UCAGCUAGUUGUCGUUGAGUU...x10098*
......................................................................UCAGCUAGUUGUCGUUGAGU....x6048
......................................................................UCAGCUAGUUGUCGUUGA......x214
......................................................................UCAGCUAGUUGUCGUUGAG.....x162
......................................................................UCAGCUAGUUGUCGUUGAGUUU..x38
......................................................................UCAGCUAGUUGUCGUUGAGUUUU.x1
.......................................................................CAGCUAGUUGUCGUUGAGUU...x22
.......................................................................CAGCUAGUUGUCGUUGAGU....x10
........................................................................AGCUAGUUGUCGUUGAGUU...x38
........................................................................AGCUAGUUGUCGUUGAGU....x23
.........................................................................GCUAGUUGUCGUUGAGUU...x26
.........................................................................GCUAGUUGUCGUUGAGUUU..x1

Sequence   1 Structure      1
 ENERGY =  -63.5    1
Bases    1 to   94

        10        20        30        40
G   C G           A                        C  G
 GAA G UCAACGACAAC AGCUGAAGGGAUGCUUUUGUUCAG AC \
 CUU U AGUUGCUGUUG UCGACUUCCCUACGAAAGCAAGUC UG A
-   U G           A                        U  U
    90        80        70        60        50
 __________________________________________________
```

Esi-MIR11397

```
1       10        20        30        40        50        60        70        80        90        100
UGAGUUUCUCUGCGUCGACCCUAACGGGGCUAGUUGGUGGACAGGAAACGUCCUUACCAGCUAGGCCCCGUUGGAUCGACGCUGUUGAACUCGUACAG
.((((((..(.(((((((.((.((((((((((((((((((((.......))))..)))))))).)))))))))).))))))).)..))))))......
....UUUCUCUGCGUCGACCCUAACG........................................................................x7*
....UUUCUCUGCGUCGACCCUAAC.........................................................................x2
.....UUCUCUGCGUCGACCCUAAC.........................................................................x1
......................................................................UUGGAUCGACGCUGUUGAACU.......x174*
......................................................................UUGGAUCGACGCUGUUGAAC........x13
......................................................................UUGGAUCGACGCUGUUGAACUC......x3
......................................................................UUGGAUCGACGCUGUUGAA.........x2
.......................................................................UGGAUCGACGCUGUUGAACU.......x4
........................................................................GGAUCGACGCUGUUGAACUC......x13
........................................................................GGAUCGACGCUGUUGAACU.......x2

Sequence   1 Structure      1
 ENERGY =  -55.4    1
Bases    1 to   98

             10        20        30           40
U-----      CU U       C  U        -        --    AGG
      GAGUUU  C GCGUCGA CC AACGGGGC UAGUUGGU  GGAC   \
      CUCAAG  G CGCAGCU GG UUGCCCCG AUCGACCA  CCUG   A
GACAUG      UU U       A  -        G        UU    CAA
       90        80         70        60        50
```

Esi-MIR11398

```
1       10        20        30        40        50        60        70        80        90       100       110       120       130       140
CAAGGGGAAGUGGUCCCGUCGAAGUCGUCGAUCUCCCAAUGGAACUCCAUCUUGAAUGAUUUCAGACUAUGGACUUCUUUUGGGAGAUGGAUGAAUUCGAAGGGACAAUAUUUCUUUGGUAGAUUGGUUUCACGU
((((((((.((.(((((.(((((.(((((.(((((((((.((((.(((((.(((((....)))))...))))).)))).))))))))).))))).))))).))))).)).))))))))((.((......))))..
.................GUCGAAGUCGUCGAUCUCCCA.................................................................................................x1
..................UCGAAGUCGUCGAUCUCCCAA................................................................................................x13*
..................UCGAAGUCGUCGAUCUCCCAAU...............................................................................................x5
.................................................................................GGGAGAUGGAUGAAUUCGAAG.................................x23*
.................................................................................GGGAGAUGGAUGAAUUCGAA..................................x3
.................................................................................GGGAGAUGGAUGAAUUCGA...................................x2
.................................................................................GGGAGAUGGAUGAAUUCGAAGGG...............................x1
..................................................................................GGAGAUGGAUGAAUUCGAAGG................................x6
...................................................................................GAGAUGGAUGAAUUCGAAGGGA..............................x1
....................................................................................AGAUGGAUGAAUUCGAAGGG...............................x2
....................................................................................AGAUGGAUGAAUUCGAAGG................................x1
.......................................................................................UGGAUGAAUUCGAAGGGACAA...........................x1

Sequence   1 Structure      1
 ENERGY =  -60.6    1
Bases    1 to  135

          10        20        30        40        50
          A  G     G     G     G         U    C     C--     U
--CAAGGGGA GU GUCCC UCGAA UCGUC AUCUCCCAA GGAA UCCAU   UUGAA G
  GUUUCUUU UA CAGGG AGCUU AGUAG UAGAGGGUU UCUU AGGUA   GACUU A
\         A  A     A     A     G         U    C     UCA     U
        110       100        90        80        70        60

   120
  --  A  UU
    GU GA   G
    CA CU   G
  UG  -  UU
      130
```

Figure S3. Additional loci in the Ectocarpus genome that resemble miRNA loci.

Weak Candidate 1

```
1       10        20        30        40        50        60        70        80        90       100       110       120       130       140
UUGGUCGUUCUUCCUUUCAACGUCUCCUAGGCUCGAUGAUCGCCAAAAAGGAACACAAACAGCGGAGAUGCUGUUUGUAUUUCUUGUGGCGAUCAUCGAGCCUAGGAGACGUUACAAGGACGGACGACGAAUGACG
((.(((((((.(((((..(((((((((((((((((((((((((((..((((((.((((((((((....)))))))))).)))))).)))))))))))))))))))))))))))..))))).))))))).)).....

......................UCUCCUAGGCUCGAUGAUCGCC............................................................................................x28
......................UCUCCUAGGCUCGAUGAUCGC.............................................................................................x17
......................UCUCCUAGGCUCGAUGAUCG..............................................................................................x2
......................UCUCCUAGGCUCGAUGAUCGCCA...........................................................................................x1
.......................CUCCUAGGCUCGAUGAUCGCCA...........................................................................................x3
.......................CUCCUAGGCUCGAUGAUCGCC............................................................................................x2
........................UCCUAGGCUCGAUGAUCGCC............................................................................................x213*
........................UCCUAGGCUCGAUGAUCGC.............................................................................................x12
........................UCCUAGGCUCGAUGAUCGCCA...........................................................................................x10
........................UCCUAGGCUCGAUGAUCG..............................................................................................x1
.........................................................................................CGAUCAUCGAGCCUAGGAGA...........................x2*
.........................................................................................CGAUCAUCGAGCCUAGGAGACG.........................x1
......................................................................................................UAGGAGACGUUACAAGGACG..............x1
```

```
Most common read: UCCUAGGCUCGAUGAUCGCC (freq 213)

Complementarity: 20/20

Best mirbase match: NONE (e-value: NONE)



Sequence   1 Structure      1
 ENERGY =   -93.2    13p
Bases    1 to  136

             10        20        30        40        50        60
-----  G       U     UC                          AA      C          G
     UU GUCGUUC UCCUU  AACGUCUCCUAGGCUCGAUGAUCGCCA  AAGGAA ACAAACAGCG A
     AA CAGCAGG AGGAA  UUGCAGAGGAUCCGAGCUACUAGCGGU  UUCUUU UGUUUGUCGU G
GCAGU  G       C     CA                          G-      A          A
    130       120       110       100        90         80        70
```

____

Weak Candidate 2

```
1        10        20        30        40        50        60        70        80        90        100       110       120       130       140       150       160       170
UGGCCGCACACAACGCCCUGUCCGCUUGAGAGAACUGUCGUAGCAGCGACGUCAUUGGAAGCCUCGGCUUUUGUCGUGGCGACAAAUGUCGAGGCAACAAACGUGGAGGUUUCCAAUGCUAUCGCUGCUCCGCAGGUCUUUGAAGCGGACAGGGCGUUGUGUGAGGUCAAGG
(((((.(((((((((((((((((((((.(((((.(((.((.((((((((...(((((((((((((.((.(((((.((..((((....))))..)).))))).)).)))))))))))))...)))))))).))))).))))).))))))))))))))))))))).)))))...

UGGCCGCACACAACGCCCUGU.......................................................................................................................................................x1
......................CGCUUGAGAGAACUGUCGUAGC................................................................................................................................x4*
......................CGCUUGAGAGAACUGUCGUAG.................................................................................................................................x1
...........................................................................................................GGUUUCCAAUGCUAUCGCUGC............................................x1
................................................................................................................................UCCGCAGGUCUUUGAAGCGGA.......................x59*
```

```
Most common read: UCCGCAGGUCUUUGAAGCGGA (freq 59)

Complementarity: 18/21

Best mirbase match: hsa-miR-6840-5p (e-value: 5.8)

Query  1   UCCGCAGGUCUUUG  14
           || |||||||||||
Sbjct  23  UCUGCAGGUCUUUG  10


Sequence   1 Structure      1
 ENERGY =  -118.9    1
Bases    1 to  172

           10        20        30        40        50        60        70        80
---     G                     G     A   U  U        CGU            G  U     C  GG    A
   UGGCC CACACAACGCCCUGUCCGCUU AGAGA CUG CG AGCAGCGA   CAUUGGAAGCCUC GC UUUGU GU  CGAC A
   ACUGG GUGUGUUGCGGGACAGGCGAA UUUCU GAC GC UCGUCGCU   GUAACCUUUGGAG UG AAACA CG  GCUG A
GGA     A                     G     G   -  C        AUC            G  C     A  GA    U
170       160       150       140        130       120       110       100        90
```

____

Weak Candidate 3

```
1       10        20        30        40        50        60        70        80        90       100       110       120       130       140       150
UUAUUUCUUCUUUGAAGUGUAGGGACCCCCGGUUGGGAUACGAUUCGAUGGCCCGCCCCAAACGCCAAUGAACGCGCGUUCAUGCACGUUUUGAAGCGGGCCAGCGGGUUAUAUCCCAACCGCGAUUCCUAUCCUUCAAGGAAAGGAAACGAA
...((((((((((((((.(((((((....(((((((((((.((((((.((((((((..((((((...((((((....))))))...)).))))..))))))))).)))))).)))))))))))...))))))).)))))))).)))))).....

........UCUUUGAAGUGUAGGGACCCC............................................................................................................................x1
........UCUUUGAAGUGUAGGGACCCCCG..........................................................................................................................x1
........UCUUUGAAGUGUAGGGACCCCCGGU........................................................................................................................x1
..........UUUGAAGUGUAGGGACCCCCG..........................................................................................................................x16
..........UUUGAAGUGUAGGGACCCCCGG.........................................................................................................................x14
..........UUUGAAGUGUAGGGACCCCCGGU........................................................................................................................x4
..........UUUGAAGUGUAGGGACCCCC...........................................................................................................................x1
...........UUGAAGUGUAGGGACCCCCGGU........................................................................................................................x199
...........UUGAAGUGUAGGGACCCCCGG.........................................................................................................................x159
...........UUGAAGUGUAGGGACCCCCG..........................................................................................................................x87
...........UUGAAGUGUAGGGACCCCC...........................................................................................................................x4
...........UUGAAGUGUAGGGACCCCCGGUU.......................................................................................................................x1
............UGAAGUGUAGGGACCCCCGGUU.......................................................................................................................x2292*
............UGAAGUGUAGGGACCCCCGGU........................................................................................................................x108
............UGAAGUGUAGGGACCCCCGG.........................................................................................................................x46
............UGAAGUGUAGGGACCCCCG..........................................................................................................................x21
............UGAAGUGUAGGGACCCCCGGUUG......................................................................................................................x4
............UGAAGUGUAGGGACCCCC...........................................................................................................................x1
.............GAAGUGUAGGGACCCCCGGUU.......................................................................................................................x6
...............AGUGUAGGGACCCCCGGUU.......................................................................................................................x1
................................UUGGGAUACGAUUCGAUGG......................................................................................................x1
................................UUGGGAUACGAUUCGAUGGCC....................................................................................................x1
.................................UGGGAUACGAUUCGAUGGCCC...................................................................................................x9
.................................UGGGAUACGAUUCGAUGGCC....................................................................................................x4
.................................UGGGAUACGAUUCGAUGGCCCG..................................................................................................x1
.................................UGGGAUACGAUUCGAUGGC.....................................................................................................x1
..................................GGGAUACGAUUCGAUGGC.....................................................................................................x1
.......................................................................................................................CCGCGAUUCCUAUCCUUCAAG.............x2*

Most common read: UGAAGUGUAGGGACCCCCGGUU (freq 2292)

Complementarity: 17/22

Best mirbase match: tca-miR-3845-3p (e-value: 2.0)

Query  3   AAGUGUAGGGACCCCCG  19
           |||||| ||||||| ||
Sbjct  3   AAGUGUUGGGACCCACG  19


Sequence   1 Structure      1
 ENERGY =   -77.2    1
Bases    1 to  153

           10        20        30        40        50        60         70
UUA--      -        U       CCCC           C      A        CC    -  CCA      G
     UUUCUU CUUUGAAG GUAGGGA    CGGUUGGGAUA GAUUCG UGGCCCGC  CAAA CG   AUGAAC C
     AAGGAA GGAACUUC UAUCCUU    GCCAACCCUAU UUGGGC ACCGGGCG  GUUU GC   UACUUG G
AAGCA      A        C       AGC-           A      G        AA    U  ACG      C
 150       140       130        120       110       100        90        80
```

Weak Candidate 4

```
1       10        20        30        40        50        60        70        80        90       100       110       120       130       140       150
CAGCUCGGCGGGCAAGCCAAGGUUCUUGGCCAUCAUCCUCAGGUUCCACCGGAGGUUGAACUCCAGCCACGCCGAGUGCUUGCUCGAGUUUCAACCUCCGGUGGAACUUGAGGAUGAUGGCCAAGCACCGUGGCUUACCCACGUC
.....((..(((.((((((.(((.(((((((((((((((((((((((((((((((((((((((.((((((.....)))...))).))).)))))))))))))))))))))))))))))))))))).))).)))))).))).))..
```

```
........CGGGCAAGCCAAGGUUCUUGGCCA.................................................................................................................x1
......................UUCUUGGCCAUCAUCCUCAGG......................................................................................................x1
..............................CAUCAUCCUCAGGUUCCACCGGA............................................................................................x1
................................UCAUCCUCAGGUUCCACCGGA............................................................................................x37638*
................................UCAUCCUCAGGUUCCACCGGAG...........................................................................................x1510
................................UCAUCCUCAGGUUCCACCGG.............................................................................................x770
................................UCAUCCUCAGGUUCCACCG..............................................................................................x11
................................UCAUCCUCAGGUUCCACC...............................................................................................x3
.................................CAUCCUCAGGUUCCACCGGA............................................................................................x258
.................................CAUCCUCAGGUUCCACCGG.............................................................................................x1
.................................CAUCCUCAGGUUCCACCGGAG...........................................................................................x1
..................................AUCCUCAGGUUCCACCGGA............................................................................................x196
..................................AUCCUCAGGUUCCACCGGAG...........................................................................................x2
...................................UCCUCAGGUUCCACCGGA............................................................................................x1
................................................................................................UCCGGUGGAACUUGAGGAUGA............................x14
..................................................................................................CGGUGGAACUUGAGGAUGAUG..........................x444*
..................................................................................................CGGUGGAACUUGAGGAUGAU...........................x19
..................................................................................................CGGUGGAACUUGAGGAUGA............................x7
...................................................................................................GGUGGAACUUGAGGAUGAUG..........................x4
```

Most common read: UCAUCCUCAGGUUCCACCGGA (freq 37638)

Complementarity: 21/21

Best mirbase match: esi-miR3455-5p (e-value: 0.22)

```
Query  1   UCAUCCUCAGGUUCCACCGGA  21
           |||| |||| |||||| ||||
Sbjct  1   UCAUUCUCAAGUUCCAUCGGA  21
```

```
Sequence   1 Structure      1
 ENERGY =  -105.0    1
Bases    1 to  145

        10        20        30        40        50        60            70
CAGCU  GC   C      A   U                                    -   C   ---   GC
     CG  GGG AAGCCA GGU CUUGGCCAUCAUCCUCAGGUUCCACCGGAGGUUGAA CUC AGC   CAC  \
     GC  CCC UUCGGU CCA GAACCGGUAGUAGGAGUUCAAGGUGGCCUCCAACUU GAG UCG   GUG  C
CU---  A-   A      G   C                                    U   C   UUC   AG
       140       130       120       110       100        90        80
```

Weak Candidate 5

```
1       10        20        30        40        50        60        70        80        90        100       110       120       130       140       150
CCGUUUCCUCGAUCAGUGCUUCUAGUGAAUUGGCAAGAUCCUCGACCAUGAUGAGCAUGUUGCGUUGGCGCAUUGGUCGCCAAUGCCGCAUGCCCGUCAUGCUCCCGGAUCUUCCUAAUGCACCAGAAGCAUUGGUCGAGGAAGCGG
(((((((((((((((((((((((.(((.(((((.(((((((..((.(((((((.((((((.((((((((((....).))))))))).)))))).))))))).))..))))))).))))).))).)))))))))))))))))))))))

........UCGAUCAGUGCUUCUAGUGAA......................................................................................................................x1
............UCAGUGCUUCUAGUGAAUUGG..................................................................................................................x1
..............................UGGCAAGAUCCUCGACCAUGAUG..............................................................................................x1
..............................UGGCAAGAUCCUCGACCAUGAU...............................................................................................x1
...............................GGCAAGAUCCUCGACCAUGAUG..............................................................................................x1
................................GCAAGAUCCUCGACCAUGAUG..............................................................................................x61*
................................GCAAGAUCCUCGACCAUGAU...............................................................................................x6
.................................CAAGAUCCUCGACCAUGAUG..............................................................................................x2
..................................AAGAUCCUCGACCAUGAUG..............................................................................................x1
................................................................................................UCAUGCUCCCGGAUCUUCCUA..............................x2766*
................................................................................................UCAUGCUCCCGGAUCUUCCU...............................x668
................................................................................................UCAUGCUCCCGGAUCUUCCUAA.............................x88
................................................................................................UCAUGCUCCCGGAUCUUCC................................x9
................................................................................................UCAUGCUCCCGGAUCUUCCUAAU............................x5
```

```
................................................................................................UCAUGCUCCCGGAUCUUC.................................x3
.................................................................................................CAUGCUCCCGGAUCUUCCUA..............................x10
.................................................................................................CAUGCUCCCGGAUCUUCCU...............................x2
..................................................................................................AUGCUCCCGGAUCUUCCUA..............................x8
......................................................................................................................UGCACCAGAAGCAUUGGUCGA........x35
......................................................................................................................UGCACCAGAAGCAUUGGUCGAG.......x7
......................................................................................................................UGCACCAGAAGCAUUGGU...........x1
.......................................................................................................................GCACCAGAAGCAUUGGUCGA........x1
.......................................................................................................................GCACCAGAAGCAUUGGUCGAG.......x1
.........................................................................................................................ACCAGAAGCAUUGGUCGAG.......x1
```

Most common read: UCAUGCUCCCGGAUCUUCCUA (freq 2766)

Complementarity: 17/21

Best mirbase match: NONE (e-value: NONE)

```
Sequence   1 Structure      1
 ENERGY =   -96.3    1
Bases    1 to  147

        10        20        30        40        50        60        70
                       A   A     C       UC  C       A      U         - A
CCGUUUCCUCGAUCAGUGCUUCU GUG AUUGG AAGAUCC  GA CAUGAUG GCAUGU GCGUUGGCG C U
GGCGAAGGAGCUGGUUACGAAGA CAC UAAUC UUCUAGG  CU GUACUGC CGUACG CGUAACCGC G U
                       C   G     C       CC  C       C      C         U G
     140       130       120       110       100        90        80
```

Weak Candidate 6

```
1       10         20         30         40         50         60         70         80         90         100        110        120        130        140        150        160        170
CUCAUUCCUCAUGUUCCGAGAUGAGAUGAGAUGGGACGGAAAAAUUCCAAGGUGAGCUUCAUCGAAUUCCGAUCAAGGCUUGAUUUCUUGAUCGGAAUCUGAUGAAGGUCACUUCGAAAUGCUCCGUCCCUUCCCAUCCCAUCCCGGAGCAUGAGGAAUUCCGAU
...(((((((((((((((.((((.((((.((.((((((((..(.(((..((((((.((((((((.(((((((((((((.......))))))))))))).)))))))).)))))).))).)..)))))))).)).)))).)))).)))))))))))))))......

.....UCCUCAUGUUCCGAGAUGAGA...........................................................................................................................................x1
........UCAUGUUCCGAGAUGAGAUGA........................................................................................................................................x1
.................GAGAUGAGAUGAGAUGGGACG...............................................................................................................................x61*
.................GAGAUGAGAUGAGAUGGGACGG..............................................................................................................................x3
.................GAGAUGAGAUGAGAUGGGA.................................................................................................................................x1
.................GAGAUGAGAUGAGAUGGGAC................................................................................................................................x1
..................AGAUGAGAUGAGAUGGGACGG..............................................................................................................................x3
..................AGAUGAGAUGAGAUGGGACGGA.............................................................................................................................x3
..................AGAUGAGAUGAGAUGGGACG...............................................................................................................................x2
.....................UGAGAUGAGAUGGGACGG..............................................................................................................................x1
......................................GAAAAAUUCCAAGGUGAGCUUCA........................................................................................................x1
.............................................................UCGAAUUCCGAUCAAGGCUUGAUU................................................................................x2
.............................................................UCGAAUUCCGAUCAAGGCUUGAUUU...............................................................................x1
............................................................................GGCUUGAUUUCUUGAUCGG......................................................................x1
..............................................................................................................................UCCCUUCCCAUCCCAUCCCGG..................x775*
..............................................................................................................................UCCCUUCCCAUCCCAUCCCGGA.................x46
..............................................................................................................................UCCCUUCCCAUCCCAUCCCG...................x24
..............................................................................................................................UCCCUUCCCAUCCCAUCCC....................x3
..............................................................................................................................UCCCUUCCCAUCCCAUCC.....................x1
................................................................................................................................CCUUCCCAUCCCAUCCCG...................x1
................................................................................................................................CCUUCCCAUCCCAUCCCGG..................x1
........................................................................................................................................UCCCAUCCCGGAGCAUGAGGA........x1
```

Most common read: UCCCUUCCCAUCCCAUCCCGG (freq 775)

Complementarity: 17/21

```
Best mirbase match: cin-miR-5605-5p (e-value: 1.5)

Query  1   UCCCUUCCCAUCCCAUCCC  19
           |||| ||  ||||||||||
Sbjct  1   UCCCAUCUUAUCCCAUCCC  19


Sequence   1 Structure      1
 ENERGY =   -95.0    1
Bases    1 to  165

           10        20        30        40        50        60        70        80
CUC---               A    A    A  U        AA A   CA      G        A             CUU
      AUUCCUCAUGUUCCG GAUG GAUG GA GGGACGGA  A UUC  AGGUGA CUUCAUCG AUUCCGAUCAAGG   \
      UAAGGAGUACGAGGC CUAC CUAC CU CCCUGCCU  U AAG  UUCACU GAAGUAGU UAAGGCUAGUUCU   G
UAGCCU               C    C    C  U        CG A   C-      G        C             UUA
   160       150       140       130       120        110       100        90
```

Weak Candidate 7

```
1       10        20        30        40        50        60        70        80        90        100
CCUUUCAACGUCUCCUAGGCUCGAUGAUCGCCAAAAAGGAACACAAACAGCGGAGAUGCUGUUUGUAUUUCUUGUGGCGAUCAUCGAGCCUAGGAGACGUU
.......((((((((((((((((((((((((((..((((((.((((((((((....)))))))))).)))))).))))))))))))))))))))))))))).

..........UCUCCUAGGCUCGAUGAUCGCC.....................................................................x28
..........UCUCCUAGGCUCGAUGAUCGC......................................................................x17
..........UCUCCUAGGCUCGAUGAUCG.......................................................................x2
..........UCUCCUAGGCUCGAUGAUCGCCA....................................................................x1
...........CUCCUAGGCUCGAUGAUCGCCA....................................................................x3
...........CUCCUAGGCUCGAUGAUCGCC.....................................................................x2
............UCCUAGGCUCGAUGAUCGCC.....................................................................x213*
............UCCUAGGCUCGAUGAUCGC......................................................................x12
............UCCUAGGCUCGAUGAUCGCCA....................................................................x10
............UCCUAGGCUCGAUGAUCG.......................................................................x1
.............................................................................CGAUCAUCGAGCCUAGGAGA....x2*
.............................................................................CGAUCAUCGAGCCUAGGAGACG..x1
```

Most common read: UCCUAGGCUCGAUGAUCGCC (freq 213)

Complementarity: 20/20

Best mirbase match: NONE (e-value: NONE)

```
Sequence   1 Structure      1
 ENERGY =   -74.2    1
Bases    1 to  101

        10        20        30        40        50
CCUUUCA                          AA      C          G
       ACGUCUCCUAGGCUCGAUGAUCGCCA  AAGGAA ACAAACAGCG A
       UGCAGAGGAUCCGAGCUACUAGCGGU  UUCUUU UGUUUGUCGU G
U------                          G-      A          A
     100        90        80         70        60
```

Weak Candidate 8

```
1       10        20        30        40        50        60        70        80        90        100
UUGUCGCCGCGAAGCGAUAGUCCCACUAAGCUGGACGCGUGUCGUAGCGUAUGACACGUGUCUAGCUUAGUGGGAGUACCGCUUCGCGGCAAUAAGCAGA
((((.((((((((((.((.(((((((((((((((((((((((((((...))))))))))))))))))))))))))).)).)))))))))).)))).....

...UCGCCGCGAAGCGAUAGUCCC............................................................................x1
......CCGCGAAGCGAUAGUCCCACU.........................................................................x6*
......CCGCGAAGCGAUAGUCCCAC..........................................................................x1
......................................................................UGGGAGUACCGCUUCGCGGCA.........x2854*
......................................................................UGGGAGUACCGCUUCGCGGC..........x212
......................................................................UGGGAGUACCGCUUCGCGGCAA........x99
......................................................................UGGGAGUACCGCUUCGCGG...........x38
......................................................................UGGGAGUACCGCUUCGCG............x4
......................................................................UGGGAGUACCGCUUCGCGGCAAU.......x1
.......................................................................GGGAGUACCGCUUCGCGGCA.........x16
.......................................................................GGGAGUACCGCUUCGCGGCAA........x1
```

Most common read: UGGGAGUACCGCUUCGCGGCA (freq 2854)

Complementarity: 18/21

Best mirbase match: tae-miR1117 (e-value: 1.8)

```
Query  5   AGUACCGCUUCGCGGCA  21
           ||||||| |||| ||||
Sbjct  2   AGUACCGGUUCGUGGCA  18
```

```
Sequence   1 Structure      1
0 ENERGY =   -75.5    1
Bases    1 to  100

             10        20        30        40
-----    C           A  G                        G
     UUGU GCCGCGAAGCG UA UCCCACUAAGCUGGACGCGUGUCGUA \
     AAUA CGGCGCUUCGC AU AGGGUGAUUCGAUCUGUGCACAGUAU C
AGACG    A           C  G                        G
.        90        80        70        60        50
```

Weak Candidate 9

```
1       10        20        30        40        50        60        70        80        90        100       110
CACCGUUUACGAGGCACGCUGUAUCAUCGAAGCGGCGGCCUCGCGUAUCUGAGACCACCGCCUCGGUGAUACAGUGCGCCUCGUCGACGGUGGUGCAACAAACC
((((((.(((((((.((((((((((((((.((((.((.((((......)))).)).)))).)))))))))))))).))))))).))))))..............

....GUUUACGAGGCACGCUGUAUC...............................................................................x25*
....GUUUACGAGGCACGCUGUAU................................................................................x13
....GUUUACGAGGCACGCUGUAUCA..............................................................................x1
....GUUUACGAGGCACGCUGU..................................................................................x1
.....UUUACGAGGCACGCUGUAUCA..............................................................................x14
.....UUUACGAGGCACGCUGUAUC...............................................................................x4
.....UUUACGAGGCACGCUGUAU................................................................................x1
.......UACGAGGCACGCUGUAUCAUCG...........................................................................x2
.....................................................................UACAGUGCGCCUCGUCGACGG..............x22370*
.....................................................................UACAGUGCGCCUCGUCGACG...............x291
.....................................................................UACAGUGCGCCUCGUCGACGGU.............x131
.....................................................................UACAGUGCGCCUCGUCGAC................x9
.....................................................................UACAGUGCGCCUCGUCGA.................x9
......................................................................ACAGUGCGCCUCGUCGACGG..............x464
......................................................................ACAGUGCGCCUCGUCGACG...............x6
......................................................................ACAGUGCGCCUCGUCGACGGU.............x1
.......................................................................CAGUGCGCCUCGUCGACGG..............x234
```

```
.......................................................................CAGUGCGCCUCGUCGACG...............x1
........................................................................AGUGCGCCUCGUCGACGG..............x105
```

Most common read: UACAGUGCGCCUCGUCGACGG (freq 22370)

Complementarity: 19/21

Best mirbase match: mdo-miR-7386c-5p (e-value: 8.6)

```
Query  2   ACAGUGCGCCUCGUCGACG  20
           | |||||||||| || | |
Sbjct  21  AUAGUGCGCCUCAUCCAAG  3
```

```
Sequence   1 Structure      1
 ENERGY =   -66.2    1
Bases    1 to  104

                    10        20        30        40
------------       U       A              A    C  C    CG
            CACCGUU ACGAGGC CGCUGUAUCAUCGA GCGG GG CUCG  U
            GUGGCAG UGCUCCG GUGACAUAGUGGCU CGCC CC GAGU  A
CCAAACAACGUG       C       C              C    A  A    CU
  100        90        80        70        60        50
```

____

Weak Candidate 10

```
1       10        20        30        40        50        60        70        80        90        100
UGAUGGUUGUGAACCUCCCUGCCGCGAACAUCACUUGUUCCAGCCGCAAACAUGCACAUUGCACAUGUGAUGUUUGCGGCAGGAAGGUCUACAACCAUCA
(((((((((((.((((.(((((((((((((((((.(((..(((..(((....)))...))).))).))))))))))))))))).)))).)))))))))))

......UUGUGAACCUCCCUGCCGCGAA........................................................................x1
.........UGAACCUCCCUGCCGCGAACA......................................................................x2430*
.........UGAACCUCCCUGCCGCGAAC.......................................................................x83
.........UGAACCUCCCUGCCGCGAACAU.....................................................................x23
.........UGAACCUCCCUGCCGCGAA........................................................................x7
..........GAACCUCCCUGCCGCGAACA......................................................................x1
........................................................................UUUGCGGCAGGAAGGUCUACA.......x45*
........................................................................UUUGCGGCAGGAAGGUCUAC........x1
........................................................................UUUGCGGCAGGAAGGUCUACAA......x1
```

Most common read: UGAACCUCCCUGCCGCGAACA (freq 2430)

Complementarity: 19/21

Best mirbase match: esi-miR3451-3p (e-value: 0.023)

```
Query  1   UGAACCUCCCUGCCGCGA  18
           ||||||||||||||||||
Sbjct  18  UGAACCUCCCUGCCGCGA  1
```

```
Sequence   1 Structure      1
0 ENERGY =   -59.2    1
Bases    1 to  100

        10        20        30        40         50
           A    C                 U   UC   CC-   A
UGAUGGUUGUG ACCU CCUGCCGCGAACAUCAC UGU  CAG   GCA A
ACUACCAACAU UGGA GGACGGCGUUUGUAGUG ACA  GUU   CGU C
           C    A                 U   C-   ACA   A
```

```
.        90        80        70         60
```

____

Weak Candidate 11

```
1       10         20         30         40         50         60         70         80         90         100
ACAGCUUCUUCUUGGCCUGCGCCUUGCUGCUCGCCGCCGAUGGUGCCCGGCACCAGCGGCGGCGAGCAGCAAGGCACCGAACCAGAAGAGGCCGUCG
((.((((((((.(..(.(.((((((((((((((((((.((((((...)))))).))))))))))))))))))).).)..).))))))))).))..

.....UUCUUCUUGGCCUGCGCCUUG.......................................................................x1
......UCUUCUUGGCCUGCGCCUUGC......................................................................x40*
......UCUUCUUGGCCUGCGCCUUG.......................................................................x4
......UCUUCUUGGCCUGCGCCUU........................................................................x1
......................................................................AAGGCACCGAACCAGAAGAGG......x16*
```

Most common read: UCUUCUUGGCCUGCGCCUUGC (freq 40)

Complementarity: 16/21

Best mirbase match: NONE (e-value: NONE)

```
Sequence   1 Structure      1
 ENERGY =   -71.6    1
Bases    1 to   97

          10        20        30        40
--  A         U GC U C                   A      C
  AC GCUUCUUCU G  C G GCCUUGCUGCUCGCCGCCG UGGUGC \
  UG CGGAGAAGA C  G C CGGAACGACGAGCGGCGGC ACCACG C
GC  C         C AA C A                   G      G
      90        80        70        60        50
```

____

Weak Candidate 12

```
1       10         20         30         40         50         60         70         80         90         100
AGCCUGGGGAACCAACUGAGGCAAGCAGUGAUACCCUGCCACGCGCUGGUGUAGCAGGGCAUGCGCUGCUUGCCUGAGGGUAUUUCCAUGGCCAUGG
.(((((((((((..((.((((((((((((((.((((((.((((....)))).)))))).)).))))))))))))).))))).)))))).))).....

....UGGGGAACCAACUGAGGCAAGC.......................................................................x3*
.....................................................................UUGCCUGAGGGUAUUUCCAUGG......x138*
......................................................................UGCCUGAGGGUAUUUCCAUGG......x7
.......................................................................GCCUGAGGGUAUUUCCAUGG......x1
```

Most common read: UUGCCUGAGGGUAUUUCCAUGG (freq 138)

Complementarity: 19/22

Best mirbase match: bbe-miR-4864-5p (e-value: 9.3)

```
Query  2   UGCCUGAGGGUAUUUCCAU  20
           |||||||||  || ||| |
Sbjct  19  UGCCUGAGGUGAUCUCCCU  1
```

```
Sequence   1 Structure      1
 ENERGY =   -57.6    1
Bases    1 to   97
```

```
             10         20        30         40
A----   -      -   AA  G            -  A      C    G
     GCC UGGGGA ACC  CU AGGCAAGCAGUG AU CCCUGC ACGC C
     CGG ACCUUU UGG  GA UCCGUUCGUCGC UA GGGACG UGUG U
GGUAC   U      A   --  G            G  C      A    G
      90        80          70        60        50
```

Weak Candidate 13

```
1       10         20         30         40         50         60         70         80         90         100
AAGGCGGCCAGGAAGAACUUUAGCCAGACUGAGACGUCCACUGACCGUCAGCGGGCCGCCUCCGUCUGGCUAAAGUUCUUCCGAGCCGUCCGGUAGUGCG
..((((((..(((((((((((((((((((.(((.(((((.((((...)))).))).)).))).)))))))))))))))))))..))))))..........

...GCGGCCAGGAAGAACUUUAGC............................................................................x3
...GCGGCCAGGAAGAACUUUAGCCA..........................................................................x1
....CGGCCAGGAAGAACUUUAGCC...........................................................................x19*
....CGGCCAGGAAGAACUUUAGC............................................................................x16
....CGGCCAGGAAGAACUUUA..............................................................................x4
....CGGCCAGGAAGAACUUUAG.............................................................................x2
....CGGCCAGGAAGAACUUUAGCCA..........................................................................x2
.....GGCCAGGAAGAACUUUAGCCA..........................................................................x1
......GCCAGGAAGAACUUUAGCCA..........................................................................x2
.....................................................................CUAAAGUUCUUCCGAGCCGUC..........x2957*
.....................................................................CUAAAGUUCUUCCGAGCCGU...........x31
.....................................................................CUAAAGUUCUUCCGAGCCGUCC.........x22
.....................................................................CUAAAGUUCUUCCGAGCC.............x2
......................................................................UAAAGUUCUUCCGAGCCGUC..........x49
......................................................................UAAAGUUCUUCCGAGCCGUCC.........x29
......................................................................UAAAGUUCUUCCGAGCCGUCCGG.......x3
......................................................................UAAAGUUCUUCCGAGCCGUCCGGU......x3
......................................................................UAAAGUUCUUCCGAGCCGUCCG........x2
......................................................................UAAAGUUCUUCCGAGCCGU...........x1
...........................................................................UUCUUCCGAGCCGUCCGGUA.....x1
...............................................................................UCCGAGCCGUCCGGUAGUGCGx1
```

Most common read: CUAAAGUUCUUCCGAGCCGUC (freq 2957)

Complementarity: 19/21

Best mirbase match: cbr-miR-124c (e-value: 8.6)

```
Query  2   UAAAGUUCUUCCGAGCCGU  20
           ||| |  ||||| ||||||
Sbjct  1   UAAGGCACUUCCAAGCCGU  19
```

```
Sequence   1 Structure      1
0 ENERGY =   -61.9    1
Bases    1 to  100

                10        20        30         40
AA--------      CA                   U   A  -   A    C
          GGCGGC  GGAAGAACUUUAGCCAGAC GAG CG UCC CUGA \
          CUGCCG  CCUUCUUGAAAUCGGUCUG CUC GC GGG GACU C
GCGUGAUGGC      AG                   C   C  C   C    G
.        90        80        70        60        50
```

Weak Candidate 14

```
1       10        20        30        40        50        60        70        80        90        100
GGACCGUGAUGUGCGCGUUGGAUCAGUGCCACGGCAACUCGGACUCAGACGUCUGGGUUGCCGUGGAACUGAUCCAACGUGCACAACACGGUCCGAA
((((((((.((((((((((((((((((.((((((((((((((((......)))))))))))))))).)))))))))))))))))).))))))))...

.....GUGAUGUGCGCGUUGGAUCAG.......................................................................x3
......UGAUGUGCGCGUUGGAUCAGU......................................................................x240*
......UGAUGUGCGCGUUGGAUCAG.......................................................................x4
......UGAUGUGCGCGUUGGAUCA........................................................................x2
..........................UGCCACGGCAACUCGGAC.....................................................x2
..........................UGCCACGGCAACUCGGACUCAGA................................................x1
.....................................................................UGAUCCAACGUGCACAACACG.......x3*
```

Most common read: UGAUGUGCGCGUUGGAUCAGU (freq 240)

Complementarity: 20/21

Best mirbase match: cel-miR-44-5p (e-value: 5.8)

```
Query  2   GAUGUGCGCGUUGG  15
           ||||||| ||||||
Sbjct  4   GAUGUGCUCGUUGG  17
```

```
Sequence   1 Structure      1
 ENERGY =   -83.3    1
Bases    1 to   97

           10        20        30        40
---        A                  G                UC
   GGACCGUG UGUGCGCGUUGGAUCAGU CCACGGCAACUCGGAC  A
   CCUGGCAC ACACGUGCAACCUAGUCA GGUGCCGUUGGGUCUG  G
AAG        A                  A                CA
      90        80        70        60        50
```

Weak Candidate 15

```
1       10        20        30        40        50        60        70        80        90        100
GCCCCGGUAACGAGGACGGCGGCACUUUUGCAAGUGUCGCGAUUAUCAAUUGUGAUCGCGACACUUGCGCAAGCGCCGCCAUCAUCGUCACCAAGGCA
(((..(((.((((.((.((((((.(((.((((((((((((((((((.....)))))))))))))))))).))).)))))).)).)))).)))..))).

.......UAACGAGGACGGCGGCACUUU......................................................................x1860*
.......UAACGAGGACGGCGGCACUU.......................................................................x68
.......UAACGAGGACGGCGGCACU........................................................................x11
.......UAACGAGGACGGCGGCACUUUU.....................................................................x7
........AACGAGGACGGCGGCACUUU......................................................................x2
..........CGAGGACGGCGGCACUUU......................................................................x1
.......................................................................AGCGCCGCCAUCAUCGUCACC......x37*
.......................................................................AGCGCCGCCAUCAUCGUCA........x3
.......................................................................AGCGCCGCCAUCAUCGUCACCA.....x1
........................................................................GCGCCGCCAUCAUCGUCACC......x2
```

Most common read: UAACGAGGACGGCGGCACUUU (freq 1860)

Complementarity: 16/21

Best mirbase match: hsa-miR-371b-5p (e-value: 4.8)

```
Query  6   AGGACGGCGGCACUUU  21
           | || |||||||||||
Sbjct  7   AAGAUGGCGGCACUUU  22
```

```
Sequence   1 Structure      1
 ENERGY =   -62.7    1
Bases    1 to   98

         10        20        30        40
-   CC   A    G  C      A   U                CA
 GCC  GGU ACGA GA GGCGGC CUU UGCAAGUGUCGCGAUUAU  \
 CGG  CCA UGCU CU CCGCCG GAA GCGUUCACAGCGCUAGUG  A
A   AA   C    A  A      C   C                UU
       90        80        70        60        50
```

____

```
Weak Candidate 16

1       10        20        30        40        50        60        70        80        90        100
GAAAGGCUUUACAACAACCAGCUCAAGGAAUAUUUUGGUUCAGAGCAAUAUGCUGAACGAAAGUAUCCCGUAAGCUGGUUGUCGUAGAGCCUUUCCAAAA
((((((((((((.((((((((((...((.(((((((.((((((.........)))))).))))))).))...)))))))))).)))))))))))).....

......CUUUACAACAACCAGCUCAAG.........................................................................x14*
......CUUUACAACAACCAGCUCAA..........................................................................x2
......CUUUACAACAACCAGCUCAAGG........................................................................x2
........UUACAACAACCAGCUCAAGGA.......................................................................x2
.....................................................................GUAAGCUGGUUGUCGUAGAGCC.........x3
.....................................................................GUAAGCUGGUUGUCGUAGAG...........x1
.....................................................................GUAAGCUGGUUGUCGUAGA............x1
......................................................................UAAGCUGGUUGUCGUAGAGCC.........x155362*
......................................................................UAAGCUGGUUGUCGUAGAGC..........x43185
......................................................................UAAGCUGGUUGUCGUAGAG...........x6657
......................................................................UAAGCUGGUUGUCGUAGA............x1618
......................................................................UAAGCUGGUUGUCGUAGAGCCU........x445
......................................................................UAAGCUGGUUGUCGUAGAGCCUU.......x63
......................................................................UAAGCUGGUUGUCGUAGAGCCUUU......x7
.......................................................................AAGCUGGUUGUCGUAGAGCC.........x436
.......................................................................AAGCUGGUUGUCGUAGAGC..........x29
.......................................................................AAGCUGGUUGUCGUAGAG...........x8
.......................................................................AAGCUGGUUGUCGUAGAGCCU........x2
........................................................................AGCUGGUUGUCGUAGAGCC.........x700
........................................................................AGCUGGUUGUCGUAGAGC..........x36
.........................................................................GCUGGUUGUCGUAGAGCC.........x19
...........................................................................UGGUUGUCGUAGAGCCUUUCC....x1
............................................................................GGUUGUCGUAGAGCCUUUCC....x1

Most common read: UAAGCUGGUUGUCGUAGAGCC (freq 155362)

Complementarity: 18/21

Best mirbase match: NONE (e-value: NONE)


Sequence   1 Structure      1
0 ENERGY =   -52.7    1
Bases    1 to  100

             10        20        30        40
-----            A          CAA  A       G      AGCA
     GAAAGGCUUUAC ACAACCAGCU   GG AUAUUUU GUUCAG    \
     CUUUCCGAGAUG UGUUGGUCGA   CC UAUGAAA CAAGUC    A
AAAAC            C          AUG  C       G      GUAU
.        90        80        70        60        50
```

```
_______________________________________________


Weak Candidate 17

1       10        20        30        40        50        60        70        80        90        100
GAAAGGCUCUACAACAACCAGCUUACGGGAUGUUUUGGUUCAGAGCAAUAUGCUGAACGAAAGUAUCCCAUAAGCUGGUUGUCGUAAAGCCUUUCCAAAC
((((((((.(((.((((((((((((.((((((((((.((((((.........)))))).)))))))))).)))))))))))).))).)))))))).....

..AAGGCUCUACAACAACCAGCUUACG.........................................................................x2
......CUCUACAACAACCAGCUUACG.........................................................................x106*
......CUCUACAACAACCAGCUUA...........................................................................x1
......CUCUACAACAACCAGCUUAC..........................................................................x1
.......UCUACAACAACCAGCUUACG.........................................................................x22
.......UCUACAACAACCAGCUUACGG........................................................................x14
.........UACAACAACCAGCUUACGGGA......................................................................x57
.................................................AUGCUGAACGAAAGUAUCCCA..............................x1
......................................................................UAAGCUGGUUGUCGUAAAGCC.........x31096*
......................................................................UAAGCUGGUUGUCGUAAAGC..........x6716
......................................................................UAAGCUGGUUGUCGUAAAG...........x1018
......................................................................UAAGCUGGUUGUCGUAAA............x160
......................................................................UAAGCUGGUUGUCGUAAAGCCU........x37
......................................................................UAAGCUGGUUGUCGUAAAGCCUU.......x2
.......................................................................AAGCUGGUUGUCGUAAAGCC.........x16
.......................................................................AAGCUGGUUGUCGUAAAG...........x4
.......................................................................AAGCUGGUUGUCGUAAAGC..........x3
........................................................................AGCUGGUUGUCGUAAAGCC.........x9
........................................................................AGCUGGUUGUCGUAAAGC..........x4
.........................................................................GCUGGUUGUCGUAAAGCC.........x3

Most common read: UAAGCUGGUUGUCGUAAAGCC (freq 31096)

Complementarity: 19/21

Best mirbase match: mmu-miR-5622-5p (e-value: 4.0)

Query  1   UAAGCUGGUUGUCGUAAA  18
           |||||||| ||| || ||
Sbjct  18  UAAGCUGGGUGUGGUGAA  1


Sequence   1 Structure      1
0 ENERGY =   -55.5    1
Bases    1 to  100

             10        20        30        40
-----        C   A           C          G      AGCA
     GAAAGGCU UAC ACAACCAGCUUA GGGAUGUUUU GUUCAG    \
     CUUUCCGA AUG UGUUGGUCGAAU CCCUAUGAAA CAAGUC    A
CAAAC        A   C           A          G      GUAU
.       90        80        70        60        50

_______________________________________________


Weak Candidate 18

1       10        20        30        40        50        60        70        80        90        100       110
GAAAGGCUCUACAACAACCAGCUUCUGGGAUAUUUUGGUUCAGAGCAAUAUGCUGAACGAAAGUAUCCCAUAAGCUGGUUGUCGUAGAGCCUUUCCAGAACCAC
((((((((((((.(((((((((((.(((((((((((.((((((.........)))))).))))))))))).))))))))))).)))))))))))).........

......CUCUACAACAACCAGCUUCUG.............................................................................x77*
.......UCUACAACAACCAGCUUCUG.............................................................................x2
.................................................AUGCUGAACGAAAGUAUCCCA..................................x1
```

```
....................................................................CAUAAGCUGGUUGUCGUAGAGC..............x1
.....................................................................AUAAGCUGGUUGUCGUAG.................x1
.....................................................................AUAAGCUGGUUGUCGUAGAGCC.............x1
......................................................................UAAGCUGGUUGUCGUAGAGCC.............x155362*
......................................................................UAAGCUGGUUGUCGUAGAGC..............x43185
......................................................................UAAGCUGGUUGUCGUAGAG...............x6657
......................................................................UAAGCUGGUUGUCGUAGA................x1618
......................................................................UAAGCUGGUUGUCGUAGAGCCU............x445
......................................................................UAAGCUGGUUGUCGUAGAGCCUU...........x63
......................................................................UAAGCUGGUUGUCGUAGAGCCUUU..........x7
.......................................................................AAGCUGGUUGUCGUAGAGCC.............x436
.......................................................................AAGCUGGUUGUCGUAGAGC..............x29
.......................................................................AAGCUGGUUGUCGUAGAG...............x8
.......................................................................AAGCUGGUUGUCGUAGAGCCU............x2
........................................................................AGCUGGUUGUCGUAGAGCC.............x700
........................................................................AGCUGGUUGUCGUAGAGC..............x36
.........................................................................GCUGGUUGUCGUAGAGCC.............x19
...........................................................................UGGUUGUCGUAGAGCCUUUCC........x1
............................................................................GGUUGUCGUAGAGCCUUUCC........x1
..............................................................................UUGUCGUAGAGCCUUUCCAGA.....x1
...............................................................................UGUCGUAGAGCCUUUCCAGA.....x1
```

Most common read: UAAGCUGGUUGUCGUAGAGCC (freq 155362)

Complementarity: 19/21

Best mirbase match: NONE (e-value: NONE)

```
Sequence   1 Structure      1
 ENERGY =   -65.0    1
Bases    1 to  104

                 10        20        30        40
---------            A           C           G      AGCA
         GAAAGGCUCUAC ACAACCAGCUU UGGGAUAUUUU GUUCAG    \
         CUUUCCGAGAUG UGUUGGUCGAA ACCCUAUGAAA CAAGUC    A
CACCAAGAC            C           U           G      GUAU
  100        90        80        70        60        50
```

Weak Candidate 19

```
1        10        20        30        40        50        60        70        80        90        100       110
GAAAGGCUCUACAACAACCAGCUUACGGGAUGUUUUGGUUCAGGGCAAUAUGCUGAACGAAAGUAUCCCAUAAGCUGGUUGUCGUCGAGCCUUUCCAGAACCAC
(((((((((.((.((((((((((((.((((((((((.((((((.........)))))).)))))))))).)))))))))))).)).))))))))).........

..AAGGCUCUACAACAACCAGCUUACG.............................................................................x2
......CUCUACAACAACCAGCUUACG.............................................................................x106*
......CUCUACAACAACCAGCUUA...............................................................................x1
......CUCUACAACAACCAGCUUAC..............................................................................x1
.......UCUACAACAACCAGCUUACG.............................................................................x22
.......UCUACAACAACCAGCUUACGG............................................................................x14
.........UACAACAACCAGCUUACGGGA..........................................................................x57
.................................................AUGCUGAACGAAAGUAUCCCA..................................x1
....................................................................CAUAAGCUGGUUGUCGUCGAGC..............x3
....................................................................CAUAAGCUGGUUGUCGUCGAGCC.............x1
.....................................................................AUAAGCUGGUUGUCGUCGAGCC.............x3
......................................................................UAAGCUGGUUGUCGUCGAGCC.............x49416*
......................................................................UAAGCUGGUUGUCGUCGAGC..............x25402
......................................................................UAAGCUGGUUGUCGUCGAG...............x5942
......................................................................UAAGCUGGUUGUCGUCGA................x1055
```

```
......................................................................UAAGCUGGUUGUCGUCGAGCCU............x175
......................................................................UAAGCUGGUUGUCGUCGAGCCUU...........x41
......................................................................UAAGCUGGUUGUCGUCGAGCCUUU..........x3
.......................................................................AAGCUGGUUGUCGUCGAGCC.............x120
.......................................................................AAGCUGGUUGUCGUCGAGC..............x50
.......................................................................AAGCUGGUUGUCGUCGAG...............x16
.......................................................................AAGCUGGUUGUCGUCGAGCCU............x2
........................................................................AGCUGGUUGUCGUCGAGCC.............x56
........................................................................AGCUGGUUGUCGUCGAGC..............x31
........................................................................AGCUGGUUGUCGUCGAGCCU............x1
.........................................................................GCUGGUUGUCGUCGAGCC.............x9
```

Most common read: UAAGCUGGUUGUCGUCGAGCC (freq 49416)

Complementarity: 19/21

Best mirbase match: asu-miR-133-5p (e-value: 1.8)

```
Query  5   CUGGUUGUCGUCGAGCC  21
           |||||||| | ||||||
Sbjct  2   CUGGUUGUAGCCGAGCC  18
```

```
Sequence   1 Structure      1
 ENERGY =   -58.2    1
Bases    1 to  104

                 10        20        30        40
---------         U  A            C          G      GGCA
         GAAAGGCUC AC ACAACCAGCUUA GGGAUGUUUU GUUCAG    \
         CUUUCCGAG UG UGUUGGUCGAAU CCCUAUGAAA CAAGUC    A
CACCAAGAC         C  C            A          G      GUAU
  100        90        80        70        60        50
```

____

Weak Candidate 20

```
1        10        20        30        40        50        60        70        80        90        100       110
GCAAGACUCCACAACAAGCAGCUCAAGGGAUAUUUUGGUUCAGAACAAUGUGCUGAGCAAAACUAUCCCUUGAGCUGGUUGUUGUAGAGCCUUUCCAGAACCAC
..(((.(((.(((((((.((((((((((((((.(((.((((((.((...)).)))))).))).)))))))))))))).))))))).))).)))...........

......CUCCACAACAAGCAGCUCAAG.............................................................................x56*
......CUCCACAACAAGCAGCUCAA..............................................................................x3
.......UCCACAACAAGCAGCUCAAG.............................................................................x2
................................UUUUGGUUCAGAACAAUGUGC...................................................x1
.....................................................................UUGAGCUGGUUGUUGUAGAGC..............x11
.....................................................................UUGAGCUGGUUGUUGUAGAG...............x4
.....................................................................UUGAGCUGGUUGUUGUAGA................x2
......................................................................UGAGCUGGUUGUUGUAGAGCC.............x205866*
......................................................................UGAGCUGGUUGUUGUAGAGC..............x80033
......................................................................UGAGCUGGUUGUUGUAGAG...............x9732
......................................................................UGAGCUGGUUGUUGUAGA................x2455
......................................................................UGAGCUGGUUGUUGUAGAGCCU............x1427
......................................................................UGAGCUGGUUGUUGUAGAGCCUU...........x77
......................................................................UGAGCUGGUUGUUGUAGAGCCUUU..........x10
.......................................................................GAGCUGGUUGUUGUAGAGCC.............x918
.......................................................................GAGCUGGUUGUUGUAGAGC..............x85
.......................................................................GAGCUGGUUGUUGUAGAG...............x23
.......................................................................GAGCUGGUUGUUGUAGAGCCU............x6
........................................................................AGCUGGUUGUUGUAGAGCC.............x87
........................................................................AGCUGGUUGUUGUAGAGC..............x19
........................................................................AGCUGGUUGUUGUAGAGCCUU...........x5
........................................................................AGCUGGUUGUUGUAGAGCCU............x3
```

```
.........................................................................GCUGGUUGUUGUAGAGCC.............x74
...........................................................................UGGUUGUUGUAGAGCCUUUCC........x1
..............................................................................UUGUUGUAGAGCCUUUCCAG......x1
..............................................................................UUGUUGUAGAGCCUUUCCAGA.....x1
.................................................................................UUGUAGAGCCUUUCCAGAACC..x2
..................................................................................UGUAGAGCCUUUCCAGAACCA.x2
```

Most common read: UGAGCUGGUUGUUGUAGAGCC (freq 205866)

Complementarity: 18/21

Best mirbase match: NONE (e-value: NONE)

```
Sequence   1 Structure      1
 ENERGY =   -50.4    1
Bases    1 to  104

                 10        20        30        40
GC---------   A   C       G              U   G      A  A
           AAG CUC ACAACAA CAGCUCAAGGGAUA UUU GUUCAG AC \
           UUC GAG UGUUGUU GUCGAGUUCCCUAU AAA CGAGUC UG A
CACCAAGACCU   C   A       G              C   A      G  U
  100        90        80        70        60        50
```

Weak Candidate 21

```
1       10        20        30        40        50        60        70        80        90        100
UCUUCUACACAAGCGCUGCGUCGACGUCUCCUCCUCCUCCUCCUCCUCCUGCUGAAUCGGAGGGUGAGACGUAGCAGCAGCCGCUUGUGUCGAAGA
(((((.(((((((((((((....(((((((.((((((.....................)))))).))))))).....)))).))))))))).)))))

.....UACACAAGCGCUGCGUCGACG......................................................................x163*
.....UACACAAGCGCUGCGUCGACGU.....................................................................x20
.....UACACAAGCGCUGCGUCGAC.......................................................................x7
.....UACACAAGCGCUGCGUCGA........................................................................x6
.....UACACAAGCGCUGCGUCGACGUC...................................................................x3
......ACACAAGCGCUGCGUCGACG......................................................................x3
...........................CUCCUCCUCCUCCUCCUCC..................................................x1
..............................CUCCUCCUCCUCCUCCUCC...............................................x1
.........................................................CGGAGGGUGAGACGUAGCAGC..................x1
.......................................................................UAGCAGCAGCCGCUUGUGUCGA...x60*
.......................................................................UAGCAGCAGCCGCUUGUGUCGAA..x14
.......................................................................UAGCAGCAGCCGCUUGUGUC.....x1
```

Most common read: UACACAAGCGCUGCGUCGACG (freq 163)

Complementarity: 16/21

Best mirbase match: NONE (e-value: NONE)

```
Sequence   1 Structure      1
 ENERGY =   -48.7    1
Bases    1 to   96

        10         20        30        40
     U         -    GUCG       C      UCCUCCUCCU
```

```
UCUUC ACACAAGCG CUGC    ACGUCUC UCCUCC          \
AGAAG UGUGUUCGC GACG    UGCAGAG GGGAGG          C
     C         C    ACGA       U      CUAAGUCGUC
     90        80        70        60        50
```

Weak Candidate 22

```
1       10        20        30        40        50        60        70        80        90        100
CCAUCAUUUCUCAAGUUCCAUCGGAGGUUGAAUUCCAGCAACGCCGAGUCUUUGCUGGAGUUCAAACCUCCGGUGGAAUCUGAGGAUAGUGGCGA
((((.((((((.((((((((((((((((((((((((((((.(......).)))))))))))).))))))))))))))).))))))).))))...

...UCAUUUCUCAAGUUCCAUCGGA..........................................................................x7633*
...UCAUUUCUCAAGUUCCAUCGGAG.........................................................................x127
...UCAUUUCUCAAGUUCCAUCGG...........................................................................x89
...UCAUUUCUCAAGUUCCAUCG............................................................................x2
...UCAUUUCUCAAGUUCCAUC.............................................................................x2
....CAUUUCUCAAGUUCCAUCGGA..........................................................................x26
....CAUUUCUCAAGUUCCAUCGGAG.........................................................................x3
.....AUUUCUCAAGUUCCAUCGGA..........................................................................x16
.......................................ACGCCGAGUCUUUGCUGGAGUUC................................x1
...................................................................UCCGGUGGAAUCUGAGGAUAG......x39
...................................................................UCCGGUGGAAUCUGAGGAUA.......x2
...................................................................UCCGGUGGAAUCUGAGGAUAGUG....x1
....................................................................CCGGUGGAAUCUGAGGAUAGUG....x2
....................................................................CCGGUGGAAUCUGAGGAUAGU.....x1
....................................................................CCGGUGGAAUCUGAGGAUAG......x1
.....................................................................CGGUGGAAUCUGAGGAUAGUG....x345*
.....................................................................CGGUGGAAUCUGAGGAUAGU.....x43
.....................................................................CGGUGGAAUCUGAGGAUAG......x7
.....................................................................CGGUGGAAUCUGAGGAUAGUGGC..x2
......................................................................GGUGGAAUCUGAGGAUAGUG....x1
.......................................................................GUGGAAUCUGAGGAUAGUG....x1
........................................................................UGGAAUCUGAGGAUAGUGGCG.x2
```

Most common read: UCAUUUCUCAAGUUCCAUCGGA (freq 7633)

Complementarity: 19/21

Best mirbase match: esi-miR3455-5p (e-value: 0.001)

```
Query  1   UCAUUUCUCAAGUUCCAUCGGA  21
           ||||||||||||||||||||
Sbjct  1   UCAUUUCUCAAGUUCCAUCGGA  21


Sequence   1 Structure      1
 ENERGY =   -57.7    1
Bases    1 to   94

           10        20         30        40
---    C       A               -           C CC
   CCAU AUUCUCA GUUCCAUCGGAGGUU GAAUUCCAGCAA G  G
   GGUG UAGGAGU UAAGGUGGCCUCCAA CUUGAGGUCGUU C  A
AGC    A       C               A           U UG
   90        80        70        60        50
```

Weak Candidate 23

```
1       10        20        30        40        50        60        70        80        90        100
GCCGGAGAUGGACAGCGUUGUAGAGGUUCGGGGACGGCAUGGCCGUGUUUCUCGAUGACGUCCCCGAACCUAUGCAACGCUGUCUAUGUCGUGCGAUCGA
((..((.(((((((((((((.((((((((((((.(((.(..........).))).)))))))))))))).))))))))))))))).))..))......

.....AGAUGGACAGCGUUGUAGAGG..........................................................................x375*
.....AGAUGGACAGCGUUGUAGAG...........................................................................x55
.....AGAUGGACAGCGUUGUAG.............................................................................x14
.....AGAUGGACAGCGUUGUAGA............................................................................x11
.....AGAUGGACAGCGUUGUAGAGGU.........................................................................x2
........UGGACAGCGUUGUAGAGGUUC.......................................................................x1
................................................UUCUCGAUGACGUCCCCGAACC..............................x2
.................................................UCUCGAUGACGUCCCCGAACC..............................x2
.................................................UCUCGAUGACGUCCCCGAAC...............................x1
......................................................................UAUGCAACGCUGUCUAUGUCG.........x1113*
......................................................................UAUGCAACGCUGUCUAUGUC..........x80
......................................................................UAUGCAACGCUGUCUAUGU...........x14
......................................................................UAUGCAACGCUGUCUAUGUCGU........x1
.......................................................................AUGCAACGCUGUCUAUGUCG.........x3
```

Most common read: UAUGCAACGCUGUCUAUGUCG (freq 1113)

Complementarity: 18/21

Best mirbase match: cel-miR-794-3p (e-value: 4.8)

```
Query  6   AACGCUGUCUAUGUCG  21
           |||| ||||||| |||
Sbjct  5   AACGUUGUCUAUCUCG  20
```

```
Sequence   1 Structure      1
0 ENERGY =   -61.9    1
Bases    1 to  100

              10        20        30        40
------  CG  G               G             G   G CCGU
      GC  GA AUGGACAGCGUUGUA AGGUUCGGGGACG CAU G    G
      CG  CU UAUCUGUCGCAACGU UCCAAGCCCCUGC GUA C    U
AGCUAG  UG  G               A             A   G UCUU
.        90        80        70        60        50
```

Weak Candidate 24

```
1       10        20        30        40        50        60        70        80        90        100
GGGGUAAAUAUCGGAGCUCACCUUACAUCCCUUGGUAAAUGAAGUGCUGUAGUCUAUUUACCAAGGGAUGUCAGGUGAGCUCCGGGAAUUGCCUGAUAGCG
.((((((...((((((((((((.(((((((((((((((((.(.((...)).).))))))))))))))))).))))))))))))...)))))).......

....UAAAUAUCGGAGCUCACCUUA............................................................................x6*
......AAUAUCGGAGCUCACCUUACA..........................................................................x3
......AAUAUCGGAGCUCACCUUAC...........................................................................x1
......................................................................UCAGGUGAGCUCCGGGAAUUG..........x471*
......................................................................UCAGGUGAGCUCCGGGAAUU...........x8
......................................................................UCAGGUGAGCUCCGGGAAU............x4
......................................................................UCAGGUGAGCUCCGGGAAUUGC.........x1
.......................................................................CAGGUGAGCUCCGGGAAUUG..........x1
........................................................................AGGUGAGCUCCGGGAAUUG..........x2
```

Most common read: UCAGGUGAGCUCCGGGAAUUG (freq 471)

Complementarity: 17/21

Best mirbase match: ppt-miR1032 (e-value: 8.6)

```
Query  3   AGGUGAGCUCCGGGAAUUG  21
           ||||||   || |||||||
Sbjct  1   AGGUGACUGCCUGGAAUUG  19
```

```
Sequence   1 Structure      1
 ENERGY =   -59.2    1
Bases    1 to  101

              10        20        30        40
G------      AUA             U                 A G  C
       GGGUAA   UCGGAGCUCACCU ACAUCCCUUGGUAAAUG A UG \
       UCCGUU   GGCCUCGAGUGGA UGUAGGGAACCAUUUAU U AU U
GCGAUAG      AAG             C                 C G  G
 .        90        80        70        60        50
```

Weak Candidate 25

```
1       10         20         30         40         50         60         70         80         90         100
GUUUGUGUGCUCGGCAGCUUCUCGUGGCACACUUGGAGGAUAUCCGUAUGUAUCUCGACGGAUGCCCUUCCAAGUGUUCCACGAGAAAUUUCCUAGCAUAC
....(((((((.((.((.(((((((((.(((((((((((.(((((((.((.....))))))))).)).))))))))).))))))))).)).)).)))))))

.........CUCGGCAGCUUCUCGUGGCACA......................................................................x4
.........CUCGGCAGCUUCUCGUGGCAC.......................................................................x1
..........UCGGCAGCUUCUCGUGGCACA......................................................................x16604*
..........UCGGCAGCUUCUCGUGGCAC.......................................................................x1045
..........UCGGCAGCUUCUCGUGGCA........................................................................x103
..........UCGGCAGCUUCUCGUGGC.........................................................................x12
..........UCGGCAGCUUCUCGUGGCACAC.....................................................................x9
...........CGGCAGCUUCUCGUGGCACA......................................................................x38
...........CGGCAGCUUCUCGUGGCAC.......................................................................x7
...........CGGCAGCUUCUCGUGGCACACU....................................................................x1
............GGCAGCUUCUCGUGGCACA......................................................................x10
............GGCAGCUUCUCGUGGCAC.......................................................................x1
.............GCAGCUUCUCGUGGCACA......................................................................x1
.......................GUGGCACACUUGGAGGAUA...........................................................x1
..............................................UAUGUAUCUCGACGGAUGCCCU.................................x2
..........................................................................UGUUCCACGAGAAAUUUCCUAG.....x1
............................................................................UUCCACGAGAAAUUUCCUAGC....x262*
............................................................................UUCCACGAGAAAUUUCCUAGCA...x10
............................................................................UUCCACGAGAAAUUUCCUAG.....x3
.............................................................................UCCACGAGAAAUUUCCUAGCA...x16
.............................................................................UCCACGAGAAAUUUCCUAGC....x2
```

Most common read: UCGGCAGCUUCUCGUGGCACA (freq 16604)

Complementarity: 17/21

Best mirbase match: cbn-miR-49 (e-value: 0.33)

```
Query  1   UCGGCAGCUUCUCGUGG  17
           || ||||||||||||||
Sbjct  22  UCUGCAGCUUCUCGUGG  6
```

```
Sequence   1 Structure      1
 ENERGY =   -53.4    1
Bases    1 to  101
```

```
        10        20        30         40        50
GUUU       C  C  C         C         -  A       A  UA
    GUGUGCU GG AG UUCUCGUGG ACACUUGGA GG UAUCCGU UG  \
    CAUACGA CC UU AAGAGCACC UGUGAACCU CC GUAGGCA GC  U
----       U  U  A         U         U  C       -  UC
   100        90        80        70        60
```

Weak Candidate 26

```
1        10        20        30        40        50        60        70        80        90        100
CCGUACUCCGACUGGGUGUACCAGUCCGGUUAAGGUGGUCGGGCUUACCGAUUACCUCAACCGGACUGGUACACCCAGCCGGAGUUCGAACCGG
.((.((((((.((((((((((((((((((((.((((((((((.....)))))))))).)))))))))))))))))))).)))))).))......

...UACUCCGACUGGGUGUACCAG......................................................................x159*
...UACUCCGACUGGGUGUACCA.......................................................................x118
...UACUCCGACUGGGUGUACC........................................................................x14
...UACUCCGACUGGGUGUACCAGU.....................................................................x1
....ACUCCGACUGGGUGUACCA.......................................................................x1
........................UCCGGUUAAGGUGGUCGG....................................................x1
........................UCCGGUUAAGGUGGUCGGGCU.................................................x1
..................................................................UGGUACACCCAGCCGGAGUUC.......x41
..................................................................UGGUACACCCAGCCGGAGUU........x3
..................................................................UGGUACACCCAGCCGGAGU.........x1
..................................................................UGGUACACCCAGCCGGAGUUCG......x1
...................................................................GGUACACCCAGCCGGAGUUCG......x46*
...................................................................GGUACACCCAGCCGGAGUUC.......x40
...................................................................GGUACACCCAGCCGGAGUU........x2
...................................................................GGUACACCCAGCCGGAGUUCGA.....x1
.....................................................................UACACCCAGCCGGAGUUCGAA....x44
.....................................................................UACACCCAGCCGGAGUUCGA.....x9
.....................................................................UACACCCAGCCGGAGUUCGAAC...x1
```

Most common read: UACUCCGACUGGGUGUACCAG (freq 159)

Complementarity: 19/21

Best mirbase match: hvt-miR-H4-3p (e-value: 3.3)

```
Query  1   UACUCCGACUGGGUGUACCA  20
           ||||||||| | | || |||
Sbjct  26  UACUCCGACGGCGGGUGCCA  7
```

```
Sequence   1 Structure      1
 ENERGY =   -72.6    1
Bases    1 to   94

             10        20        30        40
C-----  U      A                    A          GC
      CG ACUCCG CUGGGUGUACCAGUCCGGUU AGGUGGUCGG  \
      GC UGAGGC GACCCACAUGGUCAGGCCAA UCCAUUAGCC  U
GGCCAA  U      C                    C          AU
   90        80        70        60        50
```

Weak Candidate 27

```
1       10        20        30        40        50        60        70        80        90        100
CCUCGAUUGUUGUCUUAGCCGUUCUCUUGUCCUCCCGCUCCCUCUCCUCCUCCUCAUUUGGAGGGAACGGGAGGACAAGGGAGCGAUCAAGACAGCAAUCG
...(((((((((((((...((((((((((((((((((.(((((((..............))))))).))))))))))))))))))...)))))))))))))

..UCGAUUGUUGUCUUAGCCGUU..............................................................................x1
.........UUGUCUUAGCCGUUCUCUUGU.......................................................................x1
..........UGUCUUAGCCGUUCUCUUGUC......................................................................x141*
..........UGUCUUAGCCGUUCUCUUGU.......................................................................x25
..........UGUCUUAGCCGUUCUCUUGUCC.....................................................................x1
...........GUCUUAGCCGUUCUCUUGUC......................................................................x2
...........................................................................CAAGGGAGCGAUCAAGACAGC.....x3*
...........................................................................CAAGGGAGCGAUCAAGACAGCA....x2
```

Most common read: UGUCUUAGCCGUUCUCUUGUC (freq 141)

Complementarity: 18/21

Best mirbase match: mml-miR-3122 (e-value: 2.7)

```
Query  9   CCGUUCUCUUGUC  21
           |||||||||||||
Sbjct  18  CCGUUCUCUUGUC  6
```

```
Sequence   1 Structure      1
 ENERGY =   -64.5    1
Bases    1 to  101

        10        20        30        40        50
CCU             AGC                  C       CCUCCU
   CGAUUGUUGUCUU   CGUUCUCUUGUCCUCCCG UCCCUCU      C
   GCUAACGACAGAA   GCGAGGGAACAGGAGGGC AGGGAGG      C
---             CUA                  A       UUUACU
  100        90        80        70        60
```

Weak Candidate 28

```
1       10        20        30        40        50        60        70        80        90        100
GAGGAUCCUCUUGAAGAAGCUCUUGAACCAUCACAACGAACUCGUCGUCUCAGAGCGCGUUUGUGGUGGUUCCAGAGUGGCUUCGAGUGGAUCCU
.(((((((.(((((((..(((((.((((((((((((((..(((.........)))..)).)))))))))))).)))))..))))))).)))))))

....AUCCUCUUGAAGAAGCUCUUG......................................................................x1
....AUCCUCUUGAAGAAGCUCUUGA.....................................................................x1
.....UCCUCUUGAAGAAGCUCUUGA.....................................................................x3396*
.....UCCUCUUGAAGAAGCUCUUG......................................................................x76
.....UCCUCUUGAAGAAGCUCUUGAA....................................................................x72
.....UCCUCUUGAAGAAGCUCUU.......................................................................x2
.......CUCUUGAAGAAGCUCUUGA.....................................................................x10
.......CUCUUGAAGAAGCUCUUG......................................................................x2
.......CUCUUGAAGAAGCUCUUGAA....................................................................x1
........UCUUGAAGAAGCUCUUGA.....................................................................x3
.....................................................................UUCCAGAGUGGCUUCGAGUGG.....x2
......................................................................UCCAGAGUGGCUUCGAGUGGA....x9
........................................................................CAGAGUGGCUUCGAGUGGAUC..x20*
........................................................................CAGAGUGGCUUCGAGUGGAU...x3
........................................................................CAGAGUGGCUUCGAGUGGAUCC.x2
.........................................................................AGAGUGGCUUCGAGUGGAUCC.x2
```

Most common read: UCCUCUUGAAGAAGCUCUUGA (freq 3396)

Complementarity: 17/21

```
Best mirbase match: gga-miR-6605-5p (e-value: 4.0)

Query  1   UCCUCUUGAAGAAGCUCU  18
           || | || ||||||||||
Sbjct  23  UCUUGUUCAAGAAGCUCU  6


Sequence   1 Structure      1
 ENERGY =   -55.5    1
Bases    1 to   95

        10        20        30         40
G       U       AA     U            -  AA   GUCG
 AGGAUCC CUUGAAG  GCUCU GAACCAUCACAA CG  CUC    \
 UCCUAGG GAGCUUC  UGAGA CUUGGUGGUGUU GC  GAG    U
-       U       GG     C            U  GC   ACUC
     90        80        70        60         50
```

```
Weak Candidate 29

1       10        20        30        40        50        60        70        80        90        100
AAGCAUGGCAAGCAGGGAUGGUUCACGUGUUCAGCGAAAGACACGUGCUGGAUGUCUCUCGCUGAACACGUCAACCAUACCCUGCUUGGCGCGUUUCGAG
((((.((.(((((((((((((((.(((((((((((((.((((((......).))))).))))))))))))).)))))).))))))))).)).))))....

.....UGGCAAGCAGGGAUGGUUCAC..........................................................................x3*
.....UGGCAAGCAGGGAUGGUUCA...........................................................................x1
......GGCAAGCAGGGAUGGUUCACG.........................................................................x1
.......GCAAGCAGGGAUGGUUCACG.........................................................................x2
...........................UGUUCAGCGAAAGACACGUGCU...................................................x2
..............................................GCUGGAUGUCUCUCGCUGAACACG..............................x1
...............................................CUGGAUGUCUCUCGCUGAACACG..............................x1
................................................UGGAUGUCUCUCGCUGAACACG..............................x5
................................................UGGAUGUCUCUCGCUGAACA................................x1
....................................................UGUCUCUCGCUGAACACG..............................x1
......................................................................UCAACCAUACCCUGCUUGGCG.........x248*
......................................................................UCAACCAUACCCUGCUUGGC..........x147
......................................................................UCAACCAUACCCUGCUUGG...........x25
......................................................................UCAACCAUACCCUGCUUGGCGCGUU.....x4
......................................................................UCAACCAUACCCUGCUUGGCGC........x3
......................................................................UCAACCAUACCCUGCUUGGCGCGU......x1

Most common read: UCAACCAUACCCUGCUUGGCG (freq 248)

Complementarity: 18/21

Best mirbase match: hsa-miR-6731-5p (e-value: 4.8)

Query  1   UCAACCAUACCCUGCU  16
           || || ||||||||||
Sbjct  22  UCCACAAUACCCUGCU  7


Sequence   1 Structure      1
0 ENERGY =   -61.9    1
Bases    1 to  100

            10         20        30         40
----    A  G         -      C             A     - GU
    AAGC UG CAAGCAGGG AUGGUU ACGUGUUCAGCGA AGACA C  G
    UUUG GC GUUCGUCCC UACCAA UGCACAAGUCGCU UCUGU G  C
GAGC    C  G         A      C             C     A GU
```

```
.        90        80        70        60        50
```

___

Weak Candidate 30

```
1       10         20         30         40         50         60         70         80         90         100
GAACAUCAUCAUCUCUUCGAGUGACGAGUCGCUGUCAGUCACUGACAGCCGUCGUCAGCGACUGACAGCGACUCAUAACUCGAAGAGAUGAUGAUGUUC
((((((((((((((((((((...((((((((((((((.(((((.......))))).)))))))))))))))...)))))))))))))))))))))

........UCAUCUCUUCGAGUGACGAGU.......................................................................x1522*
........UCAUCUCUUCGAGUGACGAGUC......................................................................x4
........UCAUCUCUUCGAGUGACGAG........................................................................x4
........UCAUCUCUUCGAGUGACGA.........................................................................x2
.........CAUCUCUUCGAGUGACGAGU.......................................................................x2
..........AUCUCUUCGAGUGACGAGU.......................................................................x1
...........UCUCUUCGAGUGACGAGUCG.....................................................................x1
...............UUCGAGUGACGAGUCGCUGUC................................................................x1
......................................................................ACUCAUAACUCGAAGAGAUGA........x2
........................................................................UCAUAACUCGAAGAGAUGAUG......x2518*
........................................................................UCAUAACUCGAAGAGAUGAUGA.....x89
........................................................................UCAUAACUCGAAGAGAUGAU.......x7
........................................................................UCAUAACUCGAAGAGAUGA........x7
........................................................................UCAUAACUCGAAGAGAUG.........x2
.........................................................................CAUAACUCGAAGAGAUGAUG......x3
.........................................................................CAUAACUCGAAGAGAUGAUGA.....x3
..........................................................................AUAACUCGAAGAGAUGAUG......x4
...........................................................................UAACUCGAAGAGAUGAUG......x1
```

Most common read: UCAUAACUCGAAGAGAUGAUG (freq 2518)

Complementarity: 18/21

Best mirbase match: NONE (e-value: NONE)

```
Sequence   1 Structure      1
 ENERGY =   -76.8    1
Bases    1 to   99

        10        20        30        40
                      GAC              A     AGC
GAACAUCAUCAUCUCUUCGAGU   GAGUCGCUGUCAGUC CUGAC   \
CUUGUAGUAGUAGAGAAGCUCA   CUCAGCGACAGUCAG GACUG   C
                      AUA              C     CUG
        90        80        70        60
```

___

Weak Candidate 31

```
1       10         20         30         40         50         60         70         80         90         100
UUCUCCAGUUGACUCUUCAACAACCCCGCCUCUGCCUCGCUUUCGACGAAGGUUUUCCGAAUGCCAGGCAGAGGCGUGGUCGUUGAAGAGUGAACUGGA
...((((((.(((((((((.(((.((((((((((.((.((((..(((....)))))))).)).))))))))))).))).)))))))))).)))))))

.....CAGUUGACUCUUCAACAACCCCGCC......................................................................x1
........UUGACUCUUCAACAACCCCGCC......................................................................x57
........UUGACUCUUCAACAACCCCGC.......................................................................x39
........UUGACUCUUCAACAACCCCGCCU.....................................................................x7
........UUGACUCUUCAACAACCCCG........................................................................x1
.........UGACUCUUCAACAACCCCGCC......................................................................x54279*
.........UGACUCUUCAACAACCCCGC.......................................................................x10536
.........UGACUCUUCAACAACCCCGCCU.....................................................................x2072
```

```
.........UGACUCUUCAACAACCCCG.......................................................................x527
.........UGACUCUUCAACAACCCC........................................................................x42
.........UGACUCUUCAACAACCCCGCCUC...................................................................x2
.........UGACUCUUCAACAACCCCGCCUCU..................................................................x1
..........GACUCUUCAACAACCCCGCC.....................................................................x136
..........GACUCUUCAACAACCCCGCCU....................................................................x100
..........GACUCUUCAACAACCCCGC......................................................................x15
..........GACUCUUCAACAACCCCG.......................................................................x1
...........ACUCUUCAACAACCCCGCC.....................................................................x8
...........ACUCUUCAACAACCCCGCCU....................................................................x2
...........ACUCUUCAACAACCCCGC......................................................................x1
.............UCUUCAACAACCCCGCCU....................................................................x3
..............................UCUGCCUCGCUUUCGACGA..................................................x4
..............................UCUGCCUCGCUUUCGACGAA.................................................x3
..................................................................GGCAGAGGCGUGGUCGUUGA.............x1
.........................................................................GCGUGGUCGUUGAAGAGUGA......x204
.........................................................................GCGUGGUCGUUGAAGAGUGAA.....x85
.........................................................................GCGUGGUCGUUGAAGAGUG.......x19
.........................................................................GCGUGGUCGUUGAAGAGUGAAC....x2
..........................................................................CGUGGUCGUUGAAGAGUGAAC....x1676*
..........................................................................CGUGGUCGUUGAAGAGUGAA.....x419
..........................................................................CGUGGUCGUUGAAGAGUGA......x280
..........................................................................CGUGGUCGUUGAAGAGUGAACU...x13
..........................................................................CGUGGUCGUUGAAGAGUG.......x9
...........................................................................GUGGUCGUUGAAGAGUGAAC....x12
...........................................................................GUGGUCGUUGAAGAGUGA......x4
...........................................................................GUGGUCGUUGAAGAGUGAA.....x2
............................................................................UGGUCGUUGAAGAGUGAACUG..x3
............................................................................UGGUCGUUGAAGAGUGAACUGG.x1
```

Most common read: UGACUCUUCAACAACCCCGCC (freq 54279)

Complementarity: 18/21

Best mirbase match: ptr-miR-649 (e-value: 4.0)

```
Query  2   GACUCUUCAACAACCCCG  19
           ||||||| |||||| | |
Sbjct  22  GACUCUUGAACAACACAG  5


Sequence   1 Structure      1
 ENERGY =   -63.6    1
Bases    1 to   99

        10        20        30        40        50
UUC       G          A   C           C  U    AC   G
   UCCAGUU ACUCUUCAAC ACC CGCCUCUGCCU GC UUCG  GAA G
   AGGUCAA UGAGAAGUUG UGG GCGGAGACGGA CG AAGC  CUU U
---       G          C   U           C  U    --   U
           90        80        70        60
```

Weak Candidate 32

```
1       10        20        30        40        50        60        70        80        90        100
CUCUCCAGUUGGCUCUUCAACAACUUCGCCUCUGCCUCGCUUUCGUCAAUGUUUCCGAAAGCAAGGCAGAGGCGUAGCUGUUGGAGAACCACGUGGAGA
.((((((..(((.(((((((((.((.(((((((((((.(((((((.........))))))).))))))))))).)).))))))))).)))..))))))

.......GUUGGCUCUUCAACAACUUCG.......................................................................x1
........UUGGCUCUUCAACAACUUCGC......................................................................x228
........UUGGCUCUUCAACAACUUCGCC.....................................................................x58
........UUGGCUCUUCAACAACUUCG.......................................................................x3
```

```
.........UGGCUCUUCAACAACUUCGCC.....................................................................x15267*
.........UGGCUCUUCAACAACUUCGC......................................................................x1136
.........UGGCUCUUCAACAACUUCG.......................................................................x17
.........UGGCUCUUCAACAACUUCGCCU....................................................................x16
.........UGGCUCUUCAACAACUUC........................................................................x9
..........GGCUCUUCAACAACUUCGCC.....................................................................x86
..........GGCUCUUCAACAACUUCGC......................................................................x11
...........GCUCUUCAACAACUUCGCC.....................................................................x1
..............................UCUGCCUCGCUUUCGUCAAU.................................................x96
..............................UCUGCCUCGCUUUCGUCAAUG................................................x9
..............................UCUGCCUCGCUUUCGUCAA..................................................x8
..............................UCUGCCUCGCUUUCGUCAAUGUUU.............................................x1
................................UGCCUCGCUUUCGUCAAU.................................................x1
...................................................UUUCCGAAAGCAAGGCAGAGG...........................x4
....................................................UUCCGAAAGCAAGGCAGAGG...........................x1
........................................................................CGUAGCUGUUGGAGAACCACG......x305*
........................................................................CGUAGCUGUUGGAGAACCAC.......x14
........................................................................CGUAGCUGUUGGAGAACC.........x3
........................................................................CGUAGCUGUUGGAGAACCA........x1
.........................................................................GUAGCUGUUGGAGAACCACG......x6
.........................................................................GUAGCUGUUGGAGAACCAC.......x1
..........................................................................UAGCUGUUGGAGAACCACGUGG...x143
..........................................................................UAGCUGUUGGAGAACCACGUG....x103
..........................................................................UAGCUGUUGGAGAACCACGU.....x4
..........................................................................UAGCUGUUGGAGAACCACGUGGA..x1
...........................................................................AGCUGUUGGAGAACCACGUG....x1
...........................................................................AGCUGUUGGAGAACCACGUGG...x1
```

Most common read: UGGCUCUUCAACAACUUCGCC (freq 15267)

Complementarity: 18/21

Best mirbase match: NONE (e-value: NONE)

```
Sequence   1 Structure      1
 ENERGY =   -61.8    1
Bases    1 to   99

        10        20        30        40        50
C      GU   C         A  U           C       UCAA
 UCUCCA  UGG UCUUCAACA CU CGCCUCUGCCU GCUUUCG    U
 AGAGGU  ACC AGAGGUUGU GA GCGGAGACGGA CGAAAGC    G
-      GC   A         C  U           A       CUUU
         90        80        70        60
```

Weak Candidate 33

```
1        10        20        30        40        50        60        70        80        90        100
CUCUCCAGUUGGCUCUUCAACAACUUCGCCUCUGCCUCGCUUUCGUCAAUGUUUCCGAAAGCAAGGCAGAGGCGUGGCUGUUGAAGAACCAAGUGGAAA
...((((.((((.(((((((((.((.(((((((((((.(((((((..........))))))).))))))))))).)).))))))))).)))).))))..

.......GUUGGCUCUUCAACAACUUCG.......................................................................x1
........UUGGCUCUUCAACAACUUCGC......................................................................x228
........UUGGCUCUUCAACAACUUCGCC.....................................................................x58
........UUGGCUCUUCAACAACUUCG.......................................................................x3
.........UGGCUCUUCAACAACUUCGCC.....................................................................x15267*
.........UGGCUCUUCAACAACUUCGC......................................................................x1136
.........UGGCUCUUCAACAACUUCG.......................................................................x17
```

```
.........UGGCUCUUCAACAACUUCGCCU....................................................................x16
.........UGGCUCUUCAACAACUUC........................................................................x9
..........GGCUCUUCAACAACUUCGCC.....................................................................x86
..........GGCUCUUCAACAACUUCGC......................................................................x11
...........GCUCUUCAACAACUUCGCC.....................................................................x1
..............................UCUGCCUCGCUUUCGUCAAU.................................................x96
..............................UCUGCCUCGCUUUCGUCAAUG................................................x9
..............................UCUGCCUCGCUUUCGUCAA..................................................x8
..............................UCUGCCUCGCUUUCGUCAAUGUUU.............................................x1
................................UGCCUCGCUUUCGUCAAU.................................................x1
...................................................UUUCCGAAAGCAAGGCAGAGG...........................x4
....................................................UUCCGAAAGCAAGGCAGAGG...........................x1
.......................................................................GCGUGGCUGUUGAAGAACC.........x1
........................................................................CGUGGCUGUUGAAGAACCAAG......x713*
........................................................................CGUGGCUGUUGAAGAACCAA.......x20
........................................................................CGUGGCUGUUGAAGAACC.........x5
........................................................................CGUGGCUGUUGAAGAACCA........x1
.........................................................................GUGGCUGUUGAAGAACCAAG......x6
.........................................................................GUGGCUGUUGAAGAACCAAGU.....x4
..........................................................................UGGCUGUUGAAGAACCAAGUGG...x334
..........................................................................UGGCUGUUGAAGAACCAAGUG....x296
..........................................................................UGGCUGUUGAAGAACCAAGUGGA..x52
..........................................................................UGGCUGUUGAAGAACCAAGU.....x7
..........................................................................UGGCUGUUGAAGAACCAAGUGGAA.x5
..........................................................................UGGCUGUUGAAGAACCAAG......x3
...........................................................................GGCUGUUGAAGAACCAAGUGG...x1
```

Most common read: UGGCUCUUCAACAACUUCGCC (freq 15267)

Complementarity: 18/21

Best mirbase match: NONE (e-value: NONE)

```
Sequence   1 Structure      1
 ENERGY =   -61.1    1
Bases    1 to   99

        10        20        30        40        50
CUC    G    C         A  U           C       UCAA
   UCCA UUGG UCUUCAACA CU CGCCUCUGCCU GCUUUCG    U
   AGGU AACC AGAAGUUGU GG GCGGAGACGGA CGAAAGC    G
AA-    G    A         C  U           A       CUUU
         90        80        70        60
```

Weak Candidate 34

```
1       10        20        30        40        50        60        70        80        90        100
GGCAUCAGGAACAAGGUCAGCUGCACCGUAGCGAAUGUCGGAGAAGCCGUGCUCAUUCGUUGCGGUGCAGCUGACCUCGUACCGGAUGCCGUAGUCUUCA
((((((.((.((.((((((((((((((((((((((((.(((.....)))....)))))))))))))))))))))))).)).)).))))))..........

...AUCAGGAACAAGGUCAGCUGC............................................................................x42*
...AUCAGGAACAAGGUCAGCUGCA...........................................................................x3
....UCAGGAACAAGGUCAGCUGC............................................................................x19
....UCAGGAACAAGGUCAGCUGCA...........................................................................x10
.................................................................UGCAGCUGACCUCGUACCGGA..............x2
....................................................................AGCUGACCUCGUACCGGAUGC...........x75*
....................................................................AGCUGACCUCGUACCGGAUG............x11
....................................................................AGCUGACCUCGUACCGGAU.............x2
```

```
....................................................................AGCUGACCUCGUACCGGA..............x2
```

Most common read: AGCUGACCUCGUACCGGAUGC (freq 75)

Complementarity: 18/21

Best mirbase match: NONE (e-value: NONE)

```
Sequence   1 Structure      1
0 ENERGY =   -64.4    1
Bases    1 to  100

                  10        20        30           40
----------      A  A  A                        U---   AG
          GGCAUC GG AC AGGUCAGCUGCACCGUAGCGAAUG    CGG  \
          CCGUAG CC UG UCCAGUCGACGUGGCGUUGCUUAC    GCC  A
ACUUCUGAUG      G  A  C                        UCGU   GA
.        90        80        70        60        50
```

Weak Candidate 35

```
1       10        20        30        40        50        60        70        80        90        100
CCCGUUGGUCCCAAGCACGGAGGUGGGGGCUUCGAAGGAGAUCGACGAUGCCACGACCGUCUCCUUCGAAACCCCCACUUCCGUGCCUAGGACCAAUGG
.((((((((((...(((((((((((((((.((((((((((((.(.((......)).).)))))))))))).)))))))))))))))...))))))))))

........UCCCAAGCACGGAGGUGGGGG......................................................................x13*
........UCCCAAGCACGGAGGUGGGGGC.....................................................................x1
.........................................................................CCCACUUCCGUGCCUAGGACC.....x1*
```

Most common read: UCCCAAGCACGGAGGUGGGGG (freq 13)

Complementarity: 18/21

Best mirbase match: hsa-miR-6808-5p (e-value: 4.8)

```
Query  4   CAAGCACGGAGGUGGG  19
           || ||| |||||||||
Sbjct  1   CAGGCAGGGAGGUGGG  16
```

```
Sequence   1 Structure      1
 ENERGY =   -71.7    1
Bases    1 to   99

        10        20        30        40        50
C          CAA              C            C A  AU
 CCGUUGGUCC   GCACGGAGGUGGGGG UUCGAAGGAGAU G CG  G
 GGUAACCAGG   CGUGCCUUCACCCCC AAGCUUCCUCUG C GC  C
-          AUC              A            C A  AC
         90        80        70        60
```

Weak Candidate 36

```
1       10        20        30        40        50        60        70        80        90        100
UUGUGCCCCCUCCGGGGUGAGCGCAGCGCUUGUUCGUUACGUCCAUAAUGGACGUGACGAACAAGCGCUUCGGUCACACCGGAGGGUGCACGAUUCACC
((((((.((((((((.((((.((.(((((((((((((((((((((...))))))))))))))))))))).)).)))).)))))))).))))))......
```

```
.....CCCCCUCCGGGGUGAGCGCAG.........................................................................x11*
..........................................CCAUAAUGGACGUGACGAA......................................x1
.....................................................................UCGGUCACACCGGAGGGUGCA.........x66*
.....................................................................UCGGUCACACCGGAGGGUGC..........x3
```

Most common read: UCGGUCACACCGGAGGGUGCA (freq 66)

Complementarity: 17/21

Best mirbase match: NONE (e-value: NONE)

```
Sequence   1 Structure      1
 ENERGY =   -75.8    1
Bases    1 to   99

              10        20        30        40
------      C        G    G  C                     U
      UUGUGC CCCUCCGG GUGA CG AGCGCUUGUUCGUUACGUCCA \
      AGCACG GGGAGGCC CACU GC UCGCGAACAAGCAGUGCAGGU A
CCACUU      U        A    G  U                     A
        90        80        70        60        50
```

Weak Candidate 37

```
1       10        20        30        40        50        60        70        80        90        100
GUUCGGGUGGGGGCUGUUGGCCAGGCUGGGGAGGGGGGGGGGGUUACUCAUGUAAAUGCACCCCUCCGCCCCAGCCUUGCGAACAAUCCUCACCCGAA
.((((((((((((.((((.((.(((((((((.((((((((.(.((((....)))).).).))))))).))))))))).)).)))).))))))))))))

.......UGGGGGCUGUUGGCCAGGCUG......................................................................x1184*
.......UGGGGGCUGUUGGCCAGGCU.......................................................................x236
.......UGGGGGCUGUUGGCCAGGCUGG.....................................................................x79
.......UGGGGGCUGUUGGCCAGG.........................................................................x8
.......UGGGGGCUGUUGGCCAGGC........................................................................x3
........GGGGGCUGUUGGCCAGGCU.......................................................................x2
........GGGGGCUGUUGGCCAGGCUG......................................................................x2
........................................................................AGCCUUGCGAACAAUCCUCACC....x12
.........................................................................GCCUUGCGAACAAUCCUCACC....x43*
.........................................................................GCCUUGCGAACAAUCCUCAC.....x4
.........................................................................GCCUUGCGAACAAUCCUCA......x1
```

Most common read: UGGGGGCUGUUGGCCAGGCUG (freq 1184)

Complementarity: 18/21

Best mirbase match: cgr-miR-1285 (e-value: 5.8)

```
Query  8   UGUUGGCCAGGCUG  21
           ||||| ||||||||
Sbjct  21  UGUUGCCCAGGCUG  8
```

```
Sequence   1 Structure      1
 ENERGY =   -71.5    1
Bases    1 to   98

        10        20        30         40
G            C    G  C         A       - G G    U
```

```
 UUCGGGUGGGGG UGUU GC AGGCUGGGG GGGGGGG G G UUAC C
 AAGCCCACUCCU ACAA CG UCCGACCCC CCUCCCC C U AAUG A
-            A    G  U         G       A G A    U
        90        80        70        60        50
```

Weak Candidate 38

```
1       10        20        30        40        50        60        70        80        90        100
UAGCUCCGGCGGCGGAGGUGGCACUCACCGCAGCGCACGCCGGCGAUGUCGGAGUUGAGUUUCGGUGACUGCCGCCUCCUCUGACUUAGCUACCUUA
(((((..(((((.((((((((((.((((((.(((.(((.(((((...))))).).)).))).)))))).)))))))))).))).)).))))).....

....UCCGGCGGCGGAGGUGGCACU........................................................................x27*
......CGGCGGCGGAGGUGGCACU........................................................................x1
.....................................................................UGCCGCCUCCUCUGACUUAGC.......x27*
.....................................................................UGCCGCCUCCUCUGACUUAG........x2
.....................................................................UGCCGCCUCCUCUGACUUAGCU......x2
```

Most common read: UCCGGCGGCGGAGGUGGCACU (freq 27)

Complementarity: 17/21

Best mirbase match: hsa-miR-6727-3p (e-value: 1.8)

```
Query  3   CGGCGGCGGAGGUGGCA  19
           | |||| ||||||||||
Sbjct  20  CUGCGGAGGAGGUGGCA  4
```

```
Sequence   1 Structure      1
 ENERGY =   -51.2    1
Bases    1 to   97

              10        20        30         40
-----     CC  -   C          C      C   G  - G     G
     UAGCU  GG CGG GGAGGUGGCA UCACCG AGC CA C CCGGC \
     AUCGA  UC GUC CCUCCGCCGU AGUGGC UUG GU G GGCUG A
AUUCC     U-  A   U          C      U   A  U A     U
      90         80        70        60        50
```

Weak Candidate 39

```
1       10        20        30        40        50        60        70        80        90        100
GUCCACGUGGAUGAGAAGUGGUUCUAUCUCUUCAAGGAUAGGCAGAAGUCUUCCGCCCGUCCUUCAAGAGACAGAACCAUUUAUCGUCCACGUGGACG
((((((((((((((.((((((((((.((((((.((((((.(((.(((...))).))).)))))).)))))).)))))))))).)))))))))))))).

.......UGGAUGAGAAGUGGUUCUAUC......................................................................x2170*
.......UGGAUGAGAAGUGGUUCUAU.......................................................................x1066
.......UGGAUGAGAAGUGGUUCUA........................................................................x93
.......UGGAUGAGAAGUGGUUCU.........................................................................x50
.......UGGAUGAGAAGUGGUUCUAUCU.....................................................................x24
........GGAUGAGAAGUGGUUCUAU.......................................................................x5
........GGAUGAGAAGUGGUUCUAUC......................................................................x3
.........GAUGAGAAGUGGUUCUAUC......................................................................x1
............................UCUUCAAGGAUAGGCAGA....................................................x1
.............................................AAGUCUUCCGCCCGUCCUUCAAGAGA...........................x1
................................................UCUUCCGCCCGUCCUUCAAGA.............................x2
.................................................CUUCCGCCCGUCCUUCAAGAGA...........................x1
...................................................UCCGCCCGUCCUUCAAGAGA...........................x1
```

```
.......................................................................CAGAACCAUUUAUCGUCCACG......x2074*
.......................................................................CAGAACCAUUUAUCGUCCAC.......x82
.......................................................................CAGAACCAUUUAUCGUCCACGU.....x14
.......................................................................CAGAACCAUUUAUCGUCCA........x13
.......................................................................CAGAACCAUUUAUCGUCC.........x2
........................................................................AGAACCAUUUAUCGUCCACG......x28
```

Most common read: UGGAUGAGAAGUGGUUCUAUC (freq 2170)

Complementarity: 19/21

Best mirbase match: NONE (e-value: NONE)

```
Sequence   1 Structure      1
 ENERGY =   -63.7    1
Bases    1 to   98

         10        20        30        40
-              G          A      C      A   A   G
 GUCCACGUGGAUGA AAGUGGUUCU UCUCUU AAGGAU GGC GAA \
 CAGGUGCACCUGCU UUUACCAAGA AGAGAA UUCCUG CCG CUU U
G              A          C      C      C   C   C
       90        80        70        60        50
```

Weak Candidate 40

```
1       10        20        30        40        50        60        70        80        90        100
GUGCGUCAUGUUUACCUUUUGCACAGGUGCCCGACGUCGGGGUUCAGAUUGGGCUGAACCCCGACGUCGGGUACCUGUGAAAACGUUAGACAUGCGUCC
..(((.(((((((((.((((.((((((((((((((((((((((((((......))))))))))))))))))))))))))))))).).)))))))))))..

........UGUUUACCUUUUGCACAGGUG......................................................................x17*
........UGUUUACCUUUUGCACAGGUGC.....................................................................x9
........UGUUUACCUUUUGCACAGGU.......................................................................x1
.........................................................................CCUGUGAAAACGUUAGACAUG.....x9*
.........................................................................CCUGUGAAAACGUUAGACAU......x2
```

Most common read: UGUUUACCUUUUGCACAGGUG (freq 17)

Complementarity: 19/21

Best mirbase match: bdi-miR7735-5p (e-value: 4.8)

```
Query  1   UGUUUACCUUUUGCAC  16
           ||||| |||| |||||
Sbjct  2   UGUUUUCCUUCUGCAC  17
```

```
Sequence   1 Structure      1
 ENERGY =   -70.0    1
Bases    1 to   99

        10         20        30        40        50
GU   U        - C    G                          AU
  GCG CAUGUUUA C UUUU CACAGGUGCCCGACGUCGGGGUUCAG  U
  UGC GUACAGAU G AAAA GUGUCCAUGGGCUGCAGCCCCAAGUC  G
CC   -        U C    -                          GG
         90        80         70        60
```

Weak Candidate 41

```
1       10        20        30        40        50        60        70        80        90        100
GUGAGAGUAUGGCGGGGGUUUCGACGAGGAGUGCUACGAUGUGCCACCGGCGGUCGCAGCGCUCCUCGUCGAAACCCCAUCCAAGCUCUACCCCGUCA
...(((((.(((..(((((((((((((((((((((.((((.((((...)))))))).)))))))))))))))))))))..))).))))).........

.....AGUAUGGCGGGGGUUUCGACG........................................................................x1*
....................................................................UCGAAACCCCAUCCAAGCUCU.........x939*
....................................................................UCGAAACCCCAUCCAAGCUC..........x39
....................................................................UCGAAACCCCAUCCAAGCU...........x29
....................................................................UCGAAACCCCAUCCAAGC............x6
....................................................................UCGAAACCCCAUCCAAGCUCUA........x2
```

Most common read: UCGAAACCCCAUCCAAGCUCU (freq 939)

Complementarity: 18/21

Best mirbase match: pma-miR-430b (e-value: 7.1)

```
Query  1   UCGAAACCCCAUCCAAGCUCU  21
           ||  |||  ||| ||||||||
Sbjct  23  UCACAACAACAUUCAAGCUCU  3
```

```
Sequence   1 Structure      1
 ENERGY =   -63.9    1
Bases    1 to   98

              10        20        30        40
GUG------     A   CG                    A    G    A
         AGAGU UGG  GGGGUUUCGACGAGGAGUGCU CGAU UGCC \
         UCUCG ACC  CCCCAAAGCUGCUCCUCGCGA GCUG GCGG C
ACUGCCCCA     A   UA                    C    -    C
       90        80        70        60         50
```

Weak Candidate 42

```
1       10        20        30        40        50        60        70        80        90        100
CACCCAGUUACGUUCCGAAAUAUCGGGACGAAGAACCAUUUCAGCGAAGAAGCGCUGCAGUCGCUCUUGGUCCCGAUCUUUCGGAACUUUACUGGGUG
(((((((...((((((((((.((((((((.((((.(.(((.(((((......))))).))).).)))).)))))))).))))))))))...)))))))

.......UUACGUUCCGAAAUAUCGGGA......................................................................x35*
.......UUACGUUCCGAAAUAUCGGG.......................................................................x1
.......UUACGUUCCGAAAUAUCGGGAC.....................................................................x1
........UACGUUCCGAAAUAUCGGGA......................................................................x1
........................................................................CCGAUCUUUCGGAACUUUACU.....x1*
........................................................................CCGAUCUUUCGGAACUUUA.......x1
```

Most common read: UUACGUUCCGAAAUAUCGGGA (freq 35)

Complementarity: 17/21

Best mirbase match: NONE (e-value: NONE)

```
Sequence   1 Structure      1
 ENERGY =   -56.9    1
Bases    1 to   98

        10        20        30        40
```

```
        UAC         U        G    A C   U     AA
CACCCAGU   GUUCCGAAA AUCGGGAC AAGA C AUU CAGCG  G
GUGGGUCA   CAAGGCUUU UAGCCCUG UUCU G UGA GUCGC  A
        UUU         C        G    C C   C     GA
       90        80        70        60        50
```

Weak Candidate 43

```
1       10        20        30        40        50        60        70        80        90        100
CAUGAACCUGUUCUAGGUCCGAAGACAAUUGCCAGGAAAGAACAUAUUAUGUGGAUCUUUCCUGGCAAUUAACUUUGUGCCAUAGAGUAGGUUCAUG
((((((((((((((((.(((((..((((((((((((((.((((...))))..)))))))))))))))..))))).))).))))))))))))))))

......CCUGUUCUAGGUCCGAAGACA......................................................................x3
.......CUGUUCUAGGUCCGAAGACAA.....................................................................x5
.......CUGUUCUAGGUCCGAAGACA......................................................................x5
........UGUUCUAGGUCCGAAGACAA.....................................................................x495*
........UGUUCUAGGUCCGAAGACA......................................................................x130
........UGUUCUAGGUCCGAAGACAAU....................................................................x3
........UGUUCUAGGUCCGAAGAC.......................................................................x1
............................UUGCCAGGAAAGAACAUAUUAUGUGG...........................................x1
........................................AACAUAUUAUGUGGAUCUUUCCUGGC...............................x1
......................................................................AACUUUGUGCCAUAGAGUAGG......x1*
```

Most common read: UGUUCUAGGUCCGAAGACAA (freq 495)

Complementarity: 17/20

Best mirbase match: hsa-miR-4782-3p (e-value: 1.7)

```
Query  2   GUUCUAGGUCCGAAGACAA  20
           ||||||| |  ||||||||
Sbjct  22  GUUCUAGAUAUGAAGACAA  4
```

```
Sequence   1 Structure      1
 ENERGY =   -53.4    1
Bases    1 to   97

        10         20        30        40
               -   C     AC               A-    U
CAUGAACCUGUUCUA GGU CGAAG  AAUUGCCAGGAAAGA  CAUA \
GUACUUGGAUGAGAU CCG GUUUC  UUAACGGUCCUUUCU  GUGU U
               A   U     AA               AG    A
      90        80        70        60        50
```

Weak Candidate 44

```
1       10        20        30        40        50        60        70        80        90        100
GUUCCCGAACUCGCAAGGGAGGAUAGCCACACCAUGUUUAUUGCCGUUCAUUGACCGUGGUGUGUCUAUCUUCCUUCGAGAGUUCUGCAACAAGAACG
(((.(.((((((.(.(((((((((((.(((((((((.(((.((.....)).))).))))))))).)))))))))))).).)))))).).))).......

....CCGAACUCGCAAGGGAGGAUA.........................................................................x4*
....CCGAACUCGCAAGGGAGGAU..........................................................................x2
....................................................................UCUUCCUUCGAGAGUUCUGCA.........x903*
....................................................................UCUUCCUUCGAGAGUUCUGCAA........x8
....................................................................UCUUCCUUCGAGAGUUCUGC..........x8
```

```
....................................................................UCUUCCUUCGAGAGUUCUG...........x1
.....................................................................CUUCCUUCGAGAGUUCUGCA.........x2
```

Most common read: UCUUCCUUCGAGAGUUCUGCA (freq 903)

Complementarity: 17/21

Best mirbase match: cin-miR-4001c-5p (e-value: 8.6)

```
Query  1   UCUUCCUUCGAGAGUUCUG  19
           | |||||| || ||||| |
Sbjct  19  UGUUCCUUGGAAAGUUCCG  1
```

```
Sequence   1 Structure      1
 ENERGY =   -42.5    1
Bases    1 to   98

               10        20        30        40
-------   C C      G A           C         U   U  CC
       GUU C GAACUC C AGGGAGGAUAG CACACCAUG UUA UG  \
       CAA G CUUGAG G UUCCUUCUAUC GUGUGGUGC AGU AC  G
GCAAGAA   C U      A C           U         C   U  UU
       90        80        70        60        50
```

___

Weak Candidate 45

```
1       10        20        30        40        50        60        70        80        90        100
GGUGCCGUGGAUUGGUGGUUGGUACCUCCUCGAGCACGUACUCGACGUGACGAGCGCGUGCUGGAGGAGGUUCCAAUCACCGAUCCACGGCACCAGCCG
((((((((((((((((((((((.((((((((.(((((((.((((......)))).))))))).)))))))).)))))))))))))))))))))).....

....CCGUGGAUUGGUGGUUGGUAC..........................................................................x1
.....CGUGGAUUGGUGGUUGGUACC.........................................................................x16*
.....CGUGGAUUGGUGGUUGGUAC..........................................................................x10
.....CGUGGAUUGGUGGUUGGUA...........................................................................x2
..........................UCCUCGAGCACGUACUCGACGUGA.................................................x1
.............................................CGUGACGAGCGCGUGCUGGAGGAGG.............................x1
..................................................CGAGCGCGUGCUGGAGGAGG.............................x3
...................................................GAGCGCGUGCUGGAGGAGG.............................x1
....................................................AGCGCGUGCUGGAGGAGG.............................x2
......................................................................UUCCAAUCACCGAUCCACGGC........x860*
......................................................................UUCCAAUCACCGAUCCACGG.........x129
......................................................................UUCCAAUCACCGAUCCACGGCA.......x76
......................................................................UUCCAAUCACCGAUCCACG..........x8
......................................................................UUCCAAUCACCGAUCCACGGCAC......x1
.......................................................................UCCAAUCACCGAUCCACGG.........x1
.......................................................................UCCAAUCACCGAUCCACGGCA.......x1
```

Most common read: UUCCAAUCACCGAUCCACGGC (freq 860)

Complementarity: 20/21

Best mirbase match: mdo-miR-7275-3p (e-value: 4.0)

```
Query  1   UUCCAAUCACCGAUCCAC  18
           | ||||||||| || |||
Sbjct  23  UCCCAAUCACCCAUUCAC  6
```

```
Sequence   1 Structure      1
 ENERGY =   -82.5    1
Bases    1 to   99
```

```
            10        20        30        40
-----                 U        G       A    AC
     GGUGCCGUGGAUUGGUGGUUGG ACCUCCUC AGCACGU CUCG  G
     CCACGGCACCUAGCCACUAACC UGGAGGAG UCGUGCG GAGC  U
GCCGA                 U        G       C    AG
       90        80        70        60        50
```

____

```
Weak Candidate 46

1       10        20        30        40        50        60        70        80        90        100
GAUGUCCGUGAUUGCACGGGUACACAUGUCAGCACCUUUCUCUAGCGCAUAGCAAAAGAUGCCGAUAUUUGCCCCAUGCACGCACGGUCAACCGGG
..((.(((((..((((.(((((...(((((.(((.((((..(((.....)))..)))).))).))))).)))))..)))).))))).))......

....UCCGUGAUUGCACGGGUACACA......................................................................x1*
.....................................................................UGCCCCAUGCACGCACGGUCA......x1821*
.....................................................................UGCCCCAUGCACGCACGGUC.......x42
.....................................................................UGCCCCAUGCACGCACGGU........x24
.....................................................................UGCCCCAUGCACGCACGGUCAA.....x4
.....................................................................UGCCCCAUGCACGCACGG.........x3
......................................................................GCCCCAUGCACGCACGGUCA......x2
........................................................................CCCAUGCACGCACGGUCA......x1

Most common read: UGCCCCAUGCACGCACGGUCA (freq 1821)

Complementarity: 16/21

Best mirbase match: NONE (e-value: NONE)



Sequence   1 Structure      1
 ENERGY =   -34.1    1
Bases    1 to   96

           10         20        30        40
GA----  U     AU    C-     CAC     A   C    CU   GC
      UG CCGUG  UGCA  GGGUA   AUGUC GCA CUUU  CUA  \
      AC GGCAC  ACGU  CCCGU   UAUAG CGU GAAA  GAU  G
GGGCCA  U     GC    AC     U--     C   A    AC   AC
     90        80        70          60        50
```

____

Previously annotated Ectocarpus miRNAS listed in miRBase, which we now consider to also be weak candidates.

```
esi-MIR3450
GTCGTTGGGTACATCGCCCGACCACTCTATGAAACCGGGTCATTCCCGACCGAAGTTCGTCCAAAGGCAAGGCACCTTTCGACGGACCCCGGTCAGCAATGACCCGGTTTTATAGAATGGTCTGGCGACGTACCAACGAA
.((((((.(((.(((((.(((((.((((((((((((((((((((.(.(((((..(((((((.(((((.......))))).)))))))..))))).).))))))))))))))))))).))))).))))).)))))))))).
.........XXXXXXXXXXXXXXXXXXXXX...................................................................................*********************......
.....TGGGTACATCGCCCGACCACT..................................................................................................................x1.(0.01%)
.....TGGGTACATCGCCCGACCACTCT................................................................................................................x1.(0.01%)
......GGGTACATCGCCCGACCACTCTAT..............................................................................................................x1.(0.01%)
.......GGTACATCGCCCGACCACTCTAT..............................................................................................................x1.(0.01%)
........GTACATCGCCCGACCACT..................................................................................................................x1.(0.01%)
........GTACATCGCCCGACCACTCT................................................................................................................x2.(0.02%)
.........TACATCGCCCGACCACTCTA...............................................................................................................x509.(5.72%)
.........TACATCGCCCGACCACTCTATG.............................................................................................................x12.(0.13%)
.........TACATCGCCCGACCACTCT................................................................................................................x59.(0.66%)
.........TACATCGCCCGACCACTCTAT..............................................................................................................x5818.(65.42%)*
```

```
..........ACATCGCCCGACCACTCTAT..............................................................................................................x136.(1.53%)
..........ACATCGCCCGACCACTCTA...............................................................................................................x29.(0.33%)
..........ACATCGCCCGACCACTCT................................................................................................................x3.(0.03%)
...........CATCGCCCGACCACTCTA...............................................................................................................x2.(0.02%)
...........CATCGCCCGACCACTCTAT..............................................................................................................x12.(0.13%)
............ATCGCCCGACCACTCTAT..............................................................................................................x3.(0.03%)
...........................TATGAAACCGGGTCATTCCCGA...........................................................................................x1.(0.01%)
.............................TGAAACCGGGTCATTCCCGACC.........................................................................................x1.(0.01%)
.............................TGAAACCGGGTCATTCCCGA...........................................................................................x2.(0.02%)
..................................................CGAAGTTCGTCCAAAGGCAA......................................................................x1.(0.01%)
.................................................................GGCAAGGCACCTTTCGACGGA......................................................x1.(0.01%)
.......................................................................................CCCGGTCAGCAATGACCCGGTTTTAT...........................x1.(0.01%)
........................................................................................CCGGTCAGCAATGACCCGGTTTTATA..........................x1.(0.01%)
.........................................................................................CGGTCAGCAATGACCCGGTTTTAT...........................x3.(0.03%)
............................................................................................TCAGCAATGACCCGGTTTTAT...........................x1400.(15.74%)*
............................................................................................TCAGCAATGACCCGGTTT..............................x2.(0.02%)
............................................................................................TCAGCAATGACCCGGTTTTATA..........................x124.(1.39%)
............................................................................................TCAGCAATGACCCGGTTTTA............................x90.(1.01%)
.............................................................................................CAGCAATGACCCGGTTTTAT...........................x8.(0.09%)
.............................................................................................CAGCAATGACCCGGTTTTA............................x1.(0.01%)
.............................................................................................CAGCAATGACCCGGTTTTATA..........................x1.(0.01%)
..............................................................................................AGCAATGACCCGGTTTTATAG.........................x2.(0.02%)
..............................................................................................AGCAATGACCCGGTTTTATA..........................x1.(0.01%)
..............................................................................................AGCAATGACCCGGTTTTAT...........................x8.(0.09%)
..............................................................................................AGCAATGACCCGGTTTTATAGA........................x3.(0.03%)
...............................................................................................GCAATGACCCGGTTTTAT...........................x1.(0.01%)
...........................................................................................................TTTTATAGAATGGTCTGGCGA............x1.(0.01%)
............................................................................................................TTTATAGAATGGTCTGGCGAC...........x2.(0.02%)
................................................................................................................TAGAATGGTCTGGCGACGT.........x1.(0.01%)
................................................................................................................TAGAATGGTCTGGCGACGTAC.......x374.(4.21%)
................................................................................................................TAGAATGGTCTGGCGACGTACC......x21.(0.24%)
................................................................................................................TAGAATGGTCTGGCGACGTA........x13.(0.15%)
.................................................................................................................AGAATGGTCTGGCGACGTACC......x219.(2.46%)
.................................................................................................................AGAATGGTCTGGCGACGTACCA.....x2.(0.02%)
.................................................................................................................AGAATGGTCTGGCGACGTAC.......x17.(0.19%)
...................................................................................................................AATGGTCTGGCGACGTACCAACG..x1.(0.01%)
Complementarity = 17

Folding energy = -89.30

Most common read: UACAUCGCCCGACCACUCUAU (freq 5818) matching esi-miR3450-5p (e-value: 0.002)

Query  1   UACAUCGCCCGACCACUCUAU  21
           |||||||||||||||||||||
Sbjct  1   UACAUCGCCCGACCACUCUAU  21


        10        20        30        40        50        60        70
G       G   A     C     C                   C C     AA       C     CAA
 UCGUUGG UAC UCGCC GACCA UCUAUGAAACCGGGUCAUU C GACCG  GUUCGUC AAAGG   \
 AGCAACC AUG AGCGG CUGGU AGAUAUUUUGGCCCAGUAA G CUGGC  CAGGCAG UUUCC   G
A       -   C     U     A                   C A     CC       C     ACG
.        130       120       110       100        90        80
```

```
esi-MIR3451
CCTACTGATGGTTGTAGACCTTCCTGCCGCAAACATCACATGTGCAATGTGCATGTTTGCGGCTGGAACAAGTGATGTTCGCGGCAGGGAGGTTCACAACCATCAGTAG
.((((((((((((((..(((((((((((((.((((((((.(((.(...(((((....))).))..).))).)))))))).)))))))))))))..))))))))))))))
...........XXXXXXXXXXXXXXXXXXXXXXX.............................................**********************........
..TACTGATGGTTGTAGACCTTC......................................................................................x1.(0.02%)
.....TGATGGTTGTAGACCTTCCTG...................................................................................x4.(0.07%)
......GATGGTTGTAGACCTTCCTG...................................................................................x1.(0.02%)
```

```
........TGGTTGTAGACCTTCCTGCCG................................................................................x1.(0.02%)
........TGGTTGTAGACCTTCCTG...................................................................................x1.(0.02%)
........TGGTTGTAGACCTTCCTGCC.................................................................................x2.(0.03%)
...........TTGTAGACCTTCCTGCCGCAA.............................................................................x71.(1.2%)
...........TTGTAGACCTTCCTGCCGCAAACA..........................................................................x190.(3.21%)
...........TTGTAGACCTTCCTGCCGCAAAC...........................................................................x1767.(29.82%)*
...........TTGTAGACCTTCCTGCCGCA..............................................................................x3.(0.05%)
...........TTGTAGACCTTCCTGCCGCAAA............................................................................x1184.(19.98%)
...........TTGTAGACCTTCCTGCCGCAAACAT.........................................................................x7.(0.12%)
............TGTAGACCTTCCTGCCGCAAA............................................................................x65.(1.1%)
............TGTAGACCTTCCTGCCGCAA.............................................................................x3.(0.05%)
............TGTAGACCTTCCTGCCGCAAAC...........................................................................x153.(2.58%)
............TGTAGACCTTCCTGCCGCAAACA..........................................................................x16.(0.27%)
.............GTAGACCTTCCTGCCGCAAAC...........................................................................x7.(0.12%)
.............GTAGACCTTCCTGCCGCAAA............................................................................x3.(0.05%)
.............GTAGACCTTCCTGCCGCAA.............................................................................x1.(0.02%)
..............TAGACCTTCCTGCCGCAAACA..........................................................................x96.(1.62%)
..............TAGACCTTCCTGCCGCAAA............................................................................x2.(0.03%)
..............TAGACCTTCCTGCCGCAAAC...........................................................................x28.(0.47%)
.............................................................................TTCGCGGCAGGGAGGTTCAC............x14.(0.24%)
.............................................................................TTCGCGGCAGGGAGGTTCA.............x5.(0.08%)
.............................................................................TTCGCGGCAGGGAGGTTCACA...........x502.(8.47%)
.............................................................................TTCGCGGCAGGGAGGTTCACAA..........x19.(0.32%)
..............................................................................TCGCGGCAGGGAGGTTCA.............x1.(0.02%)
..............................................................................TCGCGGCAGGGAGGTTCACA...........x422.(7.12%)
..............................................................................TCGCGGCAGGGAGGTTCACAAC.........x4.(0.07%)
..............................................................................TCGCGGCAGGGAGGTTCAC............x8.(0.13%)
..............................................................................TCGCGGCAGGGAGGTTCACAA..........x1332.(22.48%)*
...............................................................................CGCGGCAGGGAGGTTCACAACC........x1.(0.02%)
...............................................................................CGCGGCAGGGAGGTTCACA...........x1.(0.02%)
...............................................................................CGCGGCAGGGAGGTTCAC............x1.(0.02%)
................................................................................GCGGCAGGGAGGTTCACAA..........x3.(0.05%)
................................................................................GCGGCAGGGAGGTTCACA...........x6.(0.1%)
.................................................................................CGGCAGGGAGGTTCACAA..........x1.(0.02%)
```

```
Complementarity = 19

Folding energy = -71.40

Most common read: UUGUAGACCUUCCUGCCGCAAAC (freq 1767) matching esi-miR3451-5p (e-value: 2e-04)

Query  1   UUGUAGACCUUCCUGCCGCAAAC  23
           |||||||||||||||||||||||
Sbjct  1   UUGUAGACCUUCCUGCCGCAAAC  23


        10        20        30        40         50
C              AG             A        A   G AAU  -   U
 CUACUGAUGGUUGU  ACCUUCCUGCCGC AACAUCAC UGU C   GU GCA G
 GAUGACUACCAACA  UGGAGGGACGGCG UUGUAGUG ACA G   CG CGU U
-              CU             C        A   A GU-  G   U
        100        90        80        70         60
```

____

```
esi-MIR3452
TACATAGACCAGTTCGGACGGCGGTGTAGTACGCTTCCGAAAACGGGAGCAAGGCTAGCTCCTTCCGCTGGATGTCCATGT
.((((.(((..((((((.(((.((.(((((..(((((((....)))))))...))).)).))..)))))))))))).))))
...*********************......................................XXXXXXXXXXXXXXXXXXX
TACATAGACCAGTTCGGAC..............................................................x26.(0.64%)
TACATAGACCAGTTCGGACGGC...........................................................x2.(0.05%)
TACATAGACCAGTTCGGA...............................................................x7.(0.17%)
```

```
TACATAGACCAGTTCGGACG.............................................................x301.(7.46%)
TACATAGACCAGTTCGGACGG............................................................x1084.(26.85%)*
.ACATAGACCAGTTCGGACGG............................................................x3.(0.07%)
.ACATAGACCAGTTCGGACG.............................................................x42.(1.04%)
.ACATAGACCAGTTCGGAC..............................................................x6.(0.15%)
..CATAGACCAGTTCGGACG.............................................................x2.(0.05%)
..CATAGACCAGTTCGGACGG............................................................x1.(0.02%)
....TAGACCAGTTCGGACGGCGGT........................................................x15.(0.37%)
....TAGACCAGTTCGGACGGCGG.........................................................x9.(0.22%)
....TAGACCAGTTCGGACGGCG..........................................................x2.(0.05%)
............TTCGGACGGCGGTGTAGTACG................................................x5.(0.12%)
............TTCGGACGGCGGTGTAGTAC.................................................x1.(0.02%)
.............TCGGACGGCGGTGTAGTACGC...............................................x10.(0.25%)
..............CGGACGGCGGTGTAGTACGC...............................................x1.(0.02%)
...................GGCGGTGTAGTACGCTTCCGA.........................................x1.(0.02%)
....................GCGGTGTAGTACGCTTCCGAA........................................x1.(0.02%)
.......................GTGTAGTACGCTTCCGAAAAC.....................................x1.(0.02%)
........................TGTAGTACGCTTCCGAAAACGG...................................x5.(0.12%)
........................TGTAGTACGCTTCCGAAAACG....................................x2.(0.05%)
........................TGTAGTACGCTTCCGAAAAC.....................................x1.(0.02%)
.........................GTAGTACGCTTCCGAAAACGG...................................x2.(0.05%)
..........................TAGTACGCTTCCGAAAACGGG..................................x1.(0.02%)
..................................TTCCGAAAACGGGAGCAAGGC..........................x1.(0.02%)
...................................TCCGAAAACGGGAGCAAGGCTA........................x1.(0.02%)
.......................................................TAGCTCCTTCCGCTGGATGTC.....x3.(0.07%)
...........................................................TCCTTCCGCTGGATGTCCATG.x1.(0.02%)
...........................................................TCCTTCCGCTGGATGTCCAT..x1.(0.02%)
.............................................................CTTCCGCTGGATGTCCATGTx1.(0.02%)
..............................................................TTCCGCTGGATGTCCATGTx2463.(61.01%)*
..............................................................TTCCGCTGGATGTCCATG.x23.(0.57%)
...............................................................TCCGCTGGATGTCCATGTx12.(0.3%)
```

Complementarity = 15

Folding energy = -31.60

Most common read: UUCCGCUGGAUGUCCAUGU (freq 2463) matching esi-miR3452-3p (e-value: 0.010)

```
Query  1   UUCCGCUGGAUGUCCAUGU  19
           |||||||||||||||||||
Sbjct  1   UUCCGCUGGAUGUCCAUGU  19
```

```
       10        20          30         40
U    A   CA      A   C-  U  -   AC-       A
 ACAU GAC  GUUCGG CGG  GG GU AGU   GCUUCCG A
 UGUA CUG  UAGGUC GCC  CC CG UCG   CGAGGGC A
-    C   --      -   UU  U  A   GAA       A
 80          70         60        50
```

---

esi-MIR3454a

```
GCTCGTGGAGTTTTTCCCAGCTCCGACGGGATGGCGCTCGTGACTAGGTCCGCCGCCTTGTATCATACGAGCGTGTGTATACCCGTGCGACCCAAGGCGGCGGAACTAGTCACGAGCGCCCTTCCATCGGAGCTAGGTAGAATTTCACGAG
.(((((((((((((.((.((((((((.((((.(((((((((((((((.((((((((((((..((.((((.(.(((....)))))))).))..)))))))))))).))))))))))))))).)))).)))))))).)).)))))))))))))
.............XXXXXXXXXXXXXXXXXXXXX......................................................*********************..........
..TCGTGGAGTTTTTCCCAGCTC................................................................................................................................x1.(0.0%)
..........TTTTTCCCAGCTCCGACGGGATGG.....................................................................................................................x3.(0.0%)
..........TTTTTCCCAGCTCCGACGGGA........................................................................................................................x12.(0.01%)
..........TTTTTCCCAGCTCCGACGGGAT.......................................................................................................................x2.(0.0%)
..........TTTTTCCCAGCTCCGACGG..........................................................................................................................x1.(0.0%)
...........TTTTCCCAGCTCCGACGGGATG......................................................................................................................x1.(0.0%)
...........TTTTCCCAGCTCCGACGGGA........................................................................................................................x1.(0.0%)
```

...........TTTTCCCAGCTCCGACGGGATGG...x3.(0.0%)
...........TTTTCCCAGCTCCGACGGGAT...x9.(0.01%)
............TTTCCCAGCTCCGACGGGAT...x11.(0.01%)
............TTTCCCAGCTCCGACGGGATG..x21.(0.02%)
............TTTCCCAGCTCCGACGGGA..x17.(0.02%)
............TTTCCCAGCTCCGACGGGATGG...x137.(0.14%)
.............TTCCCAGCTCCGACGGGA..x580.(0.6%)
.............TTCCCAGCTCCGACGGGATGG...x87814.(90.86%)*
.............TTCCCAGCTCCGACGGGAT...x176.(0.18%)
.............TTCCCAGCTCCGACGGGATGGC..x373.(0.39%)
.............TTCCCAGCTCCGACGGGATGGCG...x9.(0.01%)
.............TTCCCAGCTCCGACGGGATG..x2291.(2.37%)
..............TCCCAGCTCCGACGGGATGGCG...x21.(0.02%)
..............TCCCAGCTCCGACGGGATGG...x971.(1.0%)
..............TCCCAGCTCCGACGGGAT...x5.(0.01%)
..............TCCCAGCTCCGACGGGATGGC..x20.(0.02%)
..............TCCCAGCTCCGACGGGATG..x20.(0.02%)
...............CCCAGCTCCGACGGGATGGCGC..x1.(0.0%)
...............CCCAGCTCCGACGGGATGG...x45.(0.05%)
...............CCCAGCTCCGACGGGATG..x1.(0.0%)
................CCAGCTCCGACGGGATGG...x329.(0.34%)
.................CAGCTCCGACGGGATGGCGCTCGTGA..x1.(0.0%)
.....................TCCGACGGGATGGCGCTCGTG...x7.(0.01%)
.....................TCCGACGGGATGGCGCTCGT..x2.(0.0%)
...............................TGGCGCTCGTGACTAGGTCCGCCG..x1.(0.0%)
...............................TGGCGCTCGTGACTAGGTCCGC..x10.(0.01%)
...............................TGGCGCTCGTGACTAGGTCC..x15.(0.02%)
...............................TGGCGCTCGTGACTAGGT..x1.(0.0%)
...............................TGGCGCTCGTGACTAGGTCCG...x51.(0.05%)
..................................CGCTCGTGACTAGGTCCGCC...x53.(0.05%)
..................................CGCTCGTGACTAGGTCCGCCGC...x40.(0.04%)
..................................CGCTCGTGACTAGGTCCGCCG..x404.(0.42%)
..................................CGCTCGTGACTAGGTCCG...x4.(0.0%)
..................................CGCTCGTGACTAGGTCCGC..x3.(0.0%)
...................................GCTCGTGACTAGGTCCGCCG..x10.(0.01%)
...................................GCTCGTGACTAGGTCCGCC...x1.(0.0%)
....................................CTCGTGACTAGGTCCGCCG..x3.(0.0%)
.....................................TCGTGACTAGGTCCGCCGCC..x4.(0.0%)
.....................................TCGTGACTAGGTCCGCCGC...x1.(0.0%)
.....................................TCGTGACTAGGTCCGCCGCCT...x27.(0.03%)
....TGACTAGGTCCGCCGCCTTG...x4.(0.0%)
....TGACTAGGTCCGCCGCCTT..x1.(0.0%)
....GCGGAACTAGTCACGAGC..................................x22.(0.02%)
....GCGGAACTAGTCACGAGCGCCC..............................x68.(0.07%)
....GCGGAACTAGTCACGAGCG.................................x20.(0.02%)
....GCGGAACTAGTCACGAGCGCC...............................x1521.(1.57%)*
....GCGGAACTAGTCACGAGCGC................................x180.(0.19%)
....CGGAACTAGTCACGAGCGCC...............................x12.(0.01%)
....CGGAACTAGTCACGAGCGCCC..............................x60.(0.06%)
....CGGAACTAGTCACGAGCG.................................x1.(0.0%)
....CGGAACTAGTCACGAGCGCCCT.............................x1.(0.0%)
....CGGAACTAGTCACGAGCGC................................x3.(0.0%)
....GGAACTAGTCACGAGCGCCCTT............................x1.(0.0%)
....GGAACTAGTCACGAGCGCC...............................x3.(0.0%)
....GGAACTAGTCACGAGCGCCC..............................x2.(0.0%)
....GGAACTAGTCACGAGCGC................................x1.(0.0%)
....GAACTAGTCACGAGCGCC...............................x2.(0.0%)
....TCACGAGCGCCCTTCCATCGG.....................x5.(0.01%)
....CCCTTCCATCGGAGCTAGGTA............x2.(0.0%)
....CTTCCATCGGAGCTAGGTAG...........x30.(0.03%)
....CTTCCATCGGAGCTAGGTA............x35.(0.04%)
....CTTCCATCGGAGCTAGGTAGAA.........x71.(0.07%)
....CTTCCATCGGAGCTAGGTAGA..........x850.(0.88%)
....CTTCCATCGGAGCTAGGT.............x7.(0.01%)

```
.........................................................................................................................TTCCATCGGAGCTAGGTAGAATT.......x1.(0.0%)
.........................................................................................................................TTCCATCGGAGCTAGGTAG...........x1.(0.0%)
.........................................................................................................................TTCCATCGGAGCTAGGTAGAA.........x181.(0.19%)
.........................................................................................................................TTCCATCGGAGCTAGGTA............x4.(0.0%)
.........................................................................................................................TTCCATCGGAGCTAGGTAGA..........x18.(0.02%)
.........................................................................................................................TTCCATCGGAGCTAGGTAGAAT........x1.(0.0%)
..........................................................................................................................TCCATCGGAGCTAGGTAGAAT........x24.(0.02%)
..........................................................................................................................TCCATCGGAGCTAGGTAGAA.........x2.(0.0%)
................................................................................................................................GGAGCTAGGTAGAATTTCACG..x1.(0.0%)
```

Complementarity = 16

Folding energy = -107.00

Most common read: UUCCCAGCUCCGACGGGAUGG (freq 87814) matching esi-miR3454b-5p (e-value: 0.002)

```
Query  1   UUCCCAGCUCCGACGGGAUGG  21
           |||||||||||||||||||||
Sbjct  1   UUCCCAGCUCCGACGGGAUGG  21
```

```
        10        20        30        40        50        60        70
G             U  C        C    U               G            UA  A    A C   U
 CUCGUGGAGUUUU CC AGCUCCGA GGGA GGCGCUCGUGACUAG UCCGCCGCCUUG  UC UACG G GUG G
 GAGCACUUUAAGA GG UCGAGGCU CCUU CCGCGAGCACUGAUC AGGCGGCGGAAC  AG GUGC C CAU U
-             U  A        A    C               A            CC  C    - -   A
150       140       130       120       110       100       90          80
```

____

esi-MIR3454b

```
GCTCGTGGAGTTTTTCCCAGCTCCGACGGGATGGCGCTCGTGACTAGGTCCGCCGCCTTGTATCACACGAGCGTGTGTATATCTGTGCGACTCAAGGCGGCGGAACTAGTCACGAGCGCCCTTCCGTCGGAGCTGGGTAGAATTTCACGAGC
((((((((((((.(((((((((((((((.((((((((((((((.(((((((((((..((.(((((.....................)).))).))..))))))))))).)))))))))))))).))))))))))))))).))))))))))))
.............XXXXXXXXXXXXXXXXXXXXX......................................................................................*********************...........
..TCGTGGAGTTTTTCCCAGCTC.................................................................................................................................x1.(0.0%)
..........TTTTTCCCAGCTCCGACGGGAT........................................................................................................................x2.(0.0%)
..........TTTTTCCCAGCTCCGACGGGA.........................................................................................................................x12.(0.01%)
..........TTTTTCCCAGCTCCGACGG...........................................................................................................................x1.(0.0%)
..........TTTTTCCCAGCTCCGACGGGATGG......................................................................................................................x3.(0.0%)
...........TTTTCCCAGCTCCGACGGGAT........................................................................................................................x9.(0.01%)
...........TTTTCCCAGCTCCGACGGGATGG......................................................................................................................x3.(0.0%)
...........TTTTCCCAGCTCCGACGGGA.........................................................................................................................x1.(0.0%)
...........TTTTCCCAGCTCCGACGGGATG.......................................................................................................................x1.(0.0%)
............TTTCCCAGCTCCGACGGGATG.......................................................................................................................x21.(0.02%)
............TTTCCCAGCTCCGACGGGA.........................................................................................................................x17.(0.02%)
............TTTCCCAGCTCCGACGGGATGG......................................................................................................................x137.(0.14%)
............TTTCCCAGCTCCGACGGGAT........................................................................................................................x11.(0.01%)
.............TTCCCAGCTCCGACGGGATGG......................................................................................................................x87814.(91.0%)*
.............TTCCCAGCTCCGACGGGAT........................................................................................................................x176.(0.18%)
.............TTCCCAGCTCCGACGGGATGGCG....................................................................................................................x9.(0.01%)
.............TTCCCAGCTCCGACGGGATGGC.....................................................................................................................x373.(0.39%)
.............TTCCCAGCTCCGACGGGATG.......................................................................................................................x2291.(2.37%)
.............TTCCCAGCTCCGACGGGA.........................................................................................................................x580.(0.6%)
..............TCCCAGCTCCGACGGGATGGCG....................................................................................................................x21.(0.02%)
..............TCCCAGCTCCGACGGGATG.......................................................................................................................x20.(0.02%)
..............TCCCAGCTCCGACGGGATGG......................................................................................................................x971.(1.01%)
..............TCCCAGCTCCGACGGGATGGC.....................................................................................................................x20.(0.02%)
..............TCCCAGCTCCGACGGGAT........................................................................................................................x5.(0.01%)
...............CCCAGCTCCGACGGGATG.......................................................................................................................x1.(0.0%)
...............CCCAGCTCCGACGGGATGG......................................................................................................................x45.(0.05%)
...............CCCAGCTCCGACGGGATGGCGC...................................................................................................................x1.(0.0%)
................CCAGCTCCGACGGGATGG......................................................................................................................x329.(0.34%)
.................CAGCTCCGACGGGATGGCGCTCGTGA.............................................................................................................x1.(0.0%)
```

```
....................TCCGACGGGATGGCGCTCGTG.............................................................................................................x7.(0.01%)
....................TCCGACGGGATGGCGCTCGT..............................................................................................................x2.(0.0%)
..............................TGGCGCTCGTGACTAGGTCCGC..................................................................................................x10.(0.01%)
..............................TGGCGCTCGTGACTAGGT......................................................................................................x1.(0.0%)
..............................TGGCGCTCGTGACTAGGTCC....................................................................................................x15.(0.02%)
..............................TGGCGCTCGTGACTAGGTCCGCCG................................................................................................x1.(0.0%)
..............................TGGCGCTCGTGACTAGGTCCG...................................................................................................x51.(0.05%)
.................................CGCTCGTGACTAGGTCCGCC.................................................................................................x53.(0.05%)
.................................CGCTCGTGACTAGGTCCGCCGC...............................................................................................x40.(0.04%)
.................................CGCTCGTGACTAGGTCCGC..................................................................................................x3.(0.0%)
.................................CGCTCGTGACTAGGTCCGCCG................................................................................................x404.(0.42%)
.................................CGCTCGTGACTAGGTCCG...................................................................................................x4.(0.0%)
..................................GCTCGTGACTAGGTCCGCCG................................................................................................x10.(0.01%)
..................................GCTCGTGACTAGGTCCGCC.................................................................................................x1.(0.0%)
...................................CTCGTGACTAGGTCCGCCG................................................................................................x3.(0.0%)
....................................TCGTGACTAGGTCCGCCGCC..............................................................................................x4.(0.0%)
....................................TCGTGACTAGGTCCGCCGCCT.............................................................................................x27.(0.03%)
....................................TCGTGACTAGGTCCGCCGC...............................................................................................x1.(0.0%)
.......................................TGACTAGGTCCGCCGCCTTG...........................................................................................x4.(0.0%)
.......................................TGACTAGGTCCGCCGCCTT............................................................................................x1.(0.0%)
.......................................................................TGTGTATATCTGTGCGACTCAAG........................................................x1.(0.0%)
.......................................................................TGTGTATATCTGTGCGACTCAAGGCG.....................................................x1.(0.0%)
...............................................................................TCTGTGCGACTCAAGGCGGC...................................................x1.(0.0%)
...............................................................................TCTGTGCGACTCAAGGCGGCGG.................................................x1.(0.0%)
...................................................................................TGCGACTCAAGGCGGCGG.................................................x1.(0.0%)
...................................................................................TGCGACTCAAGGCGGCGGAACT.............................................x1.(0.0%)
.........................................................................................TCAAGGCGGCGGAACTAGTCA........................................x8.(0.01%)
.................................................................................................GCGGAACTAGTCACGAGC...................................x22.(0.02%)
.................................................................................................GCGGAACTAGTCACGAGCG..................................x20.(0.02%)
.................................................................................................GCGGAACTAGTCACGAGCGC.................................x180.(0.19%)
.................................................................................................GCGGAACTAGTCACGAGCGCC................................x1521.(1.58%)*
.................................................................................................GCGGAACTAGTCACGAGCGCCC...............................x68.(0.07%)
..................................................................................................CGGAACTAGTCACGAGCG..................................x1.(0.0%)
..................................................................................................CGGAACTAGTCACGAGCGC.................................x3.(0.0%)
..................................................................................................CGGAACTAGTCACGAGCGCC................................x12.(0.01%)
..................................................................................................CGGAACTAGTCACGAGCGCCC...............................x60.(0.06%)
..................................................................................................CGGAACTAGTCACGAGCGCCCT..............................x1.(0.0%)
...................................................................................................GGAACTAGTCACGAGCGC.................................x1.(0.0%)
...................................................................................................GGAACTAGTCACGAGCGCCC...............................x2.(0.0%)
...................................................................................................GGAACTAGTCACGAGCGCC................................x3.(0.0%)
...................................................................................................GGAACTAGTCACGAGCGCCCTT.............................x1.(0.0%)
....................................................................................................GAACTAGTCACGAGCGCC................................x2.(0.0%)
...........................................................................................................TCACGAGCGCCCTTCCGTCGG......................x1.(0.0%)
.....................................................................................................................CCTTCCGTCGGAGCTGGG...............x9.(0.01%)
.....................................................................................................................CCTTCCGTCGGAGCTGGGTA.............x2.(0.0%)
.....................................................................................................................CCTTCCGTCGGAGCTGGGTAG............x4.(0.0%)
......................................................................................................................CTTCCGTCGGAGCTGGGTA.............x26.(0.03%)
......................................................................................................................CTTCCGTCGGAGCTGGGT..............x7.(0.01%)
......................................................................................................................CTTCCGTCGGAGCTGGGTAG............x25.(0.03%)
......................................................................................................................CTTCCGTCGGAGCTGGGTAGA...........x392.(0.41%)
......................................................................................................................CTTCCGTCGGAGCTGGGTAGAA..........x40.(0.04%)
......................................................................................................................CTTCCGTCGGAGCTGGGTAGAATT........x1.(0.0%)
.......................................................................................................................TTCCGTCGGAGCTGGGTAGAAT.........x23.(0.02%)
.......................................................................................................................TTCCGTCGGAGCTGGGTA.............x7.(0.01%)
.......................................................................................................................TTCCGTCGGAGCTGGGTAG............x5.(0.01%)
.......................................................................................................................TTCCGTCGGAGCTGGGTAGAA..........x264.(0.27%)
.......................................................................................................................TTCCGTCGGAGCTGGGTAGA...........x67.(0.07%)
.......................................................................................................................TTCCGTCGGAGCTGGGTAGAATT........x5.(0.01%)
........................................................................................................................TCCGTCGGAGCTGGGTAGA...........x1.(0.0%)
........................................................................................................................TCCGTCGGAGCTGGGTAGAA..........x2.(0.0%)
........................................................................................................................TCCGTCGGAGCTGGGTAGAATTT.......x1.(0.0%)
........................................................................................................................TCCGTCGGAGCTGGGTAGAATT........x10.(0.01%)
........................................................................................................................TCCGTCGGAGCTGGGTAGAAT.........x37.(0.04%)
............................................................................................................................TCGGAGCTGGGTAGAATTT.......x1.(0.0%)
```

```
..............................................................................................................................TCGGAGCTGGGTAGAATTTCA.....x71.(0.07%)
..............................................................................................................................TCGGAGCTGGGTAGAATTTC......x12.(0.01%)
..............................................................................................................................TCGGAGCTGGGTAGAATTTCACG...x1.(0.0%)
...............................................................................................................................CGGAGCTGGGTAGAATTTC......x1.(0.0%)
................................................................................................................................GGAGCTGGGTAGAATTTCACG...x40.(0.04%)
................................................................................................................................GGAGCTGGGTAGAATTTCACGA..x4.(0.0%)
................................................................................................................................GGAGCTGGGTAGAATTTCAC....x4.(0.0%)
.................................................................................................................................GAGCTGGGTAGAATTTCACGA..x1.(0.0%)
.................................................................................................................................GAGCTGGGTAGAATTTCACG...x1.(0.0%)
...................................................................................................................................GCTGGGTAGAATTTCACGAGCx2.(0.0%)
```

Complementarity = 18

Folding energy = -120.90

Most common read: UUCCCAGCUCCGACGGGAUGG (freq 87814) matching esi-miR3454b-5p (e-value: 0.002)

```
Query  1   UUCCCAGCUCCGACGGGAUGG  21
           |||||||||||||||||||||
Sbjct  1   UUCCCAGCUCCGACGGGAUGG  21
```

```
        10        20        30        40        50        60         70
              U                U               G            UA  A   -  GCGUG
GCUCGUGGAGUUUU CCCAGCUCCGACGGGA GGCGCUCGUGACUAG UCCGCCGCCUUG  UC CAC GA     \
CGAGCACUUUAAGA GGGUCGAGGCUGCCUU CCGCGAGCACUGAUC AGGCGGCGGAAC  AG GUG CU     U
              U                C               A            UC  C   U  AUAUG
150       140       130       120       110       100        90        80
```

____

```
esi-MIR3454c
GAGTTTTTCCCAGCTCCGACGGGATGGCACTCGTGACTAGTTCCGCCGCCTTGTATCACACGAGCGTGTGTATCCCCGTGCGACTCAAGGCGGCGGAACTAGCGACGAGCGCCCTTCCATCGGAGCTGGGTAGAATTCA
(((((((.(((((((((((.((((.(((.(((((..(((((((((((((((((..((.((((.(.(.......)).)))).))..)))))))))))))))))..))))).))).)))).))))))))))).))))))).
......XXXXXXXXXXXXXXXXXXXXX......................................................................................*********************.....
...TTTTTCCCAGCTCCGACGG.....................................................................................................................x1.(0.0%)
...TTTTTCCCAGCTCCGACGGGATGG................................................................................................................x3.(0.0%)
...TTTTTCCCAGCTCCGACGGGA...................................................................................................................x12.(0.01%)
...TTTTTCCCAGCTCCGACGGGAT..................................................................................................................x2.(0.0%)
....TTTTCCCAGCTCCGACGGGATGG................................................................................................................x3.(0.0%)
....TTTTCCCAGCTCCGACGGGA...................................................................................................................x1.(0.0%)
....TTTTCCCAGCTCCGACGGGAT..................................................................................................................x9.(0.01%)
....TTTTCCCAGCTCCGACGGGATG.................................................................................................................x1.(0.0%)
.....TTTCCCAGCTCCGACGGGATG.................................................................................................................x21.(0.02%)
.....TTTCCCAGCTCCGACGGGATGG................................................................................................................x137.(0.12%)
.....TTTCCCAGCTCCGACGGGA...................................................................................................................x17.(0.01%)
.....TTTCCCAGCTCCGACGGGAT..................................................................................................................x11.(0.01%)
......TTCCCAGCTCCGACGGGA...................................................................................................................x580.(0.49%)
......TTCCCAGCTCCGACGGGATGG................................................................................................................x87814.(74.3%)*
......TTCCCAGCTCCGACGGGATGGC...............................................................................................................x373.(0.32%)
......TTCCCAGCTCCGACGGGATG.................................................................................................................x2291.(1.94%)
......TTCCCAGCTCCGACGGGATGGCA..............................................................................................................x4.(0.0%)
......TTCCCAGCTCCGACGGGAT..................................................................................................................x176.(0.15%)
.......TCCCAGCTCCGACGGGATGG................................................................................................................x971.(0.82%)
.......TCCCAGCTCCGACGGGAT..................................................................................................................x5.(0.0%)
.......TCCCAGCTCCGACGGGATGGC...............................................................................................................x20.(0.02%)
.......TCCCAGCTCCGACGGGATG.................................................................................................................x20.(0.02%)
.......TCCCAGCTCCGACGGGATGGCA..............................................................................................................x1.(0.0%)
........CCCAGCTCCGACGGGATG.................................................................................................................x1.(0.0%)
........CCCAGCTCCGACGGGATGG................................................................................................................x45.(0.04%)
.........CCAGCTCCGACGGGATGG................................................................................................................x329.(0.28%)
..........CAGCTCCGACGGGATGGCACT............................................................................................................x1.(0.0%)
..............TCCGACGGGATGGCACTCGTG........................................................................................................x2.(0.0%)
```

```
........................TGGCACTCGTGACTAGTTCCGCCG............................................................................................x1.(0.0%)
..........................GCACTCGTGACTAGTTCCGCCG............................................................................................x1.(0.0%)
..........................GCACTCGTGACTAGTTCCGCC.............................................................................................x1.(0.0%)
...........................CACTCGTGACTAGTTCCG...............................................................................................x6.(0.01%)
...........................CACTCGTGACTAGTTCCGCCGC...........................................................................................x122.(0.1%)
...........................CACTCGTGACTAGTTCCGC..............................................................................................x15.(0.01%)
...........................CACTCGTGACTAGTTCCGCC.............................................................................................x126.(0.11%)
...........................CACTCGTGACTAGTTCCGCCG............................................................................................x884.(0.75%)
............................ACTCGTGACTAGTTCCGCCG............................................................................................x6.(0.01%)
..............................TCGTGACTAGTTCCGCCGCCT.........................................................................................x7.(0.01%)
........................................TTCCGCCGCCTTGTATCACAC...............................................................................x1.(0.0%)
..........................................................CACGAGCGTGTGTATCCCCGTGCGA.........................................................x1.(0.0%)
....................................................................TGTATCCCCGTGCGACTCAAGGCG................................................x1.(0.0%)
......................................................................TATCCCCGTGCGACTCAAGG..................................................x1.(0.0%)
......................................................................TATCCCCGTGCGACTCAAGGC.................................................x1.(0.0%)
......................................................................TATCCCCGTGCGACTCAAGGCG................................................x1.(0.0%)
..............................................................................TGCGACTCAAGGCGGCGGAACT........................................x1.(0.0%)
..............................................................................TGCGACTCAAGGCGGCGG............................................x1.(0.0%)
............................................................................................GCGGAACTAGCGACGAGCGCC...........................x335.(0.28%)
............................................................................................GCGGAACTAGCGACGAGCG.............................x22.(0.02%)
............................................................................................GCGGAACTAGCGACGAGC..............................x11.(0.01%)
............................................................................................GCGGAACTAGCGACGAGCGC............................x83.(0.07%)
............................................................................................GCGGAACTAGCGACGAGCGCCC..........................x1.(0.0%)
.............................................................................................CGGAACTAGCGACGAGCGCCC..........................x8.(0.01%)
.............................................................................................CGGAACTAGCGACGAGCG.............................x1.(0.0%)
...............................................................................................GAACTAGCGACGAGCGCC...........................x1.(0.0%)
................................................................................................................CCCTTCCATCGGAGCTGGGTA.......x2.(0.0%)
.................................................................................................................CCTTCCATCGGAGCTGGGTAG......x7.(0.01%)
.................................................................................................................CCTTCCATCGGAGCTGGGTAGA.....x2.(0.0%)
..................................................................................................................CTTCCATCGGAGCTGGGT........x27.(0.02%)
..................................................................................................................CTTCCATCGGAGCTGGGTAG......x386.(0.33%)
..................................................................................................................CTTCCATCGGAGCTGGGTAGA.....x11314.(9.57%)*
..................................................................................................................CTTCCATCGGAGCTGGGTA.......x261.(0.22%)
..................................................................................................................CTTCCATCGGAGCTGGGTAGAAT...x16.(0.01%)
..................................................................................................................CTTCCATCGGAGCTGGGTAGAATT..x9.(0.01%)
..................................................................................................................CTTCCATCGGAGCTGGGTAGAA....x3234.(2.74%)
...................................................................................................................TTCCATCGGAGCTGGGTA.......x93.(0.08%)
...................................................................................................................TTCCATCGGAGCTGGGTAGAATT..x41.(0.03%)
...................................................................................................................TTCCATCGGAGCTGGGTAGAA....x6374.(5.39%)
...................................................................................................................TTCCATCGGAGCTGGGTAGA.....x710.(0.6%)
...................................................................................................................TTCCATCGGAGCTGGGTAGAAT...x120.(0.1%)
...................................................................................................................TTCCATCGGAGCTGGGTAG......x136.(0.12%)
....................................................................................................................TCCATCGGAGCTGGGTAGA.....x19.(0.02%)
....................................................................................................................TCCATCGGAGCTGGGTAGAATT..x91.(0.08%)
....................................................................................................................TCCATCGGAGCTGGGTAGAAT...x752.(0.64%)
....................................................................................................................TCCATCGGAGCTGGGTAG......x1.(0.0%)
....................................................................................................................TCCATCGGAGCTGGGTAGAA....x77.(0.07%)
.....................................................................................................................CCATCGGAGCTGGGTAGAA....x2.(0.0%)
.....................................................................................................................CCATCGGAGCTGGGTAGA.....x1.(0.0%)
......................................................................................................................CATCGGAGCTGGGTAGAATT..x9.(0.01%)
......................................................................................................................CATCGGAGCTGGGTAGAATTC.x2.(0.0%)
......................................................................................................................CATCGGAGCTGGGTAGAA....x3.(0.0%)
......................................................................................................................CATCGGAGCTGGGTAGAAT...x6.(0.01%)
.......................................................................................................................ATCGGAGCTGGGTAGAAT...x1.(0.0%)
```

Complementarity = 17

Folding energy = -94.50

Most common read: UUCCCAGCUCCGACGGGAUGG (freq 87814) matching esi-miR3454b-5p (e-value: 0.002)

```
Query  1   UUCCCAGCUCCGACGGGAUGG  21
           |||||||||||||||||||||
Sbjct  1   UUCCCAGCUCCGACGGGAUGG  21
```

```
        10         20         30         40         50         60
-       U           C    U    A     GA                 UA  A    A C UGU
 GAGUUUU CCCAGCUCCGA GGGA GGC CUCGU  CUAGUUCCGCCGCCUUG  UC CACG G G   \
 CUUAAGA GGGUCGAGGCU CCUU CCG GAGCA  GAUCAAGGCGGCGGAAC  AG GUGC C C   G
A       U           A    C    C     GC                 UC  C    C - UAU
      130        120        110        100         90         80
```

---

esi-MIR3454d

```
CGCGGAGTTCTTCCAAGCTCCGACGGGATGGCGATCGTGACTAGGTCCGCCGCCTTGTATCACACGAGCGTGTATGTCCGTGCGACTCAAGGCGGCAGAACTAGCCACGGGCACCCTTCCATCGGAGCTCGTCAGAATGCCACGA
((.((.(((((..(.((((((((.((((.((.(..((((.((((.((.(((((((((..((.((((..((....))..)))).))..)))))))))).)).)))).)))).).)).)))).)))))))).)..))))).)).)).
..........XXXXXXXXXXXXXXXXXXXXX..................................................................................******************............
.......TTCTTCCAAGCTCCGACGGGA.....................................................................................................................x3.(0.0%)
.......TTCTTCCAAGCTCCGACGG.......................................................................................................................x2.(0.0%)
........TCTTCCAAGCTCCGACGGGAT....................................................................................................................x9.(0.01%)
.........CTTCCAAGCTCCGACGGGATGG..................................................................................................................x28.(0.02%)
.........CTTCCAAGCTCCGACGGGAT....................................................................................................................x2.(0.0%)
.........CTTCCAAGCTCCGACGGGATG...................................................................................................................x4.(0.0%)
..........TTCCAAGCTCCGACGGGATGG..................................................................................................................x115339.(89.24%)*
..........TTCCAAGCTCCGACGGGATGGC.................................................................................................................x373.(0.29%)
..........TTCCAAGCTCCGACGGGAT....................................................................................................................x1989.(1.54%)
..........TTCCAAGCTCCGACGGGATG...................................................................................................................x7143.(5.53%)
..........TTCCAAGCTCCGACGGGA.....................................................................................................................x873.(0.68%)
...........TCCAAGCTCCGACGGGATGGCGA...............................................................................................................x8.(0.01%)
...........TCCAAGCTCCGACGGGATG...................................................................................................................x89.(0.07%)
...........TCCAAGCTCCGACGGGAT....................................................................................................................x80.(0.06%)
...........TCCAAGCTCCGACGGGATGGCG................................................................................................................x19.(0.01%)
...........TCCAAGCTCCGACGGGATGG..................................................................................................................x1101.(0.85%)
...........TCCAAGCTCCGACGGGATGGC.................................................................................................................x600.(0.46%)
...........TCCAAGCTCCGACGGGATGGCGAT..............................................................................................................x1.(0.0%)
............CCAAGCTCCGACGGGATGG..................................................................................................................x34.(0.03%)
............CCAAGCTCCGACGGGATG...................................................................................................................x1.(0.0%)
.............CAAGCTCCGACGGGATGGC.................................................................................................................x8.(0.01%)
.............CAAGCTCCGACGGGATGG..................................................................................................................x201.(0.16%)
...............................CGATCGTGACTAGGTCCGCCG.............................................................................................x69.(0.05%)
...............................CGATCGTGACTAGGTCCGCC..............................................................................................x3.(0.0%)
...............................CGATCGTGACTAGGTCCGCCGC............................................................................................x5.(0.0%)
................................GATCGTGACTAGGTCCGCC..............................................................................................x1.(0.0%)
................................GATCGTGACTAGGTCCGCCG.............................................................................................x1.(0.0%)
..................................TCGTGACTAGGTCCGCCGCC...........................................................................................x4.(0.0%)
..................................TCGTGACTAGGTCCGCCGC............................................................................................x1.(0.0%)
..................................TCGTGACTAGGTCCGCCGCCT..........................................................................................x27.(0.02%)
.....................................TGACTAGGTCCGCCGCCTTG........................................................................................x4.(0.0%)
.....................................TGACTAGGTCCGCCGCCTT.........................................................................................x1.(0.0%)
....................................................CCTTGTATCACACGAGCGTGTA.......................................................................x2.(0.0%)
..............................................................................................GCAGAACTAGCCACGGGCAC...............................x69.(0.05%)
..............................................................................................GCAGAACTAGCCACGGGCA................................x9.(0.01%)
..............................................................................................GCAGAACTAGCCACGGGCACCC.............................x3.(0.0%)
..............................................................................................GCAGAACTAGCCACGGGC.................................x9.(0.01%)
..............................................................................................GCAGAACTAGCCACGGGCACCCT............................x1.(0.0%)
..............................................................................................GCAGAACTAGCCACGGGCACC..............................x646.(0.5%)*
...............................................................................................CAGAACTAGCCACGGGCACC..............................x15.(0.01%)
...............................................................................................CAGAACTAGCCACGGGCACCC.............................x36.(0.03%)
...............................................................................................CAGAACTAGCCACGGGCACCCT............................x1.(0.0%)
...............................................................................................CAGAACTAGCCACGGGCAC...............................x1.(0.0%)
................................................................................................AGAACTAGCCACGGGCACCC.............................x1.(0.0%)
........................................................................................................CCACGGGCACCCTTCCATCGG....................x1.(0.0%)
...............................................................................................................CACCCTTCCATCGGAGCTCGT.............x1.(0.0%)
...................................................................................................................CTTCCATCGGAGCTCGTCAGAA........x29.(0.02%)
...................................................................................................................CTTCCATCGGAGCTCGTCAG..........x5.(0.0%)
```

```
...................................................................................................................CTTCCATCGGAGCTCGTCAGA.........x191.(0.15%)
...................................................................................................................CTTCCATCGGAGCTCGTCAGAAT.......x1.(0.0%)
...................................................................................................................CTTCCATCGGAGCTCGTCA...........x6.(0.0%)
....................................................................................................................TTCCATCGGAGCTCGTCAGAAT.......x12.(0.01%)
....................................................................................................................TTCCATCGGAGCTCGTCAGAA........x140.(0.11%)
....................................................................................................................TTCCATCGGAGCTCGTCA...........x1.(0.0%)
....................................................................................................................TTCCATCGGAGCTCGTCAGA.........x14.(0.01%)
....................................................................................................................TTCCATCGGAGCTCGTCAG..........x3.(0.0%)
.....................................................................................................................TCCATCGGAGCTCGTCAGAA........x1.(0.0%)
.....................................................................................................................TCCATCGGAGCTCGTCAGAAT.......x27.(0.02%)
.......................................................................................................................CATCGGAGCTCGTCAGAA........x1.(0.0%)
.........................................................................................................................TCGGAGCTCGTCAGAATGCCA...x1.(0.0%)
.........................................................................................................................TCGGAGCTCGTCAGAATGCC....x1.(0.0%)
```

Complementarity = 15

Folding energy = -77.80

```
Query  1   UUCCAAGCUCCGACGGGAUGG  21
           |||||||||||||||||||||
Sbjct  1   UUCCAAGCUCCGACGGGAUGG  21
```

```
         10        20        30        40        50        60        70
-  C  A     UC A        C    U  C AU    A    G  C         UA  A    AG  U
 CG GG GUUCU  C AGCUCCGA GGGA GG G  CGUG CUAG UC GCCGCCUUG  UC CACG  CG G
 GC CC UAAGA  G UCGAGGCU CCUU CC C  GCAC GAUC AG CGGCGGAAC  AG GUGC  GU U
A  A  G     CU C        A    C  A GG    C    A  A         UC  C    CU  A
   140       130       120       110       100        90        80
```

```
esi-MIR3454e
GGTGGAATTCTTCCCAGCTCCGACGGAATGGCGCTCGTGGTTAGTTCGGCCGTCTTGTATCACGCGAGCGTTTGTATACCCGTGCGACTCAAGGCGGCGGAACTAGTCACGAGCGCCCTTCCATCGGAGCTAGGTAGAATCTCACCA
((((..(((((.((.((((((((.((((.((((((((((..((((((.(((((((((..((.((((.(.((......))))))).))..))))))))).))))))..)))))))))).)))).)))))))).)).)))))..)))).
..........XXXXXXXXXXXXXXXXXXXXX....................................................................................*********************.........
.......TTCTTCCCAGCTCCGACGGAA.......................................................................................................................x20.(0.01%)
........TCTTCCCAGCTCCGACGGA........................................................................................................................x2.(0.0%)
........TCTTCCCAGCTCCGACGGAATG.....................................................................................................................x2.(0.0%)
........TCTTCCCAGCTCCGACGGAAT......................................................................................................................x256.(0.07%)
........TCTTCCCAGCTCCGACGGAA.......................................................................................................................x13.(0.0%)
........TCTTCCCAGCTCCGACGG.........................................................................................................................x3.(0.0%)
.........CTTCCCAGCTCCGACGGAATGG....................................................................................................................x146.(0.04%)
.........CTTCCCAGCTCCGACGGAAT......................................................................................................................x6.(0.0%)
.........CTTCCCAGCTCCGACGGAATG.....................................................................................................................x20.(0.01%)
.........CTTCCCAGCTCCGACGGA........................................................................................................................x3.(0.0%)
..........TTCCCAGCTCCGACGGAATG.....................................................................................................................x10543.(2.83%)
..........TTCCCAGCTCCGACGGAAT......................................................................................................................x3728.(1.0%)
..........TTCCCAGCTCCGACGGAATGGC...................................................................................................................x646.(0.17%)
..........TTCCCAGCTCCGACGGAATGGCG..................................................................................................................x3.(0.0%)
..........TTCCCAGCTCCGACGGAATGG....................................................................................................................x344355.(92.48%)*
..........TTCCCAGCTCCGACGGAA.......................................................................................................................x1113.(0.3%)
...........TCCCAGCTCCGACGGAAT......................................................................................................................x239.(0.06%)
...........TCCCAGCTCCGACGGAATGGCG..................................................................................................................x68.(0.02%)
...........TCCCAGCTCCGACGGAATGGC...................................................................................................................x675.(0.18%)
...........TCCCAGCTCCGACGGAATG.....................................................................................................................x457.(0.12%)
...........TCCCAGCTCCGACGGAATGG....................................................................................................................x4686.(1.26%)
............CCCAGCTCCGACGGAATGG....................................................................................................................x94.(0.03%)
............CCCAGCTCCGACGGAATG.....................................................................................................................x4.(0.0%)
.............CCAGCTCCGACGGAATGGC...................................................................................................................x29.(0.01%)
.............CCAGCTCCGACGGAATGG....................................................................................................................x1662.(0.45%)
..............CAGCTCCGACGGAATGGCGCT................................................................................................................x5.(0.0%)
..................TCCGACGGAATGGCGCTCGT.............................................................................................................x2.(0.0%)
```

```
..................TCCGACGGAATGGCGCTCGTG............................................................................................................x5.(0.0%)
............................TGGCGCTCGTGGTTAGTTCGGC.................................................................................................x2.(0.0%)
............................TGGCGCTCGTGGTTAGTTCGG..................................................................................................x4.(0.0%)
...............................CGCTCGTGGTTAGTTCGGCC................................................................................................x41.(0.01%)
...............................CGCTCGTGGTTAGTTCGGC.................................................................................................x12.(0.0%)
...............................CGCTCGTGGTTAGTTCGGCCG...............................................................................................x287.(0.08%)
...............................CGCTCGTGGTTAGTTCGG..................................................................................................x58.(0.02%)
..................................................................AGCGTTTGTATACCCGTGCGA............................................................x2.(0.0%)
..........................................................................TATACCCGTGCGACTCAAGGC....................................................x5.(0.0%)
..........................................................................TATACCCGTGCGACTCAAGG.....................................................x2.(0.0%)
..........................................................................TATACCCGTGCGACTCAAGGCG...................................................x3.(0.0%)
........................................................................................TCAAGGCGGCGGAACTAGTCA......................................x8.(0.0%)
................................................................................................GCGGAACTAGTCACGAGC.................................x22.(0.01%)
................................................................................................GCGGAACTAGTCACGAGCGCCC.............................x68.(0.02%)
................................................................................................GCGGAACTAGTCACGAGCG................................x20.(0.01%)
................................................................................................GCGGAACTAGTCACGAGCGCC..............................x1521.(0.41%)*
................................................................................................GCGGAACTAGTCACGAGCGC...............................x180.(0.05%)
.................................................................................................CGGAACTAGTCACGAGCGCC..............................x12.(0.0%)
.................................................................................................CGGAACTAGTCACGAGCGCCC.............................x60.(0.02%)
.................................................................................................CGGAACTAGTCACGAGCGC...............................x3.(0.0%)
..................................................................................................GGAACTAGTCACGAGCGCC..............................x3.(0.0%)
..................................................................................................GGAACTAGTCACGAGCGCCC.............................x2.(0.0%)
...................................................................................................GAACTAGTCACGAGCGCC..............................x2.(0.0%)
..........................................................................................................TCACGAGCGCCCTTCCATCGG....................x5.(0.0%)
...................................................................................................................CCCTTCCATCGGAGCTAGGTA...........x2.(0.0%)
.....................................................................................................................CTTCCATCGGAGCTAGGTA...........x35.(0.01%)
.....................................................................................................................CTTCCATCGGAGCTAGGTAGAA........x71.(0.02%)
.....................................................................................................................CTTCCATCGGAGCTAGGTAGA.........x850.(0.23%)
.....................................................................................................................CTTCCATCGGAGCTAGGT............x7.(0.0%)
.....................................................................................................................CTTCCATCGGAGCTAGGTAG..........x30.(0.01%)
......................................................................................................................TTCCATCGGAGCTAGGTAGA.........x18.(0.0%)
......................................................................................................................TTCCATCGGAGCTAGGTA...........x4.(0.0%)
......................................................................................................................TTCCATCGGAGCTAGGTAGAA........x181.(0.05%)
.......................................................................................................................TCCATCGGAGCTAGGTAGAAT.......x24.(0.01%)
.......................................................................................................................TCCATCGGAGCTAGGTAGAA........x2.(0.0%)
```

Complementarity = 16

Folding energy = -99.60

Most common read: UUCCCAGCUCCGACGGAAUGG (freq 344355) matching esi-miR3454e-5p (e-value: 0.002)

```
Query  1   UUCCCAGCUCCGACGGAAUGG  21
           |||||||||||||||||||||
Sbjct  1   UUCCCAGCUCCGACGGAAUGG  21
```

```
         10        20        30        40        50        60        70
-    GA     U  C        C    U          GU      G         UA  A    A C  UU
 GGUG  AUUCU CC AGCUCCGA GGAA GGCGCUCGUG  UAGUUC GCCGUCUUG  UC CGCG G GU  G
 CCAC  UAAGA GG UCGAGGCU CCUU CCGCGAGCAC  AUCAAG CGGCGGAAC  AG GUGC C CA  U
A    UC     U  A        A    C          UG      G         UC  C    - -  UA
     140       130       120       110       100        90         80
```

____

esi-MIR3454f

```
CGCGGAGTTCTTCCAAGCTCCGACGGGATGGCGATCGTGACTAGGTCCGCCGCCTTGTATCACACGAGCGTGGGTACACCCGTGCGACTCAAGGCGGCGGAACTAGCCACGAGCGTCCTTCCGTCGGAGCTGGGTAGAATTTCACGA
((.((((((((.((.(((((((((((((.((((.(((((.((((.((((((((((((..((.((((.(.(((....)))))))).))..)))))))))))).)))).))))).)))).))))))))))))).)).)))))))).)).
..........XXXXXXXXXXXXXXXXXXXXX......................................................................................*********************.........
.......TTCTTCCAAGCTCCGACGGGA.......................................................................................................................x3.(0.0%)
.......TTCTTCCAAGCTCCGACGG.........................................................................................................................x2.(0.0%)
........TCTTCCAAGCTCCGACGGGAT......................................................................................................................x9.(0.01%)
```

```
.........CTTCCAAGCTCCGACGGGATGG.....................................................................................................................x28.(0.02%)
.........CTTCCAAGCTCCGACGGGAT.......................................................................................................................x2.(0.0%)
.........CTTCCAAGCTCCGACGGGATG......................................................................................................................x4.(0.0%)
..........TTCCAAGCTCCGACGGGATGG.....................................................................................................................x115339.(89.13%)*
..........TTCCAAGCTCCGACGGGATGGC....................................................................................................................x373.(0.29%)
..........TTCCAAGCTCCGACGGGAT.......................................................................................................................x1989.(1.54%)
..........TTCCAAGCTCCGACGGGATG......................................................................................................................x7143.(5.52%)
..........TTCCAAGCTCCGACGGGA........................................................................................................................x873.(0.67%)
...........TCCAAGCTCCGACGGGATGGCGA..................................................................................................................x8.(0.01%)
...........TCCAAGCTCCGACGGGATGGCGAT.................................................................................................................x1.(0.0%)
...........TCCAAGCTCCGACGGGATGGC....................................................................................................................x600.(0.46%)
...........TCCAAGCTCCGACGGGATG......................................................................................................................x89.(0.07%)
...........TCCAAGCTCCGACGGGAT.......................................................................................................................x80.(0.06%)
...........TCCAAGCTCCGACGGGATGG.....................................................................................................................x1101.(0.85%)
...........TCCAAGCTCCGACGGGATGGCG...................................................................................................................x19.(0.01%)
............CCAAGCTCCGACGGGATGG.....................................................................................................................x34.(0.03%)
............CCAAGCTCCGACGGGATG......................................................................................................................x1.(0.0%)
.............CAAGCTCCGACGGGATGGC....................................................................................................................x8.(0.01%)
.............CAAGCTCCGACGGGATGG.....................................................................................................................x201.(0.16%)
...............................CGATCGTGACTAGGTCCGCCGC...............................................................................................x5.(0.0%)
...............................CGATCGTGACTAGGTCCGCC.................................................................................................x3.(0.0%)
...............................CGATCGTGACTAGGTCCGCCG................................................................................................x69.(0.05%)
................................GATCGTGACTAGGTCCGCC.................................................................................................x1.(0.0%)
................................GATCGTGACTAGGTCCGCCG................................................................................................x1.(0.0%)
..................................TCGTGACTAGGTCCGCCGCC..............................................................................................x4.(0.0%)
..................................TCGTGACTAGGTCCGCCGC...............................................................................................x1.(0.0%)
..................................TCGTGACTAGGTCCGCCGCCT.............................................................................................x27.(0.02%)
.....................................TGACTAGGTCCGCCGCCTTG...........................................................................................x4.(0.0%)
.....................................TGACTAGGTCCGCCGCCTT............................................................................................x1.(0.0%)
........................................................................GGGTACACCCGTGCGACTCAAGGC....................................................x2.(0.0%)
...........................................................................TACACCCGTGCGACTCAAGGC....................................................x1.(0.0%)
...................................................................................TGCGACTCAAGGCGGCGG...............................................x1.(0.0%)
...................................................................................TGCGACTCAAGGCGGCGGAACT...........................................x1.(0.0%)
.................................................................................................GCGGAACTAGCCACGAGCGTC..............................x171.(0.13%)
.................................................................................................GCGGAACTAGCCACGAGCGT...............................x41.(0.03%)
.................................................................................................GCGGAACTAGCCACGAGC.................................x10.(0.01%)
.................................................................................................GCGGAACTAGCCACGAGCG................................x4.(0.0%)
..................................................................................................CGGAACTAGCCACGAGCGTCC.............................x1.(0.0%)
..................................................................................................................CGTCCTTCCGTCGGAGCTGGG.............x2.(0.0%)
....................................................................................................................TCCTTCCGTCGGAGCTGG..............x1.(0.0%)
....................................................................................................................TCCTTCCGTCGGAGCTGGGTA...........x39.(0.03%)
....................................................................................................................TCCTTCCGTCGGAGCTGGGTAG..........x26.(0.02%)
....................................................................................................................TCCTTCCGTCGGAGCTGGGT............x14.(0.01%)
....................................................................................................................TCCTTCCGTCGGAGCTGGG.............x1.(0.0%)
....................................................................................................................TCCTTCCGTCGGAGCTGGGTAGA.........x2.(0.0%)
.....................................................................................................................CCTTCCGTCGGAGCTGGGTAG..........x4.(0.0%)
.....................................................................................................................CCTTCCGTCGGAGCTGGG.............x9.(0.01%)
.....................................................................................................................CCTTCCGTCGGAGCTGGGTA...........x2.(0.0%)
......................................................................................................................CTTCCGTCGGAGCTGGGTA...........x26.(0.02%)
......................................................................................................................CTTCCGTCGGAGCTGGGT............x7.(0.01%)
......................................................................................................................CTTCCGTCGGAGCTGGGTAG..........x25.(0.02%)
......................................................................................................................CTTCCGTCGGAGCTGGGTAGAA........x40.(0.03%)
......................................................................................................................CTTCCGTCGGAGCTGGGTAGA.........x392.(0.3%)*
......................................................................................................................CTTCCGTCGGAGCTGGGTAGAATT......x1.(0.0%)
.......................................................................................................................TTCCGTCGGAGCTGGGTAGAA........x264.(0.2%)
.......................................................................................................................TTCCGTCGGAGCTGGGTAGAAT.......x23.(0.02%)
.......................................................................................................................TTCCGTCGGAGCTGGGTAGA.........x67.(0.05%)
.......................................................................................................................TTCCGTCGGAGCTGGGTA...........x7.(0.01%)
.......................................................................................................................TTCCGTCGGAGCTGGGTAGAATT......x5.(0.0%)
.......................................................................................................................TTCCGTCGGAGCTGGGTAG..........x5.(0.0%)
........................................................................................................................TCCGTCGGAGCTGGGTAGA.........x1.(0.0%)
........................................................................................................................TCCGTCGGAGCTGGGTAGAATT......x10.(0.01%)
........................................................................................................................TCCGTCGGAGCTGGGTAGAA........x2.(0.0%)
........................................................................................................................TCCGTCGGAGCTGGGTAGAATTT.....x1.(0.0%)
```

```
.......................................................................................................................TCCGTCGGAGCTGGGTAGAAT.......x37.(0.03%)
...........................................................................................................................TCGGAGCTGGGTAGAATTTCA...x71.(0.05%)
...........................................................................................................................TCGGAGCTGGGTAGAATTTC....x12.(0.01%)
...........................................................................................................................TCGGAGCTGGGTAGAATTTCACG.x1.(0.0%)
...........................................................................................................................TCGGAGCTGGGTAGAATTT.....x1.(0.0%)
............................................................................................................................CGGAGCTGGGTAGAATTTC....x1.(0.0%)
.............................................................................................................................GGAGCTGGGTAGAATTTCACG.x40.(0.03%)
.............................................................................................................................GGAGCTGGGTAGAATTTCACGAx4.(0.0%)
.............................................................................................................................GGAGCTGGGTAGAATTTCAC..x4.(0.0%)
..............................................................................................................................GAGCTGGGTAGAATTTCACGAx1.(0.0%)
..............................................................................................................................GAGCTGGGTAGAATTTCACG.x1.(0.0%)
```

Complementarity = 17

Folding energy = -99.00

Most common read: UUCCAAGCUCCGACGGGAUGG (freq 115339) matching esi-miR3454d-5p (e-value: 0.002)

```
Query  1   UUCCAAGCUCCGACGGGAUGG  21
           |||||||||||||||||||||
Sbjct  1   UUCCAAGCUCCGACGGGAUGG  21
```

```
         10        20        30        40        50        60        70
-  C        U  A             U    A     A    G            UA  A    A C   G
 CG GGAGUUCU CC AGCUCCGACGGGA GGCG UCGUG CUAG UCCGCCGCCUUG  UC CACG G GUG G
 GC CUUUAAGA GG UCGAGGCUGCCUU CUGC AGCAC GAUC AGGCGGCGGAAC  AG GUGC C CAC U
A  A        U  G             C    G     C    A            UC  C    - -   A
     140       130       120       110       100        90         80
```

```
esi-MIR3454g
GTGGAATTCTACCCAGCTCCGACGGAATGGCGCTCGTGGCTAGGTCCCCGCCGCCTTGTATCACACGAGCGTGTATGTCCGTGCGACTCAAGGCGGCAGAACTAGCCACGGGCACCCTTCCATCGGAGCTCGTCAGAATGCCACG
((((.(((((..(.((((((((.((((.((.((((((((((((.((...(((((((((..((.((((..((....))..)))).))..))))))))).)).)))))))))))).)).)))).)))))))).)..))))).)))).
.........XXXXXXXXXXXXXXXXXXXXX...................................................................................................................
....................................................................................................................******************...........
......TTCTACCCAGCTCCGACGGA.......................................................................................................................x1.(0.0%)
......TTCTACCCAGCTCCGACGGAA......................................................................................................................x2.(0.0%)
.......TCTACCCAGCTCCGACGGAAT.....................................................................................................................x42.(0.08%)
........CTACCCAGCTCCGACGGAATG....................................................................................................................x1.(0.0%)
........CTACCCAGCTCCGACGGAATGG...................................................................................................................x16.(0.03%)
.........TACCCAGCTCCGACGGAATGG...................................................................................................................x44713.(87.33%)*
.........TACCCAGCTCCGACGGAATG....................................................................................................................x2182.(4.26%)
.........TACCCAGCTCCGACGGAA......................................................................................................................x89.(0.17%)
.........TACCCAGCTCCGACGGAAT.....................................................................................................................x824.(1.61%)
.........TACCCAGCTCCGACGGAATGGC..................................................................................................................x76.(0.15%)
..........ACCCAGCTCCGACGGAATG....................................................................................................................x9.(0.02%)
..........ACCCAGCTCCGACGGAATGG...................................................................................................................x212.(0.41%)
..........ACCCAGCTCCGACGGAAT.....................................................................................................................x4.(0.01%)
..........ACCCAGCTCCGACGGAATGGC..................................................................................................................x1.(0.0%)
...........CCCAGCTCCGACGGAATGG...................................................................................................................x94.(0.18%)
...........CCCAGCTCCGACGGAATG....................................................................................................................x4.(0.01%)
............CCAGCTCCGACGGAATGGCG.................................................................................................................x1.(0.0%)
............CCAGCTCCGACGGAATGGC..................................................................................................................x29.(0.06%)
............CCAGCTCCGACGGAATGG...................................................................................................................x1662.(3.25%)
.............CAGCTCCGACGGAATGGCGCT...............................................................................................................x5.(0.01%)
.................TCCGACGGAATGGCGCTCGT............................................................................................................x2.(0.0%)
.................TCCGACGGAATGGCGCTCGTG...........................................................................................................x5.(0.01%)
.....................................................CCTTGTATCACACGAGCGTGTA......................................................................x2.(0.0%)
...............................................................................................GCAGAACTAGCCACGGGCACCC............................x3.(0.01%)
...............................................................................................GCAGAACTAGCCACGGGCAC..............................x69.(0.13%)
...............................................................................................GCAGAACTAGCCACGGGCA...............................x9.(0.02%)
...............................................................................................GCAGAACTAGCCACGGGC................................x9.(0.02%)
```

```
...............................................................................................GCAGAACTAGCCACGGGCACCCT...........................x1.(0.0%)
...............................................................................................GCAGAACTAGCCACGGGCACC.............................x646.(1.26%)*
................................................................................................CAGAACTAGCCACGGGCACCC............................x36.(0.07%)
................................................................................................CAGAACTAGCCACGGGCAC..............................x1.(0.0%)
................................................................................................CAGAACTAGCCACGGGCACCCT...........................x1.(0.0%)
................................................................................................CAGAACTAGCCACGGGCACC.............................x15.(0.03%)
.................................................................................................AGAACTAGCCACGGGCACCC............................x1.(0.0%)
.........................................................................................................CCACGGGCACCCTTCCATCGG...................x1.(0.0%)
................................................................................................................CACCCTTCCATCGGAGCTCGT............x1.(0.0%)
....................................................................................................................CTTCCATCGGAGCTCGTCAGAA.......x29.(0.06%)
....................................................................................................................CTTCCATCGGAGCTCGTCAG.........x5.(0.01%)
....................................................................................................................CTTCCATCGGAGCTCGTCA..........x6.(0.01%)
....................................................................................................................CTTCCATCGGAGCTCGTCAGA........x191.(0.37%)
....................................................................................................................CTTCCATCGGAGCTCGTCAGAAT......x1.(0.0%)
.....................................................................................................................TTCCATCGGAGCTCGTCAG.........x3.(0.01%)
.....................................................................................................................TTCCATCGGAGCTCGTCAGAA.......x140.(0.27%)
.....................................................................................................................TTCCATCGGAGCTCGTCAGAAT......x12.(0.02%)
.....................................................................................................................TTCCATCGGAGCTCGTCA..........x1.(0.0%)
.....................................................................................................................TTCCATCGGAGCTCGTCAGA........x14.(0.03%)
......................................................................................................................TCCATCGGAGCTCGTCAGAA.......x1.(0.0%)
......................................................................................................................TCCATCGGAGCTCGTCAGAAT......x27.(0.05%)
........................................................................................................................CATCGGAGCTCGTCAGAA.......x1.(0.0%)
..........................................................................................................................TCGGAGCTCGTCAGAATGCC...x1.(0.0%)
..........................................................................................................................TCGGAGCTCGTCAGAATGCCA..x1.(0.0%)
```

Complementarity = 15

Folding energy = -91.00

Most common read: UACCCAGCUCCGACGGAAUGG (freq 44713) matching esi-miR3454g-5p (e-value: 0.002)

```
Query  1   UACCCAGCUCCGACGGAAUGG  21
           |||||||||||||||||||||
Sbjct  1   UACCCAGCUCCGACGGAAUGG  21
```

```
         10        20        30        40        50        60        70
-    A     AC C        C    U  C            G  CCC         UA  A    AG  U
 GUGG AUUCU  C AGCUCCGA GGAA GG GCUCGUGGCUAG UC   GCCGCCUUG  UC CACG  CG G
 CACC UAAGA  G UCGAGGCU CCUU CC CGGGCACCGAUC AG   CGGCGGAAC  AG GUGC  GU U
G    G     CU C        A    C  A            A  A--         UC  C    CU  A
   140       130       120       110       100          90        80
```

____

```
esi-MIR3455
CGGTTCTTGACCATCATTCTCAAGTTCCATCGGAGGTTGAATTCCAGCAACGCCGAGTCTTTGCTGGAGTTCAAACCTCCGGTGGAATCTGAGGATAGTGGCGAAGAACC
.(((((((..((((.(((((((..((((((((((((((((((((((((((.(......).)))))))))))))).))))))))))))))..))))))).))))..)))))))
.............XXXXXXXXXXXXXXXXXXXXX.............................................*********************..........
.............TCATTCTCAAGTTCCATCG..............................................................................x2.(0.02%)
.............TCATTCTCAAGTTCCATCGGAG...........................................................................x127.(1.52%)
.............TCATTCTCAAGTTCCATCGG.............................................................................x89.(1.07%)
.............TCATTCTCAAGTTCCATC...............................................................................x2.(0.02%)
.............TCATTCTCAAGTTCCATCGGA............................................................................x7633.(91.46%)*
..............CATTCTCAAGTTCCATCGGA............................................................................x26.(0.31%)
..............CATTCTCAAGTTCCATCGGAG...........................................................................x3.(0.04%)
...............ATTCTCAAGTTCCATCGGA............................................................................x16.(0.19%)
.................................................ACGCCGAGTCTTTGCTGGAGTTC......................................x1.(0.01%)
.............................................................................TCCGGTGGAATCTGAGGATA.............x2.(0.02%)
.............................................................................TCCGGTGGAATCTGAGGATAGTG..........x1.(0.01%)
.............................................................................TCCGGTGGAATCTGAGGATAG............x39.(0.47%)
..............................................................................CCGGTGGAATCTGAGGATAG............x1.(0.01%)
..............................................................................CCGGTGGAATCTGAGGATAGT...........x1.(0.01%)
```

```
..............................................................................CCGGTGGAATCTGAGGATAGTG..........x2.(0.02%)
...............................................................................CGGTGGAATCTGAGGATAGTG..........x345.(4.13%)*
...............................................................................CGGTGGAATCTGAGGATAGT...........x43.(0.52%)
...............................................................................CGGTGGAATCTGAGGATAG............x7.(0.08%)
...............................................................................CGGTGGAATCTGAGGATAGTGGC........x2.(0.02%)
................................................................................GGTGGAATCTGAGGATAGTG..........x1.(0.01%)
.................................................................................GTGGAATCTGAGGATAGTG..........x1.(0.01%)
..................................................................................TGGAATCTGAGGATAGTGGCG.......x2.(0.02%)
```

Complementarity = 17

Folding energy = -68.40

Most common read: UCAUUCUCAAGUUCCAUCGGA (freq 7633) matching esi-miR3455-5p (e-value: 0.002)

```
Query  1   UCAUUCUCAAGUUCCAUCGGA  21
           |||||||||||||||||||||
Sbjct  1   UCAUUCUCAAGUUCCAUCGGA  21
```

```
        10        20        30         40        50
C       GA    C       AG             -           C CC
 GGUUCUU  CCAU AUUCUCA  UUCCAUCGGAGGUU GAAUUCCAGCAA G  G
 CCAAGAA  GGUG UAGGAGU  AAGGUGGCCUCCAA CUUGAGGUCGUU C  A
-       GC    A       CU             A           U UG
 .       100        90        80        70        60
```

____

esi-MIR3456

```
GCCACCACTTGAGGAACGTCGCGGAGTCAGACCACGCGTTGGCCTGCGAGAAGTGCTTCAGAGGTGGA
.(((((.((.((((.((.((((((.(((((........)))))))))))...)).)))))).))))).
........XXXXXXXXXXXXXXXXXXXXX............*********************......
.CCACCACTTGAGGAACGTCG...............................................x1.(0.03%)
.CCACCACTTGAGGAACGTCGC..............................................x1.(0.03%)
..CACCACTTGAGGAACGTCGCG.............................................x1.(0.03%)
....CCACTTGAGGAACGTCGCGGA...........................................x6.(0.15%)
....CCACTTGAGGAACGTCGCGG............................................x1.(0.03%)
.....CACTTGAGGAACGTCGCGGAG..........................................x1.(0.03%)
.....CACTTGAGGAACGTCGCGGA...........................................x2.(0.05%)
......ACTTGAGGAACGTCGCGGAGT.........................................x1.(0.03%)
.......CTTGAGGAACGTCGCGGA...........................................x1.(0.03%)
.......CTTGAGGAACGTCGCGGAGTC........................................x60.(1.51%)
.......CTTGAGGAACGTCGCGGAGTCA.......................................x3.(0.08%)
.......CTTGAGGAACGTCGCGGAGT.........................................x20.(0.5%)
........TTGAGGAACGTCGCGGAGTCAGAC....................................x3.(0.08%)
........TTGAGGAACGTCGCGGAGTCAGA.....................................x5.(0.13%)
........TTGAGGAACGTCGCGGAG..........................................x4.(0.1%)
........TTGAGGAACGTCGCGGAGT.........................................x10.(0.25%)
........TTGAGGAACGTCGCGGAGTCA.......................................x2320.(58.22%)*
........TTGAGGAACGTCGCGGAGTCAG......................................x20.(0.5%)
........TTGAGGAACGTCGCGGAGTC........................................x62.(1.56%)
.........TGAGGAACGTCGCGGAGTC........................................x9.(0.23%)
.........TGAGGAACGTCGCGGAGTCA.......................................x131.(3.29%)
.........TGAGGAACGTCGCGGAGTCAGAC....................................x1.(0.03%)
.........TGAGGAACGTCGCGGAGTCAG......................................x1020.(25.6%)
.........TGAGGAACGTCGCGGAGTCAGA.....................................x95.(2.38%)
.........TGAGGAACGTCGCGGAGT.........................................x1.(0.03%)
..........GAGGAACGTCGCGGAGTCA.......................................x5.(0.13%)
..........GAGGAACGTCGCGGAGTCAGA.....................................x2.(0.05%)
..........GAGGAACGTCGCGGAGTCAG......................................x4.(0.1%)
...........AGGAACGTCGCGGAGTCA.......................................x1.(0.03%)
...........AGGAACGTCGCGGAGTCAGAC....................................x3.(0.08%)
```

```
...........AGGAACGTCGCGGAGTCAGA.....................................x1.(0.03%)
............GGAACGTCGCGGAGTCAGACC...................................x4.(0.1%)
............GGAACGTCGCGGAGTCAG......................................x1.(0.03%)
..................TCGCGGAGTCAGACCACGCGTT............................x2.(0.05%)
.....................CGGAGTCAGACCACGCGTTGG..........................x4.(0.1%)
.........................GTCAGACCACGCGTTGGC.........................x1.(0.03%)
..........................TCAGACCACGCGTTGGCC........................x1.(0.03%)
...........................CAGACCACGCGTTGGCCTGCGA...................x1.(0.03%)
..................................CGCGTTGGCCTGCGAGAAGTG.............x1.(0.03%)
......................................TTGGCCTGCGAGAAGTGCTTC.........x17.(0.43%)
......................................TTGGCCTGCGAGAAGTGCT...........x1.(0.03%)
......................................TTGGCCTGCGAGAAGTGCTTCAGA......x1.(0.03%)
......................................TTGGCCTGCGAGAAGTGCTT..........x11.(0.28%)
......................................TTGGCCTGCGAGAAGTGC............x1.(0.03%)
.......................................TGGCCTGCGAGAAGTGCTTCA........x38.(0.95%)
.......................................TGGCCTGCGAGAAGTGCTTC.........x4.(0.1%)
.......................................TGGCCTGCGAGAAGTGCTTCAG.......x3.(0.08%)
.........................................GCCTGCGAGAAGTGCTTCAGA......x3.(0.08%)
...........................................CTGCGAGAAGTGCTTCAGAGG....x1.(0.03%)
............................................TGCGAGAAGTGCTTCAGAGG....x12.(0.3%)
............................................TGCGAGAAGTGCTTCAGAGGT...x80.(2.01%)*
.............................................GCGAGAAGTGCTTCAGAGGT...x1.(0.03%)
..............................................CGAGAAGTGCTTCAGAGGTGG.x1.(0.03%)
..............................................CGAGAAGTGCTTCAGAGGTGGAx1.(0.03%)
```

Complementarity = 16

Folding energy = -28.70

Most common read: UUGAGGAACGUCGCGGAGUCA (freq 2320) matching esi-miR3456-5p (e-value: 0.002)

```
Query  1   UUGAGGAACGUCGCGGAGUCA  21
           |||||||||||||||||||||
Sbjct  1   UUGAGGAACGUCGCGGAGUCA  21
```

```
        10          20        30
G     A  U    A  G--      A     ACC
 CCACC CU GAGG AC   UCGCGG GUCAG   A
 GGUGG GA CUUC UG   AGCGUC CGGUU   C
A     A  -    G  AAG      -     GCG
       60          50         40
```

---

esi-MIR3461

```
GTCTTCGACGGCGAGGTTGGACTAGCAGCGGAAAATCTGGACGCGATTTTGGAGGGGCATGCAACGGTTGTATGTCCCTCCAACACCCCCTCCGTTTCTTCCGCTGCCAATCCGACCTCGCTGTAGGAGAA
.(((((.((((((((((((((...(((((((((.(..((((.......(((((((((((((((.....))))))))))))))).......))))..).)))))))))...)))))))))))))).))))).
....XXXXXXXXXXXXXXXXXXXXX....................................................................................********************..
.TCTTCGACGGCGAGGTTGGACTA...........................................................................................................x1.(0.03%)
.TCTTCGACGGCGAGGTTGGACT............................................................................................................x62.(1.93%)
.TCTTCGACGGCGAGGTTGGAC.............................................................................................................x13.(0.4%)
..CTTCGACGGCGAGGTTGGAC.............................................................................................................x1.(0.03%)
..CTTCGACGGCGAGGTTGGACT............................................................................................................x16.(0.5%)
...TTCGACGGCGAGGTTGGACTAG..........................................................................................................x5.(0.16%)
...TTCGACGGCGAGGTTGGACT............................................................................................................x15.(0.47%)
...TTCGACGGCGAGGTTGGACTA...........................................................................................................x206.(6.4%)
...TTCGACGGCGAGGTTGGA..............................................................................................................x1.(0.03%)
...TTCGACGGCGAGGTTGGACTAGC.........................................................................................................x1.(0.03%)
....TCGACGGCGAGGTTGGACTAGCA........................................................................................................x2.(0.06%)
....TCGACGGCGAGGTTGGACTAG..........................................................................................................x1934.(60.08%)*
....TCGACGGCGAGGTTGGACT............................................................................................................x17.(0.53%)
....TCGACGGCGAGGTTGGACTA...........................................................................................................x114.(3.54%)
....TCGACGGCGAGGTTGGACTAGC.........................................................................................................x17.(0.53%)
```

```
.....CGACGGCGAGGTTGGACTAGC.........................................................................................................x2.(0.06%)
......GACGGCGAGGTTGGACTAG..........................................................................................................x4.(0.12%)
......GACGGCGAGGTTGGACTA...........................................................................................................x2.(0.06%)
.........GGCGAGGTTGGACTAGCAGCG.....................................................................................................x1.(0.03%)
......................TAGCAGCGGAAAATCTGGACG........................................................................................x1.(0.03%)
......................TAGCAGCGGAAAATCTGGACGCG......................................................................................x1.(0.03%)
......................TAGCAGCGGAAAATCTGGACGC.......................................................................................x3.(0.09%)
......................TAGCAGCGGAAAATCTGGA..........................................................................................x1.(0.03%)
.......................AGCAGCGGAAAATCTGGACGCG......................................................................................x4.(0.12%)
.......................AGCAGCGGAAAATCTGGACGCGA.....................................................................................x2.(0.06%)
.......................AGCAGCGGAAAATCTGGACGC.......................................................................................x1.(0.03%)
.......................AGCAGCGGAAAATCTGGACG........................................................................................x1.(0.03%)
.........................CAGCGGAAAATCTGGACGCGA.....................................................................................x1.(0.03%)
.......................................................................................CCCTCCGTTTCTTCCGCTGC........................x5.(0.16%)
.......................................................................................CCCTCCGTTTCTTCCGCTGCCAAT....................x1.(0.03%)
........................................................................................CCTCCGTTTCTTCCGCTGCCAA.....................x16.(0.5%)
.........................................................................................CTCCGTTTCTTCCGCTGCCA......................x1.(0.03%)
...........................................................................................................CAATCCGACCTCGCTGTAGGA...x4.(0.12%)
............................................................................................................AATCCGACCTCGCTGTAGGA...x1.(0.03%)
..............................................................................................................TCCGACCTCGCTGTAGGA...x6.(0.19%)
..............................................................................................................TCCGACCTCGCTGTAGGAGAAx641.(19.91%)*
..............................................................................................................TCCGACCTCGCTGTAGGAGA.x113.(3.51%)
................................................................................................................CGACCTCGCTGTAGGAGAAx2.(0.06%)
```

Complementarity = 16

Folding energy = -78.54

Most common read: UCGACGGCGAGGUUGGACUAG (freq 1934) matching esi-miR3461-5p (e-value: 0.002)

```
Query  1   UCGACGGCGAGGUUGGACUAG  21
           |||||||||||||||||||||
Sbjct  1   UCGACGGCGAGGUUGGACUAG  21


        10        20        30        40        50        60
G     G              CUA         A UC    CGCGAUU              AC
 UCUUC ACGGCGAGGUUGGA   GCAGCGGAA A  UGGA       UUGGAGGGGCAUGCA  \
 AGAGG UGUCGCUCCAGCCU   CGUCGCCUU U  GCCU       AACCUCCCUGUAUGU  G
A     A              AAC         C UU    CCCCCAC              UG
 .       120       110       100        90        80        70

__________________________________________________
```

esi-MIR3462

```
CAAACGCGCGTCCTTGTCCCGGCATCCCGAACGTCGGGTATGTAGTGCAGCCGGGCTCGGCGCACTCGTGGTTTACAAAGCAGGAGTGAGCCGAGCCCGGCTGCACCACGCAGCCGACGTTCGGGATTCTGGGACGAGGACCCACGTTTT
.(((((.(.(((((((((((((.((((((((((((((.(.(((.(((((((((((((((((.(((((.((.((....)).)).))))).))))))))))))))))).))).).))))))))))))))).))))))))))))).).))))).
.............................................XXXXXXXXXXXXXXXXXXXXX....................*********************...........................................
............CTTGTCCCGGCATCCCGAACGT....................................................................................................................x1.(0.07%)
.............TTGTCCCGGCATCCCGAA.......................................................................................................................x5.(0.36%)
.............TTGTCCCGGCATCCCGAACG.....................................................................................................................x22.(1.58%)
.............TTGTCCCGGCATCCCGAAC......................................................................................................................x6.(0.43%)
.............TTGTCCCGGCATCCCGAACGTC...................................................................................................................x2.(0.14%)
.............TTGTCCCGGCATCCCGAACGT....................................................................................................................x552.(39.74%)
..............TGTCCCGGCATCCCGAACGTC...................................................................................................................x4.(0.29%)
..............TGTCCCGGCATCCCGAACGT....................................................................................................................x2.(0.14%)
..............TGTCCCGGCATCCCGAACG.....................................................................................................................x1.(0.07%)
.................................TCGGGTATGTAGTGCAGCCGGG...............................................................................................x6.(0.43%)
.................................TCGGGTATGTAGTGCAGCCGG................................................................................................x6.(0.43%)
..................................CGGGTATGTAGTGCAGCCGGG...............................................................................................x4.(0.29%)
.............................................TGCAGCCGGGCTCGGCGCACT....................................................................................x691.(49.75%)*
.............................................TGCAGCCGGGCTCGGCGCA......................................................................................x1.(0.07%)
.............................................TGCAGCCGGGCTCGGCGCAC.....................................................................................x2.(0.14%)
```

```
..............................................GCAGCCGGGCTCGGCGCACT....................................................................................x1.(0.07%)
......................................................................................................................GTTCGGGATTCTGGGACGAGG...........x27.(1.94%)
......................................................................................................................GTTCGGGATTCTGGGACGAGGA..........x1.(0.07%)
......................................................................................................................GTTCGGGATTCTGGGACGAG............x4.(0.29%)
.......................................................................................................................TTCGGGATTCTGGGACGAGGAC.........x3.(0.22%)
.......................................................................................................................TTCGGGATTCTGGGACGAGG...........x6.(0.43%)
.......................................................................................................................TTCGGGATTCTGGGACGAG............x1.(0.07%)
.......................................................................................................................TTCGGGATTCTGGGACGAGGA..........x38.(2.74%)*
........................................................................................................................TCGGGATTCTGGGACGAGGAC.........x3.(0.22%)
```

Complementarity = 19

Folding energy = -118.20

Most common read: UGCAGCCGGGCUCGGCGCACU (freq 691) matching mmu-miR-7648-5p (e-value: 3.2)

```
Query  6   CCGGGCUCGGCGC  18
           |||||||||||||
Sbjct  8   CCGGGCUCGGCGC  20
```

```
        10        20        30        40        50        60        70
C     C C             C              G A   A                 G     G  G  U
 AAACG G GUCCUUGUCCCGG AUCCCGAACGUCGG U UGU GUGCAGCCGGGCUCGGC CACUC UG UU A
 UUUGC C CAGGAGCAGGGUC UAGGGCUUGCAGCC A GCA CACGUCGGCCCGAGCCG GUGAG AC AA C
U     A C             U              G C   C                 A     G  G  A
.         140       130       120       110       100        90        80
```

---

esi-MIR3463

```
GCGCGACGTCATTGCTGGTGCTCGTTGGAGGCGAAGCCTTGGTGGCCAGGTGCAGCGGGCTCAGGAAGGGGCTCAGCA
((..((.(((.((.((((.((((((((.((((...((....)).)))...).)))))))))))).))..))))).)).
........XXXXXXXXXXXXXXXXXXXXX.......................*********************.....
.......GTCATTGCTGGTGCTCGTTGG..................................................x3.(0.01%)
.......GTCATTGCTGGTGCTCGTTGGA.................................................x1.(0.0%)
........TCATTGCTGGTGCTCGTTGGA.................................................x33817.(89.46%)*
........TCATTGCTGGTGCTCGTT....................................................x1.(0.0%)
........TCATTGCTGGTGCTCGTTGGAGG...............................................x10.(0.03%)
........TCATTGCTGGTGCTCGTTG...................................................x14.(0.04%)
........TCATTGCTGGTGCTCGTTGGAG................................................x750.(1.98%)
........TCATTGCTGGTGCTCGTTGG..................................................x2682.(7.09%)
.........CATTGCTGGTGCTCGTTGGA.................................................x43.(0.11%)
.........CATTGCTGGTGCTCGTTGGAG................................................x10.(0.03%)
.........CATTGCTGGTGCTCGTTGG..................................................x8.(0.02%)
..........ATTGCTGGTGCTCGTTGGAGG...............................................x1.(0.0%)
..........ATTGCTGGTGCTCGTTGGA.................................................x19.(0.05%)
..........ATTGCTGGTGCTCGTTGG..................................................x4.(0.01%)
...........TTGCTGGTGCTCGTTGGAG................................................x9.(0.02%)
...........TTGCTGGTGCTCGTTGGAGG...............................................x32.(0.08%)
...........TTGCTGGTGCTCGTTGGA.................................................x11.(0.03%)
...........TTGCTGGTGCTCGTTGGAGGC..............................................x1.(0.0%)
............TGCTGGTGCTCGTTGGAGGCG.............................................x5.(0.01%)
............TGCTGGTGCTCGTTGGAGGC..............................................x11.(0.03%)
............TGCTGGTGCTCGTTGGAG................................................x2.(0.01%)
............TGCTGGTGCTCGTTGGAGG...............................................x9.(0.02%)
..............CTGGTGCTCGTTGGAGGCGAA...........................................x1.(0.0%)
...............TGGTGCTCGTTGGAGGCGAAG..........................................x8.(0.02%)
...............TGGTGCTCGTTGGAGGCGA............................................x1.(0.0%)
...............TGGTGCTCGTTGGAGGCGAA...........................................x3.(0.01%)
..................TGCTCGTTGGAGGCGAAGCCT.......................................x2.(0.01%)
.............................GGCGAAGCCTTGGTGGCCAGG............................x1.(0.0%)
.................................AAGCCTTGGTGGCCAGGT...........................x1.(0.0%)
```

```
......................................TTGGTGGCCAGGTGCAGCG.....................x2.(0.01%)
......................................TTGGTGGCCAGGTGCAGCGGG...................x20.(0.05%)
......................................TTGGTGGCCAGGTGCAGCGG....................x25.(0.07%)
.......................................TGGTGGCCAGGTGCAGCGGG...................x3.(0.01%)
..................................................TGCAGCGGGCTCAGGAAGG.........x42.(0.11%)
..................................................TGCAGCGGGCTCAGGAAGGG........x104.(0.28%)*
..................................................TGCAGCGGGCTCAGGAAGGGG.......x144.(0.38%)
..................................................TGCAGCGGGCTCAGGAAGGGGC......x1.(0.0%)
...................................................GCAGCGGGCTCAGGAAGGGG.......x1.(0.0%)
...................................................GCAGCGGGCTCAGGAAGG.........x1.(0.0%)
```

Complementarity = 16

Folding energy = -30.00

Most common read: UCAUUGCUGGUGCUCGUUGGA (freq 33817) matching esi-miR3463-5p (e-value: 0.002)

```
Query  1   UCAUUGCUGGUGCUCGUUGGA  21
           |||||||||||||||||||||
Sbjct  1   UCAUUGCUGGUGCUCGUUGGA  21
```

```
         10         20           30
-  GC  C   A-  G    U        G ---   GAA  C
 GC  GA GUC  UU CUGG GCUCGUUG A   GGC   GC U
 CG  CU CGG  AA GACU CGGGCGAC U   CCG   UG U
A  A-  -   GG  G    -        G GGA   G--  G
         70         60        50          40
```

---

esi-MIR3464

```
AGACCCCTCGACGGTCGGAGTATGGGTTGCGATGGAATCATGGTAGATAAACATGCAGACGACGTCCAGGGGAATATGAAAACTGGACATCGTCTGCATGTTTATCTAACATGATTCCATCGCAACCCATACTCCGACGGTCGAGGGGTCT
((((((((((((.(((((((((((((((((((((((((((((.(((((((((((((((((((.((((((.............)))))).))))))))))))))))))).))))))))))))))))))))))))))))).))))))))))))
..............*********************....................................................................................................................
.......TCGACGGTCGGAGTATGGGTT...........................................................................................................................x1.(0.0%)
...........CGGTCGGAGTATGGGTTGCGA.......................................................................................................................x3.(0.01%)
.............GTCGGAGTATGGGTTGCGATG.....................................................................................................................x8.(0.02%)
.............GTCGGAGTATGGGTTGCGAT......................................................................................................................x1.(0.0%)
..............TCGGAGTATGGGTTGCGATGGA...................................................................................................................x31.(0.09%)
..............TCGGAGTATGGGTTGCGA.......................................................................................................................x6.(0.02%)
..............TCGGAGTATGGGTTGCGATGG....................................................................................................................x801.(2.34%)*
..............TCGGAGTATGGGTTGCGATG.....................................................................................................................x69.(0.2%)
..............TCGGAGTATGGGTTGCGAT......................................................................................................................x9.(0.03%)
..............TCGGAGTATGGGTTGCGATGGAA..................................................................................................................x7.(0.02%)
................GGAGTATGGGTTGCGATGG....................................................................................................................x2.(0.01%)
................................TGGAATCATGGTAGATAAACA..................................................................................................x15.(0.04%)
................................TGGAATCATGGTAGATAAACATG................................................................................................x1.(0.0%)
................................TGGAATCATGGTAGATAAAC...................................................................................................x1.(0.0%)
................................TGGAATCATGGTAGATAAACAT.................................................................................................x1.(0.0%)
....................................................................GGGGAATATGAAAACTGGACAT.............................................................x1.(0.0%)
..................................................................................................TGTTTATCTAACATGATTCCA................................x34.(0.1%)
..................................................................................................TGTTTATCTAACATGATTC..................................x2.(0.01%)
....................................................................................................TTTATCTAACATGATTCCATC..............................x4.(0.01%)
.....................................................................................................TTATCTAACATGATTCCATCG.............................x3.(0.01%)
......................................................................................................TATCTAACATGATTCCATCGCA...........................x1.(0.0%)
......................................................................................................TATCTAACATGATTCCATCGC............................x10.(0.03%)
.......................................................................................................................TCGCAACCCATACTCCGACG............x278.(0.81%)
.......................................................................................................................TCGCAACCCATACTCCGACGGT..........x105.(0.31%)
.......................................................................................................................TCGCAACCCATACTCCGACGGTC.........x8.(0.02%)
.......................................................................................................................TCGCAACCCATACTCCGA..............x18.(0.05%)
.......................................................................................................................TCGCAACCCATACTCCGACGG...........x32537.(95.1%)*
.......................................................................................................................TCGCAACCCATACTCCGAC.............x14.(0.04%)
```

```
........................................................................................................................CGCAACCCATACTCCGACG............x1.(0.0%)
........................................................................................................................CGCAACCCATACTCCGACGG...........x165.(0.48%)
........................................................................................................................CGCAACCCATACTCCGACGGT..........x1.(0.0%)
.........................................................................................................................GCAACCCATACTCCGACGG...........x72.(0.21%)
.................................................................................................................................TACTCCGACGGTCGAGGGGTC.x2.(0.01%)
```

Complementarity = 19

Folding energy = -129.12

Most common read: UCGCAACCCAUACUCCGACGG (freq 32537) matching esi-miR3464-3p (e-value: 0.002)

```
Query  1   UCGCAACCCAUACUCCGACGG  21
           |||||||||||||||||||||
Sbjct  1   UCGCAACCCAUACUCCGACGG  21
```

```
        10        20        30        40        50        60        70
            G                             G                   C      GGGAAU
AGACCCCUCGAC GUCGGAGUAUGGGUUGCGAUGGAAUCAUG UAGAUAAACAUGCAGACGA GUCCAG      \
UCUGGGGAGCUG CAGCCUCAUACCCAACGCUACCUUAGUAC AUCUAUUUGUACGUCUGCU CAGGUC      A
            G                             A                   A      AAAAGU
 .       140       130       120       110       100        90        80
```

____

```
esi-MIR3465
GCAGACCAACTGAAGATCTACCAGGACAAGAGGCGCCCCAATCGCAACTGCCCGCGCTGCAACTGGCCCCGCAGTCGCAGCGCGGGCAGTTGCGATCGGCGCACTTCTTGTCCTGGTAGATCTTCAGTTGGTCTGCA
(((((((((((((((((((((((((((((((((.((.((.((((((((((((((((((((.((((......)))).)))))))))))))))))))).)).)).))))))))))))))))))))))))))))))))).
...........................*********************.........................................................................................
..........................................................................................XXXXXXXXXXXXXXXXXXXXX..........................
....ACCAACTGAAGATCTACCAGG................................................................................................................x3.(0.14%)
....ACCAACTGAAGATCTACCAGGA...............................................................................................................x2.(0.09%)
.....CCAACTGAAGATCTACCAGGAC..............................................................................................................x1.(0.05%)
.....CCAACTGAAGATCTACCAGGA...............................................................................................................x2.(0.09%)
......CAACTGAAGATCTACCAGGACA.............................................................................................................x8.(0.38%)
......CAACTGAAGATCTACCAGGAC..............................................................................................................x20.(0.94%)*
......CAACTGAAGATCTACCAGGA...............................................................................................................x3.(0.14%)
.......AACTGAAGATCTACCAGGACA.............................................................................................................x3.(0.14%)
........ACTGAAGATCTACCAGGAC..............................................................................................................x1.(0.05%)
..........................CAAGAGGCGCCCCAATCGCAA..........................................................................................x1.(0.05%)
...........................AAGAGGCGCCCCAATCGCAAC.........................................................................................x7.(0.33%)
...........................AAGAGGCGCCCCAATCGCAA..........................................................................................x1.(0.05%)
............................AGAGGCGCCCCAATCGCAAC.........................................................................................x1.(0.05%)
................................................TGCCCGCGCTGCAACTGGCCC....................................................................x6.(0.28%)
................................................TGCCCGCGCTGCAACTGGC......................................................................x5.(0.23%)
................................................TGCCCGCGCTGCAACTGGCC.....................................................................x3.(0.14%)
.....................................................................CGCAGTCGCAGCGCGGGCAGT...............................................x1.(0.05%)
.........................................................................................TTGCGATCGGCGCACTTCTTGT..........................x1.(0.05%)
.........................................................................................TTGCGATCGGCGCACTTCTT............................x1.(0.05%)
.........................................................................................TTGCGATCGGCGCACTTCTTG...........................x30.(1.41%)
.........................................................................................TTGCGATCGGCGCACTTCTTGTC.........................x1.(0.05%)
..........................................................................................TGCGATCGGCGCACTTCTTG...........................x19.(0.89%)
..........................................................................................TGCGATCGGCGCACTTCTT............................x4.(0.19%)
..........................................................................................TGCGATCGGCGCACTTCT.............................x2.(0.09%)
..........................................................................................TGCGATCGGCGCACTTCTTGTC.........................x167.(7.83%)
..........................................................................................TGCGATCGGCGCACTTCTTGT..........................x1390.(65.2%)*
..........................................................................................TGCGATCGGCGCACTTCTTGTCC........................x99.(4.64%)
...........................................................................................GCGATCGGCGCACTTCTTGT..........................x2.(0.09%)
.............................................................................................GATCGGCGCACTTCTTGT..........................x2.(0.09%)
...............................................................................................TCGGCGCACTTCTTGTCCTGGTA...................x1.(0.05%)
........................................................................................................TTCTTGTCCTGGTAGATCTTC............x3.(0.14%)
............................................................................................................TGTCCTGGTAGATCTTCAGTT........x39.(1.83%)
..............................................................................................................TCCTGGTAGATCTTCAGTTGG......x127.(5.96%)
```

```
..............................................................................................................TCCTGGTAGATCTTCAGTTG.......x16.(0.75%)
...............................................................................................................CCTGGTAGATCTTCAGTTGGT.....x24.(1.13%)
...............................................................................................................CCTGGTAGATCTTCAGTTGG......x7.(0.33%)
................................................................................................................CTGGTAGATCTTCAGTTG.......x1.(0.05%)
................................................................................................................CTGGTAGATCTTCAGTTGGT.....x15.(0.7%)
................................................................................................................CTGGTAGATCTTCAGTTGGTC....x74.(3.47%)
.................................................................................................................TGGTAGATCTTCAGTTGGT.....x5.(0.23%)
.................................................................................................................TGGTAGATCTTCAGTTGGTCT...x11.(0.52%)
.................................................................................................................TGGTAGATCTTCAGTTGGTC....x23.(1.08%)
```

Complementarity = 17

Folding energy = -123.80

Most common read: UGCGAUCGGCGCACUUCUUGU (freq 1390) matching NONE (e-value: NONE)

```
         10        20        30        40        50        60
-                                 C  C  A                    A    GC
 GCAGACCAACUGAAGAUCUACCAGGACAAGAGG GC CC AUCGCAACUGCCCGCGCUGC ACUG  C
 CGUCUGGUUGACUUCUAGAUGGUCCUGUUCUUC CG GG UAGCGUUGACGGGCGCGACG UGAC  C
A                                 A  C  C                    C    GC
     130       120       110       100        90        80        70
```

____

esi-MIR3467

```
GCGGACGCCTTCAAGGGCCACGGGATGCTGCGGCGAGCCAAGACCATAGTCAAGGGTATGTCCCGCCGCGCCGCGCTGCGGGGATACCCTTGACTACGGTCTTGGCTCGCCGCAGCATCCCCTGGCCCTTGAAGGCGTCCGCA
((((((((((((((((((.((((((((((((((((((((((.((((((((((((.(((((.(((...))).)))))))))))))))))))).)))))))))))))))))))))).)))))))))))))))))).
.............................*********************.............................................XXXXXXXXXXXXXXXXXXXXX............................
....ACGCCTTCAAGGGCCACGGGATGC...................................................................................................................x1.(0.0%)
.......CCTTCAAGGGCCACGGGATG....................................................................................................................x1.(0.0%)
.......CCTTCAAGGGCCACGGGATGC...................................................................................................................x1.(0.0%)
.......CCTTCAAGGGCCACGGGA......................................................................................................................x6.(0.0%)
.......CCTTCAAGGGCCACGGGATGCT..................................................................................................................x34.(0.03%)
........CTTCAAGGGCCACGGGATG....................................................................................................................x13.(0.01%)
........CTTCAAGGGCCACGGGATGCTG.................................................................................................................x8.(0.01%)
........CTTCAAGGGCCACGGGAT.....................................................................................................................x4.(0.0%)
........CTTCAAGGGCCACGGGATGCT..................................................................................................................x923.(0.72%)
........CTTCAAGGGCCACGGGATGC...................................................................................................................x37.(0.03%)
.........TTCAAGGGCCACGGGATGC...................................................................................................................x34.(0.03%)
.........TTCAAGGGCCACGGGATGCT..................................................................................................................x1078.(0.84%)
.........TTCAAGGGCCACGGGATGCTG.................................................................................................................x1879.(1.46%)*
.........TTCAAGGGCCACGGGATGCTGC................................................................................................................x1.(0.0%)
.........TTCAAGGGCCACGGGATG....................................................................................................................x13.(0.01%)
..........TCAAGGGCCACGGGATGC...................................................................................................................x2.(0.0%)
..........TCAAGGGCCACGGGATGCTG.................................................................................................................x9.(0.01%)
..........TCAAGGGCCACGGGATGCT..................................................................................................................x18.(0.01%)
..........TCAAGGGCCACGGGATGCTGC................................................................................................................x1.(0.0%)
...........CAAGGGCCACGGGATGCTG.................................................................................................................x2.(0.0%)
............AAGGGCCACGGGATGCTG.................................................................................................................x3.(0.0%)
.........................TGCTGCGGCGAGCCAAGACCA.................................................................................................x5.(0.0%)
.........................TGCTGCGGCGAGCCAAGACCAT................................................................................................x1.(0.0%)
..........................GCTGCGGCGAGCCAAGACCA.................................................................................................x1.(0.0%)
............................TGCGGCGAGCCAAGACCATAGT.............................................................................................x70.(0.05%)
............................TGCGGCGAGCCAAGACCAT................................................................................................x7.(0.01%)
............................TGCGGCGAGCCAAGACCATA...............................................................................................x404.(0.31%)
............................TGCGGCGAGCCAAGACCA.................................................................................................x3.(0.0%)
............................TGCGGCGAGCCAAGACCATAGTC............................................................................................x1.(0.0%)
............................TGCGGCGAGCCAAGACCATAG..............................................................................................x1766.(1.37%)
.............................GCGGCGAGCCAAGACCATAG..............................................................................................x13.(0.01%)
```

```
.............................GCGGCGAGCCAAGACCATAGT.............................................................................................x5.(0.0%)
.............................GCGGCGAGCCAAGACCATA...............................................................................................x2.(0.0%)
.................................................TCAAGGGTATGTCCCGCCGCG.........................................................................x2.(0.0%)
.................................................TCAAGGGTATGTCCCGCCGCGCCG......................................................................x1.(0.0%)
.........................................................ATGTCCCGCCGCGCCGCGCT..................................................................x4.(0.0%)
.........................................................ATGTCCCGCCGCGCCGCG....................................................................x1.(0.0%)
...................................................................GCGCCGCGCTGCGGGGATACCCTTGA..................................................x4.(0.0%)
....................................................................CGCCGCGCTGCGGGGATACCCTTGA..................................................x1.(0.0%)
.........................................................................................TTGACTACGGTCTTGGCTCGC.................................x5.(0.0%)
.........................................................................................TTGACTACGGTCTTGGCTCGCC................................x1.(0.0%)
..........................................................................................TGACTACGGTCTTGGCTCGC.................................x1.(0.0%)
.............................................................................................CTACGGTCTTGGCTCGCCGCA.............................x15.(0.01%)
.............................................................................................CTACGGTCTTGGCTCGCCGCAG............................x18.(0.01%)
.............................................................................................CTACGGTCTTGGCTCGCCGC..............................x1.(0.0%)
..............................................................................................TACGGTCTTGGCTCGCCGCAGC...........................x637.(0.5%)
..............................................................................................TACGGTCTTGGCTCGCCGC..............................x540.(0.42%)
..............................................................................................TACGGTCTTGGCTCGCCGCAG............................x60871.(47.34%)
..............................................................................................TACGGTCTTGGCTCGCCGCAGCAT.........................x4.(0.0%)
..............................................................................................TACGGTCTTGGCTCGCCGCA.............................x59229.(46.07%)*
..............................................................................................TACGGTCTTGGCTCGCCGCAGCA..........................x189.(0.15%)
..............................................................................................TACGGTCTTGGCTCGCCG...............................x31.(0.02%)
...............................................................................................ACGGTCTTGGCTCGCCGCA.............................x173.(0.13%)
...............................................................................................ACGGTCTTGGCTCGCCGCAGC...........................x6.(0.0%)
...............................................................................................ACGGTCTTGGCTCGCCGCAG............................x261.(0.2%)
...............................................................................................ACGGTCTTGGCTCGCCGC..............................x4.(0.0%)
................................................................................................CGGTCTTGGCTCGCCGCAGC...........................x1.(0.0%)
................................................................................................CGGTCTTGGCTCGCCGCAGCA..........................x44.(0.03%)
................................................................................................CGGTCTTGGCTCGCCGCA.............................x30.(0.02%)
................................................................................................CGGTCTTGGCTCGCCGCAG............................x49.(0.04%)
.................................................................................................GGTCTTGGCTCGCCGCAG............................x13.(0.01%)
...................................................................................................TCTTGGCTCGCCGCAGCATC........................x15.(0.01%)
...................................................................................................TCTTGGCTCGCCGCAGCAT.........................x2.(0.0%)
...................................................................................................TCTTGGCTCGCCGCAGCA..........................x1.(0.0%)
...................................................................................................TCTTGGCTCGCCGCAGCATCC.......................x52.(0.04%)
...................................................................................................................CATCCCCTGGCCCTTGAAGGC.......x2.(0.0%)
...................................................................................................................CATCCCCTGGCCCTTGAAGGCG......x1.(0.0%)
...................................................................................................................CATCCCCTGGCCCTTGAA..........x2.(0.0%)
...................................................................................................................CATCCCCTGGCCCTTGAAGG........x6.(0.0%)
.....................................................................................................................TCCCCTGGCCCTTGAAGGCGT.....x6.(0.0%)
..........................................................................................................................TGGCCCTTGAAGGCGTCCGCAx2.(0.0%)
```

Complementarity = 19

Folding energy = -142.50

Most common read: UACGGUCUUGGCUCGCCGCAG (freq 60871) matching esi-miR3467-3p (e-value: 0.002)

```
Query  1   UACGGUCUUGGCUCGCCGCAG  21
           |||||||||||||||||||||
Sbjct  1   UACGGUCUUGGCUCGCCGCAG  21
```

```
         10        20        30        40        50        60        70
-                    C                        A             G      C   C
 GCGGACGCCUUCAAGGGCCA GGGAUGCUGCGGCGAGCCAAGACC UAGUCAAGGGUAU UCCCGC GCG \
 CGCCUGCGGAAGUUCCCGGU CCCUACGACGCCGCUCGGUUCUGG AUCAGUUCCCAUA GGGGCG CGC C
A                    C                        C             -      U   G
 140       130       120       110       100        90         80
```

---

esi-MIR3469

AACGTTGTGAGCTACAATCCGACCATCCTCCGCCTCTGGGGTGGATATGTGAACACCGTCGTCATCCTCGACATGCGTACTGTTGGATGGCGACGGTGTTCACATGTCCACCCCAGAGGCGGAGAGTGGTCGGATTGTAGCTCACAACGT

```
.((((((((((((((((((((((..((((((((((((((((((((((((((((((((((((((((((..((((.......))))))))))))))))))))))))))))))))))))))))))))))..))))))))))))))))))))))
.................................*********************.............................................XXXXXXXXXXXXXXXXXXXXX..............................
.......TGAGCTACAATCCGACCATCC..........................................................................................................................x16.(0.05%)
.......TGAGCTACAATCCGACCATC...........................................................................................................................x4.(0.01%)
.......TGAGCTACAATCCGACCATCCT.........................................................................................................................x2.(0.01%)
.......TGAGCTACAATCCGACCATCCTCC.......................................................................................................................x1.(0.0%)
...........CTACAATCCGACCATCCTCCGCC....................................................................................................................x1.(0.0%)
...........CTACAATCCGACCATCCTCCG......................................................................................................................x12.(0.04%)
...........CTACAATCCGACCATCCTCCGC.....................................................................................................................x3.(0.01%)
............TACAATCCGACCATCCTCCGC.....................................................................................................................x7107.(23.47%)*
............TACAATCCGACCATCCTCC.......................................................................................................................x20.(0.07%)
............TACAATCCGACCATCCTCCGCC....................................................................................................................x47.(0.16%)
............TACAATCCGACCATCCTC........................................................................................................................x5.(0.02%)
............TACAATCCGACCATCCTCCG......................................................................................................................x674.(2.23%)
............TACAATCCGACCATCCTCCGCCT...................................................................................................................x1.(0.0%)
.............ACAATCCGACCATCCTCCGC.....................................................................................................................x84.(0.28%)
.............ACAATCCGACCATCCTCCG......................................................................................................................x3.(0.01%)
..............CAATCCGACCATCCTCCGC.....................................................................................................................x8.(0.03%)
...............AATCCGACCATCCTCCGC.....................................................................................................................x4.(0.01%)
................................CCTCTGGGGTGGATATGTGAA.................................................................................................x2.(0.01%)
.................................CTCTGGGGTGGATATGTGA..................................................................................................x2.(0.01%)
.................................CTCTGGGGTGGATATGTGAAC................................................................................................x552.(1.82%)
.................................CTCTGGGGTGGATATGTGAACA...............................................................................................x25.(0.08%)
.................................CTCTGGGGTGGATATGTGAA.................................................................................................x54.(0.18%)
..................................TCTGGGGTGGATATGTGAACA...............................................................................................x177.(0.58%)
..................................TCTGGGGTGGATATGTGAACAC..............................................................................................x1.(0.0%)
..................................TCTGGGGTGGATATGTGAA.................................................................................................x17.(0.06%)
..................................TCTGGGGTGGATATGTGAAC................................................................................................x515.(1.7%)
...................................CTGGGGTGGATATGTGAAC................................................................................................x3.(0.01%)
....................................TGGGGTGGATATGTGAACA...............................................................................................x4.(0.01%)
....................................TGGGGTGGATATGTGAACAC..............................................................................................x2.(0.01%)
.................................................TGAACACCGTCGTCATCCTCGA...............................................................................x2.(0.01%)
.................................................TGAACACCGTCGTCATCCTCG................................................................................x3.(0.01%)
.................................................................CCTCGACATGCGTACTGTTGGA...............................................................x1.(0.0%)
.....................................................................GACATGCGTACTGTTGGATGGC...........................................................x2.(0.01%)
.......................................................................CATGCGTACTGTTGGATGGCGA.........................................................x1.(0.0%)
.......................................................................CATGCGTACTGTTGGATGGCGACG.......................................................x1.(0.0%)
.......................................................................CATGCGTACTGTTGGATGGCG..........................................................x2.(0.01%)
............................................................................GTACTGTTGGATGGCGAC........................................................x1.(0.0%)
................................................................................TGTTGGATGGCGACGGTGT...................................................x1.(0.0%)
................................................................................................TGTTCACATGTCCACCCCAGA.................................x92.(0.3%)
.................................................................................................GTTCACATGTCCACCCCAGA.................................x1.(0.0%)
..................................................................................................TTCACATGTCCACCCCAGAGG...............................x290.(0.96%)
..................................................................................................TTCACATGTCCACCCCAGA.................................x2.(0.01%)
..................................................................................................TTCACATGTCCACCCCAGAG................................x3.(0.01%)
...................................................................................................TCACATGTCCACCCCAGAGGC..............................x15339.(50.67%)*
...................................................................................................TCACATGTCCACCCCAGA.................................x153.(0.51%)
...................................................................................................TCACATGTCCACCCCAGAG................................x95.(0.31%)
...................................................................................................TCACATGTCCACCCCAGAGGCG.............................x15.(0.05%)
...................................................................................................TCACATGTCCACCCCAGAGG...............................x2625.(8.67%)
....................................................................................................CACATGTCCACCCCAGAGG...............................x10.(0.03%)
....................................................................................................CACATGTCCACCCCAGAGGC..............................x116.(0.38%)
.....................................................................................................ACATGTCCACCCCAGAGGC..............................x55.(0.18%)
.....................................................................................................ACATGTCCACCCCAGAGG...............................x5.(0.02%)
......................................................................................................CATGTCCACCCCAGAGGC..............................x7.(0.02%)
........................................................................................................TGTCCACCCCAGAGGCGGAGAG........................x2.(0.01%)
........................................................................................................TGTCCACCCCAGAGGCGGAGA.........................x13.(0.04%)
.....................................................................................................................GGCGGAGAGTGGTCGGATTGT............x1.(0.0%)
......................................................................................................................GCGGAGAGTGGTCGGATTGTA...........x1.(0.0%)
.......................................................................................................................CGGAGAGTGGTCGGATTGTAG..........x5.(0.02%)
.......................................................................................................................CGGAGAGTGGTCGGATTGTA...........x1.(0.0%)
.......................................................................................................................CGGAGAGTGGTCGGATTGTAGC.........x1.(0.0%)
........................................................................................................................GGAGAGTGGTCGGATTGT............x31.(0.1%)
........................................................................................................................GGAGAGTGGTCGGATTGTAGCT........x49.(0.16%)
```

```
........................................................................................................................GGAGAGTGGTCGGATTGTAG..........x156.(0.52%)
........................................................................................................................GGAGAGTGGTCGGATTGTAGC.........x1704.(5.63%)
........................................................................................................................GGAGAGTGGTCGGATTGTA...........x45.(0.15%)
.........................................................................................................................GAGAGTGGTCGGATTGTAGC.........x5.(0.02%)
.........................................................................................................................GAGAGTGGTCGGATTGTAGCT........x16.(0.05%)
.........................................................................................................................GAGAGTGGTCGGATTGTA...........x1.(0.0%)
..........................................................................................................................AGAGTGGTCGGATTGTAGC.........x1.(0.0%)
..............................................................................................................................TGGTCGGATTGTAGCTCACAAC..x3.(0.01%)
..............................................................................................................................TGGTCGGATTGTAGCTCACA....x7.(0.02%)
..............................................................................................................................TGGTCGGATTGTAGCTCACAA...x65.(0.21%)
```

Complementarity = 20

Folding energy = -137.70

Most common read: UCACAUGUCCACCCCAGAGGC (freq 15339) matching NONE (e-value: NONE)

```
        10        20        30        40        50        60        70
A                        UC                                        UC    UGC
 ACGUUGUGAGCUACAAUCCGACCA  CUCCGCCUCUGGGGUGGAUAUGUGAACACCGUCGUCAUCC  GACA   \
 UGCAACACUCGAUGUUAGGCUGGU  GAGGCGGAGACCCCACCUGUACACUUGUGGCAGCGGUAGG  UUGU   G
-                        GA                                        --    CAU
 .       140       130       120       110       100        90           80
```

Figure S4. Results of the sRNA mapping analysis for the 23 miRNA loci proposed by Billoud et al. 2014

esi-miR90*0079

```
Most common read: UAGAAGCCGAAAUCAAACCAU (freq 9)

Complementarity: 21/21

Best mirbase match: bmo-miR-3256 (e-value: 8.6)

Query  1   UAGAAGCCGAAAUCAAACC  19
           || |||| ||||||| | |
Sbjct  21  UACAAGCGGAAAUCAGAGC  3

Sequence   1 Structure      1
 ENERGY =   -94.5    1
Bases    1 to  159
```

esi-miR90*0193

No reads aligned to precursor: esi-miR90*0193

esi-miR90*0201

```
Most common read: UGGAGAAUCCGCAGGAGAUGC (freq 159)

Complementarity: 17/21

Best mirbase match: NONE (e-value: NONE)

Sequence   1 Structure      1
 ENERGY =   -148.3    1
Bases    1 to  225
```

esi-miR95*0257

No reads aligned to precursor: esi-miR95*0257

esi-miR90*0682

```
Most common read: UCGCCGACGGUUGCCGCUGC (freq 44)

Complementarity: 17/20

Best mirbase match: ath-miR404 (e-value: 9.3)

Query  2   CGCCGACGGUUGCCGCUGC  20
           ||| | ||||||| || ||
Sbjct  6   CGCUGGCGGUUGCGGCAGC  24

Sequence   1 Structure      1
 ENERGY =   -55.7    1
Bases    1 to  90
```

esi-miR90*0796

No reads aligned to precursor: esi-miR90*0796

esi-miR90*0805

```
Most common read: UCAUCGAGGACAAGCAAAGUU (freq 455)

Complementarity: 20/21

Best mirbase match: ppy-miR-376b (e-value: 7.1)

Query  1   UCAUCGAGGACAAGCAAAGUU  21
           |||| ||||| || | | |||
Sbjct  2   UCAUAGAGGAAAAUCCAUGUU  22

Sequence   1 Structure      1
 ENERGY =   -137.8    1
Bases    1 to  200
```

esi-miR90*0829

No reads aligned to precursor: esi-miR90*0829

esi-miR95*0055

```
Most common read: UGGCCGUGUUCUGACCGGGUG (freq 15)

Complementarity: 18/21

Best mirbase match: cbn-miR-35f (e-value: 8.6)

Query  3   GCCGUGUUCUGACCGGGUG  21
           ||||| || | ||| ||||
Sbjct  20  GCCGUAUUUUCACCCGGUG  2

Sequence   1 Structure      1
 ENERGY =   -98.9    1
Bases    1 to  158
```

esi-miR95*0064

```
Most common read: UCACCGCGGCGCCCUCGCCGG (freq 2420)

Complementarity: 21/21

Best mirbase match: NONE (e-value: NONE)

Sequence   1 Structure      1
 ENERGY =   -211.3    1
Bases    1 to  216
```

esi-miR95*0076

```
Most common read: UAGUGAACUCAGUCCAGGAUGG (freq 1)

Complementarity: 15/22

Best mirbase match: rno-miR-673-3p (e-value: 2.4)

Query  5   GAACUCAGUCCAGGA  19
           ||||||||||| |||
Sbjct  15  GAACUCAGUCCCGGA  1

Sequence   1 Structure      1
 ENERGY =   -153.8    1
Bases    1 to  186
```

esi-miR95*0168

```
Most common read: UAGGGCCGUCCCAUGGUGUCC (freq 1)

Complementarity: 13/21

Best mirbase match: NONE (e-value: NONE)

Sequence   1 Structure      1
 ENERGY =   -101.7    1
Bases    1 to  151
```

esi-miR95*0195

```
1       10          20          30          40          50          60          70
CCAAGAUCGGGUGCACCCGGUGGAAAGACGGCCAAGGCCAGGUAGGGCCGGGUGCCGGCCAAUCAGAUGGUGGCAUGC
((((((((((((((((((((.(.((.(((((((.((.(.((((.(((((.((((((.(((......)))))))))).)

......UCGGGUGCACCCGGUGGAAAGA.....................................................
..........UGCACCCGGUGGAAAGACGG...................................................
..........UGCACCCGGUGGAAAGACGGC..................................................
..........UGCACCCGGUGGAAAGACGGCC.................................................
..........UGCACCCGGUGGAAAGACG....................................................
..........................................................CAAUCAGAUGGUGGCAUGC
.............................................................UCAGAUGGUGGCAUGC
..................................................................UGGUGGCAUGC
.....................................................................UGGCAUGC
.....................................................................UGGCAUGC
.....................................................................UGGCAUGC
......................................................................GGCAUGC

Most common read: UGGCCGUGUUCUGACCGGGUG (freq 15)

Complementarity: 18/21

Best mirbase match: cbn-miR-35f (e-value: 8.6)

Query  3   GCCGUGUUCUGACCGGGUG  21
           ||||| || | ||| ||||
Sbjct  20  GCCGUAUUUUCACCCGGUG  2

Sequence   1 Structure      1
 ENERGY =   -89.8    1
Bases    1 to  150

       10        20        30        40         50        60
                     G A  G       A  C A    .-G     G       G   AU
CCAAGAUCGGGUGCACCCGGU G AA ACGGCCA GG C GGUA   GGCCG GUGCCG CCA  C
GGUUUUAGCCCAUGUGGGCCA U UU UGCCGGU UU G CCAU   CUGGC UACGGU GGU  A
                     G C  G       A  A C    \ -     G       -   AG
.       140       130       120       110        80         70

                                                 90
                                             GC    A  AGC
                                               UCCC GC   G
                                               GGGG CG   U
                                             --    A  AUC
                                                 100
```

```
esi-miR95*0207

1       10        20        30        40        50        60        70        80        90        100       110       120       130       140       150       160       170       180       190       200       210
CGAAAUCUGCGCCGGAACCAAGCGGCAUCCAACAUUUGUCACAGCCGUGCUCACCGCCACGGACAGUAAACCCCUGAACGGGGUUGUCAGUAGUAGCUAUUAGUGGCAUCAGCUACUGCUGUAAACCCACAGUUCAGGAGAUUGGGGUCAGUGGCAGCGAGCACGGCUGCGACAAAUGAUGGAUACCGCUUGGUUCCGGCGCAGAUUUCG
((((((((((((((((((((((((.(((((.((((((((.((((((((((.(.(((((.(((..(((.(.((((((((((((..((((((((((...........)))))))))))..))))).).)))))))).).)))..))).))))).).)))))))))))).)))))))).))))).))))))))))))))))))))))))))

CGAAAUCUGCGCCGGAACCAA......................................................................................................251536-7
CGAAAUCUGCGCCGGAACCA.......................................................................................................467446-3
CGAAAUCUGCGCCGGAAC.........................................................................................................1020809-1
.GAAAUCUGCGCCGGAACCAAG.....................................................................................................229758-8
.GAAAUCUGCGCCGGAACCAA......................................................................................................500515-3
.....UCUGCGCCGGAACCAAGCGGC.................................................................................................83654-40
.....UCUGCGCCGGAACCAAGCGG..................................................................................................477636-3
.....UCUGCGCCGGAACCAAGC....................................................................................................1806813-1
......CUGCGCCGGAACCAAGCGGC.................................................................................................718406-2
...........CCGGAACCAAGCGGCAUCCAA...........................................................................................1112366-1
...............AACCAAGCGGCAUCCAACAU........................................................................................1884237-1
.................CCAAGCGGCAUCCAACAUUU......................................................................................3164103-1
.....................GCGGCAUCCAACAUUUGUCACA................................................................................399995-3
.....................GCGGCAUCCAACAUUUGUCAC.................................................................................1293239-1
......................CGGCAUCCAACAUUUGUCACA................................................................................1425691-1
.........................CAUCCAACAUUUGUCACAGCC.............................................................................773846-2
...........................UCCAACAUUUGUCACAGCCGU...........................................................................121573-22
...........................UCCAACAUUUGUCACAGCCG............................................................................590045-2
................................CAUUUGUCACAGCCGUGCUC.......................................................................2917619-1
..................................UUUGUCACAGCCGUGCUCACC....................................................................268996-6
....................................UGUCACAGCCGUGCUCACCGC..................................................................1230867-1
......................................UCACAGCCGUGCUCACCGCCA................................................................105562-28
......................................UCACAGCCGUGCUCACCGCC.................................................................1163779-1
...............................................UGCUCACCGCCACGGACAGUA.......................................................567136-2
.................................................................................................................................CAGUUCAGGAGAUUGGGGUCAGU.......2065990-1
.................................................................................................................................CAGUUCAGGAGAUUGGGGUCA.........3151788-1
....................................................................................................................................UUCAGGAGAUUGGGGUCAGU.......1614147-1
.....................................................................................................................................UCAGGAGAUUGGGGUCAGUGG.....299384-5
.......................................................................................................................................................................UGCGACAAAUGAUGGAUACCG....835921-2
.......................................................................................................................................................................................UACCGCUUGGUUCCGGCGCAGA.....343020-4
..........................................................................................................................................................................................CGCUUGGUUCCGGCGCAGA.....699061-2
............................................................................................................................................................................................CUUGGUUCCGGCGCAGAUUUC.2673809-1
............................................................................................................................................................................................CUUGGUUCCGGCGCAGAUUU..2932131-1
.............................................................................................................................................................................................UUGGUUCCGGCGCAGAUUUCG62502-62
.............................................................................................................................................................................................UUGGUUCCGGCGCAGAUUUC.433428-3
.............................................................................................................................................................................................UUGGUUCCGGCGCAGAUU...552907-2
..............................................................................................................................................................................................UGGUUCCGGCGCAGAUUUCG67458-55
..............................................................................................................................................................................................UGGUUCCGGCGCAGAUUU..993393-1
...............................................................................................................................................................................................GGUUCCGGCGCAGAUUUCG697164-2

Most common read: UUGGUUCCGGCGCAGAUUUCG (freq 62)

Complementarity: 21/21

Best mirbase match: NONE (e-value: NONE)


Sequence   1 Structure      1
 ENERGY =  -148.9    1
Bases    1 to  210

       10        20        30        40        50        60        70         80         90       100
                         C     A         A           A C     G   AG   A C       - -     GU            AUUAG
CGAAAUCUGCGCCGGAACCAAGCGG AUCCA CAUUUGUC CAGCCGUGCUC C GCCAC GAC  UAA C CCUGAAC G GGGUU  CAGUAGUAGCU     U
GCUUUAGACGCGGCCUUGGUUCGCC UAGGU GUAAACAG GUCGGCACGAG G CGGUG CUG  GUU G GGACUUG C CCCAA  GUCGUCAUCGA     G
                         A     A         C           C A     A   GG   A A       A A     AU            CUACG
.       200       190       180       170       160       150       140       130       120       110

______________________________________________


esi-miR95*0213

1       10        20        30        40        50        60        70        80        90        100       110       120       130       140       150       160       170       180       190       200       210       220
UGAGGCCGAGCCACUGUCUCGAGCGGUCACAGGCCAUAACAGACAAGGUGCUAGGUCCGUAGCACCCUGAUGUGACCACGGUAUUUGAAUUGUCUGCAACUCACCACCUGAUUAGGCAGCAACAACGCUUGUUUUUGAGCACCGUGGCCACGUCAGGAUGCUCUGGACCUAGCACCUUCUCACGUAUGGCCUGUGGUCGCUCGUACAGUGGCUCAGCCUCUUC
.((((.(((((((((..(((((((((((((((((((...((.(((((((((((((((.((((.((((((((.((((((.......(((.((((...(((.....)))....)))))))....(((((....)))))))))))).))))))))).)))).))))))))))))))).))...))))))))))))))))))).))))))))).)))))...

..................UCGAGCGGUCACAGGCCAUAA.....................................................................................63296-61
..................UCGAGCGGUCACAGGCCAUA......................................................................................232098-8
..................UCGAGCGGUCACAGGCCAUAAC....................................................................................2394262-1
..................UCGAGCGGUCACAGGCCAU.......................................................................................2416010-1
...................CGAGCGGUCACAGGCCAUAACA...................................................................................729075-2
...................CGAGCGGUCACAGGCCAUAA.....................................................................................1405331-1
....................GAGCGGUCACAGGCCAUAA.....................................................................................2615426-1
.....................AGCGGUCACAGGCCAUAACAGA.................................................................................285675-6
.....................AGCGGUCACAGGCCAUAA.....................................................................................784517-2
.....................AGCGGUCACAGGCCAUAACA...................................................................................1108389-1
..........................UCACAGGCCAUAACAGACAAG.............................................................................621885-2
....................................UAACAGACAAGGUGCUAGGUC...................................................................232922-8
....................................UAACAGACAAGGUGCUAGGU....................................................................313885-5
........................................AGACAAGGUGCUAGGUCCGU................................................................2120197-1
.........................................GACAAGGUGCUAGGUCCGUA...............................................................2918721-1
..........................................ACAAGGUGCUAGGUCCGUAG..............................................................480710-3
................................................UGCUAGGUCCGUAGCACCCUGA.....................................................1916233-1
................................................UGCUAGGUCCGUAGCACCCU.......................................................2695821-1
..................................................CUAGGUCCGUAGCACCCUGAUG...................................................750385-2
...................................................UAGGUCCGUAGCACCCUGAUG...................................................26230-203
...................................................UAGGUCCGUAGCACCCUGAUGU..................................................689806-2
...................................................UAGGUCCGUAGCACCCUGAU.....................................................818522-2
...................................................UAGGUCCGUAGCACCCUGA......................................................2225178-1
....................................................AGGUCCGUAGCACCCUGAUG...................................................782696-2
....................................................AGGUCCGUAGCACCCUGAUGU..................................................891202-1
.......................................................UCCGUAGCACCCUGAUGUGACC..............................................34096-145
.......................................................UCCGUAGCACCCUGAUGUGAC...............................................48573-90
.......................................................UCCGUAGCACCCUGAUGUGA................................................224145-8
.......................................................UCCGUAGCACCCUGAUGUGACCA.............................................465784-3
........................................................CCGUAGCACCCUGAUGUGACC..............................................415492-3
........................................................CCGUAGCACCCUGAUGUGAC...............................................2923778-1
............................................................AGCACCCUGAUGUGACCACGG.........................................1383729-1
.............................................................GCACCCUGAUGUGACCACGGU........................................356791-4
.............................................................GCACCCUGAUGUGACCACG..........................................2525117-1
..............................................................CACCCUGAUGUGACCACGGUA.......................................54389-76
................................................................CCCUGAUGUGACCACGGUAUU.....................................393458-4
................................................................CCCUGAUGUGACCACGGUAUUUG...................................976373-1
................................................................CCCUGAUGUGACCACGGUAUUU....................................1379166-1
................................................................CCCUGAUGUGACCACGGUAU......................................2614257-1
......................................................................UGUGACCACGGUAUUUGAAUUGUCU..........................1284833-1
......................................................................................AAUUGUCUGCAACUCACCACCU..............2427664-1
...........................................................................................UCUGCAACUCACCACCUGAUUAG.......1382003-1
.............................................................................................UGCAACUCACCACCUGAUUAG.......1160015-1
..................................................................................................................................UUGAGCACCGUGGCCACGUCA.......369490-4
..................................................................................................................................UUGAGCACCGUGGCCACGUCAGGAUG..495021-3
..................................................................................................................................UUGAGCACCGUGGCCACGUCAGGA....2534063-1
...................................................................................................................................UGAGCACCGUGGCCACGUCAGGAUGC.126502-21
...................................................................................................................................UGAGCACCGUGGCCACGUCAG......349134-4
...................................................................................................................................UGAGCACCGUGGCCACGUCA.......759033-2
...................................................................................................................................UGAGCACCGUGGCCACGU.........1526146-1
...................................................................................................................................UGAGCACCGUGGCCACGUCAGGA....2339730-1
....................................................................................................................................GAGCACCGUGGCCACGUCAGGAUGC.1529336-1
.....................................................................................................................................AGCACCGUGGCCACGUCAGGAUGC.1674067-1
.....................................................................................................................................AGCACCGUGGCCACGUCAGG.....1847353-1
......................................................................................................................................GCACCGUGGCCACGUCAGGAUG..845250-2
.......................................................................................................................................CACCGUGGCCACGUCAGGAUG..422072-3
.......................................................................................................................................CACCGUGGCCACGUCAGGAUGCU.1311860-1
.......................................................................................................................................CACCGUGGCCACGUCAGGAUGC.1606484-1
........................................................................................................................................ACCGUGGCCACGUCAGGA.....1225127-1
........................................................................................................................................ACCGUGGCCACGUCAGGAUG...1299865-1
........................................................................................................................................ACCGUGGCCACGUCAGGAUGC..1782300-1
........................................................................................................................................ACCGUGGCCACGUCAGGAUGCU.2936155-1
.........................................................................................................................................CCGUGGCCACGUCAGGAUGC.1271558-1
.........................................................................................................................................CCGUGGCCACGUCAGGAUGCU.1665656-1
.........................................................................................................................................CCGUGGCCACGUCAGGAUG..2856548-1
..........................................................................................................................................CGUGGCCACGUCAGGAUG..335357-4
.............................................................................................................................................UGGCCACGUCAGGAUGCUCU.88318-37
.............................................................................................................................................UGGCCACGUCAGGAUGCUCUG.528194-2
.............................................................................................................................................UGGCCACGUCAGGAUGCUCUGG.803607-2
.............................................................................................................................................UGGCCACGUCAGGAUGCU.2832606-1
................................................................................................................................................CCACGUCAGGAUGCUCUGGAC.150589-15
................................................................................................................................................CCACGUCAGGAUGCUCUGGACC.1192285-1
................................................................................................................................................CCACGUCAGGAUGCUCUGG.2783208-1
.................................................................................................................................................CACGUCAGGAUGCUCUGGACC.1729944-1
.....................................................................................................................................................UCAGGAUGCUCUGGACCUAGC.140260-17
.....................................................................................................................................................UCAGGAUGCUCUGGACCUAGCA.2801730-1
..........................................................................................................................................................AUGCUCUGGACCUAGCACCUUCU.1614156-1
...........................................................................................................................................................UGCUCUGGACCUAGCACCUUC.109508-26
...........................................................................................................................................................UGCUCUGGACCUAGCACCUUCU.219633-8
...........................................................................................................................................................UGCUCUGGACCUAGCACCUU.841222-2
...........................................................................................................................................................UGCUCUGGACCUAGCACCUUCUC.1158021-1
.............................................................................................................................................................CUCUGGACCUAGCACCUUCUC.7534-833
.............................................................................................................................................................CUCUGGACCUAGCACCUUCU.34606-142
.............................................................................................................................................................CUCUGGACCUAGCACCUUCUCA.160077-14
.............................................................................................................................................................CUCUGGACCUAGCACCUUC.389495-4
.............................................................................................................................................................CUCUGGACCUAGCACCUU.447711-3
..............................................................................................................................................................UCUGGACCUAGCACCUUCUC.101-65900
..............................................................................................................................................................UCUGGACCUAGCACCUUCU.170-40135
..............................................................................................................................................................UCUGGACCUAGCACCUUCUCA.1745-3664
..............................................................................................................................................................UCUGGACCUAGCACCUUC.7926-789
..............................................................................................................................................................UCUGGACCUAGCACCUUCUCAC.82991-40
...............................................................................................................................................................CUGGACCUAGCACCUUCUC.41340-112
...............................................................................................................................................................CUGGACCUAGCACCUUCU.52474-80
...............................................................................................................................................................CUGGACCUAGCACCUUCUCA.196793-10
................................................................................................................................................................UGGACCUAGCACCUUCUC.83268-40
................................................................................................................................................................UGGACCUAGCACCUUCUCA.288426-5
................................................................................................................................................................UGGACCUAGCACCUUCUCACG.2762452-1
...........................................................................................................................................................................UUCUCACGUAUGGCCUGUGGU.713726-2
.................................................................................................................................................................................CGUAUGGCCUGUGGUCGCUC.2326267-1
...................................................................................................................................................................................UAUGGCCUGUGGUCGCUCGUA.496779-3
.....................................................................................................................................................................................UGGCCUGUGGUCGCUCGUACA.2218786-1
..................................................................................................................................................................................................UCGUACAGUGGCUCAGCCUCUU.395156-3
..................................................................................................................................................................................................UCGUACAGUGGCUCAGCCUCU..841348-2
..................................................................................................................................................................................................UCGUACAGUGGCUCAGCCUCUUC3163578-1
....................................................................................................................................................................................................UACAGUGGCUCAGCCUCUUC139537-17
....................................................................................................................................................................................................UACAGUGGCUCAGCCUCU..1968087-1
....................................................................................................................................................................................................UACAGUGGCUCAGCCUCUU.3134724-1

Most common read: UCUGGACCUAGCACCUUCUC (freq 65900)

Complementarity: 18/20

Best mirbase match: bta-miR-2339 (e-value: 7.7)

Query  2   CUGGACCUAGCA  13
           ||||||||||||
Sbjct  4   CUGGACCUAGCA  15


Sequence   1 Structure      1
 ENERGY =  -139.1    1
Bases    1 to  223

         10        20        30        40        50        60        70        80          90       100
U--     C          CU                   ACA  C                U    C          A       .-AUUUGAA   U    AAC-   CC
   GAGGC GAGCCACUGU  CGAGCGGUCACAGGCCAUA   GA AAGGUGCUAGGUCCG AGCA CCUGAUGUG CCACGGU          UUG CUGC    UCA  \
   CUCCG CUCGGUGACA  GCUCGCUGGUGUCCGGUAU   CU UUCCACGAUCCAGGU UCGU GGACUGCAC GGUGCCA          AAC GACG    AGU  A
CUU     A          U-                   GCA  C                C    A          C       \ -------   -    GAUU   CC
 220       210        200       190       180       170       160       150                 120        110

                                                                                          130
                                                                                      CAAC     U
                                                                                          GCUUG U
                                                                                          CGAGU U
                                                                                      ----     U
                                                                                        140

______________________________________________


esi-miR95*0295

1       10        20        30        40        50        60        70        80        90        100       110       120       130       140       150       160       170       180       190       200       210
GCGGCUGCAGGAACGGCGGCGGCCGGCGAGGGCGCCGCGGUGACCGAAGAGCCCCUCCGCGCCGACACCGGAAGGAUCUGCCUGUCUAACAACGUCGUGUGGAUCAACCGCGCACCGCGUGCCGGCGCCGGUACCGGCAGCGGGGGGGCUCUUCGGUCACCGCGGCGCCCUCGCCGGCCGCCGCCGUUCCUGCAGCCGC
(((((((((((((((((((((((((((((((((((((((((((((((((((((((((..((((...((((((.((.(.......).)).)))))))...((((((((....))).)))))))))..)))).)))))))))))))))))))))))))))))))))))))))))))))))))))))))))))

.............................GGGCGCCGCGGUGACCGAAGA........................................................................1171219-1
..............................GGCGCCGCGGUGACCGAAGA........................................................................627047-2
.......................................................UCCGCGCCGACACCGGAAGGA..............................................425015-3
.......................................................UCCGCGCCGACACCGGAAGGAUCUG...........................................1583622-1
.........................................................CGCGCCGACACCGGAAGGAUCU............................................3020997-1
...........................................................CGCCGACACCGGAAGGAUCUGCCU........................................2050897-1
.............................................................................................................ACCGCGUGCCGGCGCCGGU.........................2710752-1
..............................................................................................................CCGCGUGCCGGCGCCGGUA........................2609149-1
...............................................................................................................................ACCGGCAGCGGGGGGGCUCUUCG....1001389-1
.....................................................................................................................................AGCGGGGGGGCUCUUCG....1727140-1
......................................................................................................................................GCGGGGGGGCUCUUCGG...1822601-1
.........................................................................................................................................GGGGGGGCUCUUCGGUCACCGCGGC...2521036-1
...........................................................................................................................................GGGGCUCUUCGGUCACCGCGG...2385407-1
............................................................................................................................................GGCUCUUCGGUCACCGCGGC...1196956-1
.............................................................................................................................................GCUCUUCGGUCACCGCGGC...2200599-1
.............................................................................................................................................GCUCUUCGGUCACCGCGG....2210375-1
...............................................................................................................................................UCUUCGGUCACCGCGGCGCCC...94392-33
...............................................................................................................................................UCUUCGGUCACCGCGGCGC.....154204-15
...............................................................................................................................................UCUUCGGUCACCGCGGCGCC....253936-7
...............................................................................................................................................UCUUCGGUCACCGCGGCGCCCU..543628-2
...............................................................................................................................................UCUUCGGUCACCGCGGCG......1387736-1
................................................................................................................................................CUUCGGUCACCGCGGCGCCCU..547219-2
.................................................................................................................................................UUCGGUCACCGCGGCGCCCU..52500-80
.................................................................................................................................................UUCGGUCACCGCGGCGCCCUC.64633-59
.................................................................................................................................................UUCGGUCACCGCGGCGCC....460516-3
.................................................................................................................................................UUCGGUCACCGCGGCGCCC...828826-2
..................................................................................................................................................UCGGUCACCGCGGCGCCCUCG.70689-52
..................................................................................................................................................UCGGUCACCGCGGCGCCCUC..124735-21
..................................................................................................................................................UCGGUCACCGCGGCGCCCU...504278-3
..................................................................................................................................................UCGGUCACCGCGGCGCCC....2928335-1
...................................................................................................................................................CGGUCACCGCGGCGCCCU...2664227-1
....................................................................................................................................................GGUCACCGCGGCGCCCUC..2936148-1
.....................................................................................................................................................GUCACCGCGGCGCCCUCGCCGGCCG...2947651-1
......................................................................................................................................................UCACCGCGGCGCCCUCGCCGG.....2624-2420
......................................................................................................................................................UCACCGCGGCGCCCUCGCC.......50183-85
......................................................................................................................................................UCACCGCGGCGCCCUCGC........71039-51
......................................................................................................................................................UCACCGCGGCGCCCUCGCCG......71931-50
......................................................................................................................................................UCACCGCGGCGCCCUCGCCGGC....105805-27
.......................................................................................................................................................CACCGCGGCGCCCUCGCCGG.....251103-7
.......................................................................................................................................................CACCGCGGCGCCCUCGCC.......938572-1
.......................................................................................................................................................CACCGCGGCGCCCUCGCCG......980731-1
........................................................................................................................................................ACCGCGGCGCCCUCGCCGG.....234983-8
.................................................................................................................................................................CCUCGCCGGCCGCCGCCGUU.....2865632-1
.........................................................................................................................................................................GCCGCCGCCGUUCCUGCA.....1432667-1

Most common read: UCACCGCGGCGCCCUCGCCGG (freq 2420)

Complementarity: 21/21

Best mirbase match: NONE (e-value: NONE)


Sequence   1 Structure      1
 ENERGY =  -194.6    1
Bases    1 to  204

       10        20        30        40        50        60          70          80        90
                                                             --    AC     .-AAG       C  U UAA
GCGGCUGCAGGAACGGCGGCGGCCGGCGAGGGCGCCGCGGUGACCGAAGAGCCCCUCCGC  GCCG  ACCGG     GAUCUGC UG C   \
CGCCGACGUCCUUGCCGCCGCCGGCCGCUCCCGCGGCGCCACUGGCUUCUCGGGGGGGCG  CGGC  UGGCC     CUAGGUG GC G   C
                                                             AC    CA     \ ---       U  U CAA
  200       190       180       170       160       150       140                 100

                                                                                 110
                                                                                AAC     -   C
                                                                                   CGCCG CAC G
                                                                                   GCGGC GUG C
                                                                                ---     C   C
                                                                                 130       120

______________________________________________


esi-miR95*0324

1       10        20        30        40        50        60        70        80        90        100       110       120       130
GUAUGGCCUGUGACCGCUCGAACAGCUGUUCGGCCUCAAUGUAAUUGCCCUGGCAUGCAGUACGACAAACGCCAGGGCAAGUACGAAGAGGCUGAGCCACUCUACGAGCGGUCACAGGCCAUAAC
.((((((((((((((((((...((..(((((((((..((((.((((((((((.((........))...)))))))))).))))..))))))))))..))...))))))))))))))))))..

GUAUGGCCUGUGACCGCUCG...........................................................................................................34433-143
GUAUGGCCUGUGACCGCUC............................................................................................................198042-10
GUAUGGCCUGUGACCGCUCGA..........................................................................................................272448-6
GUAUGGCCUGUGACCGCU.............................................................................................................799461-2
.UAUGGCCUGUGACCGCUCG...........................................................................................................7030-892
.UAUGGCCUGUGACCGCUC............................................................................................................22055-253
.UAUGGCCUGUGACCGCUCGA..........................................................................................................34190-144
.UAUGGCCUGUGACCGCUCGAA.........................................................................................................45778-97
..AUGGCCUGUGACCGCUCG...........................................................................................................64364-59
..AUGGCCUGUGACCGCUCGA..........................................................................................................512856-3
...UGGCCUGUGACCGCUCGA..........................................................................................................479599-3
.............................................UUGCCCUGGCAUGCAGUACGA............................................................2308335-1
...............................................................................................................UACGAGCGGUCACAGGCCAUA..23244-236
...............................................................................................................UACGAGCGGUCACAGGCCAUAA.318942-5
...............................................................................................................UACGAGCGGUCACAGGCCAU...385883-4
................................................................................................................CGAGCGGUCACAGGCCAUAA.1405331-1
.................................................................................................................GAGCGGUCACAGGCCAUAA.2615426-1
..................................................................................................................AGCGGUCACAGGCCAUAA.784517-2

Most common read: UAUGGCCUGUGACCGCUCG (freq 892)

Complementarity: 19/19

Best mirbase match: oan-miR-1421l-1-3p (e-value: 3.9)

Query  1   UAUGGCCUGUGACCGCUC  18
           |||  | |||||||||||
Sbjct  3   UAUCCCAUGUGACCGCUC  20


Sequence   1 Structure      1
 ENERGY =   -81.5    1
Bases    1 to  125

        10        20        30        40        50          60
G-                    AAC  CU          AA    A          A--  CAG
  UAUGGCCUGUGACCGCUCG   AG  GUUCGGCCUC  UGUA UUGCCCUGGC   UG   U
  AUACCGGACACUGGCGAGC   UC  CGAGUCGGAG  GCAU AACGGGACCG   AC   A
CA                    AUC  AC          AA    G          CAA  AGC
   120       110       100        90        80        70

______________________________________________


esi-miR95*0365

1       10        20        30        40        50        60        70        80        90        100       110       120       130       140       150       160       170       180       190       200       210       220
ACACACUGAUUCUCGGAAGCUAUCAACUUUGCUCGUCCUCGAUGAAUUGGUCCUGAUAUGUGCGUGAACGUUUUUUGGUGGAAGCAAGAGAUUUGGGUCUGAUUCGGCUGAAGAACCGGCCGCAGGCUCCAACUUCAAGCUUUUCGAAAACACGCUCACGCACAUAUCGGGACCAAUUCAUCGAGGACAAGCAAAGUUGAUAGCUUCCGAGAAUCAGUGUGU
((((((((((((((((((((((((((((((.((((((((((((((((((((((((((((((((((.(((.((((.(.((((((..(((..(((((((((...(((((......))))))))))).)))).)))..))))))).)))).))).)))))))))))))))))))))))))))))))))))))).)))))))))))))))))))))))))))))))

....ACUGAUUCUCGGAAGCUAUCA.......................................................................................................3014054-1
......UGAUUCUCGGAAGCUAUCAAC.....................................................................................................193146-10
......UGAUUCUCGGAAGCUAUCAA......................................................................................................822214-2
.......GAUUCUCGGAAGCUAUCAACU....................................................................................................2075649-1
.........UUCUCGGAAGCUAUCAACUUU..................................................................................................966352-1
..........UCUCGGAAGCUAUCAACUUUG.................................................................................................430289-3
..........UCUCGGAAGCUAUCAACUUUGCUCG.............................................................................................2763219-1
...........CUCGGAAGCUAUCAACUUUGC................................................................................................1849927-1
............UCGGAAGCUAUCAACUUUGCU...............................................................................................375558-4
............UCGGAAGCUAUCAACUUUGC................................................................................................850796-2
...................CUAUCAACUUUGCUCGUCCUC........................................................................................1766178-1
....................UAUCAACUUUGCUCGUCCUCG.......................................................................................129322-20
....................UAUCAACUUUGCUCGUCCU.........................................................................................1570015-1
.....................AUCAACUUUGCUCGUCCUCG.......................................................................................982588-1
......................UCAACUUUGCUCGUCCUCGAU.....................................................................................45371-99
......................UCAACUUUGCUCGUCCUCG.......................................................................................526795-2
......................UCAACUUUGCUCGUCCUCGA......................................................................................2991866-1
.......................CAACUUUGCUCGUCCUCGAU.....................................................................................1275996-1
........................AACUUUGCUCGUCCUCGAU.....................................................................................3144097-1
.........................ACUUUGCUCGUCCUCGAUGA...................................................................................1738376-1
...........................UUUGCUCGUCCUCGAUGAAUU................................................................................2100239-1
............................UUGCUCGUCCUCGAUGAAUUG...............................................................................278146-6
............................UUGCUCGUCCUCGAUGAAUUGG..............................................................................696820-2
............................UUGCUCGUCCUCGAUGAAU.................................................................................1136545-1
.............................UGCUCGUCCUCGAUGAAUUGG..............................................................................219485-8
.............................UGCUCGUCCUCGAUGAAUUG...............................................................................2865854-1
................................UCGUCCUCGAUGAAUUGGUCC...........................................................................658067-2
................................UCGUCCUCGAUGAAUUGGU.............................................................................818887-2
...................................UCCUCGAUGAAUUGGUCCUGA........................................................................380055-4
......................................UCGAUGAAUUGGUCCUGAUAU.....................................................................707178-2
..........................................UGAAUUGGUCCUGAUAUGUGC.................................................................1119636-1
..............................................UUGGUCCUGAUAUGUGCGUGA.............................................................14607-408
..............................................UUGGUCCUGAUAUGUGCGUGAA............................................................673476-2
...............................................UGGUCCUGAUAUGUGCGUGA.............................................................31433-162
...............................................UGGUCCUGAUAUGUGCGUGAA............................................................302842-5
...............................................UGGUCCUGAUAUGUGCGUG..............................................................2764588-1
................................................GGUCCUGAUAUGUGCGUGA.............................................................641786-2
..................................................UCCUGAUAUGUGCGUGAACGU.........................................................551132-2
.....................................................UGAUAUGUGCGUGAACGUUU.......................................................757459-2
..........................................................UGUGCGUGAACGUUUUUUGGUG..............................................2160108-1
..........................................................UGUGCGUGAACGUUUUUUGGUGG.............................................3122714-1
...................................................................................................................................................UUUCGAAAACACGCUCACGCA.....................257744-7
.....................................................................................................................................................UCGAAAACACGCUCACGCACA...................199515-10
.........................................................................................................................................................................UCGGGACCAAUUCAUCGAGGA.............143566-17
.........................................................................................................................................................................UCGGGACCAAUUCAUCGAGG..............2031628-1
..................................................................................................................................................................................UUCAUCGAGGACAAGCAAAGU.......56283-72
..................................................................................................................................................................................UUCAUCGAGGACAAGCAAA.........1180585-1
...................................................................................................................................................................................UCAUCGAGGACAAGCAAAGUU......13290-455
...................................................................................................................................................................................UCAUCGAGGACAAGCAAAGU.......488224-3
...................................................................................................................................................................................UCAUCGAGGACAAGCAAA.........2101416-1
....................................................................................................................................................................................CAUCGAGGACAAGCAAAGUUG.....669457-2
....................................................................................................................................................................................CAUCGAGGACAAGCAAAGUU......1081804-1
......................................................................................................................................................................................UCGAGGACAAGCAAAGUUGAU...1833746-1
.....................................................................................................................................................................................................UUGAUAGCUUCCGAGAAUCAG.....777202-2
.....................................................................................................................................................................................................UUGAUAGCUUCCGAGAAUCAGU....1450510-1
......................................................................................................................................................................................................UGAUAGCUUCCGAGAAUCAGU....196642-10
........................................................................................................................................................................................................AUAGCUUCCGAGAAUCAGUGU..2509078-1
.........................................................................................................................................................................................................UAGCUUCCGAGAAUCAGUGUG.137322-18

Most common read: UCAUCGAGGACAAGCAAAGUU (freq 455)

Complementarity: 20/21

Best mirbase match: ppy-miR-376b (e-value: 7.1)

Query  1   UCAUCGAGGACAAGCAAAGUU  21
           |||| ||||| || | | |||
Sbjct  2   UCAUAGAGGAAAAUCCAUGUU  22


Sequence   1 Structure      1
 ENERGY =  -159.1    1
Bases    1 to  222

       10        20        30        40        50        60        70        80        90        100       110
                                 C                                  A   U    G U      AA   AU    -     AUU      AA
ACACACUGAUUCUCGGAAGCUAUCAACUUUGCU GUCCUCGAUGAAUUGGUCCUGAUAUGUGCGUGA CGU UUUU G GGAAGC  GAG  UUGG GUCUG   CGGCUG  G
UGUGUGACUAAGAGCCUUCGAUAGUUGAAACGA CAGGAGCUACUUAACCAGGGCUAUACACGCACU GCA AAAA C UUUUCG  CUU  AACC CGGAC   GCCGGC  A
                                 A                                  C   C    G -      AA   C-    U     ---      CA
220       210       200       190       180       170       160       150       140       130         120

______________________________________________


esi-miR95*0483

1       10        20        30        40        50        60        70        80        90        100       110       120       130       140       150       160       170       180       190       200       210       220
ACACACUGAUUCUCGGAAGCUAUCAACUUUGCUCGUCCUCGAUGAAUUGGUCCUGAUAUGUGCGUGAACGUUUUUUGGUGGAAGCAAGAGAUUUGGGUCUGAUUCGGCUGAAGAACCGGCCGCAGGCUCCAACUUCAAGCUUUUCGAAAACACGCUCACGCACAUAUCGGGACCAAUUCAUCGAGGACAAGCAAAGUUGAUAGCUUCCGAGAAUCAGUGUGU
((((((((((((((((((((((((((((((.((((((((((((((((((((((((((((((((((.(((.((((.(.((((((..(((..(((((((((...(((((......))))))))))).)))).)))..))))))).)))).))).)))))))))))))))))))))))))))))))))))))).)))))))))))))))))))))))))))))))

....ACUGAUUCUCGGAAGCUAUCA.......................................................................................................3014054-1
......UGAUUCUCGGAAGCUAUCAAC.....................................................................................................193146-10
......UGAUUCUCGGAAGCUAUCAA......................................................................................................822214-2
.......GAUUCUCGGAAGCUAUCAACU....................................................................................................2075649-1
.........UUCUCGGAAGCUAUCAACUUU..................................................................................................966352-1
..........UCUCGGAAGCUAUCAACUUUG.................................................................................................430289-3
..........UCUCGGAAGCUAUCAACUUUGCUCG.............................................................................................2763219-1
...........CUCGGAAGCUAUCAACUUUGC................................................................................................1849927-1
............UCGGAAGCUAUCAACUUUGCU...............................................................................................375558-4
............UCGGAAGCUAUCAACUUUGC................................................................................................850796-2
...................CUAUCAACUUUGCUCGUCCUC........................................................................................1766178-1
....................UAUCAACUUUGCUCGUCCUCG.......................................................................................129322-20
....................UAUCAACUUUGCUCGUCCU.........................................................................................1570015-1
.....................AUCAACUUUGCUCGUCCUCG.......................................................................................982588-1
......................UCAACUUUGCUCGUCCUCGAU.....................................................................................45371-99
......................UCAACUUUGCUCGUCCUCG.......................................................................................526795-2
......................UCAACUUUGCUCGUCCUCGA......................................................................................2991866-1
.......................CAACUUUGCUCGUCCUCGAU.....................................................................................1275996-1
........................AACUUUGCUCGUCCUCGAU.....................................................................................3144097-1
.........................ACUUUGCUCGUCCUCGAUGA...................................................................................1738376-1
...........................UUUGCUCGUCCUCGAUGAAUU................................................................................2100239-1
............................UUGCUCGUCCUCGAUGAAUUG...............................................................................278146-6
............................UUGCUCGUCCUCGAUGAAUUGG..............................................................................696820-2
............................UUGCUCGUCCUCGAUGAAU.................................................................................1136545-1
.............................UGCUCGUCCUCGAUGAAUUGG..............................................................................219485-8
.............................UGCUCGUCCUCGAUGAAUUG...............................................................................2865854-1
................................UCGUCCUCGAUGAAUUGGUCC...........................................................................658067-2
................................UCGUCCUCGAUGAAUUGGU.............................................................................818887-2
...................................UCCUCGAUGAAUUGGUCCUGA........................................................................380055-4
......................................UCGAUGAAUUGGUCCUGAUAU.....................................................................707178-2
..........................................UGAAUUGGUCCUGAUAUGUGC.................................................................1119636-1
..............................................UUGGUCCUGAUAUGUGCGUGA.............................................................14607-408
..............................................UUGGUCCUGAUAUGUGCGUGAA............................................................673476-2
...............................................UGGUCCUGAUAUGUGCGUGA.............................................................31433-162
...............................................UGGUCCUGAUAUGUGCGUGAA............................................................302842-5
...............................................UGGUCCUGAUAUGUGCGUG..............................................................2764588-1
................................................GGUCCUGAUAUGUGCGUGA.............................................................641786-2
..................................................UCCUGAUAUGUGCGUGAACGU.........................................................551132-2
.....................................................UGAUAUGUGCGUGAACGUUU.......................................................757459-2
..........................................................UGUGCGUGAACGUUUUUUGGUG..............................................2160108-1
..........................................................UGUGCGUGAACGUUUUUUGGUGG.............................................3122714-1
...................................................................................................................................................UUUCGAAAACACGCUCACGCA.....................257744-7
.....................................................................................................................................................UCGAAAACACGCUCACGCACA...................199515-10
.........................................................................................................................................................................UCGGGACCAAUUCAUCGAGGA.............143566-17
.........................................................................................................................................................................UCGGGACCAAUUCAUCGAGG..............2031628-1
..................................................................................................................................................................................UUCAUCGAGGACAAGCAAAGU.......56283-72
..................................................................................................................................................................................UUCAUCGAGGACAAGCAAA.........1180585-1
...................................................................................................................................................................................UCAUCGAGGACAAGCAAAGUU......13290-455
...................................................................................................................................................................................UCAUCGAGGACAAGCAAAGU.......488224-3
...................................................................................................................................................................................UCAUCGAGGACAAGCAAA.........2101416-1
....................................................................................................................................................................................CAUCGAGGACAAGCAAAGUUG.....669457-2
....................................................................................................................................................................................CAUCGAGGACAAGCAAAGUU......1081804-1
......................................................................................................................................................................................UCGAGGACAAGCAAAGUUGAU...1833746-1
.....................................................................................................................................................................................................UUGAUAGCUUCCGAGAAUCAG.....777202-2
.....................................................................................................................................................................................................UUGAUAGCUUCCGAGAAUCAGU....1450510-1
......................................................................................................................................................................................................UGAUAGCUUCCGAGAAUCAGU....196642-10
........................................................................................................................................................................................................AUAGCUUCCGAGAAUCAGUGU..2509078-1
.........................................................................................................................................................................................................UAGCUUCCGAGAAUCAGUGUG.137322-18

Most common read: UCAUCGAGGACAAGCAAAGUU (freq 455)

Complementarity: 20/21

Best mirbase match: ppy-miR-376b (e-value: 7.1)

Query  1   UCAUCGAGGACAAGCAAAGUU  21
           |||| ||||| || | | |||
Sbjct  2   UCAUAGAGGAAAAUCCAUGUU  22


Sequence   1 Structure      1
 ENERGY =  -159.1    1
Bases    1 to  222

       10        20        30        40        50        60        70        80        90        100       110
                                 C                                  A   U    G U      AA   AU    -     AUU      AA
ACACACUGAUUCUCGGAAGCUAUCAACUUUGCU GUCCUCGAUGAAUUGGUCCUGAUAUGUGCGUGA CGU UUUU G GGAAGC  GAG  UUGG GUCUG   CGGCUG  G
UGUGUGACUAAGAGCCUUCGAUAGUUGAAACGA CAGGAGCUACUUAACCAGGGCUAUACACGCACU GCA AAAA C UUUUCG  CUU  AACC CGGAC   GCCGGC  A
                                 A                                  C   C    G -      AA   C-    U     ---      CA
220       210       200       190       180       170       160       150       140       130         120

______________________________________________


esi-miR95*0400

1       10        20        30        40        50        60        70        80        90        100       110       120       130       140       150       160       170       180       190       200
CGCAAAUCCAGGCCAUCUUGGUUCGGACUCAGGAGGAAUGAUAUCCGUCAUACGAACCCCGGAUCACGGACGAGGGUUUCGUGAUGUCGUCAGGACGGAAGCUUCCCUAUUGGCGAGAUCGCGCCCCCUCCUCCGUGACCCGGACGUCGUAUGACGAGUCAUUCCUCCGGAACCCAAACCAAGAUGGCCUGGAUUUGCG
((((((((((((((((((((.((..((.((((((((((((...(((((((((...((((.(((((((.((((...((((((.((((((((...(((....)))...))))))).))))))..))))).))))))).)))))...)))))))))).))))))))))).))..)).))))))))))))))))))))))

..............UCUUGGUUCGGACUCAGGAGG.........................................................................................................2664759-1
..............................................................................................................................................................................UCCGGAACCCAAACCAAGAUG............278164-6

Most common read: UCCGGAACCCAAACCAAGAUG (freq 6)

Complementarity: 17/21

Best mirbase match: rrv-miR-rR1-5 (e-value: 7.1)

Query  2   CCGGAACCCAAA  13
           ||||||||||||
Sbjct  1   CCGGAACCCAAA  12


Sequence   1 Structure      1
 ENERGY =  -135.9    1
Bases    1 to  199

       10        20        30        40        50        60        70        80        90       100
                     C  AC  A           AUC          ACC    A       C     UUU      G       GAC   A
CGCAAAUCCAGGCCAUCUUGGUU GG  UC GGAGGAAUGAU   CGUCAUACGA   CCGG UCACGGA GAGGG   CGUGAU UCGUCAG   GGA G
GCGUUUAGGUCCGGUAGAACCAA CC  AG CCUCCUUACUG   GCAGUAUGCU   GGCC AGUGCCU CUCCC   GCGCUA AGCGGUU   CCU C
                     A  CA  G           A--          GCA    C       C     CC-      G       AUC   U
      190       180       170       160         150       140       130       120       110

______________________________________________


esi-miR95*0424

1       10        20        30        40        50        60        70        80        90        100       110       120       130       140       150       160       170       180       190       200       210
CCGAAACUGGGCCGGAACCAAGCGGCAUCCAACAUUGGUCUCAGCCGUGCUCGCCGCGACUGACCCCAAUCUCCUGAACUGCGGGUGUAGAGCAGUAGCUGAUGCCACUAGCUACUGCUGGCACUCCGUCCGUUCAGGGGCUUACGGUCCGUGGCGGUGAGUAUGGCUGUGAAAAAAUAUUGGAUGCCGCUUGGUUCCGGCGUAGAUCUCGG
((((..(((.((((((((((((((((((((((.(((..((.((((((((((((((((.((.((((..((.((((((((.(((((((..(((((((((((.......))))))))))).)))).)))).)))))))))).)).)))).)).)))))))))))))))).))..)).))))))))))))))))))))))).)))...))))

..........CCGGAACCAAGCGGCAUCCAA.............................................................................................................1112366-1
..............AACCAAGCGGCAUCCAACAU..........................................................................................................1884237-1
.............................................UGCUCGCCGCGACUGACCCCA.........................................................................2096030-1
..........................................................CUGACCCCAAUCUCCUGAACUGC..........................................................363590-4
..........................................................CUGACCCCAAUCUCCUGAACUGCGG........................................................405256-3
..........................................................CUGACCCCAAUCUCCUGAACUG...........................................................446979-3
..........................................................CUGACCCCAAUCUCCUGAACU............................................................1720116-1
...........................................................UGACCCCAAUCUCCUGAACUGC..........................................................30424-169
...........................................................UGACCCCAAUCUCCUGAACUGCG.........................................................80599-42
...........................................................UGACCCCAAUCUCCUGAACUG...........................................................91187-35
...........................................................UGACCCCAAUCUCCUGAACUGCGG........................................................188416-11
...........................................................UGACCCCAAUCUCCUGAACU............................................................540892-2
............................................................GACCCCAAUCUCCUGAACUGC..........................................................794532-2
...............................................................CCCCAAUCUCCUGAACUGCGG........................................................41269-112
...............................................................CCCCAAUCUCCUGAACUGCG.........................................................264247-6
...............................................................CCCCAAUCUCCUGAACUGC..........................................................793665-2
...............................................................CCCCAAUCUCCUGAACUGCGGG.......................................................1232821-1
...............................................................CCCCAAUCUCCUGAACUG...........................................................1749722-1
................................................................CCCAAUCUCCUGAACUGCGGG.......................................................343076-4
................................................................CCCAAUCUCCUGAACUGCGG........................................................2250504-1
.................................................................CCAAUCUCCUGAACUGCGG........................................................160519-14
............................................................................................................................................UCCGUGGCGGUGAGUAUGGCU.........197546-10
.................................................................................................................................................................UGAAAAAAUAUUGGAUGCCGCU.....2170889-1
........................................................................................................................................................................................UUGGAUGCCGCUUGGUUCCGG......73442-49
.........................................................................................................................................................................................UGGAUGCCGCUUGGUUCCGG......185516-11
.........................................................................................................................................................................................UGGAUGCCGCUUGGUUCCGGC.....383962-4

Most common read: UGACCCCAAUCUCCUGAACUGC (freq 169)

Complementarity: 17/22

Best mirbase match: osa-miR815c (e-value: 6.4)

Query  5   CCCAAUCUCCUGAA  18
           ||||||||||| ||
Sbjct  21  CCCAAUCUCCUCAA  8


Sequence   1 Structure      1
 ENERGY =  -146.4    1
Bases    1 to  211

        10        20        30        40        50        60        70        80          90       100
    AA-   G                     C   GG  U                G  U    CC  U         U-    -    AG           AUG
CCGA   CUG GCCGGAACCAAGCGGCAUCCAA AUU  UC CAGCCGUGCUCGCCGC AC GACC  AA CUCCUGAAC  GCGG GUGU  AGCAGUAGCUG   \
GGCU   GAU CGGCCUUGGUUCGCCGUAGGUU UAA  AG GUCGGUAUGAGUGGCG UG CUGG  UU GGGGACUUG  UGCC CACG  UCGUCAUCGAU   C
    CUA   G                     A   AA  U                G  C    CA  C         CC    U    G-           CAC
 .       200       190       180       170       160       150       140       130       120        110

______________________________________________


esi-miR95*0439


**********************
 No reads aligned to precursor: esi-miR95*0439
**********************




esi-miR95*0485

1       10        20        30        40        50        60        70        80        90        100       110       120       130       140       150       160       170
CCGCCCUGAAGCGCCAGCCGCGCGACGCAAGCCGCAAUCGAAAAGGUAAGCCAGCGCGACAAGCAACUGAAUCAACGGGGUUGUCCAUCUCGUUGACCGUUGCUUGUCAGUCUGGCUUACCUUUUCGAUUGCGGGUGAUGUCGCGUGGCUGGCGCCCCAGGGCGG
((((((((..((((((((((((((((..(.((((((((((((((((((((((((.((((((((((((.(..((((((((((.....)))))))))).))))))))))).)))))))))))))))))))))))))).)..))))))))))))))))..))))))))

..........................................................................................................UCAGUCUGGCUUACCUUUUCGA....................................194654-10
..........................................................................................................UCAGUCUGGCUUACCUUUUCG.....................................1868699-1
................................................................................................................................UUGCGGGUGAUGUCGCGUGG................187298-11
................................................................................................................................UUGCGGGUGAUGUCGCGUGGC...............421216-3
.....................................................................................................................................GGUGAUGUCGCGUGGCUGG............1431248-1

Most common read: UUGCGGGUGAUGUCGCGUGG (freq 11)

Complementarity: 17/20

Best mirbase match: NONE (e-value: NONE)


Sequence   1 Structure      1
 ENERGY =  -129.8    1
Bases    1 to  165

       10        20        30        40        50         60        70        80
       AA                CA G                     C  -          U AA          UG
CCGCCCUG  GCGCCAGCCGCGCGACG  A CCGCAAUCGAAAAGGUAAGCCAG GC GACAAGCAAC G  UCAACGGGGU  \
GGCGGGAC  CGCGGUCGGUGCGCUGU  U GGCGUUAGCUUUUCCAUUCGGUC UG CUGUUCGUUG C  AGUUGCUCUA  U
       CC                AG G                     -  A          C --          CC
   160       150       140       130       120       110       100         90

______________________________________________
```
