# Supplementary material for: microRNAs and the evolution of complex multicellularity: identification of a large, diverse complement of microRNAs in the brown alga Ectocarpus
Source: Nucleic Acids Res. 2015 Jun 22;43(13):6384–98. doi: 10.1093/nar/gkv578 (PMC4513859; doi:10.1093/nar/gkv578)
Supplement: SUPPLEMENTARY DATA [file supp_gkv578_FigS1_sRNA_mapping.pdf]

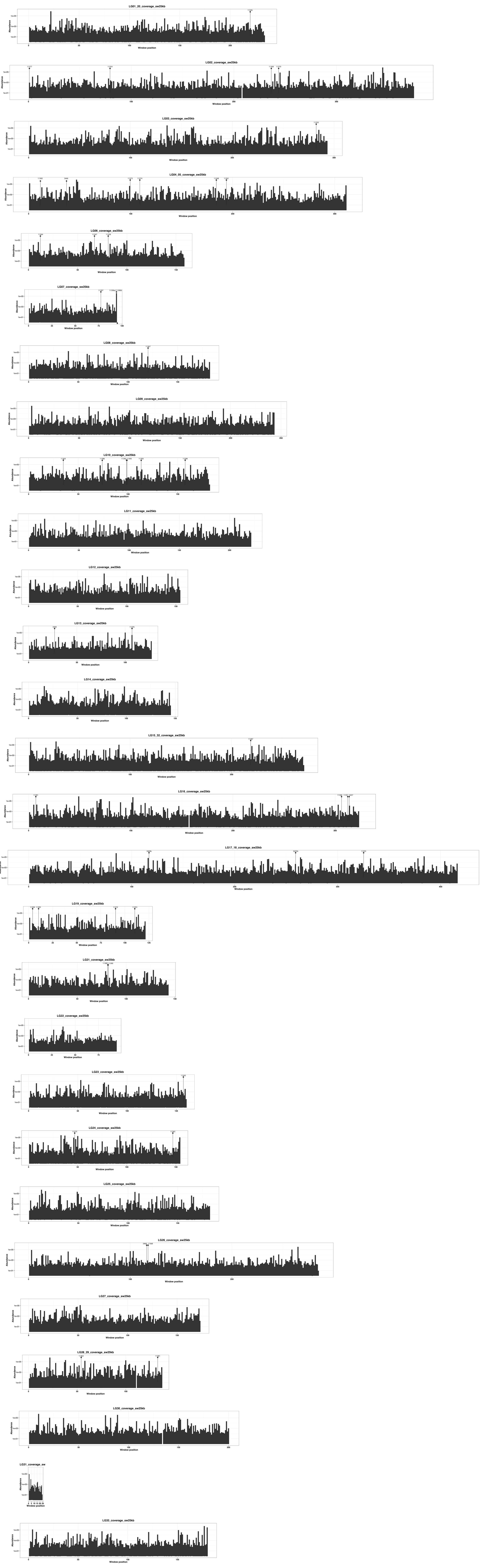
LG01_20_coverage_sw25kb
LG02_coverage_sw25kb
LG03_coverage_sw25kb
LG04_05_coverage_sw25kb
LG06_coverage_sw25kb
LG07_coverage_sw25kb
LG08_coverage_sw25kb
LG09_coverage_sw25kb
LG10_coverage_sw25kb
LG11_coverage_sw25kb
LG12_coverage_sw25kb
LG13_coverage_sw25kb
LG14_coverage_sw25kb
LG15_32_coverage_sw25kb
LG16_coverage_sw25kb
LG17_18_coverage_sw25kb
LG19_coverage_sw25kb
LG21_coverage_sw25kb
LG22_coverage_sw25kb
LG23_coverage_sw25kb
LG24_coverage_sw25kb
LG25_coverage_sw25kb
LG26_coverage_sw25kb
LG27_coverage_sw25kb
LG28_29_coverage_sw25kb
LG30_coverage_sw25kb
LG31_coverage_sw
LG33_coverage_sw25kb
Window position
Abundance
